# An insight into the phylogenetic history of *HOX *linked gene families in vertebrates

**DOI:** 10.1186/1471-2148-7-239

**Published:** 2007-11-30

**Authors:** Amir Ali Abbasi, Karl-Heinz Grzeschik

**Affiliations:** 1 Institute of Human Genetics, Philipps-University, Bahnhofstrasse 7 D35037 Marburg, Germany

## Abstract

**Background:**

The human chromosomes 2q, 7, 12q and 17q show extensive intra-genomic homology, containing duplicate, triplicate and quadruplicate paralogous regions centered on the HOX gene clusters. The fact that two or more representatives of different gene families are linked with HOX clusters is taken as evidence that these paralogous gene sets might have arisen from a single chromosomal segment through block or whole chromosome duplication events. This would imply that the constituent genes including the HOX clusters reflect the architecture of a single ancestral block (before vertebrate origin) where all of these genes were linked in a single copy.

**Results:**

In the present study we have employed the currently available set of protein data for a wide variety of vertebrate and invertebrate genomes to analyze the phylogenetic history of 11 multigene families with three or more of their representatives linked to human HOX clusters. A topology comparison approach revealed four discrete co-duplicated groups: group 1 involves the genes from GLI, HH, INHB, IGFBP (cluster-1), and SLC4A families; group 2 involves ERBB, ZNFN1A, and IGFBP (cluster-2) gene families; group 3 involves the HOX clusters and the SP gene family; group 4 involves the integrin beta chain and myosine light chain families. The distinct genes within each co-duplicated group share the same evolutionary history and are duplicated in concert with each other, while the constituent genes of two different co-duplicated groups may not share their evolutionary history and may not have duplicated simultaneously.

**Conclusion:**

We conclude that co-duplicated groups may themselves be remnants of ancient small-scale duplications (involving chromosomal segments or gene-clusters) which occurred at different time points during chordate evolution. Whereas the recent combination of genes from distinct co-duplicated groups on different chromosomal regions (human chromosomes 2q, 7, 12q, and 17q) is probably the outcome of subsequent rearrangement of genomic segments, including syntenic groups of genes.

## Background

During the evolutionary history of life on Earth there has been a trend towards drastic transitions from simple to more complex life forms, like from unicellular bacterium to simple multicellular Placozans, diploblastic organisms with two germ layers to bilaterians with a third germ layer, simple chordates to vertebrates [1]. The innovation of new structures and functions during these macroevolutionary events has in part been accomplished through expansion in the genetic toolkit, e.g. by gene duplications [2]. In fact, extensive gene duplications have been suggested at the base of vertebrate lineage which results in widespread existence of gene families in modern vertebrates [3-6]. Expansions in gene number are associated with the evolution of increased morphological and anatomical complexity and diversity achieved by vertebrates compared to basal chordates (cephalochordates/tunicates). The organization of paralogous regions (paralogons) in the human and other vertebrate genomes have led to the hypothesis of multiple block duplication events involving large chromosomal segments or even two rounds of whole genome duplication (2R hypothesis) [7-11] early in the history of vertebrate evolution after their divergence from an amphioxus-like invertebrate ancestor. In contrast to block duplication events, an alternative model of continuous wave of small-scale gene duplications (involving single genes or chromosomal segments) was suggested to explain the numerous paralogs in vertebrates [12-14].

Phylogenetic trees can be used to test the 2R hypothesis. If two rounds of genome duplication occurred, a tree for four vertebrate paralogous genes should exhibit the topology of the form (AB)(CD), where the first genome duplication produced the common ancestor of the sequences A/B and C/D and the second genome duplication split these two lineages simultaneously. Thus, under the assumption of the 2R hypothesis the neighboring gene families within potentially quadruplicated regions of the human genome should not only show the same but also the specific type of topology [13]. Nevertheless many phylogenetic analyses have not yielded a predominance of (AB)(CD) topologies, instead a high proportion of gene families showed an asymmetrical (A)(BCD) tree, in which one of the four paralogues diverged prior to others, contradicting 2R [12,13,15].

The human HOX gene clusters bearing chromosomes (Hsa 2, 7, 12 and 17) harbor one of the three large quadrupled genomic regions that have been extensively presented in the literature [8,11,14,16,17]. The fact that two or more paralogs of numerous gene families are linked with HOX genes suggests that these paralogous gene sets along with the linked HOX clusters might have arisen by duplications of an intact chromosomal segment, i.e. through block duplication events. This extensive intra-genomic synteny centered on HOX clusters has also been seen as an argument supporting two rounds of whole genome duplication events (2R hypothesis) in the vertebrate lineage [8,16].

In order to track the evolutionary events involved in structuring the mammalian HOX-bearing chromosomes, Hughes and coworkers [14] conducted a phylogenetic analysis of 42 gene families sharing members on two or more of the human chromosomes 2, 7, 12, and 17, the chromosomes that bear HOX clusters. These authors found that phylogenies of 14 HOX linked gene families supported the occurrence of genome duplications before the protostome-deutrostome split. Members of only few families were found to be duplicated within the time window of proposed whole genome/block duplication events. They argued that these genes were actually not duplicated simultaneously with the HOX clusters because the topologies of their phylogenetic trees were not consistent with the HOX cluster phylogeny.

However Larhammar and coworkers [16] advise caution in rejecting the block/chromosomal duplication hypothesis and argued that only genes that are anciently linked to HOX clusters and not those that are transported on the HOX-bearing chromosomes as a result of recent rearrangement events should be considered. They recommended the enrichment of sequence information with diverse classes of vertebrates from mammals to fishes to perform more thorough phylogenetic analysis. Larhammar and coworkers concluded that at least 14 gene families on human HOX-bearing chromosomes display phylogenetic histories compatible with duplications concomitant with the HOX clusters.

**Figure 1:**
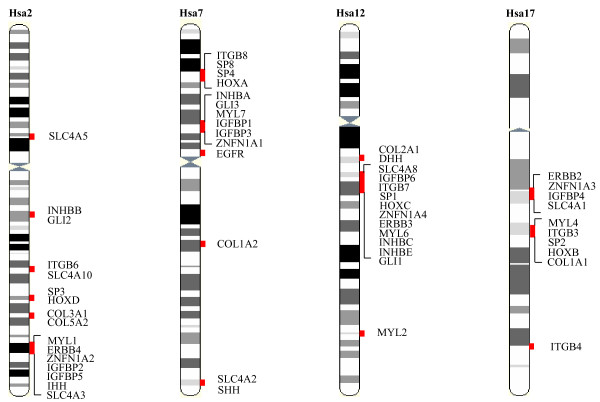
Gene families with members on at least three of the human HOX-bearing chromsomes 2, 7, 12 and 17. Restricted location of members of many of these gene familes near the HOX clusters suggests that these paralogons may have duplicated simultaneously by block/whole chromosome duplication. SLC4, solute carrier family 4; INHB, inhibins; GLI, glioma-associated oncogene homolog belonging to kruppel family; ITGB, integrin β chains; SP, transcription factor Sp; HOX, homeobox; COL, collagens; MYL, myosin light chains; EGFR/ERBB, epidermal growth factor receptor/erythroblastoma; ZNFN1A, zinc finger protein, subfamily 1A; IGFBP, insulin-like growth factor-binding protein; HH, hedgehog. None of the features of this Figure are drawn to scale.

In the present study, we exploit the accessibility of a huge amount of protein data from sequencing and annotation of increasing numbers of vertebrate genomes analyzing the phylogenetic history of 11 HOX linked gene families (Figure 1 and Table 1) to unravel the evolutionary events that brought the HOX clusters and members of these gene families in physical proximity deep in vertebrate history. All of these gene families are anciently linked to HOX clusters with 8 families (Figure 1 and Table 1) having their members on all human HOX-bearing chromosomes, while 3 gene families have paralogs linked to at least three human HOX clusters (Figure 1 and Table 1). It is of note that 9 of these families (Table 1) are among those 14 gene families, which Larhammar and coworkers [16] hypothesized to be duplicated simultaneously with the linked HOX clusters by block duplication event. For each of these 11 HOX linked gene families, the orthologous sequence information from several vertebrate representatives from mammals to bony fishes has been included. Thus, we performed a more robust and thorough phylogenetic analysis compared to previous studies. Given our phylogenetic data, we compared the topologies of those paralogous genes of the each gene family which have arisen within the time window of vertebrates-invertebrates and tetrapods-fishes divergence to test which genes have duplicated concurrently with each other and with the linked HOX clusters at the base of vertebrate lineage.

**Table 1:** Human Gene Families used in Analysis

**Gene family**	**Members**	**Chr Location**	**Human Protein Accession No**	**Molecular Function**
**Fbrillar collagen family**				
	COL2A1	12q13.11-q13.2	P02458	Extracellular matrix structural constituent, Structural constituent of bone, Phosphate transport, Cell adhesion, Skeletal development, Perception of sound.
	COL3A1	2q31	P02461	
	COL5A2	2q14-q32	Q7KZ55	
	COL1A2	7q22.1	P08123	
	COL1A1	17q21.33	P02452	
**ERBB receptor protein-tyrosine kinase**				
	ERBB2	17q21.1	Q96RT1	Epidermal growth factor receptor activity, Protein serine/threonine kinase activity, Electron transporter activity, Cell proliferation, ATP binding.
	EGFR	7p12	P00533	
	ERBB4	2q33.3-q34	Q15303	
	ERBB3	12q13	P21860	
**Insulin-like growth factor-binding protein**				
	IGFBP4	17q12-q21.1	P22692	Regulation of cell growth, Signal transduction, Skeletal development Cell proliferation.
	IGFBP1	7p13-p12	P08833	
	IGFBP2	2q33-q34	P18065	
	IGFBP6	12q13	P24592	
	IGFBP3	7p13-p12	P17936	
	IGFBP5	2q33-q36	P24593	
**Integrin beta chain family**				
	ITGB3	17q21.32	P05106	Receptor activity, Cell-matrix adhesion, Integrin-mediated signaling pathway.
	ITGB5	3q21.2	P18084	
	ITGB6	2q24.2	P18564	
	ITGB7	12q13.13	P26010	
	ITGB4	17q25	P16144	
	ITGB8	7p15.3	P26012	
**Myosin light chain**				
	MYL4	17q21-qter	P12829	Phosphoprotein phosphatase activity, Structural constituent of muscle, Muscle development, Microfilament motor activity.
	MYL6	12q13.2	P60660	
	MYL1	2q33-q34	P06741	
	MYL7	7p21-p11.2	Q01449	
	MYL2	12q23-q24.3	P10916	
**Sp1 c2h2-type zinc-finger protein family**				
	SP1	12q13.1	P08047	RNA polymerase II transcription factor activity.
	SP2	17q21.32	Q02086	
	SP3	2q31	Q02447	
	SP4	7p15	Q02446	
	SP8	7p21.2	Q8IXZ3	
**Zinc finger protein, subfamily 1A**				
	ZNFN1A1	7p13-p11.1	Q13422	DNA-dependent regulation of transcription, Specification and the maturation of the lymphocyte.
	ZNFN1A2	2qter	Q9UKS7	
	ZNFN1A3	17q21	Q9UKT9	
	ZNFN1A4	12q13	Q96JP3	
**Anion exchanger family SLC4A (AE)**				
	SLC4A1	17q21-q22	P02730	Inorganic anion exchanger activity, Bicarbonate transport, Chloride transport.
	SLC4A2	7q35-q36	P04920	
	SLC4A3	2q36	P48751	
	SLC4A5	2p13	Q14203	
	SLC4A8	12q13	O95233	
	SLC4A10	2q23-q24	Q9HCQ6	
**GLI zinc-finger protein family**				
	GLI1	12q13.2-q13.3	P08151	Regulation of transcription from RNA polymerase II promoter, Morphogenesis of limb and brain.
	GLI2	2q14	P10070	
	GLI3	7p13	P10071	
**Hedgehog family**				
	SHH	7q36	Q15465	Mesodermal cell fate determination, Proteolysis and peptidolysis, Cell-cell signaling, Intein-mediated protein splicing.
	DHH	12q12-q13.1	O43323	
	IHH	2q33-q35	Q14623	
**Inhibin**				
	INHBA	7p15-p13	P08476	Cytokine activity, Growth factor activity, Induction of apoptosis, Mesoderm development, Defense response.
	INHBB	2cen-q13	P09529	
	INHBC	12q13.1	P55103	
	INHBE	12q13.3	P58166	

Our results show that gene families with three or more paralogs linked to HOX clusters did not arise simultaneously through two rounds of whole chromosome or whole genome duplication. Instead our analysis shows that these multigene families might have arisen largely as a result of segmental or gene-cluster duplication events, which occurred at different time points during early evolution of vertebrate lineage.

## Results and Discussion

### Phylogenetic Analysis

To perform rigorous testing of the 2R hypothesis, which advocates that four-fold paralogy regions in the human HOX-bearing chromosomes might be remnants of polyploidy, we conducted a phylogenetic analysis of gene families with representatives linked to three or four of the human HOX clusters. Gene families with paralogues linked to only two HOX clusters have been left out, because their occurrence is consistent with several alternative explanatory scenarios.

### Fibrillar Collagen Family – COL

The phylogenetic tree of collagen genes was previously constructed by Bailey and coworkers [18]. Their analysis was based on sequence data from very few species (human, mouse and chicken). In this phylogeny, collagen genes on human chromosomes 7, 12 and 17 formed unresolved trichotomy, while genes on chromosome 2 formed an outgroup.

**Figure 2:**
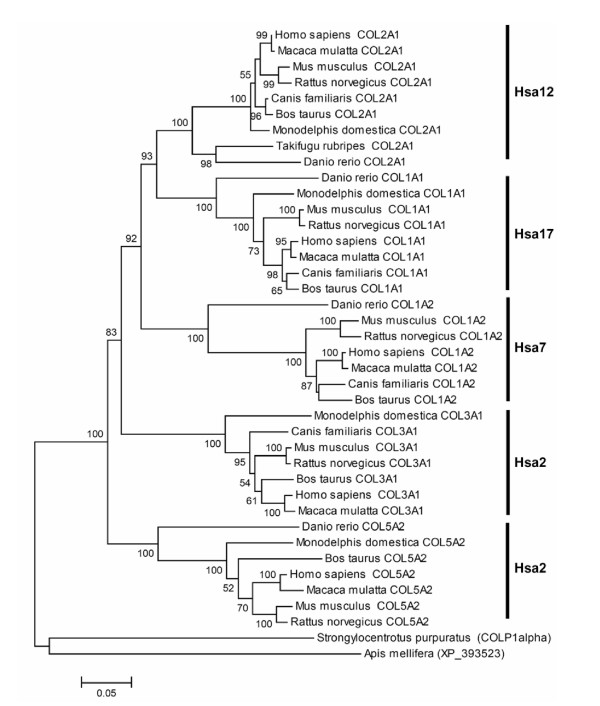
Neighbor-Joining tree of the COL family members. Uncorrected *p*-distance was used. Complete-deletion option was used. Numbers on branches represent bootstrap values (based on 1000 replications) supporting that branch; only the values ≥50% are presented here. Scale bar shows amino acid substitution per site.

Here, we reanalyze the phylogenetic history of collagen genes by including the sequences from representative members of teleost and tetrapod lineages, thus depicting a clearer picture of evolutionary relationship among members of this family (Figure 2). The phylogenetic tree suggests that duplication events giving rise to members of vertebrate collagen gene family occurred prior to the actinopterygii-sarcopterygii and after the echinoderms-chordates split. For the COL3A1 gene the respective time points have not been defined with confidence because orthologous sequences from actinopterygii are unavailable. Phylogeny indicates with bootstrap support of 83% that COL5A2 was the first molecule of this family to diverge. The remaining family members showed the topology of the form (A)(BCD) [13], i.e. (Hsa2)(Hsa12 Hsa17 Hsa7) with COL3A1 falling outside the cluster of COL2A1, COL1A1, and COL1A2 genes. The branch supporting this pattern received the bootstrap support of 92%.

### ERBB Receptor Protein Tyrosine Kinase – ERBB

**Figure 3:**
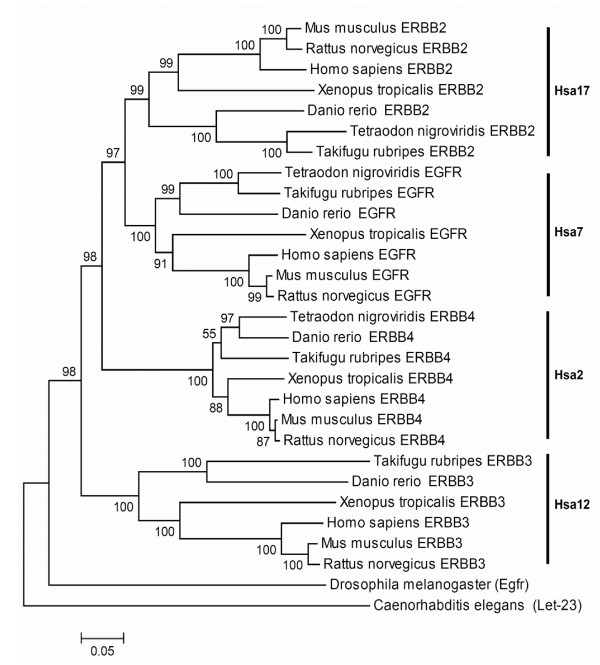
Neighbor-Joining tree of the ERBB family. Symbols and parameters are the same as described in Figure 2.

For the ERBB family a topology of the form (A)(BCD), i.e. (Hsa12)(Hsa17 Hsa7 Hsa2) received a strong bootstrap support (97%) with ERBB3 falling outside the cluster of ERBB2, EGFR and ERBB4 (Figure 3).

The phylogenetic tree showed strong evidence of duplications within the time window of deuterostomes-protostomes and actinopterygii-sarcopterygii split.

### Insuline-like Growth Factor Binding Protein – IGFBP

**Figure 4:**
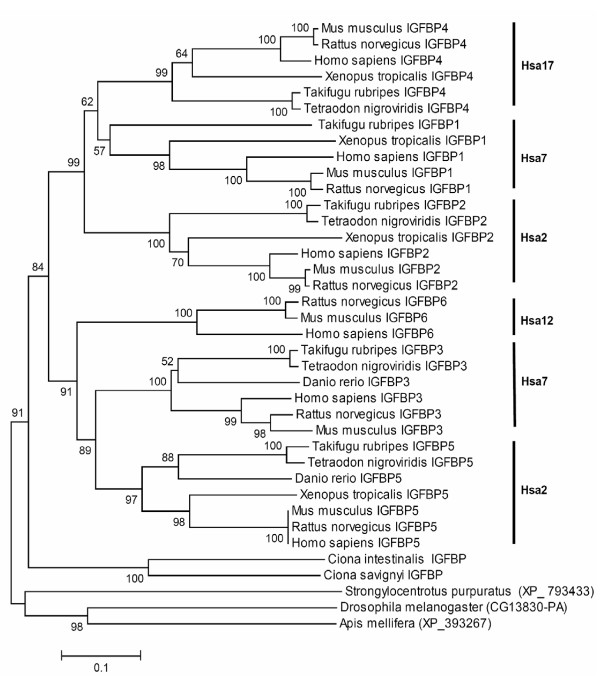
Neighbor-Joining tree of the IGFBP family. Symbols and parameters are the same as described in Figure 2.

The phylogenetic tree of IGFBP family contained two clusters: (I) a cluster in which the IGFBP5-IGFBP3 genes grouped with IGFBP6, (II) a cluster of vertebrate IGFBP4-IGFBP1 genes grouped with IGFBP2 (Figure 4). The bootstrap support for this pattern was significant, i.e. 99% and 91 % for the two relevant branches. The topology of the vertebrate IGFBP family members is unique in a sense that it can be explained by three, rather than two rounds of gene duplication events early in vertebrate history attributed to (AB)(CD) type gene topology [13]. The most parsimonious explanation for this type of topology is: two rounds of whole genome duplication (2R) followed by two independent gene duplication events or three rounds of whole genome duplication followed by two independent gene loss events. We call this topology an extended form of (AB)(CD) type gene topology in which six genes form two clusters, i.e. (ABC)(DEF).

Phylogeny of vertebrate IGFBP proteins suggests that the gene duplication events giving rise to members of this family have occurred after the urchordates-vertebrates and prior to actinopterygii- sarcopterygii split.

### Integrin *β *– ITGB

**Figure 5:**
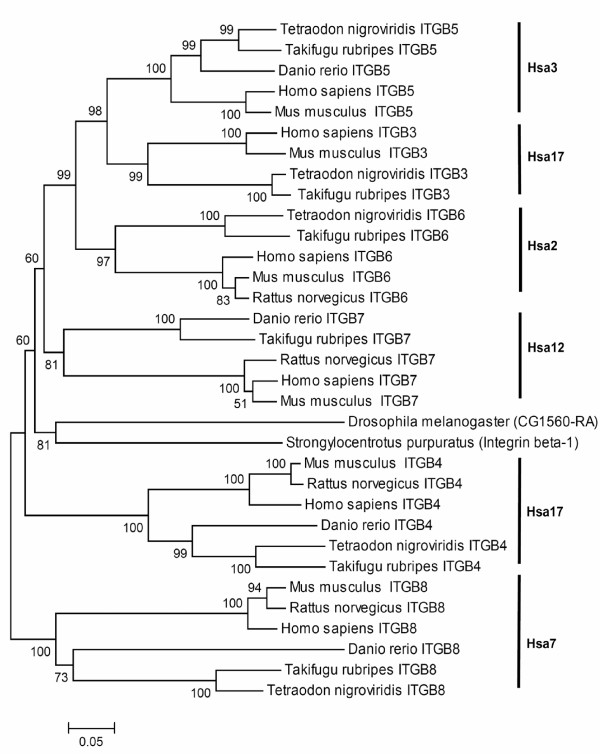
Neighbor-Joining tree of the Integrin *β *chain family. Symbols and parameters are the same as described in Figure 2.

In the phylogenetic tree of the integrin *β *family (Figure 5), vertebrate ITGB5, ITGB3, ITGB6 and ITGB7 genes clustered with homologues from *Drosophila *and sea urchin, indicating that these four members of the integrin *β *family diverged at least after the divergence of echinoderms and chordates. ITGB4 and ITGB8 genes fell outside the ITGB3-5-6-7 cluster and homologues from *Drosophila*, and sea urchin. This topology suggests that gene duplication events giving rise to the ancestor of the ITGB3-5-6-7 cluster may have occurred prior to the divergence of deuterostomes and protostomes.

### Myosin Light Chain – MYL

**Figure 6:**
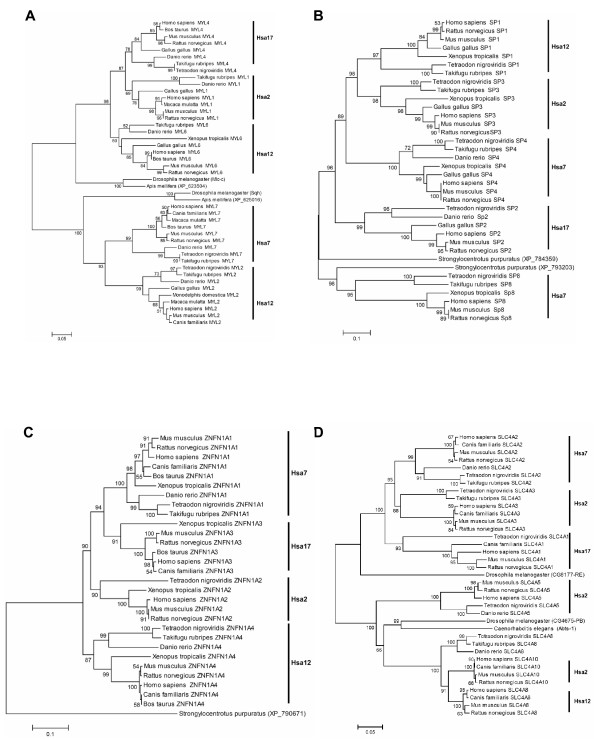
Neighbor-Joining tree of the (A) Myosin light chain family (B) SP family (C) ZNFN1A family (D) SLC4A family. Symbols and parameters are the same as described in Figure 2.

The myosin light chain family members formed two major clusters: (I) cluster including vertebrate MYL1, MYL4 and MYL6 genes and homologues from *Drosophila *and *Apis mellifera*, (II) a cluster including vertebrate MYL2, MYL7 and homologues from *Drosophila *and *Apis mellifera *(Figure 6A). Significant bootstrap support, i.e. 100%, for the internal branch separating the two clusters places the divergence of the ancestors of these two groups prior to the deuterostomes-protostomes split. Subsequent duplications might have occurred early in chordate evolution before the actinopterygii- sarcopterygii divergence.

### Sp1 c2h2-type Zinc-Finger Protein – SP

In the Sp1 c2h2-type zinc-finger protein family (Figure 6B) a significant internal branch (98% bootstrap support) separated: (I) a cluster containing the vertebrate SP1, SP2, SP3, and SP4 genes showing a topology of the form (A)(BCD), i.e. (Hsa17)(Hsa12 Hsa2 Hsa7) that grouped with a homolog from sea urchin with highly significant bootstrap support, i.e. 98%. (II) The vertebrate SP8 molecule clustered independently with a homolog from sea urchin (95% bootstrap support). The phylogeny suggests that the ancestor of vertebrate SP1-4 and SP8 genes duplicated prior to the divergence of chordates and echinoderms.

### Zinc-Finger Protein-Subfamily 1A – ZNFN1A

The vertebrate members of ZNFN1A family showed a topology of the form (A)(BCD), i.e. (Hsa12)(Hsa7 Hsa17 Hsa2), with ZNFN1A4 clustered outside the other three vertebrate genes. The branch supporting this pattern received bootstrap support of 90% (Figure 6C). The topology of the phylogenetic tree indicated that the gene duplications giving rise to ZNFN1A family members occurred within the time window of echinoderms-chordates and actinopterygii-sarcopterygii split.

### Anion Exchanger – SLC4A (AE)

The phylogenetic tree of SLC4A genes (Figure 6D) is divided into two major clusters. Cluster-1 includes vertebrate members SLC4A1, SLC4A2, SLC4A3, and a homolog from *Drosophila*; cluster-2 includes SLC4A5, SLC4A8, SLC4A10, and homologues from *Drosophila *and *C. elegans*. The internal branch separating the two clusters received highly significant (100%) bootstrap support. The topology suggests that gene duplication events giving rise to ancestors of cluster-1 and cluster-2 occurred prior to deuterostomes-protostomes divergence. Phylogeny further indicates that the mammalian SLC4A8 and SLC4A10 genes arose through the duplication of an SLC4A8-like ancestor in the tetrapod lineage at least before the divergence of Euarchontoglires from Laurasiatheria, and the branch supporting this pattern received 91% bootstrap support.

### GLI Zinc-Finger protein – GLI

**Figure 7:**
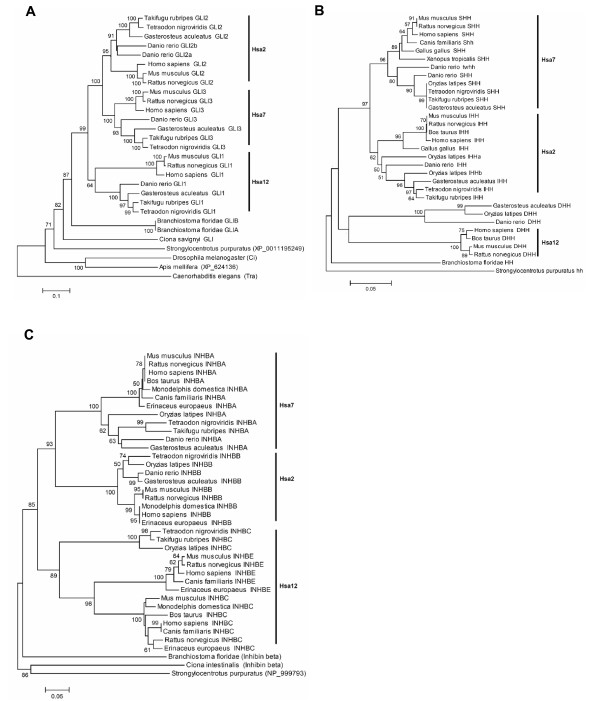
Neighbor-Joining tree of the (A) GLI family (B) Hedgehog family and (C) Inhibin family. Symbols and parameters are the same as described in Figure 2.

The phylogenetic tree indicates that the GLI1, GLI2, and GLI3 genes diverged after the separation of urchordates from vertebrates and before the divergence of tetrapods and bony fishes (Figure 7A). The phylogeny shows a topology of the from (A)(BC), i.e. (Hsa12)(Hsa7 Hsa2) with highly significant (100%) bootstrap support.

### Hedgehog – HH

Vertebrate HH family members showed a topology of the form (A)(BC), i.e. (Hsa12)(Hsa7 Hsa2), with DHH falling outside the SHH-IHH cluster. The branch supporting this pattern received significant (97%) bootstrap support (Figure 7B). Phylogeny attributed the birth of vertebrate HH family members to duplications which occurred within the time window of the cephalochordates-vertebrates and tetrapods- fishes split.

### Inhibin – INHB

The topology of vertebrate inhibin genes (Figure 7C) is similar to HH and GLI families, i.e. (Hsa12)(Hsa7 Hsa2) with 93% bootstrap support. Furthermore, the phylogenetic tree indicates that inhibin paralogs on Hsa12, i.e. INHBC and INHBE originated by a duplication event in tetrapod lineage after its divergence from bony fishes. The branch supporting this pattern received significant (96%) bootstrap support.

### Estimation of Co-duplication Events

**Figure 8:**
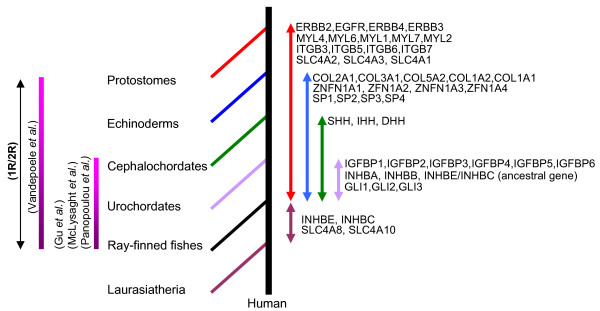
Members of HOX linked gene families that have arisen early in vertebrate history. Order of branching within phylogenetic trees was used to estimate the time windows (double headed arrows on the right) of gene duplication events relative to major cladogenetic events. For each family the lower limit of time window was defined from fish-tetrapod split and the upper limit from the branching order of available closest invertebrate ancestral sequence (Protostomes: *Drosophila*, *Apis mellifera*; Echinoderm: Sea Urchin; Cephalochordates: Amphioxus; Urchordate: *Ciona intestinalis*, *Ciona savignyi*). The INHBE, INHBC and SLC4A8, SLC4A10 genes arose after the fish-tetrapods split. Previously Proposed timing [3-6] of extensive gene duplications during early chordate evolution is given on the left of the diagram.

Given the phylogenetic data, we next sought to determine which genes could have duplicated simultaneously. To test this, we adopted the topology comparison approach [14] and selected the genes from those portions of each phylogeny, where there was a strong statistical support for duplication events within the time window of vertebrates-invertebrates and tetrapods-fishes split (Figure 8, Table 2). Furthermore we included the published phylogeny of vertebrate HOX clusters [19] in this test.

**Table 2:** Summary of the Phylogenetic Analysis of Gene Families

**Family Name**	**Hsa2**	Hsa7/3*	**Hsa12**	**Hsa17**	**Consistency with *HOX *Phylogeny**	**Topology**
ERBB	ERBB4	EGFR	ERBB3	ERBB2	-	(((17, 7)2)12) 97,98
Collagen	COL3A1 COL5A2	COL1A2	COL2A1	COL1A1	-	((((12,17)7)2)2) 93,92,83
IGFBP	IGFBP2 IGFBP5	IGFBP1 IGFBP3	IGFBP6	IGFBP4	-	((17, 7)2) ((7, 2)12)99,91
INTB	ITGB6	ITGB5*	ITGB7	ITGB3	-	(((3, 17)2)12) 98,99
MYL	MYL1	-	MYL6	MYL4	-	((17, 2)12) 87
SP	Sp3	Sp4	Sp1	Sp2	Yes	(((12,2)7)17) 98,89
ZNFN1A	ZNFN1A2	ZNFN1A1	ZNFN1A4	ZNFN1A3	-	(((7,17)2)12) 94,90
INHB	INHBB	INHBA	INHBC INHBE	-	-	((7,2)12) 93
SLC4A	SLC4A3	SLC4A2	-	SLC4A1	-	((7,2)17) 85
GLI	GLI2	GLI3	GLI1	-	-	((7,2)12) 99
HH	IHH	SHH	DHH		-	((7,2)12) 97

For each gene family the chromosomal location and topologies (in the Newick format) of those genes are given, which arose through duplications after the invertebrates-vertebrates split and before the tetrapods-fishes divergence. The percentage bootstrap support of the internal branches is given below each relevant topology.

The topology of the type where genes on chromosomes 7 and 2 clustered together and the gene on chromosome 12 formed an outgroup (Table 2) depicts the simultaneous duplication of members of five gene families, i.e. GLI, HH, INHB, IGFBP (cluster-1), and SLC4A. The third member of the SLC4A family, i.e. SLC4A1, that forms an outgroup to the SLC4A2-SLC4A3 cluster, is on a different chromosome (Hsa17), suggesting that an independent translocation event followed the co-duplication.

The topology of the type where genes on Hsa7 and Hsa17 clustered together, while the gene on Hsa2 branched next, and the gene on Hsa12 formed an outgroup (Table 2), is suggestive of another gene-cluster duplication event involving the members of ERBB, ZNFN1A, and IGFBP (cluster-2) gene families. In addition, the genes showing the topology of the type (Hsa12) (Hsa7 Hsa17 Hsa2) maintained exactly the same order on the respective chromosomal segments, with ZNFN1A genes flanked by ERBB and IGFBP family members (Figure 1). This reflects a conservation of gene order following co-duplications.

In the previously published phylogeny of vertebrate HOX clusters, HOXC and HOXD are grouped together, while the branching order of HOXA and HOXB is unresolved; two alternative topologies (((HOXC HOXD)HOXA)HOXB) and ((HOXC HOXD)(HOXA HOXB)) are equally probable [19]. Within the phylogeny of the SP family, the branching order of SP1, SP2, SP3, and SP4 genes is congruent with one of the two proposed alternative phylogenies of HOX clusters (Table 2). Consistent with the compatibility in their tree topologies, each of the relevant SP genes is closely linked with the HOX cluster (Figure 1), with human SP1 gene mapping at approximately 526 kb centromeric to HOXC, SP2; at ~614 kb centromeric to HOXB, SP3; at ~2 Mb centromeric to HOXD, and SP4; at ~5 Mb telomeric to HOXA. This implies that HOX linked SP genes share the similar evolutionary history as the HOX clusters and have arisen through the same duplication events that led to the HOX clusters.

The phylogenies of the integrin beta chain and myosine light chain families, where the vertebrate genes on Hsa17 and Hsa2 clustered together and the gene on Hsa12 formed an outgroup (Table 2) revealed a fourth simultaneous duplication event. The fact that ITGB3 on Hsa17 grouped with ITGB5 on Hsa3 suggests that an independent translocation event followed the duplication of their ancestor after its divergence from the ITGB6 gene (Figure 5).

The phylogeny of collagen genes showed a different topology (Table 2) which is inconsistent with their having duplicated concomitantly with members of any other gene family that we included in the current study.

### HOX Linked Paralogous Regions May not Reflect the Outcome of Ancient Block or Whole Chromosome Duplication Events

The occurrence of conserved paralogous regions on human HOX-bearing chromosomes Hsa 2/7/12/17 has been taken as evidence that these chromosomes are related by two rounds of block, or whole chromosome doubling events [8,11,16]. This would imply that constituent genes including HOX clusters on each of the relevant chromosomes are suggestive of the architecture of an ancestral block (before vertebrate origin) where all of these genes were linked in a single copy [20].

**Figure 9:**
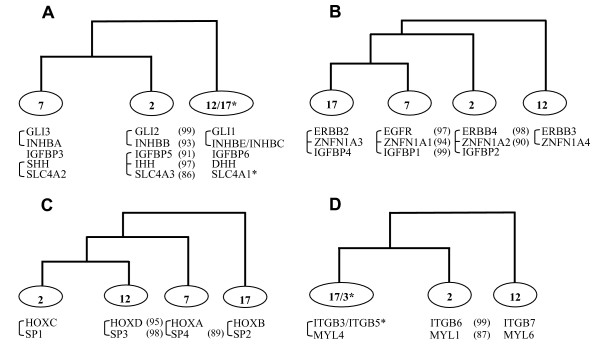
Consistencies in phylogenies of families having members on at least three of the HOX-bearing chromosomes (A) schematic topology of GLI, INHB, IGFBP, HH and SLC4A families (B) schematic topology of ERBB, ZNFN1A and IGFBP family members (C) schematic topology of HOX clusters and SP gene family (D) schematic topology of integrin beta chain and myosin light chain gene families. In each case the percentage bootstrap support of the internal branches is given in parentheses. The connecting bars on the left depict the close physical linkage of relevant genes.

To test whether the four-fold paralogy seen on human HOX-bearing chromosomes (Figure 1) is an outcome of doubling events of a single ancestral block, we employed the topology comparison approach to check the consistencies among the phylogenies of 12 gene families including the HOX clusters. We recovered four independent co-duplicated groups involving the members from total 11 gene families. The largest co-duplicated group suggests the simultaneous duplication of members of five gene families (Figure 9A) where the order and close physical linkage of constituent genes is largely disrupted, except GLI and INHB genes which are tightly bound to each other on each of the relevant chromosomes (Figure 1). The second co-duplicated group involves the members from ERBB, ZNFN1A, and IGFBP families and indicates a conservation of linkage and gene order following co-duplication events (Figure 9B). The HOX clusters and members of the SP gene family represent the third co-duplicated group (Figure 9C); again the constituent genes remained closely linked on each of the relevant chromosomal segments. The fourth co-duplicated group involves the members from two gene families (Figure 9D) where the linkage between the co-duplicated genes is largely disrupted, except on Hsa17 where MYL4 is closely linked to ITGB3 gene (Figure 1).

Our results show that extensive triplicate or quadruplicate synteny that is seen on the present day human HOX-bearing chromosomes is not the outcome of two rounds of duplications experienced by a single ancestral block. Instead, our data suggest that those members of HOX linked gene families that arose within the time window of proposed block duplication events (Figure 8) can be divided into distinct co-duplicated groups. Genes within a particular co-duplicated group share the same evolutionary history and duplicated in concert with each other, while the genes belonging to different co-duplicated groups may not share the evolutionary history and may not have duplicated simultaneously. We conclude that gene families with three or more members on human HOX-bearing chromosomes might be the outcome of gene-cluster duplication events experienced by vertebrates at different time points in their evolutionary history, whereas their current triplicate or quadruplicate distribution on these chromosomes might be the consequence of chromosomal redistribution of multigene family members through extensive rearrangement of genomic segments encompassing multiple contiguous genes. This would imply that although different co-duplicated groups within human chromosomes 2, 7, 12 and 17 are remnants of waves of small-scale duplications (segmental/gene-cluster) and chromosomal rearrangement events, they do not indicate a single ancestral block.

## Conclusion

The four-fold paralogy regions (paralogons) in the human genome, notably on HSA 1/6/9/19, HSA 4/5/8/10, HSA 1/2/8/10 and the HOX-bearing chromosomes HSA 2/7/12/17 are considered to be shaped directly by two rounds polyploidy (quadruplication of single ancestral blocks). Our results show that the constituent gene families of the HOX cluster paralogon have arisen largely by distinct duplication events, and their members were brought together in three or four collinear regions on different chromosomes (Hsa2, 7, 12 and 17) as a result of rearrangements of genomic segments including multiple contiguous genes, at least as early as before the divergence of bony fishes and tetrapods. This data suggests that linkage relationships seen on the human HOX-bearing chromosomes are not an outcome of ancient block or whole chromosome duplications and thus should not be taken as evidence for two rounds of polyploidization events (2R hypothesis). This conclusion may have important implications in resolving the controversies about the evolutionary processes that had shaped our own genome.

## Methods

### Dataset

Genes from 11 families were included in the analysis (Table 1). The chromosomal location of human gene families was obtained from Ensembl genome browser [21], 8 of these families have members on each of the human HOX-bearing chromosomes while 3 have their members on at least three of those chromosomes (Table 1 and Figure 1). Information about the molecular functions (Table 1) of selected gene families was retrieved from GeneReports available at SOURCE [22].

The closest putative orthologous sequences of human proteins in other species were obtained from Orthologue Prediction at Ensembl [21]. To enrich these gene families with sequences from those organisms for which the sequence information was not available at Ensembl, BLASTP [23] search was carried out against the protein database available at National Centre for Biotechnology Information [24] and the Joint Genome Institute [25]. Because the focus of this study was to identify the duplications events which had occurred during vertebrate evolution, only blast hits giving a higher score than the sequence of available invertebrate ancestral sequences were retained. Further confirmation of ancestral-descendents relationship among putative orthologs was done through clustering of homologous proteins within phylogenetic trees. We excluded sequences whose position within a tree was sharply in conflict with the uncontested animal phylogeny. The list of all used sequences is given as Additional file 1.

The species we chose are *Homo sapiens (human)*, *Mus musculus *(mouse), *Rattus norvegicus *(rat), *Gallus gallus *(chicken), *Macaca mulatta *(rhesus monkey), *Canis familiaris *(dog), *Bos taurus *(cow), *Monodelphis domestica *(opossum), *Xenopus tropicalis *(Frog), *Erinaceus europaeus *(hedgehog), *Danio rerio *(zebrafish), *Takifugu rubripes *(Fugu), *Tetraodon nigroviridis*, *Gasterosteus aculeatus *(Stickleback), *Oryzias latipes *(Medaka), *Ciona intestinalis *(ascidian), *Ciona savignyi *(ascidian), *Branchiostoma floridae *(Amphioxus), *Strongylocentrotus purpuratus *(sea urchin), *Drosophila melanogaster *(fruit fly), *Apis mellifera *(honey bee), *Caenorhabditis elegans *(Nematode).

### Alignment and Phylogenetic Analysis

Amino acid sequences were aligned by using CLUSTAL W [26] under default parameters. The alignments were manually refined where necessary. The phylogenetic trees for each gene family were reconstructed by using the neighbor-joining (NJ) method [27], the complete deletion option was used to exclude any site which postulated a gap in the sequences. Poisson corrected (PC) amino acid distance and uncorrected proportion (*p*) of amino acid difference were used as amino acid substitution models. Because both methods produced similar results, only the results from NJ tree based on uncorrected *p*-distance are presented here. Reliability of the resulting tree topology was tested by the bootstrap method [28] (at 1000 pseudoreplicates) which generated the bootstrap probability for each interior branch in the tree.

The phylogenetic trees of seven gene families (COL, ERBB, IGFBP, ZNFN1A, GLI, HH and INHB) were rooted with orthologous genes from invertebrates, whereas the SP phylogeny was rooted with both invertebrate and vertebrate SP8 sequences. The phylogenies of SLC4A and MYL families consisted of two subfamilies, each of which served to root the other. For the ITGB tree, vertebrate ITGB8 sequences served as an outgroup to root the remainder of the tree, while the remaining sequences served to root vertebrate ITGB8 sequences.

For each gene family the order of branching within the phylogenetic tree was used to estimate the time window for gene duplication events relative to the divergence of major taxa of organisms. This method of relative dating does not depend on the assumption of a constant rate of molecular evolution and is thus robust to differences in the rate of evolution in different branches of the tree [12]. Tree topology of each gene family was compared with other families and also with the phylogeny of HOX clusters [19] to test consistencies in duplication events.

## Authors' contributions

KHG and AAA conceived the project and designed the experiments. AAA performed the experiments and analyzed the data. AAA and KHG wrote the paper.

## Supplementary Material

Additional file 1 Complete list of protein sequences used in this study. Click here for file
