# Supplementary material for: An insight into the phylogenetic history of HOX linked gene families in vertebrates
Source: BMC Evol Biol. 2007 Nov 30;7:239. doi: 10.1186/1471-2148-7-239 (PMC2235844; doi:10.1186/1471-2148-7-239)
Supplement: Additional file 1 — Complete list of protein sequences used in this study. [file 1471-2148-7-239-S1.doc]

# Complete list of protein sequences used in this study

**Fibrillar Collagen family**

>Homo sapiens COL1A2

MLSFVDTRTLLLLAVTLCLATCQCKCLQLVWGRLGREGPPGPPGRDGEDGPTGPPGPPGP

PGPPGLGGNFAAQYDGKGVGLGPGPMGLMGPRGPPGAAGAPGPQGFQGPAGEPGEPGQTG

PAGARGPAGPPGKAGEDGHPGKPGRPGERGVVGPQGARGFPGTPGLPGFKGIRGHNGLDG

LKGQPGAPGVKGEPGAPGENGTPGQTGARGLPGERGRVGAPGPAGARGSDGSVGPVGPAG

PIGSAGPPGFPGAPGPKGEIGAVGNAGPAGPAGPRGEVGLPGLSGPVGPPGNPGANGLTG

AKGAAGLPGVAGAPGLPGPRGIPGPVGAAGATGARGLVGEPGPAGSKGESGNKGEPGSAG

PQGPPGPSGEEGKRGPNGEAGSAGPPGPPGLRGSPGSRGLPGADGRAGVMGPPGSRGASG

PAGVRGPNGDAGRPGEPGLMGPRGLPGSPGNIGPAGKEGPVGLPGIDGRPGPIGPAGARG

EPGNIGFPGPKGPTGDPGKNGDKGHAGLAGARGAPGPDGNNGAQGPPGPQGVQGGKGEQG

PPGPPGFQGLPGPSGPAGEVGKPGERGLHGEFGLPGPAGPRGERGPPGESGAAGPTGPIG

SRGPSGPPGPDGNKGEPGVVGAVGTAGPSGPSGLPGERGAAGIPGGKGEKGEPGLRGEIG

NPGRDGARGAPGAVGAPGPAGATGDRGEAGAAGPAGPAGPRGSPGERGEVGPAGPNGFAG

PAGAAGQPGAKGERGAKGPKGENGVVGPTGPVGAAGPAGPNGPPGPAGSRGDGGPPGMTG

FPGAAGRTGPPGPSGISGPPGPPGPAGKEGLRGPRGDQGPVGRTGEVGAVGPPGFAGEKG

PSGEAGTAGPPGTPGPQGLLGAPGILGLPGSRGERGLPGVAGAVGEPGPLGIAGPPGARG

PPGAVGSPGVNGAPGEAGRDGNPGNDGPPGRDGQPGHKGERGYPGNIGPVGAAGAPGPHG

PVGPAGKHGNRGETGPSGPVGPAGAVGPRGPSGPQGIRGDKGEPGEKGPRGLPGLKGHNG

LQGLPGIAGHHGDQGAPGSVGPAGPRGPAGPSGPAGKDGRTGHPGTVGPAGIRGPQGHQG

PAGPPGPPGPPGPPGVSGGGYDFGYDGDFYRADQPRSAPSLRPKDYEVDATLKSLNNQIE

TLLTPEGSRKNPARTCRDLRLSHPEWSSGYYWIDPNQGCTMDAIKVYCDFSTGETCIRAQ

PENIPAKNWYRSSKDKKHVWLGETINAGSQFEYNVEGVTSKEMATQLAFMRLLANYASQN

ITYHCKNSIAYMDEETGNLKKAVILQGSNDVELVAEGNSRFTYTVLVDGCSKKTNEWGKT

IIEYKTNKPSRLPFLDIAPLDIGGADQEFFVDIGPVCFK

>Mus musculus COL1A2

MLSFVDTRTLLLLAVTSCLATCQCNHRCTSVSDRSYISTLLHQQGPAGPRGRDGVDGPMG

PPGPPGSPGPPGSPAPPGLTGNFAAQYSDKGVSSGPGPMGLMGPRGPPGAVGAPGPQGFQ

GPAGEPGEPGQTGPAGPRGPAGSPGKAGEDGHPGKPGRPGERGVVGPQGARGFPGTPGLP

GFKGVKGHSGMDGLKGQPGAQGVKGEPGAPGENGTPGQAGARGLPGERGRVGAPGPAGAR

GSDGSVGPVGPAGPIGSAGPPGFPGAPGPKGELGPVGNPGPAGPAGPRGEVGLPGLSGPV

GPPGNPGTNGLTGAKGATGLPGVAGAPGLPGPRGIPGPAGAAGATGARGLVGEPGPAGSK

GESGNKGEPGSVGAQGPPGPSGEEGKRGSPGEAGSAGPAGPPGLRGSPGSRGLPGADGRA

GVMGPPGNRGSTGPAGIRGPNGDAGRPGEPGLMGPRGLPGSPGNVGPSGKEGPVGLPGID

GRPGPIGPAGPRGEAGNIGFPGPKGPSGDPGKPGERGHPGLAGARGAPGPDGNNGAQGPP

GPQGVQGGKGEQGPAGPPGFQGLPGPSGTTGEVGKPGERGLPGEFGLPGPAGPRGERGTP

GESGAAGPSGPIGSRGPSGAPGPDGNKGEAGAVGAPGSAGASGPGGLPGERGAAGIPGGK

GEKGETGLRGDTGNTGRDGARGIPGAVGAPGPAGASGDRGEAGAAGPSGPAGPRGSPGER

GEVGPAGPNGFAGPAGAAGQPGAKGEKGTKGPKGENGIVGPTGSVGAAGPSGPNGPPGPV

GSRGDGGPPGMTGFPGAAGRTGPPGPSGIAGPPGPPGAAGKEGIRGPRGDQGPVGRTGET

GASGPPGFVGEKGPSGEPGTAGAPGTAGPQGLLGAPGILGLPGSRGERGLPGIAGALGEP

GPLGISGPPGARGPPGAVGSPGVNGAPGEAGRDGNPGSDGPPGRDGQPGHKGERGYPGSI

GPTGAAGAPGPHGSVGPAGKHGNRGEPGPAGSVGPVGAVGPRGPSGPQGIRGDKGEPGDK

GHRGLPGLKGYSGLQGLPGLAGLHGDQGAPGPVGPAGPRGPAGPSGPVGKDGRSGQPGPV

GPAGVRGSQGSQGPAGPPGPPGPPGPPGVSGGGYDFGFEGDFYRADQPRSQPSLRPKDYE

VDATLKSLNNQIETLLTPEGSRKNPARTCRDLRLSHPEWNSDYYWIDPNQGCTMDAIKVY

CDFSTGETCIQAQPVNTPAKNSYSRAQANKHVWLGETINGGSQFEYNVEGVSSKEMATQL

AFMRLLANRASQNITYHCKNSIAYLDEETGSLNKAVLLQGSNDVELVAEGNSRFTYSVLV

DGCSKKTNEWGKTIIEYKTNKPSRLPFLDIAPLDIGGADQEFRVEVGPVCFK

>Rattus norvegicus COL1A2

MLSFVDTRTLLLLAVTSCLATCQCWRGGHPDLNRGKLHPSFHRGASGPRGRDGVDGPVGP

PGPPGAPGPPGPPGPPGLTGNFAAQYSDKGVSAGPGPMGLMGPRGPPGAVGAPGPQGFQG

PAGEPGEPGQTGPAGSRGPAGPPGKAGEDGHPGKPGRPGERGVVGPQGARGFPGTPGLPG

FKGIRGHNGLDGLKGQPGAQGVKGEPGAPGENGTPGQAGARGLPGERGRVGAPGPAGARG

SDGSVGPVGPAGPIGSAGPPGFPGAPGPKGELGPVGNPGPAGPAGPRGEAGLPGLSGPVG

PPGNPGANGLTGAKGATGLPGVAGAPGLPGPRGIPGPVGAAGATGPRGLVGEPGPAGSKG

ETGNKGEPGSAGAQGPPGPSGEEGKRGSPGEPGSAGPAGPPGLRGSPGSRGLPGADGRAG

VMGPPGNRGSTGPAGVRGPNGDAGRPGEPGLMGPRGLPGSPGNVGPAGKEGPVGLPGIDG

RPGPIGPAGPRGEAGNIGFPGPKGPSGDPGKPGEKGHPGLAGARGAPGPDGNNGAQGPPG

PQGVQGGKGEQGPAGPPGFQGLPGPSGTAGEVGKPGERGLPGEFGLPGPAGPRGERGPPG

ESGAAGPSGPIGSRGPSGAPGPDGNKGEAGAVGAPGSAGASGPGGLPGERGAAGIPGGKG

EKGETGLRGEIGNPGRDGARGAPGAIGAPGPAGASGDRGEAGAAGPSGPAGPRGSPGERG

EVGPAGPNGFAGPAGSAGQPGAKGEKGTKGPKGENGIVGPTGPVGAAGPSGPNGPPGPAG

SRGDGGPPGMTGFPGAAGRTGPPGPSGITGPPGPPGAAGKEGIRGPRGDQGPVGRTGEIG

ASGPPGFAGEKGPSGEPGTTGPPGTAGPQGLLGAPGILGLPGSRGERGLPGIAGALGEPG

PLGIAGPPGARGPPGAVGSPGVNGAPGEAGRDGNPGSDGPPGRDGQPGHKGERGYPGNIG

PTGAAGAPGPHGSVGPAGKHGNRGEPGPAGSVGPVGAVGPRGPSGPQGIRGDKGEPGDKG

ARGLPGLKGHNGLQGLPGLAGLHGDQGAPGPVGPAGPRGPAGPSGPIGKDGRSGHPGPVG

PAGVRGSQGSQGPAGPPGPPGPPGPPGVSGGGYDFGFEGDFYRADQPRSQPSLRPKDYEV

DATLKSLNNQIETLLTPEGSRKNPARTCRDLRLSHPEWKSDYYWIDPNQGCTMDAIKVYC

DFSTGETCIQAQPVNTPAKNAYSRAQANKHVWLGETINGGSQFEYNAEGVSSKEMATQLA

FMRLLANRASQNITYHCKNSIAYLDEETGRLNKAVILQGSNDVELVAEGNSRFTYTVLVD

GCSKKTNEWDKTIIEYKTNKPSRLPFLDIAPLDIGGTNQEFRVEVGPVCFK

>Canis familiaris COL1A2

MLSFVDTRTLLLLAVTSCLATCQCKCLRLVWGRRGRKGPPGPPGRDGDDGIPGPPGPPGP

PGPPGLGGNFAAQYDGKGVGLGPGPMGLMGPRGPPGASGAPGPQGFQGPAGEPGEPGQTG

PAGARGPPGPPGKAGEDGHPGKPGRPGERGVVGPQGARGFPGTPGLPGFKGIRGHNGLDG

LKGQPGAPGVKVNTKSEVLSLKQLTDKIGPLQAASIFRSCVVNLLGGNILTTFYFQGPIG

SAGPPGFPGAPGPKGEIGPVGNPGPAGPAGPRGEVGLPGVSGPVGPPGNPGANGLTGAKG

AAGLPGVAGAPGLPGPRGIPGPVGAAGATGARGLVGEPGPAGSKGESGNKGEPGSAGAQG

PPGPSGEEGKRGPNGEAGSAGPSGPPGLRGSPGSRGLPGADGRAGVMGPPGPRGATGPAG

VRGPNGDSGRPGEPGLMGPRGFPGAPGNVGPAGKEGPMGLPGIDGRPGPIGPAGARGEPG

NIGFPGPKGPTGDPGKNGDKGHAGLAGARGAPGPDGNNGAQGPPGPQGVQGGKGEQGPAG

PPGFQGLPGPAGTAGEVGKPGERYNSSNLSLSFSLFSFSQGERGPPGESGAAGPSGPIGS

RGPSGPPGPDGNKGEPGVLGAPGTAGASGPGGLPGERGAAGIPGGKGEKGETGLRGEIGN

PGRDGARGEAGPAGPAGPAGPRGSPGERGEVGPAGPNGFAGPAGAAGQPGAKGERGTKGP

KGENGPVGPTGPIGSAGPSGPNGPPGPAGSRGDGGPPGATGFPGAAGRTGPPGPSGITGP

PGPPGAAGKEGLRGPRGDQGPVGRTGETGASGPPGFTGEKGPSGEPGTAGPPGTPGPQGL

LGAPGILGLPGSRGERGLPGVAGSVGEPGPLGIAGPPGARGPPGAVGAPGVNGAPGEAGR

DGNPGNDGPPGRDGQAGHKGERGYPGNIGPVGAVGAPGPHGPVGPTGKHGNRGEPGPAGS

VGPVGAVGPRGPSGPQGIRGDKGEPGEKGPRGLPGLKGHNGLQGLPGLAGQHGDQGAPGS

VGPAGPRGPAGPSGPAGKDGRTGQPGTVGPAGIRGSQGSQGPAGPPGPPGPPGPPGPSGG

GYDFGYEGDFYRADQPRSPPSLRPKDYEVDATLKSLNNQIETLLTPEGSRKNPARTCRDL

RLSHPEWSSGYYWIDPNQGCTMDAIKVYCDFSTGETCIRAQPENIPAKNWYRNSKVKKHI

WLGETINGGTQFEYNVEGVTTKEMATQLAFMRLLANHASQNITYHCKNSIAYMDEETGNL

KKAVILQGSNDVELVAEGNSRFTYTVLVDGCSKKTNEWRKTIIEYKTNKPSRLPILDIAP

LDIGDADQEFRVDVGPVCFK

>Bos Taurus COL1A2

MLSFVDTRTLLLLAVTSCLATCQCKGSRLSPKVRELFLLTRSEAGPPGPPGRDGDDGIPG

PPGPPGPPGPPGLGGNFAAQFDAKGGGPGPMGIYFIKGRETVEETWGPQGFQGPPGEPGE

PGQTGPAGARGPPGPPGKAGEDGHPGKPGRPGERGVVGPQGARGFPGTPGLPGFKGIRGH

NGLDGLKGQPGAPGVKGEPGAPGENGTPGQTGARGLPGERGRVGAPGPAGARGSDGSVGP

VGPAGPIGSAGPPGFPGAPGPKGELGPVGNPGPAGPAGPRGEVGLPGLSGPVGPPGNPGA

NGLPGAKGAAGLPGVAGAPGLPGPRGIPGPVGAAGATGARGLVGEPGPAGSKGESGNKGE

PGAVGQPGPPGPSGEEGKRGSTGEIGPAGPPGPPGLRGNPGSRGLPGADGRAGVMGPAGS

RGATGPAGVRGPNGDSGRPGEPGLMGPRGFPGSPGNIGPAGKEGPVGLPGIDGRPGPIGP

AGARGEPGNIGFPGPKGPSGDPGKAGEKGHAGLAGARGAPGPDGNNGAQGPPGLQGVQGG

KGEQGPAGPPGFQGLPGPAGTAGEAGKPGERGIPGEFGLPGPAGARGERGPPGESGAAGP

TGPIGSRGPSGPPGPDGNKGEPGVVGAPGTAGPSGPSGLPGERGAAGIPGGKGEKGETGL

RGDIGSPGRDGARGAPGAIGAPGPAGANGDRGEAGPAGPAGPAGPRGSPGERGEVGPAGP

NGFAGPAGAAGQPGAKGERGTKGPKGENGPVGPTGPVGAAGPSGPNGPPGPAGSRGDGGP

PGATGFPGAAGRTGPPGPSGISGPPGPPGPAGKEGLRGPRGDQGPVGRSGETGASGPPGF

VGEKGPSGEPGTAGPPGTPGPQGLLGAPGFLGLPGSRGERGLPGVAGSVGEPGPLGIAGP

PGARGPPGNVGNPGVNGAPGEAGRDGNPGNDGPPGRDGQPGHKGERGYPGNAGPVGAAGA

PGPQGPVGPVGKHGNRGEPGPAGAVGPAGAVGPRGPSGPQGIRGDKGEPGDKGPRGLPGL

KGHNGLQGLPGLAGHHGDQGAPGAVGPAGPRGPAGPSGPAGKDGRIGQPGAVGPAGIRGS

QGSQGPAGPPGPPGPPGPPGPSGGGYEFGFDGDFYRADQPRSPTSLRPKDYEVDATLKSL

NNQIETLLTPEGSRKNPARTCRDLRLSHPEWSSGYYWIDPNQGCTMDAIKVYCDFSTGET

CIRAQPEDIPVKNWYRNSKAKKHVWVGETINGGTQFEYNVEGVTTKEMATQLAFMRLLAN

HASQNITYHCKNSIAYMDEETGNLKKAVILQGSNDVELVAEGNSRFTYTVLVDGCSKKTN

EWQKTIIEYKTNKPSRLPILDIAPLDIGGADQEIRLNIGPVCFK

>Macaca mulatta COL1A2

MLSFVDTRTLLLLAVTSCLATCQCKCLQLVWGRLGREGPPGPPGRDGEDGPTGPPGPPGP

PGPPGLGGNFAAQYDGKGVGLGPGPMGLMGPRGPPGAAGAPGPQGFQGPAGEPGEPGQTG

PAGSRGPAGPPGKAGEDGHPGKPGRPGERGVVGPQGARGFPGTPGLPGFKGIRGHNGLDG

LKGQPGAPGVKGEPGAPGENGTPGQTGARGLPGERGRVGAPGPAGARGSDGSVGPVGPAG

PIGSAGPPGFPGAPGPKGEIGAVGNAGPAGPAGPRGEVGLPGLSGPVGPPGNPGANGLTG

AKGAAGLPGVAGAPGLPGPRGIPGPVGAAGATGARGLVGEPGPAGSKGESGNKGEPGSAG

PQGPPGPSGEEGKRGPNGEVGSAGPPGPPGLRGGPGSRGLPGADGRAGVMGPPGSRGASG

PAGVRGPNGDAGRPGEPGLMGPRGLPGSPGNIGPAGKEGPVGLPGIDGRPGPIGPAGARG

EPGNIGFPGPKGPTGDPGKNGDKGHAGLAGARGAPGPDGNNGAQGPPGPQGVQGGKGEQG

PAGPPGFQGLPGPSGPAGEVGKPGERGLPGEFGLPGPAGARGERGPPGESGAAGPTGPIG

SRGPSGPPGPDGNKGEPGVVGAAGTAGPSGPSGLPGERGAAGIPGGKGEKGEPGLRGEIG

NPGRDGARGAPGAVGAPGPAGATGDRGEAGAAGPAGPAGPRGSPGERGEVGPAGPNGFAG

PAGAAGQPGAKGERGAKGPKGENGVVGPTGPVGAAGPSGPNGPPGPAGSRGDGGPPGMTG

FPGAAGRTGPPGPSGISGPPGPPGPSGKEGLRGPRGDQGPVGRTGEVGAVGPPGFAGEKG

PSGEAGTAGPPGTPGPQGLLGAPGILGLPGSRGERGLPGVAGVVGEPGPLGIAGPPGARG

PPGAVGSPGVNGAPGEAGRDGNPGNDGPPGRDGQPGHKGERGYPGNNGPVGAAGAPGPHG

PVGPAGKHGNRGETGPSGPVGPAGAVGPRGPSGPQGIRGDKGEPGDKGPRGLPGLKGHNG

LQGLPGLAGHHGDQGAPGSVGPAGPRGPAGPSGPAGKDGRTGHPGTVGPAGIRGPQGHQG

PAGPPGPPGPPGPPGVSGGGYDFGYDGDFYRADQPRSAPSLRPKDYEVDATLKSLNNQIE

TLLTPEGSRKNPARTCRDLRLSHPEWSSGYYWIDPNQGCTMDAIKVYCDFSTGETCIRAQ

PENIPAKNWYRSSKDKKHVWLGETINAGSQFEYNVEGVTSKEMATQLAFMRLLANYASQN

ITYHCKNSIAYMDEETGNLKKAVILQGSNDVELVAEGNSRFTYTVLVDGCSKKTNEWGKT

IIEYKTNKPSRLPFLDIAPLDIGGANQEFFVDIGPVCFK

>Danio rerio COL1A2

MLSFVDTRILLLLAVTSYLASCQCGLKGPKGPRGERGPKGPDGKPGRPGLPGPAGPPGPP

GLGGNFAAQYDGAKGPDPGPGPMGLMGPRGPSGSPGAPGAQGLQGHAGEPGEPGQAGAIG

ARGPPGPPGKNGEDGNNGRPGKPGDRGVLGAQGARGFPGTPGLPGMKGHRGYNGIDGRKG

EPGAAGAKGENGAAGSNGTPGQRGGRGLPGERGRVGPAGPAGARGADGNTGPAGPAGPLG

SAGPPGFPGAPGPKGELGPAGPTGPSGAQGQRGEPGPNGAVGPVGPPGNPGANGINGAKG

AAGLPGIAGAPGFPGPRGGPGPQGPSGASGPRGLGGDPGPVGVKGDSGVKGEPGSAGPQG

PPGPSGEEGKRGSTGEQGPTGPLGLRGPRGAAGTRGLPGLAGRSGPMGMPGPRGGVGAPG

ARGPPGDAGRAGEAGLVGARGLPGSPGSSGPPGKEGPSGAAGQDGRTGPPGPTGPRGQPG

NIGFPGPKGPSGEAGKPGEKGPVGPTGLRGSPGPDGNNGPAGPVGLAGAPGEKGEQGPSG

APGFQGLPGPAGPVGEAGKPGDRGIPGDQGVSGPAGVKGERGNPGPAGAAGAQGPIGARG

PSGTPGPDGNKGEPGAVGPAGAPGPQGAAGMPGERGAAGTPGAKGEKGEAGYRGLEGNAG

KDGARGAPGPSGPPGPAGANGDKGETGSFGPPGPAGPRGAPGERGESGPAGPSGFAGPPG

ADGQTGPRGEKGPAGGKGDAGPAGPAGPAGNTGPLGPSGPVGPPGARGDSGPTGLTGFPG

APGRVGPPGPAGIVGPAGLTGPAGKDGPRGPRGDVGPAGPPGENGMIGPLGLAGEKGPPG

EAGAPGAPGPAGPQGQLGSQGFNGLPGSRGDRGLPGIPGSVGEPGRVGPAGAPGARGPGG

NIGMPGMTGPQGEAGREGSPGNDGPPGRPGAAGIKGDRGEPGSPGTAGPVGAPGPNGPSG

AVGRPGNRGESGPSGPTGAVGPAGARGAPGPAGPRGEKGVAGEKGDRGMKGLRGHPGLQG

MPGPNGPSGDSGPAGIAGPSGPRGPAGPNGPAGKDGSNGMPGAIGPPGHRGPAGHVGPAG

PPGSPGLPGPPGPSGGGYDTSGGYDEYRADQASLRAKDYEVDATIKSLNTQIENLLSPEG

SKKNPARTCRDIRLSHPEWSSGFYWIDPNQGCTMDAIKAFCDFSTGQTCIHPHPESIPRK

NWYRSSQEKKHTWFGETINSGTEFAYNDETLSPQSMATQLAFMRLLANQAVQNITYHCKN

SIAYMDAENGNLKKAVLLQGSNDVELRAEGNSRFTFSVLEDGCSRHTGQWSKTVIEYRTN

KPSRLPILDIAPLDIGGADQEFGLDIGPVCFK

>Homo sapiens COL2A1

MIRLGAPQTLVLLTLLVAAVLRCQGQDVRQPGPKGQKGEPGDIKDIVGPKGPPGPQGPAG

EQGPRGDRGDKGEKGAPGPRGRDGEPGTPGNPGPPGPPGPPGPPGLGGNFAAQMAGGFDE

KAGGAQLGVMQGPMGPMGPRGPPGPAGAPGPQGFQGNPGEPGEPGVSGPMGPRGPPGPPG

KPGDDGEAGKPGKAGERGPPGPQGARGFPGTPGLPGVKGHRGYPGLDGAKGEAGAPGVKG

ESGSPGENGSPGPMGPRGLPGERGRTGPAGAAGARGNDGQPGPAGPPGPVGPAGGPGFPG

APGAKGEAGPTGARGPEGAQGPRGEPGTPGSPGPAGASGNPGTDGIPGAKGSAGAPGIAG

APGFPGPRGPPGPQGATGPLGPKGQTGEPGIAGFKGEQGPKGEPGPAGPQGAPGPAGEEG

KRGARGEPGGVGPIGPPGERGAPGNRGFPGQDGLAGPKGAPGERGPSGLAGPKGANGDPG

RPGEPGLPGARGLTGRPGDAGPQGKVGPSGAPGEDGRPGPPGPQGARGQPGVMGFPGPKG

ANGEPGKAGEKGLPGAPGLRGLPGKDGETGAAGPPGPAGPAGERGEQGAPGPSGFQGLPG

PPGPPGEGGKPGDQGVPGEAGAPGLVGPRGERGFPGERGSPGAQGLQGPRGLPGTPGTDG

PKGASGPAGPPGAQGPPGLQGMPGERGAAGIAGPKGDRGDVGEKGPEGAPGKDGGRGLTG

PIGPPGPAGANGEKGEVGPPGPAGSAGARGAPGERGETGPPGPAGFAGPPGADGQPGAKG

EQGEAGQKGDAGAPGPQGPSGAPGPQGPTGVTGPKGARGAQGPPGATGFPGAAGRVGPPG

SNGNPGPPGPPGPSGKDGPKGARGDSGPPGRAGEPGLQGPAGPPGEKGEPGDDGPSGAEG

PPGPQGLAGQRGIVGLPGQRGERGFPGLPGPSGEPGKQGAPGASGDRGPPGPVGPPGLTG

PAGEPGREGSPGADGPPGRDGAAGVKGDRGETGAVGAPGAPGPPGSPGPAGPTGKQGDRG

EAGAQGPMGPSGPAGARGIQGPQGPRGDKGEAGEPGERGLKGHRGFTGLQGLPGPPGPSG

DQGASGPAGPSGPRGPPGPVGPSGKDGANGIPGPIGPPGPRGRSGETGPAGPPGNPGPPG

PPGPPGPGIDMSAFAGLGPREKGPDPLQYMRADQAAGGLRQHDAEVDATLKSLNNQIESI

RSPEGSRKNPARTCRDLKLCHPEWKSGDYWIDPNQGCTLDAMKVFCNMETGETCVYPNPA

NVPKKNWWSSKSKEKKHIWFGETINGGFHFSYGDDNLAPNTANVQMTFLRLLSTEGSQNI

TYHCKNSIAYLDEAAGNLKKALLIQGSNDVEIRAEGNSRFTYTALKDGCTKHTGKWGKTV

IEYRSQKTSRLPIIDIAPMDIGGPEQEFGVDIGPVCFL

>Mus musculus COL2A1

MIRLGAPQSLVLLTLLIAAVLRCQGQDAQEAGSCLQNGQRYKDKDVWKPSSCRICVCDTG

NVLCDDIICEDPDCLNPEIPFGECCPICPADLATASVQIIPTVTRPHPTHLRHIIGPRGP

PGPQGPAGEQGPRGDRGDKGEKGAPGPRGRDGEPGTPGNPGPAGPPGPPGPPGLSAGNFA

AQMAGGYDEKAGGAQMGVMQGPMGPMGPRGPPGPAGAPGPQGFQGNPGEPGEPGVSGPMG

PRGPPGPAGKPGDDGEAGKPGKSGERGLPGPQGARGFPGTPGLPGVKGHRGYPGLDGAKG

EAGAPGVKGESGSPGENGSPGPMGPRGLPGERGRTGPAGAAGARGNDGQPGPAGPPGPVG

PAGGPGFPGAPGAKGEAGPTGARGPEGAQGSRGEPGNPGSPGPAGASGNPGTDGIPGAKG

SAGAPGIAGAPGFPGPRGPPGPQGATGPLGPKGQAGEPGIAGFKGDQGPKGETGPAGPQG

APGPAGEEGKRGARGEPGGAGPIGPPGERGAPGNRGFPGQDGLAGPKGAPGERGPSGLTG

PKGANGDPGRPGEPGLPGARGLTGRPGDAGPQGKVGPSGAPGEDGRPGPPGPQGARGQPG

VMGFPGPKGANGEPGKAGEKGLAGAPGLRGLPGKDGETGAAGPPGPSGPAGERGEQGAPG

PSGFQGLPGPPGPPGEGGKQGDQGIPGEAGAPGLVGPRGERGFPGERGSPGAQGLQGPRG

LPGTPGTDGPKGAAGPDGPPGAQGPPGLQGMPGERGAAGIAGPKGDRGDVGEKGPEGAPG

KDGGRGLTGPIGPPGPAGANGEKGEVGPPGPSGSTGARGAPGERGETGPPGPAGFAGPPG

ADGQPGAKGDQGEAGQKGDAGAPGPQGPSGAPGPQGPTGVTGPKGARGAQGPPGATGFPG

AAGRVGPPGANGNPGPAGPPGPAGKDGPKGVRGDSGPPGRAGDPGLQGPAGAPGEKGEPG

DDGPSGLDGPPGPQGLAGQRGIVGLPGQRGERGFPGLPGPSGEPGKQGAPGASGDRGPPG

PVGPPGLTGPAGEPGREGSPGADGPPGRDGAAGVKGDRGETGALGAPGAPGPPGSPGPAG

PTGKQGDRGEAGAQGPMGPSGPAGARGIAGPQGPRGDKGESGEQGERGLKGHRGFTGLQG

LPGPPGPSGDQGASGPAGPSGPRGPPGPVGPSGKDGSNGIPGPIGPPGPRGRSGETGPVG

PPGSPGPPGPPGPPGPGIDMSAFAGLGQREKGPDPMQYMRADEADSTLRQHDVEVDATLK

SLNNQIESIRSPDGSRKNPARTCQDLKLCHPEWKSGDYWIDPNQGCTLDAMKVFCNMETG

ETCVYPNPATVPRKNWWSSKSKEKKHIWFGETMNGGFHFSYGDGNLAPNTANVQMTFLRL

LSTEGSQNITYHCKNSIAYLDEAAGNLKKALLIQGSNDVEMRAEGNSRFTYTALKDGCTK

HTGKWGKTVIEYRSQKTSRLPIIDIAPMDIGGAEQEFGVDIGPVCFL

>Rattus norvegicus COL2A1

MIRLGAPQSLVLLTLLIATVLQCQGQDAQEAGSCLQNGQRYKDKDVWKPSSCRICVCDTG

NVLCDDIICEDPDCLNPEIPFGECCPICPADLATASGRPRGDRGDKGERGAPGPRGRDGE

PGTPGNPGPPGPPGPPGPPGLGGGNFAAQMAGGFDEKAGGAQMGVMQGPMGPMGPRGPPG

PAGAPGPQGFQGNPGEPGEPGVSGPMGPRGPPGPAGKPGDDGEAGKPGKAGERGLPGPQG

ARGFPGTPGLPGVKGHRGYPGLDGAKGEAGAPGVKGESGSPGENGSPGPMGPRGLPGERG

RTGPAGAAGARGNDGQPGPAGPPGPVGPAGGPGFPGAPGAKGEAGPTGARGPEGAQGSRG

EPGNPGSPGPAGASGNPGTDGIPGAKGSAGAPGIAGAPGFPGPRGPPGPQGATGPLGPKG

QTGEPGIAGFKGEQGPKGETGPAGPQGAPGPAGEEGKRGARGEPGGAGPIGPPGERGAPG

NRGFPGQDGLAGPKGAPGERGPSGLAGPKGANGDPGRPGEPGLPGARGLTGRPGDAGPQG

KVGPSGAPGEDGRPGPPGPQGARGQPGVMGFPGPKGANGEPGKAGEKGLAGAPGLRGLPG

KDGETGAAGPPGPSGPAGERGEQGAPGPSGFQGLPGPPGPPGEGGKQGDQGIPGEAGAPG

LVGPRGERGFPGERGSPGAQGLQGPRGLPGTPGTDGPKGAAGPDGPPGAQGPPGLQGMPG

ERGAAGIAGPKGDRGDVGEKGPEGAPGKDGGRGLTGPIGPPGPAGANGEKGEVGPPGPSG

STGARGAPGERGETGPPGPAGFAGPPGADGQPGAKGDQGEAGQKGDAGAPGPQGPSGAPG

PQGPTGVTGPKGARGAQGPPGATGFPGAAGRVGPPGSNGNPGPAGPPGPAGKDGPKGARG

DTGAPGRAGDPGLQGPAGAPGEKGEPGDDGPSGSDGPPGPQGLAGQRGIVGLPGQRGERG

FPGLPGPSGEPGKQGAPGASGDRGPPGPVGPPGLTGPAGEPGREGSPGADGPPGRDGAAG

VKGDRGETGALGAPGAPGPPGSPGPAGPTGKQGDRGEAGAQGPMGPSGPAGARGIAGPQG

PRGDKGEAGEPGERGLKGHRGFTGLQGLPGPPGPSGDQGTSGPAGPSGPRGPPGPVGPSG

KDGSNGIPGPIGPPGPRGRSGETGPAGPPGNPGPPGPPGPPGPGIDMSAFAGLGQREKGP

DPLQYMRADEADSTLRQHDVEVDATLKSLNNQIESIRSPDGSRKNPARTCQDLKLCHPEW

KSGDYWIDPNQGCTLDAMKVFCNMETGETCVYPNPATVPRKNWWSSKSKEKKHIWFGETM

NGGFHFSYGDGNLAPNTANVQMTFLRLLSTEGSQNITYHCKNSIAYLDEAAGNLKKALLI

QGSNDVEMRAEGNSRFTYTALKDGCTKHTGKWGKTIIEYRSQKTSRLPIVDIAPMDIGGP

DQEFGVDIGPVCFL

>Canis familiaris COL2A1

mirlgapqtlvlltllvaavlrchgqdvqkagscvqdgqryndkdvwkpepcricvcdtg

tvlcddiicedmkdclspetpfgeccpicstdlatasgqpgpkgqkgepgdikdivgpkg

ppgpqgpageqgprgdrgdkgekgapgprgrdgepgtpgnpgppgppgppgppglggnfa

aqmaggfdekaggaqmgvmqgpmgpmgprgppgpagapgpqgfqgnpgepgepgvsgpmg

prgppgppgkpgddgeagkpgksgergppgpqgargfpgtpglpgvkghrgypgldgakg

eagapgvkgesgspgengspgpmgprglpgergrtgpagaagargndgqpgpagppgpvg

paggpgfpgapgakgeagptgargpegaqgprgepgtpgspgpagasgnpgtdgipgakg

sagapgiagapgfpgprgppgpqgatgplgpkgqtgepgiagfkgeqgpkgepgpagpqg

apgpageegkrgargepggagpvgppgergapgnrgfpgqdglagpkgapgergpsglag

pkgangdpgrpgepglpgargltgrpgdagpqgkvgpsgapgedgrpgppgpqgargqpg

vmgfpgpkgangepgkagekglpgapglrglpgkdgetgaagppgpagpagergeqgapg

psgfqglpgppgppgeggkpgdqgvpgeagapglvgprgergfpgergspgaqglqgprg

lpgtpgtdgpkgasgpagppgaqgppglqgmpgergaagiagpkgdrgdvgekgpegapg

kdggrgltgpigppgpagangekgevgppgpagtagargapgergetgppgpagfagppg

adgqpgakgeqgeagqkgdagapgpqgpsgapgpqgptgvtgpkgargaqgppgatgfpg

aagrvgppgsngnpgppgppgpsgkdgpkgargdsgppgragdpglqgpagppgekgepg

ddgpsgpdgppgpqglagqrgivglpgqrgergfpglpgpsgepgkqgapgasgdrgppg

pvgppgltgpsgepgregspgadgppgrdgaagvkgdrgetgpvgapgapgspgspgpag

ptgkqgdrgeagaqgpmgpagpagargipgpqgprgdkgeageagerglkghrgftglqg

lpgppgpsgdqgasgpagpsgprgppgpvgpsgkdgangipgpigppgprgrsgetgpag

ppgnpgppgppgppgpgidmsafaglgqrekgpdplqymradqaagdlrqhdaevdatlk

slnnqiesirspegsrknpartcrdlklchpewksgdywidpnqgctldamkvfcnmetg

etcvypnpasvpkknwwsskskdkkhiwfgetinggfhfsygddnlapntanvqmtflrl

lstegsqnityhcknsiayldeaagnlkkalliqgsndveiraegnsrftytvlkdgctk

htgkwgktmieyrsqktsrlpiidiapmdiggpeqefgvdigpvcfl

>Bos taurus COL2A1

MIRLGAPQTLVLLTLLVAAVLRCHGQDVQKAGSCVQDGQRYNDKDVWKPEPCRICVCDTG

TVLCDDIICEDMKDCLSPETPFGECCPICSADLPTASGRKAPQQLKGKATSRLQIVGPKG

PPGPQGPAGEQGPRGDRGDKGEKGAPGPRGRDGEPGTPGNPGPPGPPGPPGPPGLGGNFA

AQMAGGFDEKAGGAQMGVMQGPMGPMGPRGPPGPAGAPGPQGFQGNPGEPGEPGVSGPMG

PRGPPGPPGKPGDDGEAGKPGKSGERGPPGPQGARGFPGTPGLPGVKGHRGYPGLDGAKG

EAGAPGVKGESGSPGENGSPGPMGPRGLPGERGRTGPAGAAGARGNDGQPGPAGPPGPVG

PAGGPGFPGAPGAKGEAGPTGARGPEGAQGPRGEPGTPGSPGPAGAAGNPGTDGIPGAKG

SAGAPGIAGAPGFPGPRGPPGPQGATGPLGPKGQTGEPGIAGFKGEQGPKGEPGPAGPQG

APGPAGEEGKRGARGEPGGAGPAGPPGERGAPGNRGFPGQDGLAGPKGAPGERGPSGLAG

PKGXXXXXXXXPSPSLSTKGLTGRPGDAGPQGKVGPSGAPGEDGRPGPPGPQGARGQPGV

MGFPGPKGANGEPGKAGEKGLPGAPGLRGLPGKDGETGAAGPPGPAGPAGERGEQGAPGP

SGFQGLPGPPGPPGEGGKPGDQGVPGEAGAPGLVGPRGERGFPGERGSPGSQGLQGARGL

PGTPGTDGPKGAAGPAGPPGAQGPPGLQGMPGERGAAGIAGPKGDRGDVGEKGPEGAPGK

DGGRGLTGPIGPPGPAGANGEKGEVGPPGPAGTAGARGAPGERGETGPPGPAGFAGPPGA

DGQPGAKGEQGEAGQKGDAGAPGPQGPSGAPGPQGPTGVTGPKGARGAQGPPGATGFPGA

AGRVGPPGSNGNPGPPGPPGPSGKDGPKGARGDSGPPGRAGDPGLQGPAGPPGEKGEPGD

DGPSGPDGPPGPQGLAGQRGIVGLPGQRGERGFPGLPGPSGEPGKQGAPGASGDRGPPGP

VGPPGLTGPAGEPGREGSPGADGPPGRDGAAGVKGDRGETGAVGAPGAPGPPGSPGPAGP

IGKQGDRGEAGAQGPMGPAGPAGARGMPGPQGPRGDKGETGEAGERGLKGHRGFTGLQGL

PGPPGPSGDQGASGPAGPSGPRGPPGPVGPSGKDGANGIPGPIGPPGPRGRSGETGPAGP

PGNPGPPGPPGPPGPGIDMSAFAGLGQREKGPDPLQYMRADEAAGNLRQHDAEVDATLKS

LNNQIESLRSPEGSRKNPARTCRDLKLCHPEWKSGDYWIDPNQGCTLDAMKVFCNMETGE

TCVYPNPASVPKKNWWSSKSKDKKHIWFGETINGGFHFSYGDDNLAPNTANVQMTFLRLL

STEGSQNITYHCKNSIAYLDEAAGNLKKALLIQGSNDVEIRAEGNSRFTYTVLKDGCTKH

TGKWGKTMIEYRSQKTSRLPIIDIAPMDIGGPEQEFGVDIGPVCFL

>Macaca mulatta COL2A1

MIRLGAPQTLVLLTLLVAAVLRCQGQDVQEAGSCVQDGQRYNDKDVWKPEPCRICVCDTG

TVLCDDIICEDVKDCLSPEIPFGECCPICPTDLATASGYAGHLCKAEGLKKATSHLQIVG

PKGPPGPQGPAGEQGPRGDRGDKGEKGAPGPRGRDGEPGTPGNPGPPGPPGPPGPPGLGG

NFAAQMAGGFDEKAGGAQMGVMQGPMGPMGPRGPPGPAGAPGPQGFQGNPGEPGEPGVSG

PMGPRGPPGPPGKPGDDGEAGKPGKAGERGPPGPQGARGFPGTPGLPGVKGHRGYPGLDG

AKGEAGAPGVKGESGSPGENGSPGPMGPRGLPGERGRTGPAGAAGARGNDGQPGPAGPPG

PVGPAGGPGFPGAPGAKGEAGPTGARGPEGAQGPRGEPGTPGSPGPAGASGNPGTDGIPG

AKGSAGAPGIAGAPGFPGPRGPPGPQGATGPLGPKGQTGEPGIAGFKGEQGPKGEPGPAG

PQGAPGPAGEEGKRGARGEPGGVGPIGPPGERGAPGNRGFPGQDGLAGPKGAPGERGPSG

LAGPKGANGDPGRPGEPGLPGARGLTGRPGDAGPQGKVGPSGAPGEDGRPGPPGPQGARG

QPGVMGFPGPKGANGEPGKAGEKGLPGAPGLRGLPGKDGETGAAGPPGPAGPAGERGEQG

APGPSGFQGLPGXXXXXXXXXXXXXXGVPGEAGAPGLVGPRGERGFPGERGSPGSQGLQG

ARGLPGTPGTDGPKGASGPAGPPGAQGPPGLQGMPGERGAAGIAGPKGDRGDVGEKGPEG

APGKDGGRGLTGPIGPPGPAGANGEKGEVGPPGPAGSAGARGAPGERGETGPPGPAGFAG

PPGADGQPGAKGEQGEAGQKGDAGAPGPQGPSGAPGPQGPTGVTGPKGARGAQGPPGATG

FPGAAGRVGPPGSNGNPGPPGPPGPSGKDGPKGARGDSGPPGRAGDPGLQGPAGPPGEKG

EPGDDGPSGADGPPGPQGLAGQRGIVGLPGQRGERGFPGLPGPSGEPGKQGAPGASGDRG

PPGPVGPPGLTGPAGEPGREGSPGADGPPGRDGAAGVKGDRGETGAVGAPGSPGPPGSPG

PAGPXXXXXXXXXXXXXXXMGPSGPAGARGIQGPQGPRGDKGEAGEPGERGLKGHRGFTG

LQGLPGPPGPSGDQGASGPAGPSGPRGPPGPVGPSGKDGANGIPGPIGPPGPRGRSGETG

PAGPPGNPGPPGPPGPPGPGIDMSAFAGLGPREKGPDPLQYMRADQAAGGLRQHDAEVDA

TLKSLNNQIESIRSPEGSRKNPARTCRDLKLCHPEWKSGDYWIDPNQGCTLDAMKVFCNM

ETGETCVYPNPANVPKKNWWSSKSKEKKHIWFGETINGGFHFSYGDDNLAPNTANVQMTF

LRLLSTEGSQNITYHCKNSIAYLDEAAGNLKKALLIQGSNDVEIRAEGNSRFTYTALKDD

CTKHTGKWGKTVIEYRSQKTSRLPIIDIAPMDIGGPEQEFGVDIGPVCFL

>Monodelphis domestica COL2A1

QGPAGPQGPPGPAGEEGKRGARGEPGAAGPVGPPGERGAPGNRGFPGQDGLAGPKGAPGE

RGPSGLGGPKGANGDPGRPGEPGLPGARGLTGRPGDAGPQGKVGPSGAPGEDGRPGPPGP

QGARGQPGVMGFPGPKGANGEAGKAGEKGLPGAPGLRGLPGKDGETGAAGPPGPAGPVGE

RGEQGAPGPSGFQGLPGPPGSPGEGGKPGDQGVPGEAGAPGLVGPRGERGFPGERGSPGA

QGLQGARGLPGTPGTDGPKGATGPAGPNGAQGPPGLQGMPGERGAAGIAGPKGDRGDVGE

KGPEGAPGKDGGRGLTGPIGPPGPAGPNGEKGESGPPGPAGTAGARGAPGERGETGPPGP

AGFAGPPGADGQPGAKGEQGPSGQKGDSGSPGPQGPSGAPGPQGPTGVTGPKGTRGAQGP

PGATGFPGAAGRVGPPGSNGNPGPPGPPGASGKDGPKGSRGDTGPPGRAGDPGLQGPAGA

PGEKGEPGDDGPSGPDGPPGPQGLAGQRGIVGLPGQRGERGFPGLPGPSGEPGKQGAPGG

SGDRGPPGPVGPPGLTGPSGEPGREGSPGADGPPGRDGAAGVKGDRGETGPVGGPGAPGP

PGSPGPVGPTGKQGDRGESGAQGPMGPAGPAGARGIAGPQGPRGDKGETGEAGERGLKGH

RGFTGLQGLPGPPGPSGDQGASGPAGPSGPRGPPGPVGPSGKDGSNGIPGPIGPPGPRGR

SGETGPAGPPGNPGPPGPPGPPGPGIDMSAFAGLGQREKGPDPLQYMRADQAAGNLRQHD

AEVDATLKSLNNQIESIRSPEGSRKNPARTCRDLQLCHPEWKSGDYWIDPNQGCTLDAMK

VFCNMETGETCVYPNPNSVPKKNWWSSKGKEKKHVWFGETINGGFHFSYGDDNLAPNTAN

VQMTFLRLLSTEGSQNITYHCKNSIAYLDEAAGNLKKALLIQGSNDVEIRAEGNSRFTYS

VLKDGCTKHTGKWGKTVIEYRSQKTSRLPIIDIAPMDIGGADQEFGVDIGPVCFL

>Takifugu rubripes COL2A1

LSCMQDGQRYSDKDVWKPEPCRICVCDTGTVLCDEIVCEELKDCRNPEIPFGECCPICAA

DQSPPIGRNLFISHFIINKLLHILMLFVLEEGMKVNQRVGPSGEQGPRGTRGEKGERGSA

GPRGRDGEPGTPGNPGSPGPPGPPGLGGVSISLLSWAKYRLTGRIGAQRGPMGPRGPPGP

SGAPGPQGFQGSPGDAGEPGAAGPMGPRGPPGPSGKAGEDGEAGKPGKSGERGPTGPQGA

RGFPGTPGLPGIKGHRGYSGIDGAKGETGAVGSKGESGAPGENGAPGPMGPRGLPGERGR

PGPSGVAGARGNDGLPGPAGPPGPVGPSGAPGFPGSPGSKGEAGPTGARGPEGAQGPRGE

SGTPGSPGPSGASGNPGTDGIPGAKGSAGAHGIAGAPGFPGPRGPPGPQGATGPLGPKGT

SVYPGIAGFKGEAGPKGEIGPAGLQGAPGQQGEEGKRGPRGEPGAAGPIGPPGERGAPGN

GFPGQDGLAGSKGAPGERGTSGASGPKGANGDPGRPGESGLPGARGLTGRPGDAGPQGKV

GPSGAPGEDGRPGPPGPQGARGQPGVMGFPGPKGASGEPGKSGEKGLAGAPGLRGLPGKD

GETGAAGPPGPAGLAGERGEQGQPGPSGFQGLPGPPGPPGEGGKPGDQGVPGEAGASGTT

GPRGERGFPGERGAAGPQGLQGPRGLPGTPGTDGPKGAIGPHGSLGAQGPPGLQGMPGER

GGAGIPGPKGDRGDLGEKGPEGAPGKDGARGLTGPIGPPGPSGPNGEKGETGPAGPSGAP

GTRGTPVCSRATDGSKLTVRALLLQGSDGQPGIKGEQGETGQKGDAGAPGPQGPSGAPGP

AGPTGVSGPKGARGAQGPPGATGFPGAAGRVGPPGPNGNPGPAGPAGSPGKDGPKGIRGD

GGPPGRQGDAGLRGPAGPSGEKGDAGEDGPVGPPGPSGPQGLGGQRGIVGLPGQRGERGF

PGLPGPSGEPGKQGAPGTGGDRGPPGPVGPPGLTGPAGESGREGNPGSDGPPGRDGATGI

KGDRGDTGPTGSPGAPGAPGAQGPVGQTGKQGDRGESGSQGPAGAPGPAGARGMPGPQGP

RGDKGEGGESGERGQKGHRGFTGLQGLPGPPGQAGDQGATGPAGPAGPRGPPGPVGPSGK

DGAFGLPGPIGPPGPRGRSGETGPAGPPGNSGPPGLPGPPGPGIDMSAFAGLGQTEKGPD

PLRYMRADQASGNLRQHDAEVDATLKSLNNQIENIRSPEGSKKNPARTCRDLKLCHPDWK

SGEYWIDPNQGCTVDAIKVYCDMETGETCVQPKPSSIPRKNWWTSKSKDRKHVWFSETMN

GGFHFSYGDDSLAPNTAAIQMTFLRLLSTEASQNLTYHCKNSVAYMDASTGNLKKAVLLQ

GSNDVEIRAEGNSRFTYSVLEDGCTKHTGQWGKTLIEYRSQKTSRLPIVDIAPMDIGEAH

QEFGVEVGAVCFL

>Danio rerio COL2A1

GGCSQDGQLYRDKDVWKPEPCRICVCDSGTVLCDEIVCEELRDCAKPEIPLGECCPVCAS

ADASTPELVCAAQGSEQGDQGSPGPRGRDGEPGTPGNPGPPGPPGPNGPPGLGGNPNPTL

RNAAFPPNTVYMCTESLAGPMGPRGPPGPPGKPGDDLFSSPQLLHELCCSSPLVPHAVSV

MAVGGVSGGEPGKPGNGGERGPPGPQGARGFPGTPGLPGIKGHREDASIVEKTTFTLLPF

AQTSIMITEARNESPKGPRGLPGERGRPGPSGASGARGNDGLPGGAGPPISSSFPGSPGA

KGEAGPTGARGPEGAQGPRGESGVPGASGPSGVSGNPGSDGMPGAKGSVGAPGIGGAPGF

PGPRGPPGPQGATGPLGPKGQSGDSGLAGFKGEAGPKGEIGNAGLQGAPGPAGEEGKRGP

RGEPGAAGPPGPTGERGTPGNRGFPGQDGLAGPKGAPGERGPAGVSGPKGAGGDPGRPGE

PGLLGARALTGRPGDAGPQGKDTGLQGAPGEDGRPGPPGPQGVRGQPGVMGFPGPKGGNG

EAGKAGEKGLAGAPGLRGLPGKDGETGAAGPPGPAGSAGERGEQGQPGPSGFQGLPGPPG

PPGEGGKPGDQQGVPGEAGGAGATGPRGERGFPGERGGAGPQGLQGPRGLPGTPGTDGPK

GGVGPAGTAGAQGPPGLQGMPGERGTSGNPGPKGDRDNIRGFRGPEGAPGKDGSRGLTGP

IGPTGPAGPNGEKGESGPAGPSGVAGTRGVPGDRGETGPPGPAGFAGPPGADGQPGVKGE

QGEGGQKGDAGAPGPQGPSGAPGPQGPTGVSGPKGARGAQGPPGATGFPGAAGRVGPPGP

NGNPGPAGPAGPPGKDGPKGVRGDGGPPGRPGDAGLRGSAGPAGEKGDPGEDGPHGPDGP

AGPQGLAGQRGIVGLPGQRGERGFPGLPGPSGEPGKQGAPGGPGDRGPPGPVGAPGLTGA

AGEPGREGNPGSDGPPGRDGSAGIKGDRGDTGPAGAPGAPGGPGAPGPVGPTGKQGDRGE

AGPHGPSGPPGPAGARGMPGPQGPRGDKGEGGDSGDRGQKGHRGFTGLQGLPGSPGQPGD

QGASGPSGPGGARGPPGPVGPAGKDGANGLPGPIGPPGPRGRSGETGPSGPPGTPGPPGP

PGPPGPGIDMSAFAGLSQLEKSPDPLRYMRADQAADGNHQHDAEVDATLKSLNNQMENIR

RPDGTKKSPARTCRDLKQCHPDWKSGEYWIDPNQGCTVDAIKVFCNMETGESCIYPKPAN

IPRKNWWTTKGGDRKHIWFGEAMNGGFHFNYGDENLSPNTASIQMTFLRLLSTEASQNLT

YHCRNSIAYMDESSGNLKKALLLRGSNDVEIRAEGNSRFTYSVLEDGCKKHTGQWSKTVI

EYKSQKTSRLPLVDIAPVDIGGAHQEFGVDVGAVCFL

>Homo sapiens COL1A1

MFSFVDLRLLLLLAATALLTHGQEEGQVEGQDEDIPPITCVQNGLRYHDRDVWKPEPCRI

CVCDNGKVLCDDVICDETKNCPGAEVPEGECCPVCPDGSESPTDQETTGVEGPKGDTGPR

GPRGPAGPPGRDGIPGQPGLPGPPGPPGPPGPPGLGGNFAPQLSYGYDEKSTGGISVPGP

MGPSGPRGLPGPPGAPGPQGFQGPPGEPGEPGASGPMGPRGPPGPPGKNGDDGEAGKPGR

PGERGPPGPQGARGLPGTAGLPGMKGHRGFSGLDGAKGDAGPAGPKGEPGSPGENGAPGQ

MGPRGLPGERGRPGAPGPAGARGNDGATGAAGPPGPTGPAGPPGFPGAVGAKGEAGPQGP

RGSEGPQGVRGEPGPPGPAGAAGPAGNPGADGQPGAKGANGAPGIAGAPGFPGARGPSGP

QGPGGPPGPKGNSGEPGAPGSKGDTGAKGEPGPVGVQGPPGPAGEEGKRGARGEPGPTGL

PGPPGERGGPGSRGFPGADGVAGPKGPAGERGSPGPAGPKGSPGEAGRPGEAGLPGAKGL

TGSPGSPGPDGKTGPPGPAGQDGRPGPPGPPGARGQAGVMGFPGPKGAAGEPGKAGERGV

PGPPGAVGPAGKDGEAGAQGPPGPAGPAGERGEQGPAGSPGFQGLPGPAGPPGEAGKPGE

QGVPGDLGAPGPSGARGERGFPGERGVQGPPGPAGPRGANGAPGNDGAKGDAGAPGAPGS

QGAPGLQGMPGERGAAGLPGPKGDRGDAGPKGADGSPGKDGVRGLTGPIGPPGPAGAPGD

KGESGPSGPAGPTGARGAPGDRGEPGPPGPAGFAGPPGADGQPGAKGEPGDAGAKGDAGP

PGPAGPAGPPGPIGNVGAPGAKGARGSAGPPGATGFPGAAGRVGPPGPSGNAGPPGPPGP

AGKEGGKGPRGETGPAGRPGEVGPPGPPGPAGEKGSPGADGPAGAPGTPGPQGIAGQRGV

VGLPGQRGERGFPGLPGPSGEPGKQGPSGASGERGPPGPMGPPGLAGPPGESGREGAPGA

EGSPGRDGSPGAKGDRGETGPAGPPGAPGAPGAPGPVGPAGKSGDRGETGPAGPTGPVGP

VGARGPAGPQGPRGDKGETGEQGDRGIKGHRGFSGLQGPPGPPGSPGEQGPSGASGPAGP

RGPPGSAGAPGKDGLNGLPGPIGPPGPRGRTGDAGPVGPPGPPGPPGPPGPPSAGFDFSF

LPQPPQEKAHDGGRYYRADDANVVRDRDLEVDTTLKSLSQQIENIRSPEGSRKNPARTCR

DLKMCHSDWKSGEYWIDPNQGCNLDAIKVFCNMETGETCVYPTQPSVAQKNWYISKNPKD

KRHVWFGESMTDGFQFEYGGQGSDPADVAIQLTFLRLMSTEASQNITYHCKNSVAYMDQQ

TGNLKKALLLQGSNEIEIRAEGNSRFTYSVTVDGCTSHTGAWGKTVIEYKTTKTSRLPII

DVAPLDVGAPDQEFGFDVGPVCFL

>Mus musculus COL1A1

mfsfvdlrlllllgatallthgqedipevscihnglrvpngetwkpevclicichngtav

cddvqcneeldcpnpqrregeccafcpeeyvspnsedvgvegpkgdpgpqgprgpvgppg

rdgipgqpglpgppghpgppgppglggnfasqmsygydeksagvsvpgpmgpsgprglpg

ppgapgpqgfqgppgepgepggsgpmgprgppgppgkngddgeagkpgrpgergppgpqg

arglpgtaglpgmkghrgfsgldgakgdagpagpkgepgspgengapgqmgprglpgerg

rpgppgtagargndgavgaagppgptgptgppgfpgavgakgeagpqgargsegpqgvrg

epgppgpagaagpagnpgadgqpgakgangapgiagapgfpgargpsgpqgpsgppgpkg

nsgepgapgnkgdtgakgepgatgvqgppgpageegkrgargepgpsglpgppgerggpg

srgfpgadgvagpkgpsgergapgpagpkgspgeagrpgeaglpgakgltgspgspgpdg

ktgppgpagqdgrpgpagppgargqagvmgfpgpkgtagepgkagerglpgppgavgpag

kdgeagaqgapgpagpagergeqgpagspgfqglpgpagppgeagkpgeqgvpgdlgapg

psgargergfpgergvqgppgpagprgnngapgndgakgdtgapgapgsqgapglqgmpg

ergaaglpgpkgdrgdagpkgadgspgkdgargltgpigppgpagapgdkgeagpsgppg

ptgargapgdrgeagppgpagfagppgapgapgapgpvgpagkngdrgetgpagpagpig

pagargpagpqgprgdkgetgeqgdrgikghrgfsglqgppgspgspgeqgpsgasgpag

prgppgsagspgkdglnglpgpigppgprgrtgdsgpagppgppgppgppgppsggydfs

flpqppqeksqdggryyraddanvvrdrdlevdttlkslsqqienirspegsrknpartc

rdlkmchsdwksgeywidpnqgcnldaikvycnmetgqtcvfptqpsvpqknwyispnpk

ekkhvwfgesmtdgfpfeygsegsdpadvaiqltflrlmsteasqnityhcknsvaymdq

qtgnlkkalllqgsneielrgegnsrftystlvdgctshtgtwgktvieykttktsrlpi

idvapldigapdqefgldigpacfv

>Rattus norvegicus COL1A1

mfsfvdlrlllllgatallthgqedipevscihnglrvpngetwkpdvclicichngtav

cdgvlckedldcpnpqkregeccpfcpeeyvspdaevigvegpkgdpgpqgprgpvgppg

qdgipgqpglpgppgppgppgplglggnfasqmsygydeksagvsvpgpmgpsgprglpg

ppgapgpqgfqgppgepgepggsgpmgppgppgppgkngddgeagkpgrpgergppgpqg

arglpgtaglpgmkghrgfsgldgakgdtgpagpkgepgspgengtpgqmgprglpgerg

rpgppgtagargndgavgaagppgptgptgppgfpgaagakgeagpqgargsegpqgvrg

epgppgpagaagpagnpgadgqpgakgangapgiagapgfpgargpsgpqgpsgapgpkg

tsgepgapgnkgdtgakgepgpagvqgppgpageegkrgargepgpsglpgppgerggpg

srgfpgadgvagpkgpsgergspgpagpkgspgeagrpgeaglpgakgltgspgspgpdg

ktgppgpagqdgrpgpagppgargqagvmgfpgpkgtagepgkagergvpgppgavgpag

kdgeagaqgapgpagpagergeqgpagspgfqglpgpagppgeagkpgeqgvpgdlgapg

psgargergfpgergvqgppgpagprgnngapgndgakgdtgapgapgsqgapglqgmpg

ergaaglpgpkgdrgdagpkgadgspgkdgvrgltgpigppgpagapgdkgeagpsgpag

ptgargapgdrgeagppgpagfagppgadgqpgakgepgdtgvkgdagppgpagpagppg

pignvgapgpkgsrgaagppgatgfpgaagrvgppgpsgnagppgppgpvgkeggkgprg

etgpagrpgevgppgppgpagekgspgadgpagspgtpgpqgiagqrgvvglpgqrgkrg

fpglpgpsgepgkqgpsgasgergppgpmgppglagppgesgregspgaegspgrdgapg

akgdrgetgpagppgapgapgapgpvgpagkngdrgetgpagpagpigpagargpagpqg

prgdkgetgeqgdrgikghrgfsglqgppgspgspgeqgpsgasgpagprgppgsagspg

kdglnglpgpigppgprgrtgdsgpagppgppgppgppgppsggydfsflpqppqeksqd

ggryyraddanvvrdrdlevdttlkslsqqienirspegsrknpartcrdlkmchsdwks

geywidpnqgcnldaikvycnmetgqtcvfptqpsvpqknwyispnpkekkhvwfgesmt

dgfqfeygsegsdpadvaiqltflrlmsteasqnityhcknsvaymdqqtgnlkkslllq

gsneielrgegnsrftystlvdgctshtgtwgktvieykttktsrlpiidvapldigapd

qefgmdigpacfv

>Canis familiaris COL1A1

MFSFVDLRLLLLLAATALLTHGQEEGQEEDIPPVTCVQNGLRYYDRDVWKPEACRICVCD

NGNVLCDDVICDETKNCPGAQVPPGECCPVCPDGEASPTDQETTGVEGPKGDTGPRGPRG

PAGPPGRDGIPGQPGLPGPPGPPGPPGPPGLGGNFAPQMSYGYDEKSTGGISVPGPMGPS

GPRGLPGPPGAPGPQGFQGPPGEPGEPGASGPMGPRGPPGPPGKNGDDGEAGKPGRPGER

GPPGPQGARGLPGTAGLPGMKGHRGFSGLDGAKGDAGPAGPKGEPGSPGENGAPGQMGPR

GLPGERGRPGAPGPAGARGNDGATGAAGPPGPTGPAGPPGFPGAVGAKGEAGPQGARGSE

GPQGVRGEPGPPGPAGAAGPAGNPGADGQPGAKGANGAPGIAGAPGPGARGPSGPQGPSG

PPGPKGNSGEPGAPGNKGDTGAKGEPGPTGIQGPPGPAGEEGKRGARGEPGPTGLPGPPG

ERGGPGSRGFPGADGVAGPKGPAGERGSPGPAGPKGSPGEAGRPGEAGLPGAKGLTGSPG

SPGPDGKTGPPGPAGQDGRPGPPGPPGARGQAGVMGFPGPKGAAGEPGKAGERGVPGPPG

AVGPAGKDGEAGAQGPPGPAGPAGERGEQGPAGSPGFQGLPGPAGPPGEAGKPGEQGVPG

DLGAPGPSGARGERGFPGERGVQGPPGPAGPRGANGAPGNDGAKGDAGAPGAPGSQGAPG

LQGMPGERGAAGLPGPKGDRGDAGPKGADGSPGKDGVRGLTGPIGPPGPAGAPGDKGEAG

PSGPAGPTGARGAPGDRGEPGPPGPAGFAGPPGADGQPGAKGEPGDAGAKGDAGPPGPAG

PTGPPGPIGNVGAPGPKGARGSAGPPGATGFPGAAGRVGPPGPSGNAGPPGPPGPAGKEG

GKGARGETGPAGRPGEVGPPGPPGPAGEKGSPGADGPAGAPGTPGPQGIAGQRGVVGLPG

QRGERGFPGLPGPSGEPGKQGPSGASGERGPPGPMGPPGLAGPPGESGREGSPGAEGSPG

RDGSPGPKGDRGETGPAGPPGAPGAPGAPGPVGPAGKNGDRGETGPAGPAGPIGPVGARG

PAGPQGPRGDKGETGEQGDRGIKGHRGFSGLQGPPGPPGSPGEQGPSGASGPAGPRGPPG

SAGSPGKDGLNGLPGPIGPPGPRGRTGDAGPVGPPGPPGPPGPPGPPSGGFDFSFLPQPP

QEKAHDGGRYYRADDANVVRDRDLEVDTTLKSLSQQIENIRSPEGSRKNPARTCRDLKMC

HSDWKSGEYWIDPNQGCNLDAIKVFCNMETGETCVYPTQPQVAQKNWYISKNPKEKRHVW

YGESMTDGFQFEYGGQGSDPADVAIQLTFLRLMSTEASQNITYHCKNSVAYMDQQTGNLK

KALLLQGSNEIEIRAEGNSRFTYSVTYDGCTSHTGAWGKTVIEYKTTKTSRLPIIDVAPL

DVGAPDQEFGMDIGPVCFL

>Bos taurus COL1A1

MFSFVDLRLLLLLAATALLTHGQEEGQEEGQEEDIPPVTCVQNGLRYHDRDVWKPVPCQI

CVCDNGNVLCDDVICDELKDCPNAKVPTDECCPVCPEGQESPTDQETTGVEGPKGDTGPR

GPRGPAGPPGRDGIPGQPGLPGPPGPPGPPGPPGLGGNFAPQLSYGYDEKSTGISVPGPM

GPSGPRGLPGPPGAPGPQGFQGPPGEPGEPGASGPMGPRGPPGPPGKNGDDGEAGKPGRP

GERGPPGPQGARGLPGTAGLPGMKGHRGFSGLDGAKGDAGPAGPKGEPGSPGENGAPGQM

VSVSSSGRKGGSRVEQDGARGNDGATGAAGPPGPTGPAGPPGFPGAVGAKGEGGPQGPRG

SEGPQGVRGEPGPPGPAGAAGPAGNPGADGQPGAKGANGAPGIAGAPGFPGARGPSGPQG

PSGPPGPKGNSGEPGAPGSKGDTGAKGEPGPTGIQGPPGPAGEEGKRGARGEPGPAGLPG

PPGERGGPGSRGFPGADGVAGPKGPAGERGAPGPAGPKGSPGEAGRPGEAGLPGAKGLTG

SPGSPGPDGKTGPPGPAGQDGRPGPPGPPGARGQAGVMGFPGPKGAAGEPGKAGERGVPG

PPGAVGPAGKDGEAGAQGPPGPAGPAGERGEQGPAGSPGFQGLPGPAGPPGEAGKPGEQG

VPGDLGAPGPSGARGERGFPGERGVQGPPGPAGPRGANGAPGNDGAKGDAGAPGAPGSQG

APGLQGMPGERGAAGLPGPKGDRGDAGPKGADGAPGKDGVRGLTGPIGPPGPAGAPGDKG

EAGPSGPAGPTGARGAPGDRGEPGPPGPAGFAGPPGADGQPGAKGEPGDAGAKGDAGPPG

PAGPAGPPGPIGNVGAPGPKGARGSAGPPGATGFPGAAGRVGPPGPSGNAGPPGPPGETG

PAGRPGEVGPPGPPGPAGEKGAPGADGPAWESSHPRLPPPQGAPGTPGPQGIAGQRGVVG

LPGQRGERGFPGLPGPSGEPGKQGPSGASGERGPPGPMGPPGLAGPPGESGREGAPGAEG

SPGRDGSPGAKGDRGETGPAGPPGAPGAPGAPGPVGPAGKSGDRGETGPAGPAGPIGPVG

ARGPAGPQGPRGDKGETGEQGDRGIKGHRGFSGLQGPPGPPGSPGEQGPSGASGPAGPRG

PPGSAGSPGKDGLNGLPGPIGPPGPRGRTGDAGPAGPPGPPGPPGPPGPPSGGYDLSFLP

QPPQEKAHDGGRYYRADDANVVRDRDLEVDTTLKSLSQQIENIRSPEGSRKNPARTCRDL

KMCHSDWKSGEYWIDPNQGCNLDAIKVFCNMETGETCVYPTQPSVAQKNWYISKNPKEKR

HVWYGESMTGGFQFEYGGQGSDPADVAIQLTFLRLMSTEASQNITYHCKNSVAYMDQQTG

NLKKALLLQGSNEIEIRAEGNSRFTYSVTYDGCTSHTGAWGKTVIEYKTTKTSRLPIIDV

APLDVGAPDQEFGFDVGPACFL

>Macaca mulatta COL1A1

MFSFVDLRLLLLLAATALLTHGQEEGQVEGQDEDIPPITCVQNGLRYHDRDVWKPEPCRI

CVCDNGKVLCDDVICDETKNCPGAEVPEGECCPVCPDGSESPTDQETTGVEGPKGDTGPR

GPRGPAGPPGRDGIPGQPGLPGPPGPPGPPGPPGLGGNFAPQLSYGYDEKSTGGISVPGP

MGPSGPRGLPGPPGAPGPQGFQGPPGEPGEPGASGPMGPRGPPGPPGKNGDDGEAGKPGR

PGERGPPGPQGARGLPGTAGLPGMKGHRGFSGLDGAKGDAGPAGPKGEPGSPGENGAPGQ

MGPRGLPGERGRPGAPGPAGARGNDGATGAAGPPGPTGPAGPPGFPGAVGAKGEAGPQGP

RGSEGPQGVRGEPGPPGPAGAAGPAGNPGADGQPGAKGANGAPGIAGAPGFPGARGPSGP

QGPGGPPGPKGNSGEPGAPGSKGDTGAKGEPGPVGVQGPPGPAGEEGKRGARGEPGPTGL

PGPPGERGGPGSRGFPGADGVAGPKGPAGERGSPGPAGPKGSPGEAGRPGEAGLPGAKGL

TGSPGSPGPDGKTGPPGPAGQDGRPGPPGPPGARGQAGVMGFPGPKGAAGEPGKAGERGV

PGPPGAVGPAGKDGEAGAQGPPGPAGPAGERGEQGPAGSPGFQGLPGPAGPPGEAGKPGE

QXXXXXXXXXXXXXXXGERGFPGERGVQGPPGPAGPRGANGAPGNDGAKGDAGAPGAPGS

QGAPGLQGMPGERGAAGLPGPKGDRGDAGPKGADGSPGKDGVRGLTGPIGPPGPAGAPGD

KGETGPSGPAGPTGARGAPGDRGEPGPPGPAGFAGPPGADGQPGAKGEPGDAGAKGDAGP

PGPAGPAGPPGPIGNVGAPGPKGARGSAGPPGATGFPGAAGRVGPPGPSGNAGPPGPPGP

AGKEGGKGPRGETGPAGRPGEVGPPGPPGPAGEKGSPGADGPAGAPTPGPQGIAGQRGVV

GLPGQRGERGFPGLPGPSXXXXXXXXXXXXXXXXXXXXXXXXXXXXXXXXXXXXXXXXXX

XXXXXXXXXXXXGDRGETGPAGPPGAPGAPGAPGPVGPAGKSGDRGETGPAGPAGPVGPV

GARGPAGPQGPRGDKGETGEQGDRGIKGHRGFSGLQGPPGPPGSPGEQGPSGASGPAGPR

GPPGSAGTPGKDGLNGLPGPIGPPGPRGRTGDAGPVGPPGPPGPPGPPGPPSGGFDFSFL

PQPPQEKAHDGGRYYRADDANVVRDRDLEVDTTLKSLSQQIENIRSPEGSRKNPARTCRD

LKMCHSDWKSGEYWIDPNQGCNLDAIKVFCNMETGETCVYPTQPSVAQKNWYISKNPKDK

RHVWFGESMTDGFQFEYGGEGSDPADVAIQLTFLRLMSTEASQNITYHCKNSVAYMDQQT

GNLKKALLLQGSNEIEIRAEGNSRFTYSVTVDGCTSHTGAWGKTVIEYKTTKTSRLPIID

VAPLDVGAPDQEFGFDVGPVCFL

>Monodelphis domestica COL1A1

MFSFVDPRLLLLLAVTAVLTHGQDEEDIPEGTCIQNAIKYNNGEVWKPKICQICVCDNGS

ILCDEIICEDVSNCPNVQYKDDECCPSCLGADAVASPSSLTEIGVEGPKGDTGPRGERGP

PGLPGKDGKPGNPGIQGPPGLPGIPGLGGNFASQMSYGYDEKSGGGMSVPGPMGPSGPRG

LPGPPGNPGPQGFQGPPGEPGEPGASGPMGPRGPAGPPGKNGDDGEAGKPGRPGERGPPG

PQGARGLPGTAGLPGMKGHRGFSGLDGAKGDSGPAGPKGEPGSPGENGAPGQMGPRGLPG

ERGRPGPPGPAGARGNDGATGAAGPPGPTGPAGPPGFPGAVGAKGEAGPQGSRGSEGPQG

VRGEPGPPGPAGAAGPSGNPGADGQPGAKGANGAPGIAGAPGFPGARGPSGPQGPSGAPG

PKGNSGEPGAPGNKGDPGAKGEPGPVGVQGPPGPAGEEGKRGSRGEPGPSGLPGPAGERG

GPGSRGFPGADGVAGPKGAPGERGAPGPAGPKGSPGEAGRPGEAGLPGAKGLTGSPGSPG

PDGKTGPPGPAGQDGRPGPPGPPGARGQAGVMGFPGPKGAAGEPGKAGERGVPGPPGAVG

AAGKDGEAGAQGPPGPAGPAGERGEQGPAGSPGFQGLPGPAGPPGEAGKPGEQGVPGDAG

APGPSGARGERGFPGERGVQGPPGPQGPRGSNGAPGNDGAKGDAGAPGAPGGQGPPGLQG

MPGERGAAGLPGAKGDRGDAGPKGADGAAGKDGVRGLTGPIGPPGPAGPTGDKGESGPSG

PVGPTGARGAPGERGEPGPPGPAGFAGPPGADGQPGAKGEPGDAGAKGDAGPPGPAGPTG

APGPAGNVGAPGPKGARGNAGPPGATGFPGAAGRVGPPGPSGNAGPPGPPGPAGKEGGKG

PRGETGPIGRPGEVGPPGPPGPSGEKGSPGADGPAGAPGTPGPQGIAGQRGVVGLPGQRG

ERGFPGLPGPSGEPGKQGPSGISGERGPPGPAGPPGLAGPPGESGREGSPGAEGSPGRDG

SPGPKGDRGETGPAGPPGAPGAPGAPGPVGPAGKSGDRGETGPAGPAGPVGPTGARGPSG

PQGPRGDKGETGEQGDRGMKGHRGFSGLQGPPGPPGSPGEQGPSGASGPAGPRGPPGSAG

ASGKDGLNGLPGPIGPPGPRGRTGDAGPAGPPGPPGPAGPPGPPSGGFDFSFLPQPPQEK

AHDSGRYYRADDANVRDRDLEVDTTLKSLTQQIENIRSPEGTRKNPARTCRDLRMCHSDW

KSGEYWIDPNQGCNLDAIKVFCNMETGETCVYPTQPKVPLKNWYINKNPKDKKHVWFGES

MTDGFQFEYGGEGSDPADVAIQMTFLRLMSTEASQNITYHCKNSVAYMDQQTGNLKKALL

LQGSNEIEIRAEGNSRFTYGVTEDGCTSHTGNWGKTVIEYKTTKTSRLPIIDVAPMDIGA

PNQEFGCDVGPVCFL

>Danio rerio COL1A1

MFSFVDIRLALLLSATVLLARGQGEDDRTGGSCTLDGQVYNDRDVWKPEPCQICVCDSGT

VMCDEVICEDTSDCPNPVIPHDECCPVCPDDDFQEPSVEGPRGSPGDKGERGPAGPPGND

GIPGQPGLPGPPGPPGPPGLGGNFSPQMSGGFDEKSSPMAVPGPMGPMGPRGAPGPPGPS

GPQGFTGPPGEPGEAGAPGPMGPRGAAGPPGKNGEDGESGKPGRPGERGPPGPQGARGFP

GTPGLPGIKGHRGFSGLDGAKGDAGPAGPKGEPGAPGENGTPGAMGPRGLPGERGRAGPP

GAAGARGNDGAAGAAGPPGPTGPAGPPGFPGGPGSKGEVGPQGSRGAEGPQGARGEAGNP

GPAGPAGPAGNNGADGAPGAKGAPGAPGIAGAPGFPGPRGPPGAAGAAGAPGPKGNTGEA

GAPGAKGEAGAKGEAGAQGVQGPPGPPGEEGKRGPRGEPGAGGARGPTGERGAPGARGFP

GADGAAGPRGAPGERGGPGVVGPKGATGEPGRNGEPGMPGSKGMTGSPGSPGPDGKTGPG

GAPGQDGRPGPPGPVGARGQPGVMGFPGPKGAAGEAGKPGERGVMGAIGATGAPGKDGDV

GAPGAPGPAGPAGERGEQGAAGPPGFQGLPGPQGATGEPGKSGEQGAPGEAGAPGPSGSR

GDRGFPGERGAPGPAGPVGARGSPGSAGNDGAKGESGAAGAPGAQGPPGLQGMPGERGAA

GLPGLKGDRGDQGAKGADGAAGKDGIRGMTGPIGPPGPAGAPGDKGESGAQGLVGPTGAR

GPPGERGETGAPGPAGFAGPPGADGLPGAKGEPGDNGAKGDAGAPGPAGATGAPGPQGPV

GATGPKGARGAAGPPGATGFPGAAGRVGPPGPSGNSGPPGPPGPAGKEGQKGNRGETGPA

GRTGEVGAAGPPGAPGEKGNPGAEGATGPAGIPGPQGIGGQRGIVGLPGQRGERGFPGLP

GPSGEIGKQGPSGPSGERGPPGPMGPPGLAGPPGEPGREGTPGNEGSAGRDGAAGPKGDR

GETGPSGTPGAPGPPGAAGPIGPAGKTGDRGETGPAGVPGPAGPSGPRGPSGPAGARGDK

GETGEAGERGMKGHRGFTGMPGPPGPPGPSGESGPAGASGPAGPRGPAGSAGSAGKDGMS

GLPGPIGPPGPRGRNGEIGPAGPPGPPGPPGAPGPSGGGFDIGFIAQPQEKAPDPFRHFR

ADDANVMRDRDLEVDTTLKSLSQQIESIISPDGTKKNPARTCRDLKMCHPDWKSGEYWID

PDQGCNQDAIKVYCNMETGETCVNPTESAIPKKNWYTSKNIKEKKHVWFGEAMTDGFQFE

YGSEGSKPEDVNIQLTFLRLMSTEASQNITYHCKNSIAYMDQASGNLKKALLLQGSNEIE

IRAEGNSRFTYSVTEDGCTSHTGAWGKTVIDYKTTKTSRLPIIDIAPMDVGAPNQEFGIE

VGPVCFL

>Homo sapiens COL3A1

MMSFVQKGSWLLLALLHPTIILAQQEAVEGGCSHLGQSYADRDVWKPEPCQICVCDSGSV

LCDDIICDDQELDCPNPEIPFGECCAVCPQPPTAPTRPPNGQGPQGPKGDPGPPGIPGRN

GDPGIPGQPGSPGSPGPPGICESCPTGPQNYSPQYDSYDVKSGVAVGGLAGYPGPAGPPG

PPGPPGTSGHPGSPGSPGYQGPPGEPGQAGPSGPPGPPGAIGPSGPAGKDGESGRPGRPG

ERGLPGPPGIKGPAGIPGFPGMKGHRGFDGRNGEKGETGAPGLKGENGLPGENGAPGPMG

PRGAPGERGRPGLPGAAGARGNDGARGSDGQPGPPGPPGTAGFPGSPGAKGEVGPAGSPG

SNGAPGQRGEPGPQGHAGAQGPPGPPGINGSPGGKGEMGPAGIPGAPGLMGARGPPGPAG

ANGAPGLRGGAGEPGKNGAKGEPGPRGERGEAGIPGVPGAKGEDGKDGSPGEPGANGLPG

AAGERGAPGFRGPAGPNGIPGEKGPAGERGAPGPAGPRGAAGEPGRDGVPGGPGMRGMPG

SPGGPGSDGKPGPPGSQGESGRPGPPGPSGPRGQPGVMGFPGPKGNDGAPGKNGERGGPG

GPGPQGPPGKNGETGPQGPPGPTGPGGDKGDTGPPGPQGLQGLPGTGGPPGENGKPGEPG

PKGDAGAPGAPGGKGDAGAPGERGPPGLAGAPGLRGGAGPPGPEGGKGAAGPPGPPGAAG

TPGLQGMPGERGGLGSPGPKGDKGEPGGPGADGVPGKDGPRGPTGPIGPPGPAGQPGDKG

EGGAPGLPGIAGPRGSPGERGETGPPGPAGFPGAPGQNGEPGGKGERGAPGEKGEGGPPG

VAGPPGGSGPAGPPGPQGVKGERGSPGGPGAAGFPGARGLPGPPGSNGNPGPPGPSGSPG

KDGPPGPAGNTGAPGSPGVSGPKGDAGQPGEKGSPGAQGPPGAPGPLGIAGITGARGLAG

PPGMPGPRGSPGPQGVKGESGKPGANGLSGERGPPGPQGLPGLAGTAGEPGRDGNPGSDG

LPGRDGSPGGKGDRGENGSPGAPGAPGHPGPPGPVGPAGKSGDRGESGPAGPAGAPGPAG

SRGAPGPQGPRGDKGETGERGAAGIKGHRGFPGNPGAPGSPGPAGQQGAIGSPGPAGPRG

PVGPSGPPGKDGTSGHPGPIGPPGPRGNRGERGSEGSPGHPGQPGPPGPPGAPGPCCGGV

GAAAIAGIGGEKAGGFAPYYGDEPMDFKINTDEIMTSLKSVNGQIESLISPDGSRKNPAR

NCRDLKFCHPELKSGEYWVDPNQGCKLDAIKVFCNMETGETCISANPLNVPRKHWWTDSS

AEKKHVWFGESMDGGFQFSYGNPELPEDVLDVHLAFLRLLSSRASQNITYHCKNSIAYMD

QASGNVKKALKLMGSNEGEFKAEGNSKFTYTVLEDGCTKHTGEWSKTVFEYRTRKAVRLP

IVDIAPYDIGGPDQEFGVDVGPVCFL

>Mus musculus COL3A1

prvrpgikgpagmpgfpgmkghrgfdgrngekgetgapglkgenglpgdngapgpmgprg

apgergrpglpgaagargndgargsdgqpgppgppgtagfpgspgakgevgpagspgsng

spgqrgepgpqghagaqgppgppgnngspggkgemgpagipgapgligargppgpagtng

ipgtrgpsgepgkngakgepgargergeagspgipgpkgedgkdgspgepganglpgaag

ergpsgfrgpagpngipgekgppgerggpgpagprgvagepgrdgtpggpgirgmpgspg

gpgndgkpgppgsqgesgrpgppgpsgprgqpgvmgfpgpkgndgapgkngerggpggpg

lpgpagkngetgpqgppgptgpagdkgdsgppgpqglqgipgtggppgengkpgepgpkg

evgapgapggkgdsgapgergppgtagipgarggagppgpeggkgpagppgppgasgspg

lqgmpgerggpgspgpkgekgepggagadgvpgkdgprgpagpigppgpagqpgdkgegg

spglpgiagprggpgergehgppgpagfpgapgqngepgakgergapgekgeggppgpag

ptgssgpagppgpqgvkgergspggpgtagfpggrglpgppgnngnpgppgpsgapgkdg

ppgpagnsgspgnpgiagpkgdagqpgekgppgaqgppgspgplgiagltgarglagppg

mpgprgspgpqgikgesgkpgasghngergppgpqglpgqpgtagepgrdgnpgsdgqpg

rdgspggkgdrgengspgapgapghpgppgpvgpsgksgdrgetgpagpsgapgpagarg

apgpqgprgdkgetgergsngikghrgfpgnpgppgspgaaghqgaigspgpagprgpvg

phgppgkdgtsghpgpigppgprgnrgergsegspghpgqpgppgppgapgpccgggaaa

iagvggeksggfspyygddpmdfkinteeimsslksvngqieslispdgsrknparncrd

lkfchpelksgeywvdpnqgckmdaikvfcnmetgetcinaspmtvprkhwwtdsgaekk

hvwfgesmnggfqfsygtpdlpedvvdvqlaflrllssrasqnityhcknsiaymdqasg

nvkkslklmgsnegefkaegnskftytvledgctkhtgewsktvfeyqtrkamrlpiidi

apydiggpdqefgvdigpvcfl

>Rattus norvegicus COL3A1

MMSFVQCGTWFLLTLLHPSLILAQQSNVDELGCNYLGQSYESRDVWKPEPCQICVCDSGS

VLCDDIMCDDEPLDCPNPEIPFGECCAICPQPSTPAPVIPDGNRPQGPKGDPGPPGIPGR

NGDPGLPGQPGLPGPPGSPGICESCPTGGQNYSPQFDSYDVKSGVGGMGGYPGPAGPPGP

PGPPGSSGHPGSPGSPGYQGPPGEPGQAGPAGPPGPPGAIGPSGPAGKDGESGRPGRPGE

RGLPGPPGIKGPAGIPGFPGMKGHRGFDGRNGEKGETGAPGLKGENGLPGDNGAPGPMGP

RGAPGERGRPGLPGAAGARGNDGARGSDGQPGPPGPPGTAGFPGSPGAKGEVGPAGSPGS

NGSPGQRGEPGPQGHAGAQGPPGPPGNNGSPGGKGEMGPAGIPGAPGLLGARGPPGPAGA

NGAPGQRGPSGEPGKNGAKGEPGARGERGEAGSPGIPGPKGEDGKDGSPGEPGANGVPGN

PGERGAPGFRGPAGPNGAPGEKGPAGERGGPGPAGPRGVAGEPGRDGTPGGPGIRGMPGS

PGGPGNDGKPGPPGSQGESGRPGPPGPSGPRGQPGVMGFPGPKGNDGAPGKNGERGGPGG

PGLPGPAGKNGETGPQGPPGPTGAPGDKGDAGPPGPQGLQGIPGTSGPPGENGKPGEPGP

KGEAGAPGVPGGKGDSGAPGERGPPGTAGTPGLRGGAGPPGPEGGKGPAGPPGPPGTSGP

PGLQGMPGERGGPGSPGPKGEKGEPGGAGADGVPGKDGPRGPAGPIGPPGPAGQPGDKGE

GGAPGLPGIAGPRGGPGERGEHGPPGPAGFPGAPGQNGEPGAKGERGAPGEKGEGGPPGA

AGPPGGSGPAGPPGPQGVKGERGSPGGPGAAGFPGGRGLPGPPGNNGNPGPPGPSGAPGK

DGPPGPAGNSGSPGNPGVAGPKGDAGQPGEKGPPGAQGPPGSPGPLGIAGLTGARGLAGP

PGMPGPRGSPGPQGIKGESGKPGASGHNGERGPPGPQGLPGQPGTAGEPGRDGNPGSDGQ

PGRDGSPGGKGDRGENGSPGAPGAPGHPGPPGPVGPSGKNGDRGETGPAGPSGAPGPAGA

RGAPGPQGPRGDKGETGERGSNGIKGHRGFPGNPGPPGSPGAAGHQGAVGSPGPAGPRGP

VGPHGPPGKDGSSGHPGPIGPPGPRGNRGERGSEGSPGHPGQPGPPGPPGAPGPCCGGGA

AIAGVGGEKSGGFSPYYGDDPMDFKINTEEIMSSLKSVNGQIESLISPDGSRKNPARNCR

DLKFCHPELKSGEYWVDPNQGCKMDAIKVFCNMETGETCINASPMTVPRKHWWTDAGAEK

KHVWFGESMNGGFQFSYGNPDLPEDVLDVQLAFLRLLSSRASQNITYHCKNSIAYMDQAN

GNVKKSLKLMGSNEGEFKAEGNSKFTYTVLEDGCTKHTGEWSKTVFEYQTRKAMRLPIID

IAPYDIGGPDQEFGVDIGPVCFL

>Canis familiaris COL3A1

MTSFVQKGTWLLLALLQPAVISAQQQAIDGGCSHLGQSYADRDVWKPEPCQICVCDSGSV

LCDDIICDEQELDCPNPEIPFGECCAVCPQPPTSPPRPPNGHGPQGPKGDPGPPGIPGRN

GDPGIPGQPGSPGSPGPPGICESCPTGPQNYSPQFESYDVKAGVAGGGIGGYPGPAGPPG

PPGPPGTSGHPGSPGSPGYQGPPGEPGQAGPAGPPGPPGAMGPSGPAGKDGESGRPGRPG

ERGLPGPPGMKGPAGMPGFPGMKGHRGFDGRNGEKGDTGAPGLKGENGLPGENGAPGPMG

PRGAPGERGRPGLPGAAGARGNDGARGSDGQPGPPGPPGTAGFPGSPGAKGEVGPAGSPG

SNGSPGQRGEPGPQGHAGAPGPPGPPGSNGSPGGKGEMGPAGIPGAPGLIGARGPPGPPG

TNGAPGQRGGAGEPGKNGAKGEPGPRGERGEAGSPGIPGPKGEDGKDGSPGEPGANGLPG

AAGERGAPGFRGPAGANGLPGEKGPAGERGGPGPAGPRGAPGEPGRDGVPGGPGMRGMPG

SPGGPGSDGKPGPPGSQGESGRPGPPGPSGPRGQPGVMGFPGPKGNDGAPGKNGERGGPG

GPGPQGPAGKNGETGPQGPPGPTGPAGDKGDAGPPGPQGLQGLPGTSGPPGENGKPGEPG

PKGESGSPGVPGGKGDSGAPGERGPPGAAGPMGPRGGAGPPGPEGGKGAAGPPGPPGSAG

TPGLQGMPGERGGPGGPGPKGDKGEPGSAGADGAPGKDGPRGPTGPIGPPGPAGQPGDKG

EGGAPGLPGIAGPRGGPGERGEHGPPGPAGFPGAPGQNGEPGAKGERGAPGEKGEGGPPG

VAGPPGGAGPAGPPGPQGVKGERGSPGGPGAAGFPGGRGLPGPPGNNGNPGPPGSSGAPG

KDGPPGPPGNNGAPGSPGVSGPKGDAGQPGEKGSPGPQGPPGAPGPLGIAGITGARGLAG

PPGIPGPRGSPGPQGVKGENGKPGPSGHNGERGPPGPQGLPGLAGTAGEPGRDGNPGSDG

LPGRDGAPGGKGDRGENGSPGAPGAPGHPGPPGPVGPAGKSGDRGETGPAGPSGAPGPAG

SRGPPGPQGPRGDKGETGERGLNGIKGHRGFPGNPGAPGSPGPAGHQGAIGSPGPAGPRG

PVGPSGPPGKDGTSGHPGPIGPPGPRGNRGERGSEGSPGHPGQPGPPGPPGAPGPCCGGG

AAALAAAGGEKAGGFAPYYGDEPMDFKINTEEIMTSLKSVSGQIESLVSPDGSRKHPARN

CRDLKFCHPELKSGEYWVDPNQGCKLDAIKVFCNMETGETCLNASPGSVPRKNWWTDSGA

EKKHVWFGESMDGGFQFGYGNPELPEDVLDVQLAFLRLLSSRASQNITYHCKNSIAYMDH

ASGNVKKALRLMGSNEGEFKAEGNSKFTYTVLEDGCTKHTGEWGKTVFEYRTRKAVRLPI

IDIAPYDVGGPDQEFGVDVGPVCFL

>Bos taurus COL3A1

HLDMMSFVQKGTWLLFALLHPTVILAQQEAVDGGCSHLGQSYADRDVWKPEPCQICVCDS

GSVLCDDIICDDQELDCPNPEIPFGECCAVCPQPPTAPTRPPNGQGPQGPKGDPGPPGIP

GRNGDPGPPGSPGSPGSPGPPGICESCPTGGQNYSPQYEAYDVKSGVAGGGIAGYPGPAG

PPGPPGPPGTSGHPGAPNIPNYNNKLIITEKSTDGPPGPPGAIGPSGPAGKDGESGRPGR

PGERGFPGPPGMKGPAGMPGFPGMKGHRGFDGRNGEKGETGAPGLKGENGVPGENGAPGP

MGPRGAPGERGRPGLPGAAGARGNDGARGSDGQPGPPGPPGTAGFPGSPGAKGEVGPAGS

PGSSGAPGQRGEPGPQGHAGAPGPPGPPGSNGSPGGKGEMGPAGIPGAPGLIGARGPPGP

PGTNGVPGQRGAAGEPGKNGAKGDPGPRGERGEAGSPGIAGPKGEDGKDGSPGEPGANGL

PGAAGERGVPGFRGPAGANGLPGEKGPPGDRGGPGPAGPRGVAGEPGRDGLPGGPGLRGI

PGSPGGPGSDGKPGPPGSQGETGRPGPPGSPGPRGQPGVMGFPGPKGNDGAPGKNGERGG

PGGPGPQGPAGKNGETGPQGPPGPTGPSGDKGDTGPPGPQGLQGLPGTSGPPGENGKPGE

PGPKGEAGAPGIPGGKGDSGAPGERGPPGAGGPPGPRGGAGPPGPEGGKGAAGPPGPPGS

AGTPGLQGMPGERGGPGGPGPKGDKLKEGNITGKDASNGPTGPIGPPGPAGQPGDKGESG

APGVPGIAGPRGGPGERGEQGPPGPAGFPGAPGQNGEPGAKGERGAPGEKGEGGPPGAAG

PAGGSGPAGPPGPQGVKGERGSPGGPGAAGFPGGRGPPGPPGSNGNPGPPGSSGAPGKDG

PPGPPGSNGAPGSPGISGPKGDSGPPGERGAPGPQGPPGAPGPLGIAGLTGARGLAGPPG

MPGARGSPGPQGIKGENGKPGPSGQNGERGPPGPQGLPGLAGTAGEPGRDGNPGSDGLPG

RDGAPGAKGDRGENGSPGAPGAPGHPGPPGPVGPAGKSGDRGETGPAGPSGAPGPAGSRG

PPGPQGPRGDKGETGERGAMGIKGHRGFPGNPGAPGSPGPAGHQGAVGSPGPAGPRGPVG

PSGPPGKDGASGHPGPIGPPGPRGNRGERGSEGSPGHPGQPGPPGPPGAPGPCCGAGGVA

AIAGVGAEKAGGFAPYYGDEPIDFKINTDEIMTSLKSVNGQIESLISPDGSRKNPARNCR

DLKFCHPELQSGEYWVDPNQGCKLDAIKVYCNMETGETCISASPLTIPQKNWWTDSGAEK

KHVWFGESMEGGFQFSYGNPELPEDVLDVQLAFLRLLSSRASQNITYHCKNSIAYMDHAS

GNVKKALKLMGSNEGEFKAEGNSKFTYTVLEDGCTKHTGEWGKTVFQYQTRKAVRLPIVD

IAPYDIGGPDQEFGADIGPVCFL

>Macaca mulatta COL3A1

MMSFVQKGSWLLLALLHPTIILAQQEAVEGGCSHLGQSYADRDVWKPEPCQICVCDSGSV

LCDDIICDDQELDCPNPEIPFGECCAVCPQPPTAPTRPPNGQGPQGPKGDPGPPGIPGRN

GDPGIPGQPGSPGSPGPPGICESCPTGPQNYSPQYDSYDVKSGVAVGGLGGYPGPAGPPG

PPGPPGTSGHPGSPGSPGYQGPPGEPGQAGPAGPPGPPGAMGPSGPAGKDGESGRPGRPG

ERGLPGPPGIKGPAGIPGFPGMKGHRGFDGRNGEKGETGAPGLKGENGLPGENGAPGPMG

PRGAPGERGRPGLPGAAGARGNDGARGSDGQPGPPGPPGTAGFPGSPGAKGEVGPAGSPG

SNGAPGQRGEPGLQGQAGAQGPPGPPGSSGSPGGKGEMGPAGIPGAPGLMGARGPPGPPG

ANGAPGLRGGAGEPGKNGAKGEPGPRGERGEAGIPGVPGAKGEDGKDGSPGEPGANGLPG

AAGERGAPGFRGPAGPNGIPGEKGPAGDRGAPGPAGPRGAAGEPGRDGVPGGPGMRGMPG

SPGGPGSDGKPGPPGSQGESGRPGPPGPSGPRGQPGVMGFPGPKGNDGAPGKNGERGGPG

GPGPQGPPGKNGETGPQGPPGPTGPGGDKGDTGPPGPQGLQGLPGTGGPPGENGKPGEPG

PKGDAGSPGAPGGKGDAGAPGERGPPGLAGAPGLRGGAGPPGPEGGKGAAGPPGPPGAAG

TPGLQGMPGERGGPGSPGPKGDKGEPGGPGADGVPGKDGPRGPTGPIGPPGPAGQPGDKG

EGGAPGLPGIAGPRGGPGERGEPGPPGPAGFPGAPGQNGEPGGKGERGAPGEKGEGGPPG

VAGPPGGSGPAGPPGPQGVKGERGSPGGPGAAGFPGARGLPGPPGNNGNPGPPGPGGSPG

KDGPPGPAGNTGAPGSPGVSGPKGDAGQPGEKGSPGPQGPPGAPGPLGIAGITGARGLAG

PPGMPGPRGNPGPQGVKGESGKPGANGLSGERGPPGPQGLPGLAGAAGEPGRDGNPGSDG

LPGRDGSPGGKGDRGENGSPGAPGAPGHPGPPGPVGPAGKSGDRGESGPAGPAGAPGPAG

SRGAPGLQGPRGDKGETGERGANGIKGHRGFPGNPGAPGSPGPAGQQGAIGSPGPAGPRG

PVGPSGPPGKDGTSGHPGPIGPPGPRGNRGERGSEGSPGHPGQPGPPGPPGAPGPCCGGV

GAAAIAGVGGEKAGGFAPYYGDEPMDFKINTDEIMTSLKSVNGQIESLISPDGTRKNPAR

NCRDLKFCHPELKSGEYWVDPNQGCKLDAIKVFCNMETGETCISASPLNVPRKHWWTDSG

AEKKHIWFGESMDGGFQFSYGNPELPEDVLDVQLAFLRLLSSRASQNITYHCKNSIAYMD

QASGNVKKALKLMGSNEGEFKAEGNSKFTYTVLEDGCTKHTGEWSKTVFEYRTRKAVRLP

IVDIAPYDIGGPDQEFGVDVGPVCFL

>Monodelphis domestica COL3A1

ISLNVLGNNKKLRNALMCVYTISEKAEEQERLGNVIDAGCTHLGQSYADRDVWKPEPCQI

CVCDSGSVLCDDIICDEQELDCPSPEIPFGECCPVCPQPPSPVSLNSRKKKRAVGNQQYI

SVISSIYLLPHPPRPPNGAPAGPKGDPGPPGIPGRNGDPGIPGQPGTPGTPGPPGICESC

PSINQNLPQFESYDVKSGGGGGPGLPGPPGIPGPSGPPGPPGHPGSPGTNGYQGPPGEPG

QAGPSGPPGPPGAMGPAGPAGKDGESGRPGRPGERGLPGAQGIKGPSGMPGFPGMKGHRG

FDGRNGEKGETGAPGLKGENGLPGDNGSPGPMGPRGLPGERGRPGPPGSAGSRGNDGAPG

ASGQMGPPGPPGPSGFPGSPGAKGEVGPAGSPGSNGSPGVRGEPGPQGHAGAQGPPGPPG

NNGSPGGKGETGPGGMPGPPGLSGARGPPGAAGTPGTPGTRGPAGEPGKNGGKGEPGPRG

ERGEAGIPGQPGAKGEDGKNGNPGEPGANGIPGSPGERGAPGFRGAAGSPGIPGEKGPAG

ERGGPGPAGPRGPVGEAGRDGNPGNPGQRGLPGSPGGPGSDGKPGPPGNQGESGRAGPPG

PSGPRGQPGVMGFPGPKGNDGAPGKNGERGNPGGPGPQGIAGKNGEAGPPGPPGSAGPPG

ERGETGAVGPQGFQGVPGGNGPPGENGKPGDPGPKGEAGVPGTPGGKGDAGPPGERGAPG

IPGNAGTRGVAGPPGPDGAKGPAGPPGPAGNSGPPGLQGMPGERGGAGLPGPKGDKGEPG

GKGGDGPPGKDGSRGPAGPIGPPGPAGQPGDKGEGGPAGLPGIAGPRGGPGERGEHGPPG

PAGFPGAPGQNGEPGAKGERGAPGEKGEGGPPGLAGPQGGPGPAGPPGPQGIKGDRGNPG

GPGTAGFPGARGLPGPPGSNGNPGPPGNNGAPGKDGPPGPAGNSGSPGNPGSPGPKGDAG

PPGEKGPSGSPGAPGSPGPSGVIGVTGARGLAGQPGSPGLRGGPGPQGPKGEDGKPGTSG

QNGERGPQGPQGLPGLSGMPGEPGRDGNPGSDGQPGRDGAPGGKGDRGEGGPPGSPGSPG

HPGPPGPTGPAGKSGDRGESGPAGPGGPPGPAGARGAPGAQGPRGDKGEPGERGNNGIKG

HRGFPGTPGPPGASGQPGHQGAVGSPGPAGPRGPVGPHGPPGKDGLSGHAGPIGPPGPRG

NRGERGSDGAPGHPGNPGPPGQPGAPGPCCGGGGGAAIAGFGEKASPFYGDEPTDFKMNT

EEILSSLKSVSGQVESIISPDGSRKNPARNCRDLKFCHPELKSGQYWIDPNQGCKMDAIQ

VYCNMETGETCINASPMNIPRKNWWTDSSAEKKYVWFGESMNGGFQFSYGNPDLPEDVLE

VQLAFLRLLSSRASQNITYHCKNSIAYMDQASGNVKKALKFMGSHEGEFKAEGNSKITYT

VLEDGCSKHTGEWGQTVFEYRTRKAMRLPIIDIAPFDIGGPDQEFGADIGPVCFL

>homo sapiens COL5A2

mpgergrlgpqgapgqrgahgmpgkpgpmgplgipgssgfpgnpgmkgeagptgppgepg

dpgpmgpigsrgpegppgkpgedgepgrngnpgevgfagspgargfpgapglpglkghrg

hkglegpkgevgapgskgeagptgpmgdpgtvgppgpvgergapgnrgfpgsdglpgpkg

aqgergpvgssgpkgsqgdpgrpgepglpgargltgnpgvqgpegklgplgapgedgrpg

ppgsigirgqpgsmglpgpkgssgdpgkpgeagnagvpgqrgapgkdgevgpsgpvgppg

lagergeqgppgptgfqglpgppgppgeggkpgdqgvpgdpgavgplgprgergnpgerg

epgitglpgekgmagghgpdgpkgspgpsgtpgdtgppglqgmpgergiagtpgpkgdrg

gigekgaegtagndgarglpgplgppgpagptgekgepgprglvgppgsrgnpgsrgeng

ptgavgfagpqgpdgqpgvkgepgepgqkgdagspgpqglagspgphgpngvpglkggrg

tqgppgatgfpgsagrvgppgpagapgpagplgepgkegppglrgdpgshgrvgdrgpag

ppggpgdkgdpgedgqpgpdgppgpagttgqrgivgmpgqrgergmpglpgpagtpgkvg

ptgatgdkgppgpvgppgsngpvgepgpegpagndgtpgrdgavgergdrgdpgpaglpg

sqgapgtpgpvgapgdagqrgdpgsrgpigppgragkrglpgpqgprgdkgdhgdrgdrg

qkghrgftglqglpgppgpngeqgsagipgpfgprgppgpvgpsgkegnpgplgpigppg

vrgsvgeagpegppgepgppgppgppghltaalgdimghydesmpdplpeftedqaapdd

knktdpgvhatlkslssqietmrspdgskkhpartcddlklchsakqsgeywidpnqgsv

edaikvycnmetgetcisanpssvprktwwaskspdnkpvwygldmnrgsqfaygdhqsp

ntaitqmtflrllskeasqnityicknsvgymddqaknlkkavvlkgandldikaegnir

fryivlqdtcskrngnvgktvfeyrtqnvarlpiidlapvdvggtdqefgveigpvcfv

>Mus musculus COL5A2

MGERGAPGNRGFPGSDGLPGPKGAQGERGPVGSSGPKGGQGDPGRPGEPGLPGARGLTGN

PGVQGPEGKLGPLGAPGEDGRPGPPGSIGIRGQPGSMGLPGPKGSSGDLGKPGEAGNAGV

PGQRGAPGKDGEVGPSGPVGPPGLAGERGEQGPPGPTGFQGLPGPPGPPGEGGKAGDQLI

PSEPDTHASYVHNGERGNPGERGEPGITGLPGEKGMAGGHGPDGPKGNPGPTGTIGDTGP

PGLQGMPGERGIAGTPGPKGDRGGIGEKGAEGTAGNDGARGLPGPLGPPGPAGPTGEKGE

PGPRGLVGPPGSRGNPGSRGENGPTGAVGFAGPQGPDGQPGVKGEPGEPGQKGDAGSPGP

QGLAGSPGPHGPHGVPGLKGGRGTQGPPGATGFPGSAGRVGPPGPAGAPGPAGPAGEPGK

EGPPGLRGDPGSHGRVGDRGPAGPPGSPGDKGDPGEDGQPGPDGPPGPAGTTGQRGIVGM

PGQRGERGMPGLPGPAGTPGKVGPTGATGDKGPPGPVGPPGSNGPVGEPGPEGPAGNDGT

PGRDGAVGERGDRGDPGPAGLPGSQGAPGTPGPVGAPGDAGQRGEPGSRGPVGPPGRAGK

RGLPGPQGPRGDKGDNGDRGDRGQKGHRGFTGLQGLPGPPGPNGEQGSAGIPGPFGPRGP

PGPVGPSGKEGNPGPLGPIGPPGVRGSVGEAGPEGPPGEPGPPGPPGPPGHLTAALGDIM

GHYDENMPDPLPEFTEDQAAPDDTNKTDPGIHVTLKSLSSQIETMRSPDGSKKHPARTCD

DLKLCHPTKQSGEYWIDPNQGSAEDAIKVYCNMETGETCISANPASVPRKTWWASKSPDN

KPVWYGLDMNRGSQFTYGDYQSPNTAITQMTFLRLLSKEASQNLTYICRNTVGYMDDQAK

NLKKAVVLKGSNDLEIKGEGNIRFRYTVLQDTCSKRNGNVGKTIFEYRTQNVARLPIIDV

GPVDIGNADQEFGLDIGPVCFM

>Rattus norvegicus COL5A2

GPVGPPGSNGPVGEPGPEGPAGNDGTPGRDGAVGERGDRGDPGPAGLPGSQGAPGTPGPV

GAPGDAGQRGEPGSRGPVGPPGRAGKRGLPGPQGPRGDKGDNGDRGDRGQKGHRGFTGLQ

GLPGPPGPNGEQGSAGIPGPFGPRGPPGPVGPSGKEGNPGLLGPIGPPGVRGSVGEAGPE

GPPGEPGPPGPPGPPGHLTAALGDIMGHYDENMPDPLPEFTEDQAAPDDTNKTDPGVHAT

LKSLSSQIETMRSPDGSKKHPARTCDDLKLCHPTKQSGEYWIDPNQGSAEDAIKVYCNLE

TGETCISANPASVPRKTWWASKSPDNKPVWYGLDMNRGSQFTYGDYKSPNTAITQMTFLR

LLSKEASQNLTYICKNTVGYMDDQTKNLKKAVVLKGSNDLEIKGEGNIRFRYTVLQDTCS

KRNGNVGKTVFEYRTQNVARLPIIDIGPVDIGNADQEFGFEIGPVCFM

>Bos taurus COL5A2

mmanwvearplliltvllgqfvsikaqeededegygeeiactqngqmylnrdiwkpapcq

icvcdngailcdkiqcqdvlecadpvtppgeccpvcphttgggntnfgrgrkgqkgepgl

vpvvtgirgrpgpagppgsqgprgergpkgkpgprgpqgidgepgvpgqpgapgppghps

hpgpdgmsrpfsaqmagldeksglgsqvglmpgsvgpvgprgpqglqgqqggvgpagppg

epgdpgpmgpvgargpegppgkpgedgepgrngkpgevgfagspvtpsslfpffppqpan

kacrqrqvmnlskgqdsipnskgeagptgpmgamgpmgprgmpgergrlgpqgapgqrga

hgmpgkpgpmgplgipgsagfpgnpgmkgeagptgargpegpqgqrgetgppgpvgsqgl

pgavgtdgtpgakgptgspgtsgppglagppgspgpqgstgppgirgqpgdpgvpgfkge

agpkgepgphgvqgpigppgeegkrgprgdpgtvgppgpvgergapgnrgfpgsdglpgp

kgaqgergpvgssgpkggqgdpgrpgepglpgargltgnpgvqgpegklgplgapgedgr

pgppgsigirgqpgsmglpgpkgssgdpgkpgeagnagvpgqrgapgkdgevgpsgpvgp

pglagergeqgppgptgfqglpgppgppgeagkpgdqgvpgdpgavgplgprgergnpge

rgepgitglpgekgmagghgpdgpkgspgppgtpgdtgppglqgmpgergiagtpgpkgd

rggigekgaegtagndgarglpgplgppgpsgptgekgepgprglvgppgsrgnpgsrge

ngptgavgfagpqgpdgqpgvkgepgepgqkgdagspgpqglagspgphgpngvpglkgg

rgtqgppgatgfpgsagrvgppgpagapgpagplgepgkegppglrgdpgshgrvgdrgp

agppggpgdkgdpgedgqpgpdgppgpagttgqrgivgmpgqrgergmpglpgpagtpgk

vgptgapgdkgppgpvgppgsngpvgepgpegpagndgtpgrdgavgergdrgdpgpagl

pgsqgapgtpgpvgapgdagqrgdpgsrgpigppgragkrgllgptgeqgsagipgpfgp

rgppgpvgpsgkegspgplgpigppgvrgsvgeagpegppgepgppgppgppghltaalg

dimghyddsmpdplpeftedqaapddknktdpgvhatlkslssqietmrspdgsrkhpar

tcddlklchsakksgeywidpnqgsaedaikvycnmetgetcisanpssvprktwwasks

pdnkpvwygldmnrgsqfvygdhqspnaaitqmtflrllskeasqnityicknsvgymdd

qaknlkkavvlkgsndleikaegnvrfryivlhdscskrngnvgktifeyrtqnvarlpi

idlapvdvgstdqefgveigpvcfv

>macaca mulatta COL5A2

MSRPFSAQMAGLDEKSGLGSQVGLMPGSVLLYCVPVKSPACFIHNYGGAGPTGPPGEPGD

PGPMVNVEKGEPGRNGNPGEVGFAGSPGARGFPGAPGLPGLKGHRGHKGLEGPKGEVGAP

GSKGEAGPTGSMGAMGPLGPRGMPGERGRLGPQGAPVSISAFTYTAEIERQGPLGIPGSS

GFPGNPGMKGEAGPTGARGPEGPQGQRGETGPPGPVGSPGLPGAIGTDGTPGAKGPTGSP

GTSGPPGSAGPPGSPGPQGSTGPQGIRGQLGPHGIQGPIGPPGEEGKRGPRGDPGTVGPP

GPVGERGAPGNRGFPGSDGLPGPKGAQGERGPVGSSGPKGSQGDPGRPGEPGLPGARGLT

GNPGVQGPEGKLGPLGAPGEDGRPGPPGSIGIRGQPGSMGLPGPKGSSGDPGKPGEAGNA

GVPGQRGAPGKDGEVGPSGPVGPPGLAGERGEQGPPGPTGFQGLPGPPGPPGEGGKPGDQ

VSVEIIIMATLYIAGSGERGNPGERGEPGITGLPGEKGMAGGHGPDGPKGSPGPSGTPGD

TGPPGLQGMPGERGIAGTPGPKGDRGGIGEKGAEGTAGNDGARVKFFSAVSPYTVTNILK

GEPGPRGLVGPPGSRGNPGSRGENGPTGAVGFAGPQGPDGQPGVKGEPGEPGQKGDAGSP

GPQGLAGSPGPHGPNGVPGLKGGRGTQGPPGATGFPGSAGRVGPPGPAVSQLSESIMHTG

ISAKQIILNNIVFLGGAGPLGGPGDKGDPGEDGQPGPDGPPGPAGTTGQRGIVGMPGQRG

ERGMPGLPGPAGTPGKVGPTGATGDKGPPGPVGPPGSNGPVGEPGPEGPAGNDGTPGRDG

AVGERGDRGDPGPAGLPGSQGAPGTPGPVGAPGDAGQRGDPVRRESVSHLNTIIKSKILG

PQGPRGDKGDHGDRGDRGQKGHRGFTGLQGLPGPPGPNGEQGSAGIPGPFGPRGPPGPVG

PSGKEGNPGPLGPIGPPGVRGSVGEAGPEGPPGEPGPPGPPGPPGHLTAALGDIMGHYDE

SMSDPLPEFTEDQAAPDDKNKTDPGVHATLKSLSSQIETMRSPDGSKKHPARTCDDLKLC

HSAKQSGEYWIDPNQGSVEDAIKVYCNMETGETCISANPSSVPRKTWWASKSPDNKPVWY

GLDMNRGSQFAYGDHQSPNTAITQMTFLRLLSKEASQNITYICKNSVGYMDDQAKNLKKA

VVLKGANDLDIKAEGNIRFRYIVLQDTCSKRNGNVGKTVFEYRTQNVARLPIIDLAPVDV

GGTDQEFGIEIGPVCFV

>monodelphis domestica COL5A2

VMANWVEARTLFILTFLLGQFVSIKAQDEGEDDGYDQEIACTQNGQMYLNRDIWKPAPCQ

ICVCDNGAILCDQIQCQEVLDCADPITPSGECCPVCPNTAGGGNNPGFGKNSEGQKGEPG

LVPVVTGIRGRPGPSGPPGSQGPRGERGPKGRPGARGPQGIDGEPGIPGQPGAPGPPGHP

SHPGPDGISRPFSSQMAGLDEKSGLGSQMGLMPGSVGPVGPRGPQGLQGKQGGVGPTGPP

GEPGEPGPMGPVGARGPEGPPGKPGEDGEAGRNGNPGEVGFSGSPGARGFPGAPGFPGLK

GHRGHKGLDGPKGEVGATGAKGEAGPTGAMGAMGPLGPRGMPGERGRLGPQGAPGQRGAH

GMPGKPGPMGPLGITGSAGFPGNPGMKGEAGPTGARGPEGPQGQRGETGAPGPVGSQGLP

GTVGTDGTPGAKGPTGSPGTSGPPGSTGPPGSPGPQGSTGQPGIRGQTGDPGFPGFKGEA

GPKGEPGPHGIQGPIGPVGEEGKRGPRGDPGTVGPPGPVGERGSPGNRGFPGSDGLPGPK

GAQGERGPAGSSGPKGGQGDPGRTGEPGLPGARGLTGNPGVQGPEGKLGPLGAPGEDGRP

GPAGSIGIRGQPGSMGLPGPKGSSGDPGKPGEAGNAGVPGQRGAPGKDGEVGPSGPVGPS

GLAGERGEQGPPGPTGFQGLPGTPGPPGEGGKPGDQGVPGDPGAVGPLGPRGERGNPGER

GEPGITGLPGEKGMAGGHGPDGPKGSPGPSGTPGDAGPPGLQGMPGERGIAGTPGPKGDR

GGLGEKGSEGTAGNDGARGLPGPLGPPGPAGPTGEKGEPGPRGLVGPPGSRGTPGSRGEN

GPTGAVGFAGPPGPDGQPGVKGEPGEPGQKGDAGSPGPQGLAGSPGPHGPNGVPGLKGGR

GTQGPPGATGFPGSAGRVGPPGPTGAPGPAGPLGEPGKEGPPGLRGDPGAHGRIGDRGPA

GPPGGPGDKGDSGEDGQPGPDGPPGPAGTTGQRGIVGMPGQRGERGMPGLPGPAGTPGKA

GPTGAPGDKGPSGPVGPPGANGPVGEAGPEGPAGNDGTPGRDGAVGERGDRGDPGPAGLP

GSQGASGTPGPVGATGDAGPRGDPGSRGPVGPPGRAGKRGLPGPQGPRGDKGDHGDRGDR

GQKGHRGFTGLQGLPGPPGPNGEQGSAGIPGPFGPRGPPGPVGPSGKEGNAGPLGPIGPP

GMRGTVGEAGPEGPPGEPGPPGPPGPPGHLTAPLGDIMGHYDESMSDPLPEFTEDQAAPD

DKNKTDPGVHATLKSLSSQIETMRSPDGSKKHPARTCDDLKLCHPSKPSGHYWIDPNQGC

VEDALKVYCNMDTGETCIPANPSTVPRKTWWTSRSSDYKPVWYGLDMNRGSQFVYGDSQS

PNTAVTQMTFLRLLSKEASQNITYICKNSVGYMDDQAKNLKKAVILKGANDLEIKAEGNS

RFRYTILEDTCTRRNGNVGKTVFEYRTQNVARLPIVDLAPVDIGKADQEFGIEIGSVCFV

>danio rerio COL5A2

delsctengqvytnrdiwkpepcricvcdsgtilcdevqcdevsncakvvippgeccpvc

qgdaaeggngrpggriakgqkgepgvvphivgirgrpgpmgppgapgfrgdsgpkgrpgl

rgppgydgepeagtpgsagemgfpgsprisrdtgaprlkgpqkyhereqfnkipdesgkg

hqgpdglkgergalgskgtrggqgnmgkpgpmgpmgingapgypgspgmkgqpgptgvrg

pegpqgqrgetghqgrsgptgqqgpmgtdggpgskgpvgdlgvagfkgeagpkgepgppg

sqgvlgpqgeegkrgprgdpgsigppgpvgergapgnrgfpgqdglqgpkgaqgdrgtsg

tsgpkgstgdpgrtgepglpgargltgtpgvqgaegkpgplgapgedgrpgpagstgtrg

pigtmgpigpkgfsgdpgkageqgsagvpgqrgppgkdgevgpagppgphgtagergeqg

ppgmhgfqgipgeggpvgqigprgspgptgtlgnqgppglqgmpgergisgppgpkgdrg

aigekgsegtpgsdgargltgpigpagphgpsgekgepgpkgppgpqgsrampgsrgepg

pigavgfvgppgldgqpgpkgppgepgqkgdagspgpqglagppgpqgklgvagpkggrg

tqgapgptgfpgsagrvgppgptgpigepgplglpgkegppglrgdhgppgrqgergppg

paggpgdkgdsgedgppgpdgppgpagttgqrgivglpgqrgergmaglpgpagppgkqg

tsgapgdkgppgpvgvpgangprgdpgpdgpagsdgppgkegvigaqgdrgdpgpegvlg

tvgqpgppgpvgatggvgkrgepgskgpsgppgpagkrgltgpqgprgdkgdigdhgerg

qkghrgfaglqglpgspgttgeqgatgivgpsgqrgppgpigppgkegyigqpgpmgppg

srgssgdigpeasppgppgpagpptaavddlfggahdfdsgpsppdnnqdealpmsnsts

vfqadpgvqatlkalssqidnmrspdgsrkhpartcedlkqcypqkksgeywvdpnqgsa

edaikiycnmdtgetcisaipssiprkswwsspgnkpvwfganmnggtyftygnkdqpan

tvtvqmtfirllskeasqtityhckntvgykdestgnlkkavilkasndlelkaegnnrf

rytvledscsqangnwgktvfeyrtqktarlpivdiapvdiggsdqefgidigpvcfl

>strongylocentrotus purpuratus COLP1ALPHA

mysfvdlavchrrkilltllvlfavqsfsngqaagqysegprgdkgqkgepgdadinsan

fppglpgpvgppgpsgpsgpagnngppgpngprgnpgmdgltglpgipgppgppgksgsl

vasaqtssfnkgpslagyqypqaqaagtpgprgppgppgsrgpqgltgpsgpsgetgpsg

nsgppgpsglpgrpgsdgddgtpgsqgqrgpagtpgsrgtpgmpgapgmkghqglpgmtg

skgergeggergsdgspgpvgapgpagpsgqpgergrtgpagsqgdrgadgatgsqgppg

stgpagapgmpgisgakgdagspgargspglqgargergsegsqgqtgppgvpgrdgsng

akgsagpsgaqgtpgfpgargppgpagspgpagskgdqgnpgqpgaqgesgplgprgetg

pagppgaqgesgergsrgalgpagppggvgergpmgppgmsgapgapgakgdrglpgerg

sagskgsagesgrpgepgmpgqrgltgppgkqgrdgkpgpagapgepgnsgpagasgqrg

lpglvglpgpqgqrgergedggqgspgapgltgepgkrgepgvagppgpqgsagergnqg

pqgqagsmgppgppgasgdagaqgdngppgesgpegppgargergapgergpqgltgaqg

rrgiggragnygatgppgqkgemgppgnvglqgppgelgpsgppgargppgpsgspgpdg

pagaegdrgpvgpmgpsgpsgmpgergdngepgpggapgqrgdpgdlgpqgspgspgfag

ppgrsgnpgpqgelgptgargetggpgpsgptgdpgpqgplgapgqqgergetgpqgqgg

ppgpigslgapgaqgppgptgpsgnagspgqpgargepgqsgspgqpglagrtgpsgerg

dkgndgqsgppgppgpagpagqsgilglaggsgprgpggpagppgaagsrgpagksgdrg

spgavgpagnpgpagengmpgsdgndgapgpqgsrgekgdtgasgangspgapgpigapg

aagasgprgetgstgapgplgptgargstgpagpsgpsgpagergetgpaghkghpgvsg

lpglqgtsgpmgepgapgeqgqqgtrglpgargsngndgpsgprgfdgpegprgprgegg

ssgppgppgppgppgppgqvqssygvrypsfqsggkgqgsspygyayrddskndavkiqd

tellgaisalgqqielikapqgkaktnparsckdvflnnveaesgyywvdpnlgcqkdai

qvyceaetgatcvpstnnvvsnmtwyvgktkraffssmhggdkfayiedstqmtflrlls

tsarqtvtyfcknvqgnpiflsssdvelvddqdskfnyrtledgcsssssqwgksvyeye

tkkttrlpivdfapgevgsesqmfglemgpvcfs

>apis mellifera (XP_393523)

clmedesvqaillpdnleepylsginyscimpgkrfqsfsnlsggektlatiaflfaihs

frpapffildeidaaldiiniknvvrlidskknemqfiiislkreiyscadvligvcsds

lmgpkalqlaaafvvvqlllpqadsvrtklrkqrskslqaqqpangdylydyyenyddyd

ernetittttppppsvtsntasrgglptetfyrsalstdspaqttirsfiqevrddlhtt

pernplpteivpgesnminavavpgprgepgrpgdigrpgdvgfpgqpgtpgipgppgpp

gpipdvsmyyqqlalsqasqdkgptgaaaslynpdaylqtqvgpvgprgppgppgpsgpq

gfqgvrgepgepgtpgppgapgprglpgltgkdgntgedgepgppgsvgspgfpgnpgik

gdqgaqgpkgsqgiqgprgetgrpgqpgesgpqgpqgkdgipgekgstgapglvgapgfp

gargqagipgnpgipgakgmpgfpgergfkgdngvkgdagipgprglpgppgnegkrgkr

gmrgpigtpgptgergapgapglpgpdgpmgskgqsgdrgpigpigpkgvigdpgrpgsp

glqpgnpgergiqgadgkpgeqgppglqglpgplglpgekgypgepgkagepgnpgpsgp

rgdtgkegapgpqgspgpsgtdgergppgspgprgfqgfpgatgnagtpgkdgepgvqgp

pgspgvpgnrgergfpgergaaggpglmgprgepgvqgldgppglpgvkgdkgisgpsgl

vglpglrgapgesgpkgergpigpvglegppgrigergpqgqigppgppgepaergdpgl

pglpgeigapgnsgergpqglqglqgfpgpqgliglpglkgdrgypglkgdqgnsgppgs

kgntgeagrpgnpgpignpglpgadgnkgesgsqgppgipgpqgpqgspgerglpglpgp

sgpigargirgapgesgtpgkpgpegppglvgqigpigppgppgeigaegssgkpgppgi

agrpgdkgppgavgqtglpgspglpgppgqagspgsagergskgetgpqgvegqqgprgk

pgpiglqgpkgekgipglpglpgkdgepgsrgspgrdgnpgpiglignpgpkgppgdegr

hgppgspgppgppgppgelglgydaaslaallgqgqikgpdpligdepprmfsqdmteqe

rqeliqkaykqlkssfqkfikpdgeknspaktcrdlysaypnklsgeywidpnegdarda

ilvycdakkratcllpnpvhspeiihitdqpetwlseiengmkitykadsnqigflqlls

knayqnityhcknsigyfdserktyrkgmkfltwndaeltprgnqrlryemiidecrthn

gkwgktiisyqtdktirlpiidvalrdigkpnqsfyieignvcye

**ERBB receptor protein tyrosine kinase**

>Homo sapiens ERBB4

MKPATGLWVWVSLLVAAGTVQPSDSQSVCAGTENKLSSLSDLEQQYRALRKYYENCEVVM

GNLEITSIEHNRDLSFLRSVREVTGYVLVALNQFRYLPLENLRIIRGTKLYEDRYALAIF

LNYRKDGNFGLQELGLKNLTEILNGGVYVDQNKFLCYADTIHWQDIVRNPWPSNLTLVST

NGSSGCGRCHKSCTGRCWGPTENHCQTLTRTVCAEQCDGRCYGPYVSDCCHRECAGGCSG

PKDTDCFACMNFNDSGACVTQCPQTFVYNPTTFQLEHNFNAKYTYGAFCVKKCPHNFVVD

SSSCVRACPSSKMEVEENGIKMCKPCTDICPKACDGIGTGSLMSAQTVDSSNIDKFINCT

KINGNLIFLVTGIHGDPYNAIEAIDPEKLNVFRTVREITGFLNIQSWPPNMTDFSVFSNL

VTIGGRVLYSGLSLLILKQQGITSLQFQSLKEISAGNIYITDNSNLCYYHTINWTTLFST

INQRIVIRDNRKAENCTAEGMVCNHLCSSDGCWGPGPDQCLSCRRFSRGRICIESCNLYD

GEFREFENGSICVECDPQCEKMEDGLLTCHGPGPDNCTKCSHFKDGPNCVEKCPDGLQGA

NSFIFKYADPDRECHPCHPNCTQGCNGPTSHDCIYYPWTGHSTLPQHARTPLIAAGVIGG

LFILVIVGLTFAVYVRRKSIKKKRALRRFLETELVEPLTPSGTAPNQAQLRILKETELKR

VKVLGSGAFGTVYKGIWVPEGETVKIPVAIKILNETTGPKANVEFMDEALIMASMDHPHL

VRLLGVCLSPTIQLVTQLMPHGCLLEYVHEHKDNIGSQLLLNWCVQIAKGMMYLEERRLV

HRDLAARNVLVKSPNHVKITDFGLARLLEGDEKEYNADGGKMPIKWMALECIHYRKFTHQ

SDVWSYGVTIWELMTFGGKPYDGIPTREIPDLLEKGERLPQPPICTIDVYMVMVKCWMID

ADSRPKFKELAAEFSRMARDPQRYLVIQGDDRMKLPSPNDSKFFQNLLDEEDLEDMMDAE

EYLVPQAFNIPPPIYTSRARIDSNRSEIGHSPPPAYTPMSGNQFVYRDGGFAAEQGVSVP

YRAPTSTIPEAPVAQGATAEIFDDSCCNGTLRKPVAPHVQEDSSTQRYSADPTVFAPERS

PRGELDEEGYMTPMRDKPKQEYLNPVEENPFVSRRKNGDLQALDNPEYHNASNGPPKAED

EYVNEPLYLNTFANTLGKAEYLKNNILSMPEKAKKAFDNPDYWNHSLPPRSTLQHPDYLQ

EYSTKYFYKQNGRIRPIVAENPEYLSEFSLKPGTVLPPPPYRHRNTVV

>Mus musculus ERBB4

MKLATGLWVWGSLLMAAGTVQPSASQSVCAGTENKLSSLSDLEQQYRALRKYYENCEVVM

GNLEITSIEHNRDLSFLRSIREVTGYVLVALNQFRYLPLENLRIIRGTKLYEDRYALAIF

LNYRKDGNFGLQELGLKNLTEILNGGVYVDQNKFLCYADTIHWQDIVRNPWPSNMTLVST

NGSSGCGRCHKSCTGRCWGPTENHCQTLTRTVCAEQCDGRCYGPYVSDCCHRECAGGCSG

PKDTDCFACMNFNDSGACVTQCPQTFVYNPTTFQLEHNFNAKYTYGAFCVKKCPHNFVVD

SSSCVRACPSSKMEVEENGIKMCKPCTDICPKACDGIGTGSLMSAQTVDSSNIDKFINCT

KINGNLIFLVTGIHGDPYNAIDAIDPEKLNVFRTVREITGFLNIQSWPPNMTDFSVFSNL

VTIGGRVLYSGLSLLILKQQGITSLQFQSLKEISAGNIYITDNSNLCYYHTINWTTLFST

INQRIVIRDNRRAENCTAEGMVCNHLCSNDGCWGPGPDQCLSCRRFSRGKICIESCNLYD

GEFREFENGSICVECDSQCEKMEDGLLTCHGPGPDNCTKCSHFKDGPNCVEKCPDGLQGA

NSFIFKYADQDRECHPCHPNCTQGCNGPTSHDCIYYPWTGHSTLPQHARTPLIAAGVIGG

LFILVIMALTFAVYVRRKSIKKKRALRRFLETELVEPLTPSGTAPNQAQLRILKETELKR

VKVLGSGAFGTVYKGIWVPEGETVKIPVAIKILNETTGPKANVEFMDEALIMASMDHPHL

VRLLGVCLSPTIQLVTQLMPHGCLLDYVHEHKDNIGSQLLLNWCVQIAKGMMYLEERRLV

HRDLAARNVLVKSPNHVKITDFGLARLLEGDEKEYNADGGKMPIKWMALECIHYRKFTHQ

SDVWSYGVTIWELMTFGGKPYDGIPTREIPDLLEKGERLPQPPICTIDVYMVMVKCWMID

ADSRPKFKELAAEFSRMARDPQRYLVIQGDDRMKLPSPNDSKFFQNLLDEEDLEDMMDAE

EYLVPQAFNIPPPIYTSRTRIDSNRNQFVYQDGGFATQQGMPMPYRATTSTIPEAPVAQG

ATAEMFDDSCCNGTLRKPVAPHVQEDSSTQRYSADPTVFAPERNPRGELDEEGYMTPMHD

KPKQEYLNPVEENPFVSRRKNGDLQALDNPEYHSASSGPPKAEDEYVNEPLYLNTFANAL

GSAEYMKNSVLSVPEKAKKAFDNPDYWNHSLPPRSTLQHPDYLQEYSTKYFYKQNGRIRP

IVAENPEYLSEFSLKPGTMLPPPPYRHRNTVV

>Rattus norvegicus ERBB4

MKLATGLWVWGSLLVAARTVQPSASQSVCAGTENKLSSLSDLEQQYRALRKYYENCEVVM

GNLEITSIEHNRDLSFLRSIREVTGYVLVALNQFRYLPLENLRIIRGTKLYEDRYALAIF

LNYRKDGNFGLQELGLKNLTEILNGGVYVDQNKFLCYADTIHWQDIVRNPWPSNMTLVST

IGSSGCGRCHKSCTGRCWGPTENHCQTLTRTVCAEQCDGRCYGPYVSDCCHRECAGGCSG

PKDTDCFACMNFNDSGACVTQCPQTFVYNPTTFQLEHNFNAKYTYGAFCVKKCPHNFVVD

SSSCVRACPSSKMEVEENGIKMCKPCTDICPKACDGIGTGSLMSAQTWWTSFQYKYCLCY

KIIVLNLAASLLDFSDPYNAIDAIDPEKLNVFRTVREITGFLNIQSWPPNMTDFSVFSNL

VTIGGRVLYSGLSLLILKQQGITSLQFQSLKEISAGNIYITDNSNLCYYHTINWTTLFST

VNQRIVIRDNRRAENCTAEGMVCNHLCSNDGCWGPGPDQCLSCRRFSRGKICIESCNLYD

GEFREFENGSICVECDSQCEKMEDGLLTCHGPGPDNCTKCSHFKDGPNCVEKCPDGLQGA

NSFIFKYADQDRECHPCHPNCTQGCNGPTSHDCIYYPWTGHSTLPQHARTPLIAAGVIGG

LFILVIMALTFAVYVRRKSIKKKRALRRFLETELVEPLTPSGTAPNQAQLRILKETELKR

VKVLGSGAFGTVYKGIWVPEGETVKIPVAIKILNETTGPKANVEFMDEALIMASVDHPHL

VRLLGVCLSPTIQLVTQLMPHGCLLEYVHEHKDNIGSQLLLNWCVQIAKGMMYLEERRLV

HRDLAARNVLVKSPNHVKITDFGLARLLEGDEKEYNADGGKMPIKWMALECIHYRKFTHQ

SDVWSYGVTIWELMTFGGKPYDGIPTREIPDLLEKGERLPQPPICTIDVYMVMVKCWMID

ADSRPKFKELAAEFSRMARDPQRYLVIQGDDRMKLPSPNDSKFFQNLLDEEDLEDMMDAE

EYLVPQAFNIPPPIYTSRTRIDSNRSEIGHSPPPAYTPMSGNQFVYQDGGFATQQGMPMP

YRATTSTIPEAPVAQGATAEMFDDSCCNGTLRKPVVPHVQEDSSTQRYSADPTVFAPERN

PRAELDEEGYMTPMHDKPKQEYLNPVEENPFVSRRKNGDLQALDNPEYHSASSGPPKAED

EYVNEPLYLNTFTNALGNAEYMKNSLLSVPEKAKKAFDNPDYWNHSLPPRSTLQHPDYLQ

EYSTKYFYKQNGRIRPIVAENPEYLSEFSLKPGTMLPPPPYRHRNTVV

>Xenopus tropicalis ERBB4

PLFSIAVCAGTENKLSSLSDLEQQYKALRKYYENCEVVMGNLEITSIEPGRNLSFLRSIR

EVTGYVLVALNQFDYLPLENLRIIRGIKLYEDRYALAIFLNYRKDGNYGLRELGLRNLTG

IISIFSILDKKKNILVLKNMYWEKKIRNISKWPPKCVHFSSFCENGGGRCPKSCMGRCWG

PSDQHCQRLTKTVCAEQCDGRCYGSYVSDCCHRECAGGCTGPKDTDCFSCMKFNDSGSCV

TQCPQPNIYNPTAFQLEQNPKAKYTYGAFCVNKCPHNFVVDDSSCVRACPSNKMEVEENG

IKMCKPCAGICPKVCDGIGTGSLQSAQTVDSSNIDKFINCTKINGNLHFLVTGIDGDPYH

NIAALEPQKLNIFFTVREITGFLNIQSWPRNMTDLSVFSNLVNIGGRVLYSGISLLILKQ

QSLNSLQFQSLKEISAGNVYITDNRNLCYYHTVNWTSLFRTSNQRTTIRENKKPENCTAE

GMVCDPLCSNAGCWGPGANQCLSCRYFSRGKSCVRSCNLYKGESREFVNGSVCVMCDSQC

EKMEDSIVTCYGPGPDHCTKCSNFKDGPNCVEKCPEGLQGANSFIFKYADEDKECHPCHP

NCTDGCNNPTSHDCLWNDPLTLPQHARTPLIAASVIGGLFIIVIMALTFAVYIRRKSIKK

KRALRRFLETELVEPLTPSGTAPNQAQLRILKETELKRIKILGSGAFGTVYKGIWVPEGE

TVKIPVAIKILNETTGPKANAEFMDEALIMASMDHPHLVRLLGVSLSPTIQLVTQLMPHG

CLLDYVHEHKDNIGSQLLLNWCVQIAKGMMYLEERRLVHRDLAARNVLVKSPNHVKITDF

GLARLLEGDEKEYNADGGKMPIKWMALECIHYRKFTHQSDVWSYGVTVWELMTFGGKPYD

GIPTREIPDLLEKGERLPQPPVCTIDVYMVMVKCWMIDADSRPKFKELAAEFSRMARDPQ

RYLVIQGDDRMKLPSPNDSKFFQNLLDEEDLGDMMDAEEYLVPQAFNIPPPIYTSRTRID

SNRSEIGHSPPPAYTPMSGNQFVYRDGGYTTQEAVNLPFRPTACPITELSTAQGATAEVF

DDVCCNGTLCKQVVSERIPEESSLQRYSADPTAFVPERNPRGVVGEDGYMTPMKEKPKSD

YLNPVEENPFVSRRKNGDVQAVEKPEYNNGPSTQSKSEDEYVKERLYLNSFAKPKENAEY

LKNNGLTVPEKSKKAFDNPEYWNHSLPPRSTIHHPDYLHEYSTKYFYKQNGRIRPVVAEN

PEYLTEFTLKPGTVLPPPPYRHRNTVV

>Tetraodon nigroviridis ERBB4

VCPGTDNKLSSLSDLDQQYRTLKKLYENCEVVMGNLEITSIERNRNLSFLKLPGQNQRRE

SSESGSFASCVPVLALTGWDQCQTEMRRIKLSLVLSVHLRLANAASVVPLTPVISHYQSI

REVTGYVLVALNQFDYLPLENLRIIRGITLYEGRYSLAIFLNYRRDGYYGLRQLGLRNLT

AGLLLSGSLKRSLKRRLSAYSCAIFMVISAKETPLISTCSPSPCDAATAPCSGLAFWPLE

ILHGGVLVDQNKFLCHADTIHWRDIIKNSLAELLVVSSNSSNPGCRRCHRSCNGRCWGHQ

EDQCQTLTKTVCAEQCDGRCFGPYVSDCCHRECAGGCSGPKDTDCFACTNFNDSGACVTQ

CPQPFVYNPTSFQLEHNPRAKYTYGAFCVKKCPHNFVVDHSSCVRACPSNKMEVEKNRIK

MCIPCTDICPKVCDGIGTGSLLKNQTVDASNIDKFVNCTKINGNLIFLITGIKGDMYHGI

GPLDPERLNVFRTVREITGYLNIQAWPENMTDLGVFSNLATIGGRSLYSISGISLLILKQ

RWISSFKFQSLDEISAGNVYISNNSGLCFYNTVNWTSLFRTSNQKVLIRNNRDPKECSQL

QMVCDGMCSEEGCWGSGPDQCLSCRYFKRGRSCVESCNLFEGDVPEFANGSVCLECDSQC

ERMDGNALTCLGQGPDQCVKCRHFKDGPNCVEKCPDGLQGANSFIFKYAKANNECHPCHA

NCTQGCVGPRLQDCVGMMDRTPLIAAGIIGGLFIIVILALSVAVYARRKSIKKKRALRRF

LETELVEPLTPSGTAPNQAQLRILKETELKRVKILGSGAFGTVYKGIWVPEGETVKIPVA

IKILNEATGPKANVEFMDEALIMASMEHPHLVRLLGVCLSPTIQLVTQLMPHGCLLDYVH

EHKDNIGSQLLLNWCVQIAKGGDDTMENPP

>Takifugu rubripes ERBB4

VCAGTENKLSTLSDLDQQYQTMRKNYENCEVVMGNLEITSIDRSRNLTFLRSIREVTGYV

LVALNQFDYLPLENLRIIRGTKLYEDRYALAIFLNYRRDGNSGLRQLGLKNLTEILNGGV

YVDRNNFLCHADTIHWQDIVKHPRIHPLVVPTNSSITCQKCHRSCNGRCWGPKVDQCQSL

TKTVCAEQCDGRCFGPYISDCCHRECAGGCYGPKDTDCFACTNFNDSGACVTQCPQPFFY

NSITFQLEHNVRTKYTYGAFCVKKCPHNFVVDHSSCVRACPSNKMEVEENGIKICIPCTD

ICPKACDGIGTASLQTAQTVDSSNIDKFVNCTKINGNLVFLITGIKGDSYHNIEALDPEK

LNVFRTVREITGESHERFLNIQSWPDNMTDLSVFSNLATIGGRTLYSGISLLVLKQQGIS

SLQLQSLREISAGNVHVADNSQLCYYNTINWTRLFRTAHQKALIRNNQSPEQCSKKRMVC

DPLCSGAGCWGPGPDQCLSCKYFSRGRTCVDGCNLYEGEIREYANESVCVECDGQCELAD

DDSLTCHGPGPEHCEKCLHFKDGPNCVEKCPDGLQGANSFIFKYAEANNECHPCHANCTQ

GTPLIAAGVIGVLFMVVILVLSVAVSVRRKNIKKKRALRRFLETELVEPLTPSGTAPNQA

QLRILKETELKRVKVLGSGAFGTVYKGIWVPEGEKVKIPVAVKILNEATGPKANVEFMDE

ALIMASMEHPHLVRLLGVCLSPTIQLVTQLMPHGCLLDYVHEHKDNIGSQLLLNWCVQIA

KGMMYLEERRLVHRDLAARNVLVKSPNHIKITDFGLARLLDVNEKEYNADGGKMAIKWMA

LECIHYRKFTHQSDVWSYGVTIWELMTFGGKPYDGISTRDIPDLLEKGERLPQPPICTID

VYMVMVKCWMIDADSRPKFKELAAEFTRMARDPQRYLVIQGDDCMKLPGPNDSKFFQSLL

AEEELEDLMDAEEYLVPHSFNIPPLAYTPRTRMDSNKVFFFHIPCFINTLQILKTSGYLN

LCGSHAGMASKVPLFAFQKGGGDDSSGQRYSADPTGLLSERGAKGDQDGYMAAMRDKKIS

DYLNPVEENPFVTRRRNGDAHAVMDGAGCHTLHIAAAAAAPKNQHIHGDDEYINEPLYLN

TFLNPGDKNGLTASNTIMKNKKANFDNPEYWQHSLPPKSSLHNPEYLQDCSTRFFYRQNG

RIRPAVAENQEYLSEAAMKAGNVLPPPPYRQRNTVV

>Danio rerio ERBB4

VCPGTDNKLSTLSDLDQQYKTLRKFYENCEVVMGNLEITSIERNRNLSFLKSIREVTGYV

LVALNQFDYLPLENLRIIRGTKLYEGRYSLAIFLNYRRDGYFGLRQLGLKNLTEILNGGV

YVDQNKFLCHADTIHWHDIIKNPRSELLVVPSNNSGNTCRRCHRSCNGRCWGHQEDQCQS

LTKTVCAEQCDGRCFGPYVSNCCHRECAGGCFGPKDTDCFACTNFNDSGACVTQCPQPFV

YNPTTFQLEHNPNAKYTYGAFCVKKCPHNFVVDHSSCVRACPSNKMEVEENQTKMCIPCT

DICPKVCDGIGTGSLQMAQTVDSSNIEKFVNCTKINGNLIFLITGIKGDMFHGIRALDPD

RLNVFRTVREITGFLNVQSWPENMTDLGVFSNLATIGGRSLYSGISLLILKQRWITSLQF

QSLREISAGNVYMTNNSQLCFYSTVNWTSLFRTSTQRALIKNNREPRECSKEQKICDPLC

SEAGCWGPGPDQCLSCKYFSRGKTCVKSCNLYYGDVREFANGSVCVECDSQCEKAGEKGL

TCHGPGPDHCVKCLHLKDGPNCVEKCPDGLQGANSFIFKYAETNNECHPCHPNCTQGCTG

PRIQDCVGILDRTPLIAAGLIGGLFVIVIIALGVAVYIRRKSIKKKRALRRFLETEQLVE

PLTPSGAAPNQAQLRILKETELKRVKILGAGAFGMVYKGIWVPEGETVKIPVAIKILNES

TGPKANVEFMDEALIMASMEHPHLVRLLGVCLSPTIQLVTQLMPHGCLLDYVHEHQDNIG

SQLLLNWCVQIAKLIKYKAHVKVIISARAVFFKVLKHLQCYTFCNKKILKIQLYYYYKLI

VKIPLKKCCLYAVYYYSGVTIWELMTFGGKPYDGIPTRDIPDILEKGERLPQPPICTIDV

YMVMVKCWMIDADSRPRFKELAAEFSRMARDPQRYLVIQGDDRMKLPSPNHSTFFKSLLD

EEELDDLMDADEYLVPQ

>Homo sapiens ERBB3

MRANDALQVLGLLFSLARGSEVGNSQAVCPGTLNGLSVTGDAENQYQTLYKLYERCEVVM

GNLEIVLTGHNADLSFLQWIREVTGYVLVAMNEFSTLPLPNLRVVRGTQVYDGKFAIFVM

LNYNTNSSHALRQLRLTQLTEILSGGVYIEKNDKLCHMDTIDWRDIVRDRDAEIVVKDNG

RSCPPCHEVCKGRCWGPGSEDCQTLTKTICAPQCNGHCFGPNPNQCCHDECAGGCSGPQD

TDCFACRHFNDSGACVPRCPQPLVYNKLTFQLEPNPHTKYQYGGVCVASCPHNFVVDQTS

CVRACPPDKMEVDKNGLKMCEPCGGLCPKACEGTGSGSRFQTVDSSNIDGFVNCTKILGN

LDFLITGLNGDPWHKIPALDPEKLNVFRTVREITGYLNIQSWPPHMHNFSVFSNLTTIGG

RSLYNRGFSLLIMKNLNVTSLGFRSLKEISAGRIYISANRQLCYHHSLNWTKVLRGPTEE

RLDIKHNRPRRDCVAEGKVCDPLCSSGGCWGPGPGQCLSCRNYSRGGVCVTHCNFLNGEP

REFAHEAECFSCHPECQPMEGTATCNGSGSDTCAQCAHFRDGPHCVSSCPHGVLGAKGPI

YKYPDVQNECRPCHENCTQGCKGPELQDCLGQTLVLIGKTHLTMALTVIAGLVVIFMMLG

GTFLYWRGRRIQNKRAMRRYLERGESIEPLDPSEKANKVLARIFKETELRKLKVLGSGVF

GTVHKGVWIPEGESIKIPVCIKVIEDKSGRQSFQAVTDHMLAIGSLDHAHIVRLLGLCPG

SSLQLVTQYLPLGSLLDHVRQHRGALGPQLLLNWGVQIAKGMYYLEEHGMVHRNLAARNV

LLKSPSQVQVADFGVADLLPPDDKQLLYSEAKTPIKWMALESIHFGKYTHQSDVWSYGVT

VWELMTFGAEPYAGLRLAEVPDLLEKGERLAQPQICTIDVYMVMVKCWMIDENIRPTFKE

LANEFTRMARDPPRYLVIKRESGPGIAPGPEPHGLTNKKLEEVELEPELDLDLDLEAEED

NLATTTLGSALSLPVGTLNRPRGSQSLLSPSSGYMPMNQGNLGESCQESAVSGSSERCPR

PVSLHPMPRGCLASESSEGHVTGSEAELQEKVSMCRSRSRSRSPRPRGDSAYHSQRHSLL

TPVTPLSPPGLEEEDVNGYVMPDTHLKGTPSSREGTLSSVGLSSVLGTEEEDEDEEYEYM

NRRRRHSPPHPPRPSSLEELGYEYMDVGSDLSASLGSTQSCPLHPVPIMPTAGTTPDEDY

EYMNRQRDGGGPGGDYAAMGACPASEQGYEEMRAFQGPGHQAPHVHYARLKTLRSLEATD

SAFDNPDYWHSRLFPKANAQRT

>Mus musculus ERBB3

MSAIGTLQVLGFLLSLARGSEMGNSQAVCPGTLNGLSVTGDADNQYQTLYKLYEKCEVVM

GNLEIVLTGHNADLSFLQWIREVTGYVLVAMNEFSVLPLPNLRVVRGTQVYDGKFAIFVM

LNYNTNSSHALRQLRFTQLTEILLGGVYIEKNDKLCHMDTIDWRDIVRVPDAEIVVKNNG

GNSSQSGPPCHEVCKGRCWGPGPEDCQILTKTICAPQCNGRCFGPNPNQCCHDECAGGCS

GPQDTDCFACRHFNDSGACVPRCPAPLVYNKLTFQLEPNPHIKYQYGGVCVASCPHNFVV

DQTFCVRACPADKMEVDKNGLKMCEPCRGLCPKACEGTGSGSRYQTVDSSNIDGFVNCTK

ILGNLDFLITGLNGDPWHKIPALDPEKLNVFRTVREITGYLNIQSWPPHMHNFSVFSNLT

TIGGRSLYNRGFSLLIMKNLNVTSLGFRSLKEISAGRVYISANQQLCYHHSLNWTRLLRG

PAEERLDIKYNRPLGECVAEGKVCDPLCSSGGCWGPGPGQCLSCRNYSREGVCVTHCNVL

QGEPREFVHEAHCFSCHPECQPMEGTSTCNGSGSDACARCAHFRDGPHCVNSCPHGILGA

KGPIYKYPDAQNECRPCHENCTQGCKGPELQDCLGQAEVLMSKPHLVIAVTVGLTVIFLI

LGGSFLYWRGRRIQNKRAMRRYLERGESIEPLDPSEKANKVLARIFKETELRKLKVLGSG

VFGTVHKGIWIPEGESIKIPVCIKVIEDKSGRQSFQAVTDHMLAVGSLDHAHIVRLLGLC

PGSSLQLVTQYLPLGSLLDHVRQHRETLGPQLLLNWGVQIAKGMYYLEEHSMVHRDLALR

NVMLKSPSQVQVADFGVADLLPPDDKQLLHSEAKTPIKWMALESIHFGKYTHQSDVWSYG

VTVWELMTFGAEPYAGLRLAEIPDLLEKGERLAQPQICTIDVYMVMVKCWMIDENIRPTF

KELANEFTRMARDPPRYLVIKRASGPGIPPAAEPSALSTKELQDAELEPDLDLDLDVEVE

EEGLATTLGSALSLPTGTLTRPRGSQSLLSPSSGYMPMNQSNLGEACLDSAVLGGREQFS

RPISLHPIPRGRQTSESSEGHVTGSEAELQERVSMCRSRSRSRSPRPRGDSAYHSQRHSL

LTPVTPLSPPGLEEEDGNGYVMPDTHLRGTSSSREGTLSSVGLSSVLGTEEEDEDEEYEY

MNRKRRGSPARPPRPGSLEELGYEYMDVGSDLSASLGSTQSCPLHPMAIVPSAGTTPDED

YEYMNRRRGAGGSGGDYAAMGACPAAEQGYEEMRAFQGPGHQAPHVRYARLKTLRSLEAT

DSAFDNPDYWHSRLFPKANAQRI

>Rattus norvegicus ERBB3

MRATGTLQVLCFLLSLARGSEMGNSQAVCPGTLNGLSVTGDADNQYQTLYKLYEKCEVVM

GNLEIVLTGHNADLSFLQWIREVTGYVLVAMNEFSVLPLPNLRVVRGTQVYDGKFAIFVM

LNYNTNSSHALRQLKFTQLTEILSGGVYIEKNDKLCHMDTIDWRDIVRVRGAEIVVKNNG

ANCPPCHEVCKGRCWGPGPDDCQILTKTICAPQCNGRCFGPNPNQCCHDECAGGCSGPQD

TDCFACRRFNDSGACVPRCPEPLVYNKLTFQLEPNPHTKYQYGGVCVASCPHNFVVDQTF

CVRACPPDKMEVDKHGLKMCEPCGGLCPKACEGTGSGSRYQTVDSSNIDGFVNCTKILGN

LDFLITGLNGDPWHKIPALDPEKLNVFRTVREITGYLNIQSWPPHMHNFSVFSNLTTIGG

RSLYNRGFSLLIMKNLNVTSLGFRSLKEISAGRVYISANQQLCYHHSLNWTRLLRGPSEE

RLDIKYNRPLGECLAEGKVCDPLCSSGGCWGPGPGQCLSCRNYSREGVCVTHCNFLQGEP

REFVHEAQCFSCHPECLPMEGTSTCNGSGSDACARCAHFRDGPHCVNSCPHGILGAKGPI

YKYPDAQNECRPCHENCTQGCNGPELQDCLGQAEVLMSKPHLVIAVTVGLAVILMILGGS

FLYWRGRRIQNKRAMRRYLERGESIEPLDPSEKANKVLARIFKETELRKLKVLGSGVFGT

VHKGIWIPEGESIKIPVCIKVIEDKSGRQSFQAVTDHMLAVGSLDHAHIVRLLGLCPGSS

LQLVTQYLPLGSLLDHVKQHRETLGPQLLLNWGVQIAKGMYYLEEHSMVHRDLALRNVML

KSPSQVQVADFGVADLLPPDDKQLLHSEAKTPIKWMALESIHFGKYTHQSDVWSYGVTVW

ELMTFGAEPYAGLRLAEIPDLLEKGERLAQPQICTIDVYMVMVKCWMIDENIRPTFKELA

NEFTRMARDPPRYLVIKRASGPGTPPAAEPSVLTTKELQEAELEPELDLDLDLEAEEEGL

ATSLGSALSLPTGTLTRPRGSQSLLSPSSGYMPMNQSSLGEACLDSAVLGGREQFSRPIS

LHPIPRGRPASESSEGHVTGSEAELQEKVSVCRSRSRSRSPRPRGDSAYHSQRHSLLTPV

TPLSPPGLEEEDGNGYVMPDTHLRGASSSREGTLSSVGLSSVLGTEEEDEDEEYEYMNRK

RRGSPPRPPRPGSLEELGYEYMDVGSDLSASLGSTQSCPLHPMAIVPSAGTTPDEDYEYM

NRRRGAGGAGGDYAAMGACPAAEQGYEEMRAFQGPGHHAPHVRYARLKTLRSLEATDSAF

DNPDYWHSRLFPKANAQRT

>Xenopus tropicalis ERBB3

VCSGTMNGLSVSGDSDYQLFTLKNMYSNCQIVMGNLEIVLIDNKGDLSFLTSIREVTGYV

LIAVNIFTYLPLENLRVIRGAHLYDKKYALYMSLNNQINGSLGIEKLGFHKLTEILAGGV

HITKNDHLCFTDTINWKDIVQDSKAAIEIESNSKACKPCDQACDGNCWGPEKTDCQTLTK

TVCAEQCNGRCFGKNPTECCHDECAGGCTDSSNLKCFVCRHFNDSEACVPHCPQPLIYNK

LTFQLEPNPDAKYQYGGSCVKSCPRNFVVDQSSCVRACPSNKMEVEKNGVKMCEPCSGLC

PKACEGTLHGSTYQTVDSSNIDKFINCTKILGNLDFLTIGIDGDLWRNITALDPKKLSVF

KTVKEITGYLNIQSWPKSLTDFSVFSSLSTIGGRTLHTRGFSLLVMKNSNVTSLGFRSLQ

EISAGKVYIAENQNLCYCDTVNWTSLFPTYPRQSTEIKGNRKKNKCVEEGKVCDPLCSNN

GCWGSGPDQCLSCRNYSRNGVCLQSCNFTEGDEREYAKAGVCLKCHEECQKMEGRLSCNG

SGPDGCVQCVNYRDGLHCVSQCPNGILGKDRPIFKYPDANKDCQPCHENCTHGCTGPEIK

HCTGIAAPIISSNTPTVVVAAVVATFFVLCCCALLGTLYWRGQKIRNKRAMRRYLERGEC

LDMLDPSEKTNTVQARLFKDTELKKLKILGSGVFGTVHKGVWIPDGESIKIPVCIKVILD

RTGRQTFHAVTDHMLAIGSLDHTYIVRLLGICPGSQLQLVTAYLPMGSLLQHVRKNKDSG

AIGPQLLLNWCVQIAKGMCYLEEHRMVHRNLSARNVLMKSVNQVQVSDYGVADLLYPDDK

KFCYNEIKTPVKWMALESIHFGKYTHQSDVWSYVPQKGVTLWEMMTFGAEPYTGIRLSEV

PDLLEKGERLSQPQICTIDVYMVMVKCWMIDENIRPTFKELGNEFTRMARDPPRYLVVTR

NSDALQSPLPGDPPPLSEKQVSDLESDVDTDLESEDMAMQPKTPTTLYASRQRIDYSRSQ

SALSSIAGYLPMNQSLCSSSSCYGLPLLGKGTMTSAEPIPRHGTRRREDSLGRTVSESSE

GCCTSSDLETIEDNSYGGGSLSHISRSRLDSAYRSQRESLLVGTPDDDSEYIMPGLSQSV

TEISRDGTLTSQGSRSLTPCLVNATDEEDEEYEYMNKKKPSSKEKLEYEYMDIRGTGDAS

NECIAFSPAEGDKEGDYEYGNKEDYEYMNEKGKYTNMNLVANLPQQLERPTVIPQTCHRE

ALQKGYHQPLRALEAKDCAFDNPDYWHSRLFSKTDLQMT

>Takifugu rubripes ERBB3

VCSGTQNGLSTTGTSEIQYNLTKKIYTNCEIVMGNLEITMMEHSRDFSFLQSIREVTGYI

LLAINEFSRLPLDNLRVIRGNVLYENQYALAIMINYRKDGEHGLQELGLTHLTEILQGGV

KIIKNRDLSYAPKVNWLDIVKDGAANITIFGNGPEKPCHEACGDVPCWGPGRNMCQIMTK

TVCAPQCNFRCFGRSPSECCHSECAGGCTGPLDTDCFACRRFNDSGSCVPQCPMALIYNK

YTFRLEPNPNAKFQYGSICVAQCPPNFLVDGTSCVSSCPADKTEVEKKGVKRCEPCEGLC

PKACHGTGSGTRETVDALNIDSFINCTKIQGSLHFLVTGIEGDKYKNVPPLDPEKLKIFN

TVRQITDILSVQSWPTNMSDLSVFSNLQIIEGRKLYNSYSLLVVKNPSLTSLGLRSLREI

NDGAVYITGNKRLCYHDTVNWTRLFNPHLPSQQKIEIKDNRPRSECGRLLGPGAKPVYVL

QEVQQRRDLCAGLHVLRWGADECVECANLQDGPHCISSCPQGVMGGEEIIFKYPNKQGRC

EPCHANCTKGSAIRLQMVTGIVVGVIALLFVSFAVFVLTVLYRRGLAIRRKRAMRRYMER

GESFEPLDHGEKGTKVHARILKPSELRTVKLLGNGVFGSVYKGIWIPEGETVKLPVAIKV

IHDRTGQKTFNKPTDHMIVLGSLDHMNIVRILGICPGDSLQLISQLSSHGSLLEHVRNCK

NKLSPQRLLNWCVQIAKGMYYLEENKVVHRNLAARNVLLKNNYTAQVSDYGIADLLHRDD

KKYFFNEAKAPIKWMALESILFRKYTHQSDVWSYGVTVWEMMSYGAEPYNTMRPQDVSDL

LEKGERLSQPQICTIDVYMVMVKCWMVDENVRPTFKELANEFTRMARDPPRYLVIKDTDE

HVQRSADVLDADDLDVLEVVDTEMMNQEKEDEECGLAPAPYYVPQNRTLSRLSKMDTYKV

VLSSSNVAGYLPMTPGVDNVTVWASRSRLNSGRTMSESSEGCGTVVDMERSEDVSLSGSL

KRHRNRQDSAYMSQRDSMSCGPPETPSPDMEEEDQNGYVLPGDSPKRDTLLSTSRGIPGR

IGKSHSSSLLNDPDDEEYEYMNKQACNSVSPRHNGHWMKMNKKRTSSTSSQMTACSVDTI

PSMELRRSQTMSSYNSDLEQPGSNDVKYEYMDIRSIDKEDKGKDNGQLYEYEDMDSITSA

QPEDAAVYQNIQKEGDSAARQGPAQTPGFDAYVRVRAGVGLGEPAAVDRSFDNPNYWHSR

MFLKPNAVPT

>Danio rerio ERBB3

RGYSLLVMKIPTLKSLGLRSLKRISDGGIYITGNTQLCYHHTVNWTRLFGSGPRALRRQK

SLDIKENRPQEQCRMLGSGTRSVFVLQELQKRRNMCTRLQLHVRGSDECKACAHLQDGPY

CVSSCPEGVQGENGLIIYKFPNRQNKCQPCHANCTLGSAHYGVNIVRYRLPVTAIVLSVI

FCVLLAFSVFVLSVLYRRSLSIRRKRAMRRYLQSGESFEPLEPGEKGVKVHARILRTSEL

KKIKLLGSGVFGTVHKGIWIPEGDTVKIPVAIKTIQDRTGRQTFQEITDHMLAMGSLDHA

YIVRILGICPGASLQLVTQLSPQGSLLDHIRQRRDNLNPQRLLNWCVQIAKGMYYLEEQR

MVHRNLAARNILLKSDLIVQIADYGIADLLYPDEKKYFFNEIKTPIKWMALESILFRRYT

HQSDVWSYGVTVWEMMSYGAEPYSAMRPQDVPDLLEKGERLSQPQICTIDVYMVMVKCWM

IDENVRPTFKELANEFTRMARDPHRYLVIKEEDRAPDSASDETHQNGTEIDILGVALEDQ

DDEVLEDAIDAPRYITPSRSFSRLRIDSHRSSLTPSTTAGYLPMTPGLESSVQTGWASRS

RLDSACTVSESSEGRGALELEMNDDFSSVGSLRRVRHREDSAYMSQRDSLSGPPETTSTG

TQGEEDQNEYVLPGFGESPEKETLLFTSSRSTLSQKRLSRGHSGDFLEANRGAGEEYEYM

NNQTLSLSHIPGQLKEYNQRAPRNRSASFNPGGMYSNHKPSRPSKRSSIEGSESSGGLSQ

SSTDQRSHSDGDFLSSEAEYTDGDLEGGGGEAYEYEDMDSLAAGGASGAEYQNLDGEDEE

RDEDWSGGPRSPGPYVQVHAGTNHNAFDNPDYWHSRSFHKTKAVRT

>Homo sapiens ERBB2

MELAALCRWGLLLALLPPGAASTQVCTGTDMKLRLPASPETHLDMLRHLYQGCQVVQGNL

ELTYLPTNASLSFLQDIQEVQGYVLIAHNQVRQVPLQRLRIVRGTQLFEDNYALAVLDNG

DPLNNTTPVTGASPGGLRELQLRSLTEILKGGVLIQRNPQLCYQDTILWKDIFHKNNQLA

LTLIDTNRSRACHPCSPMCKGSRCWGESSEDCQSLTRTVCAGGCARCKGPLPTDCCHEQC

AAGCTGPKHSDCLACLHFNHSGICELHCPALVTYNTDTFESMPNPEGRYTFGASCVTACP

YNYLSTDVGSCTLVCPLHNQEVTAEDGTQRCEKCSKPCARVCYGLGMEHLREVRAVTSAN

IQEFAGCKKIFGSLAFLPESFDGDPASNTAPLQPEQLQVFETLEEITGYLYISAWPDSLP

DLSVFQNLQVIRGRILHNGAYSLTLQGLGISWLGLRSLRELGSGLALIHHNTHLCFVHTV

PWDQLFRNPHQALLHTANRPEDECVGEGLACHQLCARGHCWGPGPTQCVNCSQFLRGQEC

VEECRVLQGLPREYVNARHCLPCHPECQPQNGSVTCFGPEADQCVACAHYKDPPFCVARC

PSGVKPDLSYMPIWKFPDEEGACQPCPINCTHSCVDLDDKGCPAEQRASPLTSIISAVVG

ILLVVVLGVVFGILIKRRQQKIRKYTMRRLLQETELVEPLTPSGAMPNQAQMRILKETEL

RKVKVLGSGAFGTVYKGIWIPDGENVKIPVAIKVLRENTSPKANKEILDEAYVMAGVGSP

YVSRLLGICLTSTVQLVTQLMPYGCLLDHVRENRGRLGSQDLLNWCMQIAKGMSYLEDVR

LVHRDLAARNVLVKSPNHVKITDFGLARLLDIDETEYHADGGKVPIKWMALESILRRRFT

HQSDVWSYGVTVWELMTFGAKPYDGIPAREIPDLLEKGERLPQPPICTIDVYMIMVKCWM

IDSECRPRFRELVSEFSRMARDPQRFVVIQNEDLGPASPLDSTFYRSLLEDDDMGDLVDA

EEYLVPQQGFFCPDPAPGAGGMVHHRHRSSSTRSGGGDLTLGLEPSEEEAPRSPLAPSEG

AGSDVFDGDLGMGAAKGLQSLPTHDPSPLQRYSEDPTVPLPSETDGYVAPLTCSPQPEYV

NQPDVRPQPPSPREGPLPAARPAGATLERPKTLSPGKNGVVKDVFAFGGAVENPEYLTPQ

GGAAPQPHPPPAFSPAFDNLYYWDQDPPERGAPPSTFKGTPTAENPEYLGLDVPV

>Mus musculus ERBB2

DCPPLGTRSPGAPLPRRPLPAPRLCLPSPRPAPAAWSSLSHGAGAAVIIMELAAWCRWGF

LLALLSPGAAGTQVCTGTDMKLRLPASPETHLDMLRHLYQGCQVVQGNLELTYLPANASL

SFLQDIQEVQGYMLIAHNRVKHVPLQRLRIVRGTQLFEDKYALAVLDNRDPLDNVTTAAP

GRTPEGLRELQLRSLTEILKGGVLIRGNPQLCYQDMVLWKDVLRKNNQLAPVDMDTNRSR

ACPPCAPTCKDNHCWGESPEDCQILTGTICTSGCARCKGRLPTDCCHEQCAAGCTGPKHS

DCLACLHFNHSGICELHCPALITYNTDTFESMLNPEGRYTFGASCVTTCPYNYLSTEVGS

CTLVCPPNNQEVTAEDGTQRCEKCSKPCAGVCYGLGMEHLRGARAITSDNIQEFAGCKKI

FGSLAFLPESFDGNPSSGVAPLKPEHLQVFETLEEITGYLYISAWPESFQDLSVFQNLRV

IRGRILHDGAYSLTLQGLGIHSLGLRSLRELGSGLALIHRNTHLCFVNTVPWDQLFRNPH

QALLHSGNRPEEACGLEGLVCNSLCARGHCWGPGPTQCVNCSQFLRGQECVEECRVWKGL

PREYVRGKHCLPCHPECQPQNSSETCYGSEADQCEACAHYKDSSSCVARCPSGVKPDLSY

MPIWKYPDEEGICQPCPINCTHSCVDLDERGCPAEQRASPVTFIIATVVGVLLFLIIVVV

IGILIKRRRQKIRKYTMRRLLQETELVEPLTPSGAVPNQAQMRILKETELRKLKVLGSGA

FGTVYKGIWIPDGENVKIPVAIKVLRENTSPKANKEILDEAYVMAGVGSPYVSRLLGICL

TSTVQLVTQLMPYGCLLDHVREHRGRLGSQDLLNWCVQIAKGMSYLEEVRLVHRDLAARN

VLVKSPNHVKITDFGLARLLDIDETEYHADGGKVPIKWMALESILRRRFTHQSDVWSYGV

TVWELMTFGAKPYDGIPAREIPDLLEKGERLPQPPICTIDVYMIMVKCWMIDSECRPRFR

ELVSEFSRMARDPQRFVVIQNEDLGPSSPMDSTFYRSLLEDDDMGELVDAEEYLVPQQGF

FSPDPALGTGSTAHRRHRSSSARSGGGELTLGLEPSEEEPPRSPLAPSEGAGSDVFDGDL

AVGVTKGLQSLSPHDLSPLQRYSEDPTLPLPPETDGYVAPLACSPQPEYVNQPEVRPQSP

LTPEGPPPPIRPAGATLERPKTLSPGKNGVVKDVFAFGGAVENPEYLAPRAGTASQPHPS

PAFSPAFDNLYYWDQNSSEQGPPPSTFEGTPTAENPEYLGLDVPV

>Rattus norvegicus ERBB2

MIIMELAAWCRWGFLLALLPPGIAGTQVCTGTDMKLRLPASPETHLDMLRHLYQGCQVVQ

GNLELTYVPANASLSFLQDIQEVQGYMLIAHNQVKRVPLQRLRIVRGTQLFEDKYALAVL

DNRDPQDNVAASTPGRTPEGLRELQLRSLTEILKGGVLIRGNPQLCYQDMVLWKDVFRKN

NQLAPVDIDTNRSRACPPCAPACKDNHCWGESPEDCQILTGTICTSGCARCKGRLPTDCC

HEQCAAGCTGPKHSDCLACLHFNHSGICELHCPALVTYNTDTFESMHNPEGRYTFGASCV

TTCPYNYLSTEVGSCTLVCPPNNQEVTAEDGTQRCEKCSKPCARVCYGLGMEHLRGARAI

TSDNVQEFDGCKKIFGSLAFLPESFDGDPSSGIAPLRPEQLQVFETLEEITGYLYISAWP

DSLRDLSVFQNLRIIRGRILHDGAYSLTLQGLGIHSLGLRSLRELGSGLALIHRNAHLCF

VHTVPWDQLFRNPHQALLHSGNRPEEDCAVSSGLVCNSLCAHGHCWGPGPTQCVNCSHFL

RGQECVEECRVWKGLPREYVSDKRCLPCHPECQPQNSSETCFGSEADQCAACAHYKDSSS

CVARCPSGVKPDLSYMPIWKYPDEEGICQPCPINCTHSCVDLDERGCPAEQRASPVTFII

ATVVGVLLFLILVVVVGILIKRRRQKIRKYTMRRLLQETELVEPLTPSGAMPNQAQMRIL

KETELRKVKVLGSGAFGTVYKGIWIPDGENVKIPVAIKVLRENTSPKANKEILDEAYVMA

GVGSPYVSRLLGICLTSTVQLVTQLMPYGCLLDHVREHRGRLGSQDLLNWCVQIAKGMSY

LEDVRLVHRDLAARNVLVKSPNHVKITDFGLARLLDIDETEYHADGGKVPIKWMALESIL

RRRFTHQSDVWSYGVTVWELMTFGAKPYDGIPAREIPDLLEKGERLPQPPICTIDVYMIM

VKCWMIDSECRPRFRELVSEFSRMARDPQRFVVIQNEDLGPSSPMDSTFYRSLLEDDDMG

DLVDAEEYLVPQQGFFSPDPTPGTGSTAHRRHRSSSTRSGGGELTLGLEPSEEGPPRSPL

APSEGAGSDVFDGDLAMGVTKGLQSLSPHDLSPLQRYSEDPTLPLPPETDGYVAPLACSP

QPEYVNQSEVQPQPPLTPEGPLPPVRPAGATLERPKTLSPGKNGVVKDVFAFGGAVENPE

YLVPREGTASPPHPSPAFSPAFDNLYYWDQNSSEQGPPPSNFEGTPTAENPEYLGLDVPV

>Xenopus tropicalis ERBB2

WGLCALLLLLRGRAAEEVMGEVCTGTDMKLLHPFSQRNHYETLSTIYRDCQVIQGNLEIT

YLKEDNDVSFLKDIKEVQGYVLIAHNLLSYVPLENLRIIRGTQLYQERYALAVLSNSDPS

GTAGLRELRMKNLTEILKGGVIMENNTQLCFQDTVLWADILSQSNSQRNEVVLGSSNRQC

PTCSPGCGSLPSGKSGFCWGEGQEYCQILTSTICNSGCQRCKGNHSTHCCHEQCAAGCTG

PKNSDCLACLHFNHNGVCGLHCPPLRTYNPETYESVHNEGGRYTFGASCVTSCPYNYLAT

EVGSCTLFCPQDNQEVIVGNVQKCEKCDKQCSKVCYGLGRDFLRTTQAVNASIIHHFQGC

TKIFGSLVFLSESFKPDPNKPNSVLNAQSLEVFKTLEEITGYIYIESWPEELKSLNVFEN

LRVIHGRMLQNGAYSLVLRNLSISHLGLRSLTEVSSGLVLIEGNPDLCFLESAPWASLFR

NPRQTILKTINKPQEACDQEGQNCFSRCDGNKCWGPGPSQCVRCNGFLRGHDCIESCNVP

LRYPREHVNGSQCVPCHPQCLPQNGTLTCNGTEAHQCMECAHYLDGEDCVEQCPSGVKGN

SFVPIWKYPDKDRICQLCNVNCSHSCNQLDERGCPILPDNSQVYMVASIVISILIVITIS

TAIVLYIRRRKQLKKKHTMRRLLQDELVEPLTPSGAIPNQAQMRILKETELKKMMILGSG

AFGTVYKGIWIPDGENIKIPVAIKVLRENTSPKANKEILDEAYVMAGVGSPYVCRLLGIC

LTSTVQLVTQLMPYGCLLDYVRENKDRIASRDLLNWCVQIAKGMTYLEDVRLVHRDLAAR

NVLVKSPSHVKITDFGLARLLDVDETEYHADGGKVPIKWMALESILHRRFTHQSDVWSYG

VTVWELMTFGAKPYDGIPAREIPDLLEKGERLPQPPVCTIDVYMIMVKCWMIDSEFRPKF

RDLVGELSRMARDPSRYVVIENEEFLAQASPMTTQFYSALLQEVGMDDLVDAEEYLVPQR

GFFPSEDVTSEHRSRISSTRVTDMLTTFSPFPPQSATEMQAEQALLQETAPSGAALARTM

SERSGGSGSDALSEGEYVFDPPQNEESGSPHRYTDDPLHSGEDETDRRLHFPKHSPTPTS

THTVYRQKSTLERQKSTLERQKAQQCKNGLVRELRSPPALSSLDNPDYLPPPGLILPNGF

PQAFDNPYYWNHELNLAKADGDPAQGHNGFTTPTAENPEYLGLDETMIRPRDIT

>Tetraodon nigroviridis ERBB2

PCPLSVAACMGTDMKLSLPSSLENHHEMLRLLYTGCQVVHGNLEITHLHGTPDLSFLQGI

VEVQGYVLVAHVSVSLLPLDNLRIIRGSQLYNSSYALAVLDNTLDGRGLRTLHLRSLTEI

LFGSVYVWGNPQLCFPDPQSINWRDILDQQNTDTRHRLQPRAQNCPNCSSQCLTSCWGEA

AQDCQTLTRSNCASGCQRCKGFLPNDCCHKQCAAGCTGPKDSDCLACRHFNDSGVCKENC

PPPTTYDPATFQSKPNPDRKFSFGATCVKTCPYNYLAMDVACTLNYPKPNPKDVVPQPCG

NDTQKCEKCEGDCPKVCYGPGMDDHGAVDSSGSTMVTAANVGQFNKCTKIFGSLAFHSQS

FSRDPVTNASGLSPEQLSVFRNLEEITGYLYIEAWPQEWMNLNVFDNLKVIRGRMLYKGV

FSLAIQNLQITSLGLRSLRSISGGLVLLHNNSQLCYTSSLPWESIVHPTQGPHRIVSKNL

DPQICEKQGHVCHRLCKGGCWGPGPGQCASCETFQRGTECVEHCDIYQGSVREYASESLC

VACHPECQPLNGSASCHGPGAEHCVECRKFQDGDFCVDHCPSGVKEDHQTVWKYSNATGH

CLPCNTNCTLSCTVKDERGCPVDTRAGLGTTIAAAVGGVVLFFILLGLLFFYLRRQKKLK

RKETMARILQEHELVEPLAPSGASPNQAQMRILKETELKKLKVLGAGAFGTVYKGVWAPD

GENVKIPVAIKVLRENTSPKANKEILDPLRVPSAGDLLDLHRAVVTQLMSYGCLLEYVRK

NKDHIGSQLLLNWCVQIAKVWNPEAGFVSHYVASLFFLSHLMLAGILWTQMWCVCVCVCV

CVGNELPGGSSSGAQRSSSPQCPGKESKPREDHGLRSGPSAGYRRDGISRRRGEGEMEDI

CTEDLPLINDVQSFCSFLRCRSNGWPWSQFCKGSSLIRVMCGATVCFKSLNVTIITCSNS

KMNQTPVKLLKNGISNSHLHSLQRDQTVCTSFTGVTVWELMTFGAKPYDMIPARDIPEVL

EGGERLPQPPICTIDVYMIMVKSRLADIWTFCLLAGWMIDPDYRPRFKDLVKEFTTMARD

PPRYVVIQSRDQGLDVEPSAAGGPGSMYSSLSTLSRSQYPTLPLGASAANGVWSPPYPML

ARSPSAGEQSDSVFLESPDDPSLPPASPNRYCRDPTYSNDNQGDLETDGPGFHLPQHSHH

SLPRQSHRNHVLPEYVNQEIQHPRPGILERPTTLPRKGSRADRRLPNGLSSGHSVENPGY

LIPFTARTPTSPAFDNPYYLDHLAKARTGAGASEVLDSEALEAGGAMPRHVNGFVTPTAE

NPEYLGLADTLSGHT

>Takifugu rubripes ERBB2

AACMGTDMKLSLPSSLENHYEMLRLLYTGCQVVHGNLEITHLHGNPDLSFLQGIVEVQGY

VLVARVSVSLVPLDNLRIIRGSQLYNSSYALAALDNTLDGQGLRTLALRSLTEILFGGVY

IWGNPQLCFPDPQSINWRDILDELNTDTRYRLQPRALNCPNCSSQCLTSCWGETVQDCQT

LTHSNCALGCQRCKGSLTNDCCHKQCSAGCTGPKDSDCLACRHFNDSGVCKENCPPPTIY

DPTMFQSKPNPDRKFSFGATCVKTCPYNYLAMDVACTLNYPKPNQEVIITQPCGNDTQKC

EKCEGDCPKACYGPGMDNSGVMDSHGNTMVNSSNVEQFNKCTKIFGSLAFYPQSFTRDPV

TNASGLSPQQLSVFRNLEEITGYLYIEAWPQEWMNLSVFDNLKVIRGRMLYKGVFSLAIQ

HLQITSLGLRSLRSISGGLVLLHNNSQLCYTSSLPWENIVHPTQGPHRIVSKNQDPQICE

KQGRVCHPLCEGGCWGPGPSQCASCRTFQRGSECVEQCDIYQGSVREYANESVCVACHPE

CQPLNSSASCHGPGAEHCAECRNFQDGDVCVAHCPSGAKEDHQTVWKYSNATGHCLPCNT

NCTLSCTVMDERGCPVDTRTGLGTTIAAAVGGVVLFFILLGLLFFYLRRQKKLKRKETMR

RILQEHELVEPLAPSGASPNQAQMRILKETELKKLKVLGAGAFGTVYKGVWAPDGENVKI

PVAIKVLRENTSPKANKEILDEAYVMAGVASPYVCRLLGICLTSTVQLVTQLMSYGCLLE

YVRKNKDRIGSQLLLNWCVQIAKGMSYLEEVRLVHRDLAARNVLVKNPNHVKITDFGLAR

LLDIDETEYHADGGKVPIKWMALESILHRRFTHQSDVWSYGVTVWELMTFGAKPYDMIPA

REIPEVLEGGERLPQPLICTIDVYMIMVKCWMIDPEYRPRFKDLVKEFTAMARDPPRYVV

IQNEEQMSMSSPVDNQFFRMFLAEEGNNLRELLDAEEYLVPQPNHFP

>Danio rerio ERBB2

MEADRSFGLAWVLLLLLGITAATGREVCLGTDMKLALPSSLENHYEMLRLLYTGCQVVHG

NLEITHLQGNPDLSFLQEIVEVQGYVLIAHVSVRSLPLDNLRIIRGSQLYKSNYALAVHN

NSNSSQAGLGLRELRLRSLTEILLGGVYIWGNPQLCFPRNINWEDTVSKVQNKPLHLQDI

PKNCPRCSSACKSGGCWGEKDQDCQTLTSVNCSSGCSRCKGPKPSDCCHVQCAAGCTGPK

DSDCLACRHFNDSGTCKDSCPPPTIYDPITFQSKPNKDKKFSFGATCVKQCPHNYLAMEV

ACTMVCPKANKEVISVEPDGQETQKCEKCEGECPKVCYGLGMGNLQGVSVVNSTNIGMFT

GCEKIYGSLAFLSDSFKGNTDTNSSGLQPSDLEKLKTIEEITGYLYIDAWSENLLDLSVF

ENLKVIRGRMLYKGVFSLGVQSLQIESLGLRSLRSVSGGLVLIHNNSRLCYTSSLPWTSL

LHPTQGPNLISNNNKDQQTCVSEGKICDPLCGDSGCWGPGPSQCVSCLNYKRGTECVELC

NVLHGSVREFEDGFNCVPCHPECRPINGTASCTGPGPDQCTDCMHFQDGDVCVERCPSGV

KEEQHTVWKYSNATGHCLPCETNCTVSCPLDDRGCPIQQKTGPGTTVAITVGGVLLFIIL

LALLVFYLRRQKHQKKKETIRRRLQEHELVEPLTPSGAMPNQAQMRILKETELKKLRVLG

SGAFGTVFKGIWAPDGENVRIPVAIKVLRENTSPKANKEILDEAYVMAGVASPYVCRLLG

ICLTSTVQLVTQLMPYGCLLDYVRENKDRIGSQYLLSWCVQIAKGMSYLEEVRLVHRDLA

ARNVLVKNPNHVKITDFGLARLLDIDEKEYHADGGKVPIKWMALESILHRKFTHQSDVWS

YGVTVWELMTFGMKPYESFQARDIPELLEAGERLSQPCNCTKEVYMIMVKCWQIDPDNRP

RFKDLVDEFTTMARDPSRYVVIQNEDQMSLSSPVDSEFFRILMAEEGGNVKEFLDAEEYL

VPQPGSIFNTHGEMRANGPSRHHSHRSTDQMVEVDGLPNGRELYSSVSMIEQSQYPTLPV

GATANGMWPGTQYPPLARSISHRSAGGQSDSVFLDGYVEDSCPPSSPCRYSKDPTMPNGT

DGDLETDGNMVFLSHTLPRGTHTQPEYVNQDMASERPSTLPRKASERRFILNGLSTGNSV

ENPEYLVPIGSITPTSPAFDNPYYHDIAAKAQAVARVAINGGTNHRQPNGFMTPTAENPE

YLGLADTWSGHKEYT

>Homo sapiens EGFR

MRPSGTAGAALLALLAALCPASRALEEKKVCQGTSNKLTQLGTFEDHFLSLQRMFNNCEV

VLGNLEITYVQRNYDLSFLKTIQEVAGYVLIALNTVERIPLENLQIIRGNMYYENSYALA

VLSNYDANKTGLKELPMRNLQEILHGAVRFSNNPALCNVESIQWRDIVSSDFLSNMSMDF

QNHLGSCQKCDPSCPNGSCWGAGEENCQKLTKIICAQQCSGRCRGKSPSDCCHNQCAAGC

TGPRESDCLVCRKFRDEATCKDTCPPLMLYNPTTYQMDVNPEGKYSFGATCVKKCPRNYV

VTDHGSCVRACGADSYEMEEDGVRKCKKCEGPCRKVCNGIGIGEFKDSLSINATNIKHFK

NCTSISGDLHILPVAFRGDSFTHTPPLDPQELDILKTVKEITGFLLIQAWPENRTDLHAF

ENLEIIRGRTKQHGQFSLAVVSLNITSLGLRSLKEISDGDVIISGNKNLCYANTINWKKL

FGTSGQKTKIISNRGENSCKATGQVCHALCSPEGCWGPEPRDCVSCRNVSRGRECVDKCN

LLEGEPREFVENSECIQCHPECLPQAMNITCTGRGPDNCIQCAHYIDGPHCVKTCPAGVM

GENNTLVWKYADAGHVCHLCHPNCTYGCTGPGLEGCPTNGPKIPSIATGMVGALLLLLVV

ALGIGLFMRRRHIVRKRTLRRLLQERELVEPLTPSGEAPNQALLRILKETEFKKIKVLGS

GAFGTVYKGLWIPEGEKVKIPVAIKELREATSPKANKEILDEAYVMASVDNPHVCRLLGI

CLTSTVQLITQLMPFGCLLDYVREHKDNIGSQYLLNWCVQIAKGMNYLEDRRLVHRDLAA

RNVLVKTPQHVKITDFGLAKLLGAEEKEYHAEGGKVPIKWMALESILHRIYTHQSDVWSY

GVTVWELMTFGSKPYDGIPASEISSILEKGERLPQPPICTIDVYMIMVKCWMIDADSRPK

FRELIIEFSKMARDPQRYLVIQGDERMHLPSPTDSNFYRALMDEEDMDDVVDADEYLIPQ

QGFFSSPSTSRTPLLSSLSATSNNSTVACIDRNGLQSCPIKEDSFLQRYSSDPTGALTED

SIDDTFLPVPEYINQSVPKRPAGSVQNPVYHNQPLNPAPSRDPHYQDPHSTAVGNPEYLN

TVQPTCVNSTFDSPAHWAQKGSHQISLDNPDYQQDFFPKEAKPNGIFKGSTAENAEYLRV

APQSSEFIGA

>Mus musculus EGFR

MRPSGTARTTLLVLLTALCAAGGALEEKKVCQGTSNRLTQLGTFEDHFLSLQRMYNNCEV

VLGNLEITYVQRNYDLSFLKTIQEVAGYVLIALNTVERIPLENLQIIRGNALYENTYALA

ILSNYGTNRTGLRELPMRNLQEILIGAVRFSNNPILCNMDTIQWRDIVQNVFMSNMSMDL

QSHPSSCPKCDPSCPNGSCWGGGEENCQKLTKIICAQQCSHRCRGRSPSDCCHNQCAAGC

TGPRESDCLVCQKFQDEATCKDTCPPLMLYNPTTYQMDVNPEGKYSFGATCVKKCPRNYV

VTDHGSCVRACGPDYYEVEEDGIRKCKKCDGPCRKVCNGIGIGEFKDTLSINATNIKHFK

YCTAISGDLHILPVAFKGDSFTRTPPLDPRELEILKTVKEITGFLLIQAWPDNWTDLHAF

ENLEIIRGRTKQHGQFSLAVVGLNITSLGLRSLKEISDGDVIISGNRNLCYANTINWKKL

FGTPNQKTKIMNNRAEKDCKAVNHVCNPLCSSEGCWGPEPRDCVSCQNVSRGRECVEKCN

ILEGEPREFVENSECIQCHPECLPQAMNITCTGRGPDNCIQCAHYIDGPHCVKTCPAGIM

GENNTLVWKYADANNVCHLCHANCTYGCAGPGLQGCEVWPSGPKIPSIATGIVGGLLFIV

VVALGIGLFMRRRHIVRKRTLRRLLQERELVEPLTPSGEAPNQAHLRILKETEFKKIKVL

GSGAFGTVYKGLWIPEGEKVKIPVAIKELREATSPKANKEILDEAYVMASVDNPHVCRLL

GICLTSTVQLITQLMPYGCLLDYVREHKDNIGSQYLLNWCVQIAKGMNYLEDRRLVHRDL

AARNVLVKTPQHVKITDFGLAKLLGAEEKEYHAEGGKVPIKWMALESILHRIYTHQSDVW

SYGVTVWELMTFGSKPYDGIPASDISSILEKGERLPQPPICTIDVYMIMVKCWMIDADSR

PKFRELILEFSKMARDPQRYLVIQGDERMHLPSPTDSNFYRALMDEEDMEDVVDADEYLI

PQQGFFNSPSTSRTPLLSSLSATSNNSTVACINRNGSCRVKEDAFLQRYSSDPTGAVTED

NIDDAFLPVPEYVNQSVPKRPAGSVQNPVYHNQPLHPAPGRDLHYQNPHSNAVGNPEYLN

TAQPTCLSSGFNSPALWIQKGSHQMSLDNPDYQQDFFPKETKPNGIFKGPTAENAEYLRV

APPSSEFIGA

>Rattus norvegicus EGFR

MRPSGTARTKLLLLLAALCAAGGALEEKKVCQGTSNRLTQLGTFEDHFLSLQRMFNNCEV

VLGNLEITYVQRNYDLSFLKTIQEVAGYVLIALNTVERIPLENLQIIRGNALYENTYALA

VLSNYGTNKTGLRELPMRNLQEILIGAVRFSNNPILCNMETIQWRDIVQDVFLSNMSMDV

QRHLTGCPKCDPSCPNGSCWGRGEENCQKLTKIICAQQCSRRCRGRSPSDCCHNQCAAGC

TGPRESDCLVCHRFRDEATCKDTCPPLMLYNPTTYQMDVNPEGKYSFGATCVKKCPRNYV

VTDHGSCVRACGPDYYEVEEDGVSKCKKCDGPCRKVCNGIGIGEFKDTLSINATNIKHFK

YCTAISGDLHILPVAFKGDSFTRTPPLDPRELEILKTVKEITGFLLIQAWPENWTDLHAF

ENLEIIRGRTKQHGQFSLAVVGLNITSLGLRSLKEISDGDVIISGNRNLCYANTINWKKL

FGTPNQKTKIMNNRAEKDCKATNHVCNPLCSSEGCWGPEPTDCVSCQNVSRGRECVDKCN

ILEGEPREFVENSECIQCHPECLPQTMNITCTGRGPDNCIKCAHYVDGPHCVKTCPSGIM

GENNTLVWKFADANNVCHLCHANCTYGCAGPGLKGCQQPEGPKIPSIATGIVGGLLFIVV

VALGIGLFMRRRHIVRKRTLRRLLQERELVEPLTPSGEAPNQAHLRILKETEFKKIKVLG

SGAFGTVYKGLWIPEGEKVKIPVAIKELREATSPKANKEILDEAYVMASVDNPHVCRLLG

ICLTSTVQLITQLMPYGCLLDYVREHKDNIGSQYLLNWCVQIAKGMNYLEDRRLVHRDLA

ARNVLVKTPQHVKITDFGLAKLLGAEEKEYHAEGGKVPIKWMALESILHRIYTHQSDVWS

YGVTVWELMTFGSKPYDGIPASEISSILEKGERLPQPPICTIDVYMIMVKCWMIDADSRP

KFRELILEFSKMARDPQRYLVIQGDERMHLPSPTDSNFYRALMEEEDMEDVVDADEYLIP

QQGFFNSPSTSRTPLLSSLSANSNSSAVACINRNGSCRVKEDAFLQRYSSDPTSVLTEDN

IDDTFLPVPEYINQSVPKRPAGSVQNPVYHNQPLHPAPGRDLHYQNPHSNAVSNPEYLNT

AQPTCLSSGFDSSALWIQKGSHQMSLDNPDYQQDFFPKEAKPNGIFKGPTAENAEYLRVA

PPSSEFIGA

>Xenopus tropicalis EGFR

VCLGNNNRLNQLGNTDEHYASLKRMYDGCEIVLGNLEITYLDRLNDTSFLRNIQEVGGYV

LIAINSVRSIHLENLQIVRGNMLYKSTYALAIVSNESLNTTNALEELHMGRLTEILNGGV

LISKNRKLCYLNTIQWNDIVHTDNPINKISVDKSFSPQCPKCDAESCNGSCWGPGQGNCQ

KLTKLTCAQQCSGRCRGPLPSDCCHSQCASGCTGPRENNCLACRKFRDGETCKEGCPQLQ

IYNPATYQMDLNPDGKYSFGASCVKKCPHNYVVTDHGSCVRTCNSDSQEVEENGIRICKK

CDGSCNKDKVCDGINMGKLKSVLSVNASNIDLFQNCTKVNGDVAILHVAGYGDPFTNTSK

LDPAKLSVFKSVREITGSLLIQWWPDNYTDLSVFENLETIRGRTKQHGIYSLAIVKRSIT

SLGLRSLKEVSDGDVIIKTNKNLCFVNTINFTNIFRIPKQFSVLGFLESEGNVCDPLCSE

EGCWGPGPFQCVSCRKYMRGRECVENCNFLSGEPREYTEDGKCFACNAECLQLNETQTCS

GPGADECFKCGHNSDGPFCVKTCPAGIQGENSTLIWKYPDETGACQLCHPECKLGCVGHG

LDGCPLPWSSHSVAAGVVGGILGAVVIGLVAFFFIRRSRINRKRTLRRILQERELVEPVT

PSGEAPNQALLRILKETEFKKIKVLGSGAFGTVYQGLWIPEGEGIKIPVAIKELREATSP

KANKEILDEAYVMASVENPYVCRLLGICLTSTVQLITQLMPFGCLLDYVRDNKDNIGSQH

LLNWCVQIAKGMNYLEERRLVHRDLAARNVLVKGPQHVKITDFGLAKLLGADEKAYHAEG

GKVPIKWMALESILHRTYTHQSDVWSYGVTVWELMTFGLKPYDGIPASEISNILEKGERL

PQPPICTIDVYMIMVKCWMIDAESRPKFRELSAEFTKMARDPTRYLVIQGDDRMHLPSPV

ESKIHPALVQEGLGDIVDAEEYLLPHQGFFTSPSNSRTPLLSTMGPPVREDSFVQRYSTD

PTVILEDSIDAFSEYVNQMVPKPIVETQTNAVYQNLVPLGNTVSPKETLYQNSHSNGLNN

PEYLNTGHTLVPKTDFINLDNPDYQQDFFPKEPKTNGHFKIPAAQNPEYLGLAPYLYTWA

R

>Tetraodon nigroviridis EGFR

CQGITNRLNRLSSTDDHYANMLKTYSNCTVVLENLEITHMEEHRDLSFLRSIEEVGGYVL

IALNTVSRIHLDNLRIIRGHSLYDNKFALKVISNYNKSTGLGTSELLLNSLTEILKGGVK

ISSKMLCNVDTIQWNDIVNTETKPAMELPEKSNNPQCQKCHSSCFNGSCWAPGSQNCQQL

TKLNCAQQCSRRCNGPAPSNCCNEHCAAGCTGPRATDCLACRDFQDDGVCKDSCPGLIRY

DPNLHQLVPNPHGKYNFGAKCVKTCPHNYVVTDHGACVRTCSGNTYEIEEGGIRKCAKCD

GLCPKVCNGLGTGELANTISINATNIESFRNCTKINGNINIIRTSFDGDSYTKTPKMHPS

QLDVFKTVREISGYLLIQQWPENLHSLSPFENLEIIRGRTKRGSRSLVVAGLSITSLGLR

SLKEISDGDVIITMNKNLCYTSESHWKKLFRSEGQSATIKENADVATCAQRNNICDRKCT

AEGCWGPGPDMCFTCSDYSRGGSCVDSCNILEGESRETVVNKTCVKCHPECERMNGTTTC

SAPGSGNCTRCANFQDGLFCVSRCPRGVPGEDDMLVWKYADNNKVCQLCHRNCTQGCTGP

GLAGCQDKSTSGFSLIAAGVVGGLLAVLLAGLSVFVLLRRRHIKRKRTMRRLLQERELVE

PLTPSGEAPNQALLRILKEPEFKKIKVLGSGAFGTVYKGLWVPEGEDVKIPVAIKVLREA

TSPKANKDILDEAYVMASVEHPHVCRLLGICLTSTVQLITQLMPFGCLLDYVKENKDNVG

SQYLLNWCVQIAKGMNYLEERHLVHRDLAARNVLVKTPQHVKITDFGLAKLLNADEKEYH

ADGGKGVTVWELMTFGTKPYDGIPASEIAGILEKGERLPQPPICTIDVYMIMVKCTWGWM

INAVNRPRFRELIAEFTKMARDPSRYLVIQGDDRMHLPSPTDKKLFRTLISGEGLGGRGR

CRRVFGAPAWILQQPKHFSHASAPLYNYMNQNGASDMMNPNYKYPGPPRSILPTISSDDT

ESEYLNCFKNGTIQPEYLNEVPSSSNFPFTPNTQKYAPQNSIDNPDYQQDFTPTFKTHTN

GHIPAAENTEYTSV

>Takifugu rubripes EGFR

SFKYAMAARFVTGFGLTSLLCLCCCVLAERKVCQGITNRLNLLGSREDHYANMLKTYSNC

TVVLENLEITYMEEDRDLSFLRSIEEVGGYVLIALNTVSKIRLDNLRIIRGHSLYEKKFA

LSVIANFNKSTGQGTSELLLNSLTEILKGGIKISGKVLCNVETIQWYDIVNVETKPVMEL

PVASNNPQCQKCDSSCFNGSCWAPGPQNCQTLTKLNCAQQCSKRCKGTSPIDCCNEHCAA

GCTGPRPTDCLACRDFQDDGVCKDSCPGLMRYDPNLHQLVPNPHGKYNFGATCVKTCPHN

YVVTDHGACVRTCSGNTYEVDEGGMRKCAKCEGLCPKVCNGLGTGELTNAMSINATNIES

FRNCTKINGNIAIIRTSIHGDAYTKTPKMHPSQLDVFKTVKEITGYLLIQTWPDNISSLS

PFENLEIIRGRTKRGSRSLMVANLGITSLGLRSLKEISDGDVIIMKNKNLCYTSKSHWKK

LFKSDSQHPTIDENADAATCAQRNNSCDRKCTAEGCWGSGPDMCFACSNYSRGGSCVDSC

NILEGEPREAVVDKTCVECHPECERMNGTATCIAPGSGNCTRCANSQDGLFCVSRCPQGV

PGEDETLVWKYADNNKVCQLCHKNCTQGCTGPGLAGCQNKSTSGFSMIAAGVVGGLLAAL

IAGLSVFVLLRRRHIKRKRTMRRLLQERELVEPLTPSGEAPNQALLRILKEPEFKKIKVL

GSGAFGTVYKGLWVPEGEDVKIPVAIKVLREATSPKANKEILDEAYVMASVEHPHVCRLL

GICLTSTVQLITQLMPYGCLLDYVKENRDNIGSQYLLNWCVQIAKGMNYLEERHLVHRDL

AARNVLVKTPQHVKITDFGLAKLLNADEKEYHADGGKVPIKWMALESILNRTYTHQSDVW

SYGKYCIYLCVTVWELMTFGTKPYDGIPASEIAGILEKGERLPQPPICTIDVYMIMVKCW

MIDADSRPRFRELIAEFTKMARDPSRYLVIQGDERMPLPSPTENKLFRTLISEEYMEDAI

DADEYLVPQHGFFSSPSTSHTPLLQSTVSCFMQNNEGFKSDEFAGRDGSFVHRYIPDPTD

KLLDDAFQPAPDYMNQNGVSDVMNPNYKYPGPPRSVLPTISSDDTESEYLNCFKNGTIEP

EYLNEVPSSSGFPFTPNNVVQNMQKYAPQNSIDNPDYQQDFTPTFKTHTNGHIPAAENTE

YLGPD

>Danio rerio EGFR

MAGPTEIGLFFTLLLSGSFCATPEKKVCQGANNKLTLLGTVEDHYQVLLRMYRNCTVVLE

NLEITHITEKYDLSFLKSIQEVGGYVLIAVNTVSKIPLENLRIIRGHSLYEDKFALAVLV

NYNNSIEQGVKELPLTSLTEILKGGVRFNMNNHLCNVGTIEWADILNMKSLPTIVSHNIS

YGKNCGKCDPSCFNGSCWGTGPDKCQRMTKVICAEQCSGRCKGPRPIDCCNEHCAAGCTG

PRPTDCLACKDFQDEGTCKDACPRLMLYDPNTHQLAPNPYGKYSFGATCIKTCPHNYVVT

DHGACVRTCSPGTYEVDEGGVRKCKRCEGLCPKVCNGLGMGPLANVLSINATNIDSFENC

TKISGNVAILSTTFRGDPHTNTSGLDPAKLSVLSTVKEITGYLMIQLWPESMQSLSAFEN

LEVIRGRTKTQGTYSFAVTKTAITHLGMRSLREISDGDVSIVKNKNLCYSSPEHWKRLFK

SKQQSVKMIENMDAATCANQNSTCNEMCTADGCWGPGPTMCFGCEHYSRGKHCVASCNLL

NGEPREYEVNKTCMECDPECLLMNETQTCNGPGPDKCTVCANYKDGPHCVHRCPQGVPGE

KDTLIWKYADVTHVCQPCHENCTQGCTGPDLKDCKDFKSSGLPMIAAGVVGGLLAFVILA

LGVAVLLRRRHIRRKRTLRRLLQERELVEPLTPSGEAPNQALLRILKETEFKKIKVLGSG

AFGTVHKGLWVPEGENVKIPVAIKVLREATSPKANKEIMDEAYVMASVEHPHVCRLLGIC

LTSTVQLITQLMPYGCLLDYVRENKDRIGSQHLLNWCVQIAKGMNYLEERHLVHRDLAAR

NVLVKTPQHVKITDFGLAKLLNADEKEYHADGGKVPIKWMALESIQHRTYTHQSDVWSYG

VTVWELMTFGTKPYDGIPASEIAGVLEKGERLPQPPICTIDVYMIMVKCWMIDAESRPRF

RELIAEFTKMARDPSRYLVIQGDDRMHLPSPSDSKFYRSLMSGELDEAVDADEYLVPNHS

FFSSPSTSRTQLLHSVSLNSSFGNCNSRNGNGYPVRENSMVLRYIPDPTERFQEGDFQPA

PGYNEYMNQNESSMINPVYQQPHGPPRTLLHSSPALDETEEEYLNCFKSPAPASVVEYLN

TSHTQLLSTKPFFSMDNPDYQQDFCPLELKTHTNGHLPAAQNQEYMGLEVH

>Drosophila melanogaster(CG10079-PA)

MMIISMWMSISRGLWDSSSIWSVLLILACMASITTSSSVSNAGYVDNGNMKVCIGTKSRL

SVPSNKEHHYRNLRDRYTNCTYVDGNLELTWLPNENLDLSFLDNIREVTGYILISHVDVK

KVVFPKLQIIRGRTLFSLSVEEEKYALFVTYSKMYTLEIPDLRDVLNGQVGFHNNYNLCH

MRTIQWSEIVSNGTDAYYNYDFTAPERECPKCHESCTHGCWGEGPKNCQKFSKLTCSPQC

AGGRCYGPKPRECCHLFCAGGCTGPTQKDCIACKNFFDEGVCKEECPPMRKYNPTTYVLE

TNPEGKYAYGATCVKECPGHLLRDNGACVRSCPQDKMDKGGECVPCNGPCPKTCPGVTVL

HAGNIDSFRNCTVIDGNIRILDQTFSGFQDVYANYTMGPRYIPLDPERLEVFSTVKEITG

YLNIEGTHPQFRNLSYFRNLETIHGRQLMESMFAALAIVKSSLYSLEMRNLKQISSGSVV

IQHNRDLCYVSNIRWPAIQKEPEQKVWVNENLRADLCEKNGTICSDQCNEDGCWGAGTDQ

CLTCKNFNFNGTCIADCGYISNAYKFDNRTCKICHPECRTCNGAGADHCQECVHVRDGQH

CVSECPKNKYNDRGVCRECHATCDGCTGPKDTIGIGACTTCNLAIINNDATVKRCLLKDD

KCPDGYFWEYVHPQEQGSLKPLAGRAVCRKCHPLCELCTNYGYHEQVCSKCTHYKRREQC

ETECPADHYTDEEQRECFQCHPECNGCTGPGADDCKSCRNFKLFDANETGPYVNSTMFNC

TSKCPLEMRHVNYQYTAIGPYCAASPPRSSKITANLDVNMIFIITGAVLVPTICILCVVT

YICRQKQKAKKETVKMTMALSGCEDSEPLRPSNIGANLCKLRIVKDAELRKGGVLGMGAF

GRVYKGVWVPEGENVKIPVAIKELLKSTGAESSEEFLREAYIMASVEHVNLLKLLAVCMS

SQMMLITQLMPLGCLLDYVRNNRDKIGSKALLNWSTQIAKGMSYLEEKRLVHRDLAARNV

LVQTPSLVKITDFGLAKLLSSDSNEYKAAGGKMPIKWLALECIRNRVFTSKSDVWAFGVT

IWELLTFGQRPHENIPAKDIPDLIEVGLKLEQPEICSLDIYCTLLSCWHLDAAMRPTFKQ

LTTVFAEFARDPGRYLAIPGDKFTRLPAYTSQDEKDLIRKLAPTTDGSEAIAEPDDYLQP

KAAPGPSHRTDCTDEIPKLNRYCKDPSNKNSSTGDDETDSSAREVGVGNLRLDLPVDEDD

YLMPTCQPGPNNNNNINNPNQNNMAAVGVAAGYMDLIGVPVSVDNPEYLLNAQTLGVGES

PIPTQTIGIPVMGVPGTMEVKVPMPGSEPTSSDHEYYNDTQRELQPLHRNRNTETRV

>Caenorhabditis elegans(Let-23)

MRYPPSIGSILLIIPIFLTFFGNSNAQLWKRCVSPQDCLCSGTTNGISRYGTGNILEDLE

TMYRGCRRVYGNLEITWIEANEIKKWRESTNSTVDPKNEDSPLKSINFFDNLEEIRGSLI

IYRANIQKISFPRLRVIYGDEVFHDNALYIHKNDKVHEVVMRELRVIRNGSVTIQDNPKM

CYIGDKIDWKELLYDPDVQKVETTNSHQHCYQNGKSMAKCHESCNDKCWGSGDNDCQRVY

RSVCPKSCSQCFYSNSTSSYECCDSACLGGCTGHGPKNCIACSKYELDGICIETCPSRKI

FNHKTGRLVFNPDGRYQNGNHCVKECPPELLIENDVCVRHCSDGHHYDATKDVRECEKCR

SSSCPKICTVDGHLTNETLKNLEGCEQIDGHLIIEHAFTYEQLKVLETVKIVSEYITIVQ

QNFYDLKFLKNLQIIEGRKLHNVRWALAIYQCDDLEELSLNSLKLIKTGAVLIMKNHRLC

YVSKIDWSSIITSKGKDNKPSLAIAENRDSKLCETEQRVCDKNCNKRGCWGKEPEDCLEC

KTWKSVGTCVEKCDTKGFLRNQTSMKCERCSPECETCNGLGELDCLTCRHKTLYNSDFGN

RMECVHDCPVSHFPTQKNVCEKCHPTCYDNGCTGPDSNLGYGGCKQCKYAVKYENDTIFC

LQSSGMNNVCVENDLPNYYISTYDTEGVIETHCEKCSISCKTCSSAGRNVVQNKCVCKHV

EYQPNPSERICMDQCPVNSFMVPDTNNTVCKKCHHECDQNYHCANGQSTGCQKCKNFTVF

KGDIAQCVSECPKNLPFSNPANGECLDYDIASRQRKTRMVIIGSVLFGFAVMFLFILLVY

WRCQRIGKKLKIAEMVDMPELTPIDASVRPNMSRICLIPSSELQTKLDKKLGAGAFGTVF

AGIYYPKRAKNVKIPVAIKVFQTDQSQTDEMLEEATNMFRLRHDNLLKIIGFCMHDDGLK

IVTIYRPLGNLQNFLKLHKENLGAREQVLYCYQIASGMQYLEKQRVVHRDLATRNVLVKK

FNHVEITDFGLSKILKHDADSITIKSGKVAIKWLAIEIFSKHCYTHASDVWAFGVTCWEI

ITFGQSPYQGMSTDSIHNFLKDGNRLSQPPNCSQDLYQELLRCWMADPKSRPGFEILYER

FKEFCKVPQLFLENSNKISESDLSAEERFQTERIREMFDGNIDPQMYFDQGSLPSMPSSP

TSMATFTIPHGDLMNRMQSVNSSRYKTEPFDYGSTAQEDNSYLIPKTKEVQQSAVLYTAV

TNEDGQTELSPSNGDYYNQPNTPSSSSGYYNEPHLKTKKPETSEEAEAVQYENEEVSQKE

TCL

**GLI zinc-finger protein family**

>Homo sapiens GLI1

MFNSMTPPPISSYGEPCCLRPLPSQGAPSVGTEGLSGPPFCHQANLMSGPHSYGPARETN

SCTEGPLFSSPRSAVKLTKKRALSISPLSDASLDLQTVIRTSPSSLVAFINSRCTSPGGS

YGHLSIGTMSPSLGFPAQMNHQKGPSPSFGVQPCGPHDSARGGMIPHPQSRGPFPTCQLK

SELDMLVGKCREEPLEGDMSSPNSTGIQDPLLGMLDGREDLEREEKREPESVYETDCRWD

GCSQEFDSQEQLVHHINSEHIHGERKEFVCHWGGCSRELRPFKAQYMLVVHMRRHTGEKP

HKCTFEGCRKSYSRLENLKTHLRSHTGEKPYMCEHEGCSKAFSNASDRAKHQNRTHSNEK

PYVCKLPGCTKRYTDPSSLRKHVKTVHGPDAHVTKRHRGDGPLPRAPSISTVEPKREREG

GPIREESRLTVPEGAMKPQPSPGAQSSCSSDHSPAGSAANTDSGVEMTGNAGGSTEDLSS

LDEGPCIAGTGLSTLRRLENLRLDQLHQLRPIGTRGLKLPSLSHTGTTVSRRVGPPVSLE

RRSSSSSSISSAYTVSRRSSLASPFPPGSPPENGASSLPGLMPAQHYLLRARYASARGGG

TSPTAASSLDRIGGLPMPPWRSRAEYPGYNPNAGVTRRASDPAQAADRPAPARVQRFKSL

GCVHTPPTVAGGGQNFDPYLPTSVYSPQPPSITENAAMDARGLQEEPEVGTSMVGSGLNP

YMDFPPTDTLGYGGPEGAAAEPYGARGPGSLPLGPGPPTNYGPNPCPQQASYPDPTQETW

GEFPSHSGLYPGPKALGGTYSQCPRLEHYGQVQVKPEQGCPVGSDSTGLAPCLNAHPSEG

PPHPQPLFSHYPQPSPPQYLQSGPYTQPPPDYLPSEPRPCLDFDSPTHSTGQLKAQLVCN

YVQSQQELLWEGGGREDAPAQEPSYQSPKFLGGSQVSPSRAKAPVNTYGPGFGPNLPNHK

SGSYPTPSPCHENFVVGANRASHRAAAPPRLLPPLPTCYGPLKVGGTNPSCGHPEVGRLG

GGPALYPPPEGQVCNPLDSLDLDNTQLDFVAILDEPQGLSPPPSHDQRGSSGHTPPPSGP

PNMAVGNMSVLLRSLPGETEFLNSSA

>Mus musculus GLI1

MFNPMTPPQVNSYSEPCCLRPLHSQGVPSMGTEGLSGLPFCHQANFMSGSQGYGAARETS

SCTEGSLFPPPPPPRSSVKLTKKRALSISPLSDASLDLQTVIRTSPSSLVAFINSRCTSP

GGSYGHLSIGTMSPSLGFPPQMSHQKGTSPPYGVQPCVPHDSTRGSMMLHPQARGPRATC

QLKSELDMMVGKCPEDPLEGDMSSPNSTGIQDHLLGMLDGREDLEREEKPEPESVYETDC

RWDGCSQEFDSQEQLVHHINSEHIHGERKEFVCHWGGCSRELRPFKAQYMLVVHMRRHTG

EKPHKCTFEGCRKSYSRLENLKTHLRSHTGEKPYMCEQEGCSKAFSNASDRAKHQNRTHS

NEKPYVCKLPGCTKRYTDPSSLRKHVKTVHGPDAHVTKRHRGDGPLPRAQPLSTVEPKRE

REGGSGREESRLTVPESAMPQQSPGAQSSCSSDHSPAGSAANTDSGVEMAGNAGGSTEDL

SSLDEGPCVSATGLSTLRRLENLRLDQLHQLRPIGSRGLKLPSLTHAGAPVSRRLGPPVS

LDRRSSSSSSMSSAYTVSRRSSLASPFPPGTPPENGASSLPGLTPAQHYMLRARYASARG

SGTPPTAAHSLDRMGGLSVPPWRSRTEYPGYNPNAGVTRRASDPARAADHPAPARVQRFK

SLGCVHTPPSVATGRNFDPHHPTSVYSPQPPSITENVAMDTRGLQEEPEVGTSVMGNGLN

PYMDFSSTDTLGYGGPEGTAAEPYEARGPGSLPLGPGPPTNYGPGHCAQQVSYPDPTPEN

WGEFPSHAGVYPSNKAPGAAYSQCPRLEHYGQVQVKPEQGCPVGSDSTGLAPCLNAHPSE

GSPGPQPLFSHHPQLPQPQYPQSGPYPQPPHGYLSTEPRLGLNFNPSSSHSTGQLKAQLV

CNYVQSQQELLWEGRNRGGLPNQELPYQSPKFLGGSQVSQSPAKTPAAAAAAYGSGFAPA

SANHKSGSYPAPSPCHETFTVGVNRPSHRPAAPPRLLPPLSPCYGPLKVGDTNPSCGHPE

VGRLGAGPALYPPPEGQVCNALDSLDLDNTQLDFVAILDEAQGLSPPLSHEQGDSSKNTP

SPSGPPNMAVGNMSVLLGSLPGETQFLNSSA

>Rattus norvegicus GLI1

MFNPMTSPQVNSYGEPCCLRPLPSQGVPSMGTEGLSGLPFCHQANFMSGSQGYGSARETS

SCTEGSLFPPPRSSVKLTKKRALSISPLSDASLDLQTVIRTSPSSLVAFINSRCTSPGGS

YGHLSIGTMSPSLGFPPQMSHQKGTSPPYGVQPCVPHDSTQGSMMLHPQSRGPRATCQLK

SELDIMVGKCPEEPLEGDMSSPNSTSTQDHLLGMLDGREDLEREEKPEPESVYETDCRWD

GCSQEFDSQEQLVHHINSEHIHGERKEFVCHWGGCSRELRPFKAQYMLVVHMRRHTGEKP

HKCTFEGCRKSYSRLENLKTHLRSHTGEKPYMCEQEGCSKAFSNASDRAKHQNRTHSNEK

PYVCKLPGCTKRYTDPSSLRKHVKTVHGPDAHVTKRHRGDGPLPRAQPLSTVEPKREREG

GSGREESRLTVPEGTMPQQSPGAQSSCSSDHSPAGSAANTDSGVEMAGNAGGSTEDLSSL

DEGPCVAATGLSTLRRLENLRLDQLHQLRPIGSRGLKLPSLTHAGAPVSRRLGPPVSLDR

RSSSSSSISSAYTVSRRSSLASPFPPGSPPENGASSLPGLTPAQHYMLRARYASARGSGT

PPTAAHSLDRIGGLPAPPWRSRAEYPGYNPNAGVTRRASDPARAADHPAPARVQRFKSLG

CVHTPPSVATGRNFDPHLPTSVYSPQPPSITENVAMDTRGLQGELEVGTSMMGNDLNPYM

DFSSADTLGYGGPEGTAAESYGSKGPGSLPLGPGPPTNYGPGHCAQQVSYPDPTPETWGE

FPSHAGVYPGTKALGTAYNQCPRLEHYGQVQVKPEQGCPVGSDSTGLAPCLNAHPSEGSP

DPQPLFSHHSQLPPPQYLQSGPYPQPPHDCLSLESRPGLNFNPSSHSTGQLKAQLLCNYV

QSQQELLWEGRSRGGIPNQELPYQSPKFLGGSQVSQSPTKVPAAAAAATFGSGFASPALA

NHKSVSYPAPSPCHETFTMGVNRPPHRPAAPPRLLPPLPPCYGPLKVGDANPSCGHPEVG

RLGAGPALYPPPEGQVCNPLDSLDLDNTQLDFVAILDEAQGLSPPPSHEQGDSSKNTPPP

SGPPNMAVGNMSVLLGSLPGETQFLNSSA

>Tetraodon nigroviridis GLI1

MYNPMTSAIAHGAGTGIGLCTDQYMRPPQALPPHSMMGHRGMPPPEGGNSTPYCNQSNMM

PSHHNFCQAQGNSDQIGSGDGSRFSTPRSMLKLSKKRALSISPLSDASVDLQTVIRTSPN

SLVAFVNSRGNANGASSYGHLSVGAMSPSLGYTSNISCQSRQQGSLYGGGGVTPLGHTPG

PCQGSRLPPHNPRLHAPPKHGHLKTEPGLSSVMDGMNVKSLEERSEGDVASPSSTGTQDP

LLGLLDGRDDLDKEDGKPEQEAIYETNCHWESCNKEFDTQDQLVHHINNEHIHGEKKEFV

CHWQDCSREQRPFKAQYMLVVHMRRHTGEKPHKCTFEGCNKAYSRLENLKTHLRSHTGEK

PYVCEHEGCNKAFSNASDRAKHQNRTHSNEKPYVCKIPGCTKRYTDPSSLRKHVKTVHGP

EAHITKKHRGDTGPRPPGSAMTPGGQSSELLLEKEEARRDDCKLMAPETSLKSQPSPGGQ

SSCSSERSPLGSANNNDSGVEMNLNAAGSLEDLTALEDGVGGGGGGGGEPGNVGGTMAMS

AQALKRLENLKIDKLKQICRPSPPARCAGSKLPAIPGTGDNMGMCVPPPLLSNRRVMELS

SHELGGSASVGCSNDRRGSGTSSLSSAYTVSRRSSMVSPYLSSRRSSEVSQMGGTAGGGC

HSFGSEIVADPLSPETNRRGAPCPGGGLPGLSNLTPAQQYNLKAKYAAATGGPPPTPLPN

MEQMGATTARRGGYLSEYQGQPLPPFLTQGGPRRHSANTEYGTGVIYPHQAPGNNTRRAS

DPLSPGHDSLRCPDQAYMHGRYQNPGGDVSSKAGNSTGQARPFQSEGMSNSLLQQTEYSM

STCQLSPSAPHYPSIGQGGDAGGRWSDNHSHIPTPAHELQSQQGLQYTDAQHTHFNSQPG

LYNSPGGTHKLVIKPEQQFHVGMGGRDACQSAKLQQQRMLLQQAQGYPQPAGAVMMRNSS

SSNCGFQAQNQTTFASGGSVSLGCAGKALSDGQRSETPMMQVKEMMVRNYVQSQQALMWE

QQQEQQQQNPTGDSKSVPLSPYAESGPIANALENLDLDNTRIDFTSIIEDAESSSFGPIN

NTLEGQPGPSSQASSRLTTPQNSVSLSAESGLSNMAVGDMTSMLTSLAGENKFLNTLS

>Gasterosteus aculeatus GLI1

MPVDMQPHQGLYHYETSPSHPSRGLVPSDQSPYTDVSSLRAPLLNGHPQDCRAMYNPMTP

SMAHGSGQGIGNCMDQYMRPPQAPPPHSMMGHRGMPPTDGGNSTAYCNQNNMMSSHHNFC

QFQSSSDQIGSGDGSRFSTPRSILKLSKKRALSISPLSDASVDLQTVIRTSPNSLVAFVN

SRCNPNGASSYGHLSVSAMSPSLGYSSNINCQSRQQVSMYGGGGGTPLGGHSTGPCQASR

LPPHNPRLHAPPKHGHLKTEPGLGGVMDGMNVKSLEERSEGDVASPSSTGTQDPLLGLLD

GRDDLDKEDGKPEPEAIYETNCHWESCNKEFDTQDQLVHHINNEHIHGEKKEFVCHWQEC

SREQRPFKAQYMLVVHMRRHTGEKPHKCTFEGCNKAYSRLENLKTHLRSHTGEKPYVCEH

EGCNKAFSNASDRAKHQNRTHSNEKPYVCKIPGCTKRYTDPSSLRKHVKTVHGPEAHITK

KHRGDTGPRPPGSAMTPGGQSSELLLEKEETRREDCKLLAPETTLKSQPSPGGQSSCSSE

RSPLGSANNNDSGVEMNLNAAGSLEDLTALEDGVAGGGEPVGVGTMGMSSQALKRLENLK

IDKLKQIRRPTPPGRCSNNRLPALPGPGENMGMCAPSPLLSNRRVMELSNHELGGGAPIG

CSSNDRRGSGTSSLSSAYTVSRRSSMVSPHLSSRRSSEVSQMGGTGGGGCHHLSAEKSVG

DPLSPETNRRGAPCPGGGLPGLPNLTPAQQYSLKAKYAAATGGPPPTPLPNMEQPGTPAR

RGVLSEYQGQPLPPFLQKGGPRRHSANTEYGTGVIYPHQAPGNHSRRASDPVRSVADPQA

LPKRFNSLNNVAMMGRRNALHRGSDTSLARHLYSPRPPSITENVMMEAMGMEPHLAPIDA

RDRSMMMPPGERSFMGYQQQHQSLGGVGGPLGNQLSPSHDSLSCPDQVYMQGPYQNQGGE

VTSRAGGNPMGQVRPIHSEGISNALLQQAEYSMSTCQLSPSGPHYPSIGQGGDAGGPWSD

SHGQNQRGLQYSDPSLQPQQTQAHFNNQTGLYSSPDGTHKLTIKPEQQFHPGMGGGNACQ

SAKLQQQQRMLVQQPQGYPQQTGQVLMGNSNNPSCDFQGQNQNSFPAAGGLSLGCAGTAL

ADGQRSETPMMQVKEMMVRNYVQSQQALMWEQQQEQQQQSGIKPPPLSDNMDMSGQTAIM

QHSPQHQNQTLYSSQPYPSYPNQNLAMSPPGHSRGPGSVTPKDQQLAGLQVSCYGQEMVV

PRPPQGRKPLSRQNSLSQVGVGYLGSPPHLSPVHSASSPRRVVRLPPVQHPQHQQNEMFS

PSNNNMYYSGQISMDMDKHMDPQIGTCLNQPHTMRSNLDPTGGTKTSPMTPYPESGPISN

ALEHLDLDNARIDFTSIIDDAESSSFSPINNPLHGLPGSSSQASSRLTTPQTSGLSNMAV

GDMTSMLTSLAGENKYLNTLS

>Danio rerio GLI1

MPVDMQPHQGLYHYDNTSNQPSRGLAPSVRSPYSSEASSVCVALSGGQSVNSREMYNPSM

APGACMEPYMRAPHAPPPHSMMGHRGMPPPEGMSGAPYCNQNMMTSHHNLPHNQHTSELM

ASGDASCFSTPRSMLKLSKKRALSISPLSDASVDLQTVIRTSPNSLVAFVNSRCGPNNPS

SYGHLSVGTMSPSLGFSSSINYSRPQGNIYSHPVPSCIGAPARLPPHNPRLHTPAKHTHL

KTEPVLGSVMDSINIKGLEDHSEGDVASPSSTGTQDPLLGLLEGRDDLDKEEKPEPEAIY

ETNCHWESCSKEFDTQEQLVHHINNEHIHGEKKEFVCHWKDCSREQRPFKAQYMLVVHMR

RHTGEKPHKCTFEGCNKAYSRLENLKTHLRSHTGEKPYVCEHEGCNKAFSNASDRAKHQN

RTHSNEKPYICKIPGCTKRYTDPSSLRKHVKTVHGPEAHITKKHRGDTGPRPPGLTTAGQ

SSELLIEKEERNREDCKLLAPDNTLKSQPSPGGQSSCSSERSPLGSANNNDSGVEMNLNA

AGSLEDLTTQEDSGNAGVSESSATISSGGMCMSVQALKRLENLKIDKLKQIRRPTPPGRN

AGNKLPALSATGEMMSMCAPSPLLSNRRVMELSAPDMGGVTGMSCPPNDRRGSGTSSLSS

AYTVSRRSSMVSPYLSSRRSSDVSHCQSVMGGEVPGDPLSPQNSQRAGLCQNSGGLPGLP

SLTPAQQYSLKAKYAAATGGPPPTPLPNMDQAGTPARHVGFLRECQGQPLPPFLQQGGTR

RHSANAEYGTGVIYPHQAPGNNTRRASDPVRSAADPQGLPKVQRFNSLSNVSLMSRRNAL

QQCGSDAALSRHMYSPRPPSITENVMMEAMGMDGNTEGRQQGNMIPGGDRSYMGYQHNPH

QASQLSPGQESLGCIDQVYQSQMQGQYQREESCSTGVMGQADIANNLLQQAEYGMSTCQL

SPSGPHYPSQGDGSGPWGQTNQLHSPGMQYQGAGMQGQHYTQQGIYDPTSNPNLQRVTVK

PEQFHPSMGGSSSCQNTKALHQNRHNANMQTYPLQGQGIMNRSSSASCDFHHSQMGTQPN

QGGSFQSGTGINLALAESRRSQTPMHQMKEMMVRNYVQSQQALLWEQQQEQSVSEKPDGM

DMGQTQMMQHSPQHQQANQNLYPGNTYQGYPNQNLMSPQQNRVPGSVKEQMQSSCYGPDM

IPRPPQVRKSLSRQNSLSQQAGGAYLGSPPHLSPVHSTASPRRGVRLPPVQQQQQQQQQH

SENFNNNNNPMYYSGQMHMHHDLEKTPEGPCLAQQHLTSSDPTTKPTSISYPDPAPMSNA

LEHLDLENAQIDFTSIIDDQEPSSYSPINAPIGHNQCSSQTSSRLTTPQNSITLPSGLSN

MAIGDMSSMLTSLAGENKYLNTLS

>Takifugu rubripes GLI1

MPVDMQPHQGLYHYDSSPGQPSRGLVPSDQSPYSDVSSLRAPFLNGLPQDCRTMYNPMAS

SMTHGSGPGIGLCTDQYMRPPQALPPHSMMGHRGMPPPEGGNSTPYCTQSNMMPSHHNFC

QAQGNSDLIGSGDGSRFSTPRSILKLSKKRALSISPLSDASVDLQTVIRTSPNSLVAFVN

SRGNANGASSYGHLSVGAMSPSLGYTGNISCQSRQQGSIYGGGGVTPLGGHTPGPCQGSR

LPPHNPRLHAPPKHGHLKTEPGLSGVMDGMNVKSLEERSEGDVASPSSTGTQDPLLGLLD

GRDELDKEDGKTEQEAIYETNCHWESCSKEFDTQDQLVHHINNEHIHGEKKEFVCHWQEC

SREQRPFKAQYMLVVHMRRHTGEKPHKCTFEGCNKAYSRLENLKTHLRSHTGEKPYVCEH

EGCNKAFSNASDRAKHQNRTHSNEKPYVCKIPGCTKRYTDPSSLRKHVKTVHGPEAHITK

KHRGDMGPRPPGSAMTPGGQSSELLLEKEETRRDDCKLMAPETSLKSQPSPGGQSSCSSE

RSPLGSANNNDSGVEMNLNAAGSLEDLTALEDGVGGGGGGESASAGGTMGMSAQALKRLE

NLKIDKLKQICRPSPPARCGSNKLPALPGTGENLGMCVPSPLLSNRRVMELSSHELGGSA

SAGCSNDRRGSGTSSLSSAYTVSRRSSMVSPYLSSRRSSEVSQMGGTAGGGCHVFGSEQI

VADPLSPETNRRGAPCPGAGLPGLSNLTPAQQYNLKAKYAAATGGPPPTPLPNMEQIGTT

ARRGGVLSEYQGQPLPPFLTQGGPRRHSANTEYGTGVIYPHQAPGNNTRRASDPVRSAAD

PQALPKRFNSLNNVALMGRRNALQHRGSDTSLSRHLYSPRPPSITENVMMEAMSMDTHLT

PLDSRDHSMMMPPGERSFIGYQQQHQAHGGGASLSNQLSPSHDSLSCPDQAYMHGCYQNP

GGDVTSKDGNSIGQARPHQSEGMTNTLLQQTEYSMSTCQLSPSAPHYPSISQSGNAGGRW

GNNHSQIQSSPHELQSQQGMQYTDASLQSQQTHFNNQPGLYNGPGGTHKLVIKPEQQFHP

VMGGRDACQSAKLQQQRMLLQQTQGYPQQTGQVMMRNSSNSSCGFPVQNQNTFASGGSVS

LGCAGKALSDGQRSETPMMQVKEMMVRSYVQSQQALMWEQRQEQQQQSGIKPALSDNVDL

AAQTAMIQHSPQHQNQNLYPNQPYPSYPNQNMIMSPPSRGPSSVPPKDQQLTGLQGSCYG

QEMAPRPPQGRKPLSRQNSLSQVGGVYIGSSPHLSPVHSSSPRRGVRLPPVQHPQHPQNE

MFSPSNNNNNLYCSGQVNLDKIIDSQNGPCLNQQHGMGSGLDPTSDPKSAPLGPYPESGP

ISNALENLDLDNTRIDFTSIIDDAESSSFGPVNSTLQGQPGSSPQASSHLTTPQTSMSLT

AGSGLSNMAVGDMTSMLTSLAGENKYLNTLS

>GLI2 Homo sapiens

METSASATASEKQEAKSGILEAAGFPDPGKKASPLVVAAAAAAAVAAQGVPQHLLPPFHA

PLPIDMRHQEGRYHYEPHSVHGVHGPPALSGSPVISDISLIRLSPHPAGPGESPFNAPHP

YVNPHMEHYLRSVHSSPTLSMISAARGLSPADVAQEHLKERGLFGLPAPGTTPSDYYHQM

TLVAGHPAPYGDLLMQSGGAASAPHLHDYLNPVDVSRFSSPRVTPRLSRKRALSISPLSD

ASLDLQRMIRTSPNSLVAYINNSRSSSAASGSYGHLSAGALSPAFTFPHPINPVAYQQIL

SQQRGLGSAFGHTPPLIQPSPTFLAQQPMALTSINATPTQLSSSSNCLSDTNQNKQSSES

AVSSTVNPVAIHKRSKVKTEPEGLRPASPLALTQGQVSGHGSCGCALPLSQEQLADLKED

LDRDDCKQEAEVVIYETNCHWEDCTKEYDTQEQLVHHINNEHIHGEKKEFVCRWQACTRE

QKPFKAQYMLVVHMRRHTGEKPHKCTFEGCSKAYSRLENLKTHLRSHTGEKPYVCEHEGC

NKAFSNASDRAKHQNRTHSNEKPYICKIPGCTKRYTDPSSLRKHVKTVHGPDAHVTKKQR

NDVHLRTPLLKENGDSEAGTEPGGPESTEASSTSQAVEDCLHVRAIKTESSGLCQSSPGA

QSSCSSEPSPLGSAPNNDSGVEMPGTGPGSLGDLTALDDTPPGADTSALAAPSAGGLQLR

KHMTTMHRFEQLKKEKLKSLKDSCSWAGPTPHTRNTKLPPLPGSGSILENFSGSGGGGPA

GLLPNPRLSELSASEVTMLSQLQERRDSSTSTVSSAYTVSRRSSGISPYFSSRRSSEASP

LGAGRPHNASSADSYDPISTDASRRSSEASQCSGGSGLLNLTPAQQYSLRAKYAAATGGP

PPTPLPGLERMSLRTRLALLDAPERTLPAGCPRPLGPRRGSDGPTYGHGHAGAAPAFPHE

APGGGARRASDPVRRPDALSLPRVQRFHSTHNVNPGPLPPCADRRGLRLQSHPSTDGGLA

RGAYSPRPPSISENVAMEAVAAGVDGAGPEADLGLPEDDLVLPDDVVQYIKAHASGALDE

GTGQVYPTESTGFSDNPRLPSPGLHGQRRMVAADSNVGPSAPMLGGCQLGFGAPSSLNKN

NMPVQWNEVSSGTVDALASQVKPPPFPQGNLAVVQQKPAFGQYPGYSPQGLQASPGGLDS

TQPHLQPRSGAPSQGIPRVNYMQQLRQPVAGSQCPGMTTTMSPHACYGQVHPQLSPSTIS

GALNQFPQSCSNMPAKPGHLGHPQQTEVAPDPTTMGNRHRELGVPDSALAGVPPPHPVQS

YPQQSHHLAASMSQEGYHQVPSLLPARQPGFMEPQTGPMGVATAGFGLVQPRPPLEPSPT

GRHRGVRAVQQQLAYARATGHAMAAMPSSQETAEAVPKGAMGNMGSVPPQPPPQDAGGAP

DHSMLYYYGQIHMYEQDGGLENLGSCQVMRSQPPQPQACQDSIQPQPLPSPGVNQVSSTV

DSQLLEAPQIDFDAIMDDGDHSSLFSGALSPSLLHSLSQNSSRLTTPRNSLTLPSIPAGI

SNMAVGDMSSMLTSLAEESKFLNMMT

>Mus musculus GLI2

METSAPAPALEKKEAKSGLLEDSSFPDPGKKACPLAVAAAVAAHGVPQQLLPAFHAPLPI

DMRHQEGRYHYDPHSVHSVHGPPTLSGSPVISDISLIRLSPHPAGPGESPFSAHHPYVNP

HMEHYLRSVHSSPTLSMISAARGLSPADVAHEHLKERGLFSLAAPGTNPSDYYHQMTLMA

SHPTPYGDLLMQSGGAASAPHLHDYLNPVDASRFSSPRVTPRLSRKRALSISPLSDASLD

LQRMIRTSPNSLVAYINNSRSSSAASGSYGHLSAGALSPAFTFPHPINPVAYQQILSQQR

GLGSAFGHTPPLIQPSPTFLAQQPMTLTSISTMPTQLSSSSSNCLNDANQNKQNSESAVS

STVNPITIHKRSKVKTEAEGLRPASPLGLTQEQLADLKEDLDRDDCKQEAEVVIYETNCH

WADCTKEYDTQEQLVHHINNEHIHGEKKEFVCRWQACTREQKPFKAQYMLVVHMRRHTGE

KPHKCTFEGCSKAYSRLENLKTHLRSHTGEKPYVCEHEGCNKAFSNASDRAKHQNRTHSN

EKPYICKIPGCTKRYTDPSSLRKHVKTVHGPDAHVTKKQRNDVHVRAPLLKENGDNEASA

EPGGRGPEESVEASSTSHTVEDCLHIKAIKTESSGLCQSSPGAQSSCSSEPSPLGSAPNN

DSGMEMPGTGPGSLGDLTALADTCPGADTSALAAPSTGGLQLRKHMSTVHRFEQLKREKL

KSLKDSCSWAGPAPHTRNTKLPPLPVNGSVLENFNNTGGGGPAGLLPSQRLPELTEVTML

SQLQERRDSSTSTMSSAYTVSRRSSGISPYFSSRRSSEASPLGGLRPHNASSADSYDPIS

TDASRRSSEASQCSGGGPGLLNLTPAQQYNLRAKYAAATGGPPPTPLPGLDRVSLRTRLA

LLDAPERALPGACPHPLGPRRGSDGPTYSHGHGHGYAGAAPAFPHEGPNSSTRRASDPVR

RPDPLILPRVQRFHSTHNMNPGSLPPCADRRGLHVQSHPSVDSNLTRNAYSPRPPSINEN

VVMEAVAAGVDGPGLECDLGLVEDELVLPDDVVQYIKAHTGGTLDDGIRQGYPTEGTGFP

ENSKLPSPGLQGHRRLAAADSNMGPSAPGLGGCQLSYSPSSNLNKSNMPVQWNEVSSGTV

DALPTQVKPPPFPHSNLAVVQQKPAFGQYPGYNPQSVQSSSGGLDSTQPHLQLRGAPSAS

RGSYTQQPRQPAAGSQCLGMSAAMSPQASYSQAHPQLSPNIVSGSLNQFSPSCSNMAAKP

SHLGLPQQMEVVPNATIMNGHQREHGVPNSSLAAVSQPHPVLSYPQQDSYQQGSNLLSSH

QPGFMESQQNAGFGLMQPRPPLEPNTASRHRGVRSGQQQLYARTTGQAMVTSANQETAEA

MPKGPAGTMVSLAPQPSQDTGRAQDQNTLYYYGQIHMYEQNGGCPAVQPQPPQPQACSDS

IQPEPLPSPGVNQVSSTVDSQLLEPPQIDFDAIMDDGDHSSLFSGALSPTLLHNLSQNSS

RLTTPRNSLTLPSIPAGISNMAVGDMSSMLTSLAEESKFLNMMT

>Rattus norvegicus GLI2

MTLTSITVPTQLGSNSSNCISDANQNKQNSESAVSSTVNPITIHKRSKVKTEAEGLRPAS

PLGLTQEQLADLKEDLDRDDCKQEAEVVIYETNCHWADCTKEYDTQEQLVHHINNEHIHG

EKKEFVCRWQACTREQKPFKAQYMLVVHMRRHTGEKPHKCTFEGCSKAYSRLENLKTHLR

SHTGEKPYVCEHEGCNKAFSNASDRAKHQNRTHSNEKPYICKIPGCTKRYTDPSSLRKHV

KTVHGPDAHVTKKQRNDVHLRAPLLKENGDNEAGAEPGGRGPEESVEASSTSHTAEDCLH

IKAIKTESSGLCQSSPGAQSSCSSEPSPLGSVPNNDSGVEMPGTGPGSLGDLTALDDTSP

GADTSALAVPSTGGLQLRKHMTTMHRFEQLKREKLKSLKDSCSWAGPAPHTRNTKLPPLP

VNGSVLENFNNTGGCGPAGLLPNQRLPELTEVTMLSQLQERRDSSTSTMSSAYTVSRRSS

GISPYFSSRRSSEASPLGGIRPHNASSADSYDPISTDASRRSSEASQCSGGGPGLLNLTP

AQQYSLRAKYAAATGGPPPTPLPGLDRVSLRTRLALLDAPERALPGACPQPLGPRRGSDG

PTYNHGHGYVGAAPALPHEGPNGSTRRASDPVRRPDPLVLPRVQRFHSIHNMNPGPLPPC

TDRRGLHLQSHPSVDGSLTRHAYSPRPPSINENVVMEAVAAGVEGAGLESDLGLVEDDLV

LPDDVVQYIKAHTGGALDDSTRQVYPTEGTGFPENSKLPSPGLQGHRRLAAADSNMSPSA

PGLGGCQLSYSPSSSLNKSNMPVQWNEVSSGTVDALPTQVKPPSFPQSNLVQQKPAFAHY

PGYSPQALQSGSGGLDSTQPHLQLRGAPSASRGSYTQQPRLPATGSQCLGVTAAMSPQAN

YSQTHHQLSPNVVSGSLNQFAPSCSNMAAKPSHLGLPQQMEVVPNATIISGHQRELGIAS

SSLAAVSQPHPVLGYPQQDGYQQVSGLPSSHQPSFMESQQNTGFGLMQPRPPLEPNTASR

HRGVRSGQQQQQLYARTAGQAVVTSANQETAEAMPKGTAGTVVSLTPQPSQDTGRSQDQN

TLYYYGQIHMYEQNGGCPAMQPQPPQPQGCSDNIQPEPLPSPGVNQVSSTVDSQLLEPPQ

IDFDAIMDDGDHSSLFSGALSPSLLHNLSQNSSRLTTPRNSLTLPSIPVGISNMAVGDMS

SMLTSLAEENKFLNMMT

>Tetraodon nigroviridis GLI2

MAAPRLDAHPAPRPRGFSTSVSRFSSPRLTPRLSRKRALSISPLSDASIDLQTMIRTSPN

SLVAYINNSRSSSAASSSYGHLSVGPGIRNPVGGILDLSEDLDKDECKQEPEAVYETNCH

WEGCTKEYETQEQLVHHINNDHIHGEKKEFVCRWEECSREQKPFKAQYMLVVHMRRHTGE

KPHKCTGCSKAYSRLENLKTHLRSHTGEKPYLCEHEGCNKAFSNASDRAKHQNRTHSNEV

GPQLAAMPCSVHPNSELESPSFQKPYVCKIPGCTKRYTDPSSLRKHVKTVHGPEAHVTKK

QRSDMAPRPQPPKGNGVNEANSRHGAKGVEGKLEANSTSGGMENCLQIKSIKTENSMMYQ

SSPGGHSSCSSEPSPLGSTNNNDSGVEMAMHSGGSFADLSAQDESPMVDSTVPAGGQQAG

VGLHLRKAVGHSGTVTIKLENIKKERLKTVSTSDWVSSAAQPLQGQRSSVKLPPIPAVGS

LLENSTMMSNSSVCYPGPRKGDLSSCEVTLLNQLNERRDSTTSTISSAYTVSRRSSGISP

GYSSRRSSEASQFGANRHNNVSSADSYDPISTDLSRRSSEASQCGVGGGATLLSLTPAQH

YRLKAKYAAATGGAPPTPLPNMDRMSLRTRMALYSDSQEASLHQFHQPPTGAVPRRCSDM

GYGTRSMMPHEVPSSLPRRASDPVRRPPLDPLSLPRVQRYNSMNNMNPLNVTTAERYQSL

LAQGYTRSDGNLQRYPFAPRPPSISENVAMENMAMDGMAGGDQNGEDDMVLPDDMVQYLR

SQNTDPSANNSCQVDYHSNHQNQGFQAGIAPPPAYYSQRRMAMVDASMPHSGQDSQQPFS

APPANTNKNNMPVQWNEVSSGTVDSTSKLSKQQHHPLRGNLAVVQQRHNFSSFQGPSQAL

SSNQQVVPMSQNVSTQAYANHSNQRLTHASQPQQQQQRQYIPANFGQHMSPQQGFGQEFI

PNSISGSTSMRPAQNGMASSEPQSYRARTQTDGYCRVNPMDVQQNYNVLTQQHQSIHSGA

RGSLQPRPPAEPRSSLRQHLGSNMMPPNRVPKSSALSPCDGNETSEASPKRCSGLAAQNS

NSENSVFYTGQIHMFEPSFDAPMSPCASEPPANSTTAASMASPGVNLVSSSTTDSSTGAS

GGAQHPQIDFDTMLDDGDHSSLMSGTLSPGLLQSLSQSSSRLTTPRNSLPLASVPAGIGN

MAIGDMNSMLTALAEESKFLNMMS

>Gasterosteus aculeatus GLI2

HLFPTFHTPIPIDMRHHEGRYHYEPHPLHAMHGPPGLTGSPVISDISLIRLSPHVAPGTG

ESPFSPPHHYVSPHMEHYLRSVHGSPTLSMISAARGLSPAEVTHEHLKERALFNLPPPPP

GANPTDYYHLMASASQRSPYGDLLIQSTAHLPEYISPMDVSRFSSPRLTPRLSRKRALSI

SPLSDASIDLQTMIRTSPNSLVAYINNSRSSSAASSSYGHLSVGGISPSFSFPHPINPVA

YQQLLSHQRSLNAFGHTPPLIHPSPSSFSARQHPLAASPMTTSHNTSSSEANQCVNTCKN

ASGDPAVSSTVNPLSTKRSKVKTEAEGPLPISPSSQDHCGGILDLSEDLDKDECKQEPEA

IYVTDCHWEGCSKEYDTQDQLVHHINNDHIHGEKKEFVCRWEECSREQKPFKAQYMLVVH

MRRHTGEKPHKCTFEGCAKAYSRLENLKTHLRSHTGEKPYVCEHEGCNKAFSNASDRAKH

QNRTHSNEKPYVCKIPGCTKRYTDPSSLRKHVKTVHGPDAHVTKKQRSDAPPRPQPPRGD

GENGANSKIADGRVEANSTSRGVEDCLQVKSIKTENSMTYQSSPGGHSSCSSEPSPLSSA

NNNDSGVEMALHSGGSFGDLSALDDCPMVDSTVSAGGQQAGVGLQLRKAVGHVGTVTIKL

ENIKKERLKTVRDSCPWVNSAPQPPQGQRSSMKLPPIPAVGSLLESSNMISNLSGSYPGQ

RIGDLSSSEITLLNQLNERRDSTTSTMSSAYTMSRRSSGISPCYSSRRSSEASQFGTNRH

NNISSADSYDPISTDLSRRSSEASHCGGGGVGGGGLPSVLSLTPAQHYRLKAKYAAAIGG

APPTPLPNMDRMSLRTRMALYGDPQEGTLHPFHQPDFGTVPRRCSDIGYGTRSMMPHEVP

TSLPRRASDPVRRPTLDPLSFPRVQRYNSMNSMNPVNGPPAERHQALTMQGYTRSDGSLQ

RYPFAPRPPSISENVAMENMAVDGMIIGGEQGGEDDMVLPDDVVQYLRSQNSGPSGHNLG

QGDFHSNNLTKGYQTGMTSSASFYAQRRMAMADANMTQSGQDMQSQQPFSAPPGNVNKNN

MPVQWNEVSSGTVDTTKKLSKQHQHPLRGNLAVVQPRHNLGSFQGQGQGLGSNQQVVPMS

QNMSIQGYANHNSQRMMSIPPQQHQQLRQCNPVNMSEQMSPQQGFGQEIIPNSISGSTSV

RPTRNNMAVTEVHSYRARTQVDGYCHVNQVDQQQNYSVVSQQHNIHNGGRGMLQPRPPIE

PKAIARQQTGSSMMQPNRIPKSSDLNPSFGTDTSEASPKRPTGSSAHNCNSANPNSAMLY

SGQVHMFAPTSVSFDASMPPSASHAPASNTTAADHMASPGVNHVSSSTVDSSTNAAAGTE

HAQIDFDTLLDDGDHSSLMSGTLSPGLLQSLSQSSSRLTTPRNSVTLTSVPAGIGNMAIG

DMNSMLTALAEENKFLNLIS

>Danio rerio GLI2b

MEAGVPAPAEKKDCKSSPLEGNVFSEMPKKPSPSSLSRGPHHIFPTFHSPIPIDVRHHEG

RYHYDPHALHALHGPPGLAGSPVISDISLIRLSPGAAGEAFLPPHSYVSPHVEHYLRSAH

SSPTLSMISAARGLSPAEVAHEHLKERGLFGLPPPPGASPADYYHLMAGHRNPYGELLMQ

GAGAAAAAAHLPEYFTSVDVSRFPSPRMTPRLSRKRALSISPLSDASIDLQTMIRTSPNS

LVAYINNSRSSSAASSSYGHLSVGGISPSFPFPHSINPVAYQQLLTQQRGLSAFGHTPPL

IQPSPTFSSRHPLSLSTLPTPTTNDTETKNSNGESAVSSTVNPLGNKRSKVKTELGGPRP

VSPYSPEHLSSAQDLKEDMDDCKQEAELVYETNCHWEGCSKEYDTQEQLVHHINNEHIHG

EKKEFVCRWDECSREQKPFKAQYMLVVHMRRHTGEKPHKCTFEGCSKAYSRLENLKTHLR

SHTGEKPYVCEHEGCNKAFSNASDRAKHQNRTHSNEKPYVCKIPGCTKRYTDPSSLRKHV

KTVHGPEAHVTKKQRGDLPSRPHPPKENGENEAGAKMAEEKIEANSTTRGVEDYLQVKSI

KTENSMMYQSSPGGQSSCSSEPSPLGSASLHGGGVEPAGLSGGSVGDLSALDDVPIVDST

VSTGILGLHLRKNAGPAHRLTHLKQEKLKSVRDSCSWANTAPPAPCTKLPPINTGSLLDG

LCDPGLSISSPRLGDLCPGETTVLSQLVERRDSLASTVSSAYTFSRRSSGISPCYSSRRS

SQTSQPGGRHNNISSADSYDPISTDLSRRSSEASLYGGIFSLTPAQHYRLKAKYAAATGG

APPTPLPNIDCKTLLGDSHEIPIHPPLVPRRCSDTGYANRSVLPHEVTANITRRASDPVR

RIAAERHSLYNSVNPYPTLHPLASSRHFYSTSESNVNRQQYPPRAPSISENVMMETLPDD

MESCNGVEDGLMMADDAVQCITTQNADSPQHTGVAHRNQSFGSPQHCHLQRRLALVEGNM

NAVARQTASSQQTFPGSANATKIPMPVQWNEVSSGTVDSSRSQTKQGSGRGNLQQKQNLS

SFLNVNSNQQVGLMSHNLALSPVQRSVEFNSLRINQRQANAEFLHGYLQGSASNDISLGL

LNPSGKSLTVQPGGVQNNRVCAQAQSHATFPNNQGNYSQMSSNQQGYIVASQNVNGNLLL

QPRPPTEPKPYSTQHSGPTTTAAQSNFSPAYDTSEASPKKSSSMFYTGQIHMFDSSGAMP

CPSSVGSPETNQVSSTVDSSGADPPQIDFDAMLDDGDHSSLMSGTLSPALLRSLSQNSSR

LTTPRNSVTLPPVAAGISNMAIGDMSSMLTALAEESKFLNMMS

>Danio rerio GLI2a

METTSPTSTEKKELKPSVLDGSSFTDLPKKPSPTTASRAPHLFSTFHTPIPIDMRHHEGR

YHYEPHPLHPMHGPHGLAGSPVISDISLIRLSPHAAATGESPFNPPHPYVNPHMEHYLRS

VHSSPTLSMISAARGLSPAELTHEHLKERSLFGLPPPPPGANPSEYYHLIASHRSPYGDL

LMQTGAAAAHLPDYMSPVDMSRFPSPRLTPRVSRKRALSISPVSDASIDLQTMIRTSPNS

LVAYINNSRSSSAASSSYGHLSVGAISPSFTFPHPITPVAYHQLLSQQRGLNAFGHTPPL

LQPSPALSSRQQTLVAAAALNNTTSSSTSSSTADTSTESSQNAGGDPAVSSTVNPMIFKR

SKVKTEAEGPHPISPGSQDHLELREELDKDECKQEPEAVYETNCHWEGCAKEYDTQDQLV

HHISNDHIHGEKKEFVCRWVECSREQKPFKAQYMLVVHMRRHTGEKPHKCTFEGCSKAYS

RLENLKTHLRSHTGEKPYVCEHEGCNKAFSNASDRAKHQNRTHSNEKPYVCKIPGCTKRY

TDPSSLRKHVKTVHGPEAHVTKKQRGDAPPKPHPPKGNGENEAHTKHVRGRTDGSGEANS

TTRGVEDCQHVKTIKTENTVMYQSSPGGQSSCSSEPSPLGSATNNDSGVEMAMHSGGSLG

DLSALDDTPVVDSTGSPGTSAGVGLQLRKNRAGLLQLEHIKKEKLKTVRDSCSWANAPPQ

VRNTKLPPIPSIDSLLDAPNMGTQMSVPPTQHLGDLSSYEMTMLNQLHERRDSSTSTMSS

AYISRRSSGISPCYSSRRSSEASQFGVRNNNVSSADSYDPISTDLSRRSSEASQCGGTGG

LPSLLNLTPAQHYSLKAKYAAATGGAPPTPLPNMDRMSLKTRMAMYNDSQDSSAHLHHAQ

GVVNSRRCSDTGYGAPGMMPHEVPANLPRRASDPVRRTTLDPLSLPRVQRFNSISNMNLS

RLPAYDRRAFNMQNNTWSDGSLHRHPFSQRPPSISENILMENMATDGGDQQGDDLVLPDD

MVQYLRSQNGNSNHDSGISVNGGHALEFHGNMTSQQQQFYGQRRMGMAGINGSHVEPVPE

QIADPQGMNKNNMPVQWNEVSSGSADAVSRVPKQQQQQLRGNLTVVQQKQNFGSYQGFGS

NQQIVPMSQNLASLQQAYPQRNIQRMNTVQQFRQSISNPCQNIGEQVNRQDLSYNSNQRL

ICNSMGPPNGRMVQNQELQNYRSNLMHMNNINQQNYVPLTQTAFQNAGRGLIQPRPPSEP

KPLNRQHSGSGMVQPNGCSLSNVNSSEASPKRPGEVGQHSNGNGTMYYSGEIHMLDNGID

YGSPMSPCTNQAPVASVSTMASPGVNQVTSTVDSTQSLDHTQIDFDAMLDDGDHSSLMSG

TLSPGLLQSISQNSSRLTTPRNSVTLASVPAGIGNMAIGDMSSMLTALAEESKFLNMMA

>Takifugu rubripes GLI2

MENSATTAAEKKECKSSGLDGSPSEIPKKLSPTTLSRGAHHLFPTFHTPIPIDVRHHEGR

YHYEPHPLHPMHGPPGLTGSPVISDISLIRLSPHAAPGSGESPFSPPHPYMSPHMEHYLR

SVHSSPTLSMISAARGLSPAEVSHEHLKERTLFSLPPPPPGANPTEYYHLMASQRSPYGD

LLMQSGEMGPHLFSKPFIGPLSAVSRFSSPRLTPRLSRKRALSISPLSDASIDLQTMIRT

SPNSLVAYINNSRSSSAASSSYGHLSVGPGISPSFGFPPHINPVAYQQLLSQQRGLSAFG

HTPPLIQPSPSSFSTRQHPLSASAMSAALNNSNSDTRGSFSSVCQSASGDPAVSSTVNPL

TTKRSKVKTEAEGLLPISPSSQDHGGGILDLSEDLDKDECKQEPEAVYETNCHWEGCTKE

YETQDQLVHHINNDHIHGEKKEFVCRWEECSREQKPFKAQYMLVVHMRRHTGEKPHKCTF

EGCSKAYSRLENLKTHLRSHTGEKPYLCEHEGCNKAFSNASDRAKHQNRTHSNEKPYVCK

IPGCTKRYTDPSSLRKHVKTVHGPEAHITKKQRSDVPPRPQPPKGNGVNEANSRHGAKGV

EGKIEANSTSGGMEDCLQIKSIKTENSMTYQSSPGGHSSCSSEPSPLGSTNNNDSGVEMA

MHSGGSFGDLSAQDECPMVDSTVPAGGQQAVMGLHLRKAVGHPGTVTIKLENIKKERLKT

VSTSDWVNSAAQPLQGQRSSVKLPPIPAVGSLLENSTLMSNSSISYPGQRRGDLSSCEVT

LLNQLNERRDSTTSTISSAYTISRRSSGISPGYSSRRSSEASQFGANRHNNISSADSYDP

ISTDLSRRSSEASQCGGGGLGGGATLPSLLSLTPAQHYRLKAKYAAATGGAPPTPLPNMD

RMSLRTRMALYSDSQEASLNQFHQPSPGTVPRRCSDMGYGTRSMMPHEVPGNLPRRASDP

VRRPTLDPLSLSRFQRYNSMNNMNPLNVSTAERYQSLVTQGYTRSDGNLQRYPFAPRPPS

ISENVAMENMVMDGMTGADQNGEDDLVLPDDMVQYLRSQNTGPSANNLGQVDFHSNHQNH

GFQTGMAPPPSYYSQRRMAMVDATMTHSAQDSQQPFSAPPENMNKNNMPVQWNEVSSGTV

DSTAKLSKPQHHPLRGNLAVVQQRHNFSSFQGQGQALSSNHQVVPMSQNVSMQYVPVNFG

EHMSPQQGFGQEFIPNSISGSTSMRPAQNVVASSELQRGRTQTDGGSLQPRPPAEPKSNM

RQHPGSNMMHPNRVAKSSALSPCDGTETSEASPKRRSGPAAHNSSSENSVFYTGQIHMFE

PSFDAPMSPCASEPPANSTSAANMASPGVNLVSSSTIDSSTGVSGGPQHPQIDFDTMLDD

GDHSSLMSGTLSPGLLQSLSQSSSRLTTPRNSLPLASVPAGIGNMAIGDMNSMLTALAEE

SKFLNMMS

>Homo sapiens GLI3

MEAQSHSSTTTEKKKVENSIVKCSTRTDVSEKAVASSTTSNEDESPGQTYHRERRNAITM

QPQNVQGLSKVSEEPSTSSDERASLIKKEIHGSLPHVAEPSVPYRGTVFAMDPRNGYMEP

HYHPPHLFPAFHPPVPIDARHHEGRYHYDPSPIPPLHMTSALSSSPTYPDLPFIRISPHR

NPTAASESPFSPPHPYINPYMDYIRSLHSSPSLSMISATRGLSPTDAPHAGVSPAEYYHQ

MALLTGQRSPYADIIPSAATAGTGAIHMEYLHAMDSTRFSSPRLSARPSRKRTLSISPLS

DHSFDLQTMIRTSPNSLVTILNNSRSSSSASGSYGHLSASAISPALSFTYSSAPVSLHMH

QQILSRQQSLGSAFGHSPPLIHPAPTFPTQRPIPGIPTVLNPVQVSSGPSESSQNKPTSE

SAVSSTGDPMHNKRSKIKPDEDLPSPGARGQQEQPEGTTLVKEEGDKDESKQEPEVIYET

NCHWEGCAREFDTQEQLVHHINNDHIHGEKKEFVCRWLDCSREQKPFKAQYMLVVHMRRH

TGEKPHKCTFEGCTKAYSRLENLKTHLRSHTGEKPYVCEHEGCNKAFSNASDRAKHQNRT

HSNEKPYVCKIPGCTKRYTDPSSLRKHVKTVHGPEAHVTKKQRGDIHPRPPPPRDSGSHS

QSRSPGRPTQGALGEQQDLSNTTSKREECLQVKTVKAEKPMTSQPSPGGQSSCSSQQSPI

SNYSNSGLELPLTDGGSIGDLSAIDETPIMDSTISTATTALALQARRNPAGTKWMEHVKL

ERLKQVNGMFPRLNPILPPKAPAVSPLIGNGTQSNNTCSLGGPMTLLPGRSDLSGVDVTM

LNMLNRRDSSASTISSAYLSSRRSSGISPCFSSRRSSEASQAEGRPQNVSVADSYDPIST

DASRRSSEASQSDGLPSLLSLTPAQQYRLKAKYAAATGGPPPTPLPNMERMSLKTRLALL

GDALEPGVALPPVHAPRRCSDGGAHGYGRRHLQPHDAPGHGVRRASDPVRTGSEGLALPR

VPRFSSLSSCNPPAMATSAEKRSLVLQNYTRPEGGQSRNFHSSPCPPSITENVTLESLTM

DADANLNDEDFLPDDVVQYLNSQNQAGYEQHFPSALPDDSKVPHGPGDFDAPGLPDSHAG

QQFHALEQPCPEGSKTDLPIQWNEVSSGSADLSSSKLKCGPRPAVPQTRAFGFCNGMVVH

PQNPLRSGPAGGYQTLGENSNPYGGPEHLMLHNSPGSGTSGNAFHEQPCKAPQYGNCLNR

QPVAPGALDGACGAGIQASKLKSTPMQGSGGQLNFGLPVAPNESAGSMVNGMQNQDPVGQ

GYLAHQLLGDSMQHPGAGRPGQQMLGQISATSHINIYQGPESCLPGAHGMGSQPSSLAVV

RGYQPCASFGGSRRQAMPRDSLALQSGQLSDTSQTCRVNGIKMEMKGQPHPLCSNLQNYS

GQFYDQTVGFSQQDTKAGSFSISDASCLLQGTSAKNSELLSPGANQVTSTVDSLDSHDLE

GVQIDFDAIIDDGDHSSLMSGALSPSIIQNLSHSSSRLTTPRASLPFPAVHEHHQHGYRG

HEFFADLPSGRKQIPCSYAIGFRKKRLQPTEINRS

>Mus musculus GLI3

MEAQAHSSTATERKKAENSIGKCPTRTDVSEKAVASSTTSNEDESPGQIYHRERRNAITM

QPQSVQGLNKISEEPSTSSDERASLIKKEIHGSLPHLAEPSLPYRGTVFAMDPRNGYMEP

HYHPPHLFPAFHPPVPIDARHHEGRYHYDPSPIPPLHVPSALSSSPTYPDLPFIRISPHR

NPTAASESPFSPPHPYINPYMDYIRSLHCSPSLSMISAARGLSPTDAPHAGVSPAEYYHQ

MALLTGQRSPYADILPSAATAGAGAIHMEYLHAMDSTRFPSPRLSARPSRKRTLSISPLS

DHSFDLQTMIRTSPNSLVTILNNSRSSSSASGSYGHLSASAISPALSFTYPSAPVSLHMH

QQILSRQQSLGSAFGHSPPLIHPAPTFPTQRPIPGIPTVLNPVQVSSGPSESSQSKPTSE

SAVSSTGGPMHNKRSKIKPDEDLPSPGSRGQQEQPEGTTLVKEEADKDESKQEPEVIYET

NCHWEGCTREFDTQDQLVHHINNDHIHGEKKEFVCRWLDCSREQKPFKAQYMLVVHMRRH

TGEKPHKCTFEGCTKAYSRLENLKTHLRSHTGEKPYVCEHEGCNKAFSNASDRAKHQNRT

HSNEKPYVCKIPGCTKRYTDPSSLRKHVKTVHGPEAHVTKKQRGDMHPRPPPPRDSGSHS

QSRSPGRPTQGAFGEQKELSNTTSKREECLQVKTVKAEKPMTSQPSPGGQSSCSSQQSPI

SNYSNSGLELPLTDGGSVADLSAIDETPIMDSTISTATTALALQARRNPAGTKWMEHIKL

ERLKQVNGMFPRLNPILPSKAPAVSPLIGNGTQSNNNYSSGGPGTLLPSRSDLSGVDFTV

LNTLNRRDSNTSTISSAYLSSRRSSGISPCFSSRRSSEASQAEGRPQNVSVADSYDPIST

DASRRSSEASQGDGLPSLLSLTPVQQYALKAKYAAPTGGPPPTPLPHMERLSLKTKMALL

GEGRDSGVTLPPVHPPRRCSDGGGHTYRGRHLMPHDALANSVRRDSDPVRTVSENMSLAR

VQRFSSLNSFNPPNLPPSVEKRSLVLQNYTRQESSQPRYFQASPCPPSITENVALEALTM

DADANLNDEDLLPDDVVQYLNSQNQTGYGQQLQSGISEDSKVAHEPEDLDLAGLPDSHVG

QEYPALEQPCSEGSKTDLPIQWNEVSSGTSDLSSSKLKCGQQRPRQQPRGFGLYNNMVVH

PHNLWKVGTGPAGGYQTLGENSSTYNGPEHFAIHSGDGLGTNGNTFHEQPFKTQQYGSQL

NRQPLTSSALDHACGTGIQGSKLKGNSLQENGGLLDFSLSVAPNELAGNTVNGMQTQDQM

GQGYIAHQLLSGSMQHQGPSRPGQQVLGQVGATSHINIYQGTESCLPGTQDNSSQPSSMA

AIRGYQPCASYGGNRRQAMPRGNLTLQQGQLSDMSQSSRVNSIKMEAQGQSQQLCSTVQN

YSGQFYDQTMGFSQQDRKAGSFSLSDANCLLQGTCTENSELLSPGANQVTSTVDSFESHD

LEGVQIDFDAIIDDGDHTSLMSGALSPSIIQNLSHSSSRLTTPRASLPFPIPIHGHHQHG

YRGYEFFADLPCRRKQVPCSYAVGGRQGGPQTQRLK

>Rattus norvegicus GLI3

HINNDHIHGEKKEFVCRWLDCSREQKPFKAQYMLVVHMRRHTGEKPHKCTFEGCTKAYSR

LENLKTHLRSHTGEKPYVCEHEGCNKAFSNASDRAKHQNRTHSNEKPYVCKIPGCTKRYT

DPSSLRKHVKTVHGPEAHVTKKQRGDMHPRPPPPRDSGSHSQSRSPGRPTQGAFGEQKEL

SNTTSKREECLQVKTVKAEKPMTSQPSPGGQSSCSSQQSPISNYSNSGLELPLTDGGSIA

DLSAIDETPIMDSTISTATTALALQGRRNPAGTKWMEHIKLERLKQVNGMFPRLNPILPS

KAPAVSPLIGNGTQSNNNYSSGGPGTLLPSRSDLSGVDFTVLNTLNRRDSNTSTISSAYL

SSRRSSGISPCFSSRRSSEASQAEGRPQNVSVADSYDPISTDASRRSSEASQGDGLPSLL

SLTPVQQYRLKAKYAAATGGPPPTPLPHMEKLSLKTRMALLGEGRDSGVTLPPVHPPRRC

SDGGGHTYSRRHLLPHDALANSARRASDPVRTVSENMSLPRVQRFSSLNSFNPPNLPPSV

EKRSLVLQNYTRQESTHPRYFQASPCPPSITENVALEALTMDADASLNDEDFLPDDVVQY

LNSQNQTGYGQQLQSSISEDGKVAREPEDLDLPGLPDSHVGQQYPALEQPCSEGSKTDLP

IQWNEVSSGSSDLSSSKLKCGQRPTVQQARGFGLYSNMVVHPQNLWKVGTGPAGGSQTLG

ENGSSYSDPEHFAVHSGDGLGPSGDTFHEQAYKTQQYGSQLSRQPLTSSVLDSACGAGIQ

GSKLKGNSLQENGGLLDFGLSMAPNELADNIVNGIQTQEQMGQGYIAPQLLSGSMQHQGP

SRPGQQVLGQVGATSHINIYQGTESCLPGTQDKISQPSSMAVIRGYQPCASYGGSRRQAM

PRGSLTLQQGQLSDVSQTSRVNSIKMEAHGQSHQLCSSMQNYSGQFYDQTMGFSQQDRKA

GSFSLSEANCLLQENGSENSELLSPGVNQVTSTVDSFESHDLEGVQIDFDAIIDDGDHTS

LMSGALSPSIIQNLSHSSSRLTTPRASLPFPSLSMSTTNMAIGDMSSLLTSLAEESKFLA

VMQ

>Gasterosteus aculeatus GLI3

PGLAGSPAFSDISLIRISPQRNPSVGAESPFHPPHPYINPYMDYIRSLHSSPSISVLSA

TRGLSPADVISSSFLQKRLLYSAPHTGLTTAEYYHQMALLAGHRSPYATDLLPSVASTAG

ATSATALHMEYLQAMENSRFSSPRLPSRPSRKRPLPISPLSDHSFDLQTRIRHSPNSLVT

MLNNSRSSSSTSGSYGHLSAGAISPALSFAYPSTPVALHMHQQLLGRQPGMVGSAFGHSP

PLIHPSPAYATQRPVPGIPPSGLSASERSALSSDSAQTKPTSESAVSSTGDPMHHKRSKM

KPEEELPSPGAVSVQSLGRRGGNCFCFCLIKIDHPDGMTLVKEEGDKDESKQEPEVVYET

NCHWENCCREFDTQEQLVQHINNDHIHGEKKEFVCRWEECSREQKPFKAQYMLVVHMRRH

TGEKPHKCTFEGCAKAYSRLENLKTHLRSHTGEKPYVCEHEGCNKAFSNASDRAKHQNRT

HSNEKPYVCKIQGCTKRYTDPSSLRKHVKTVHGPEAHVTKKQRGDLPLGGYTDQREYNHA

TSKQDECLQVKSIKTEKPMTSQPSPGGQSTCSSDQSPVSHYPCSGVQLAVSAGRSLGEGH

CVGERAPIMDSTVSTATAAMLTLQARRSVGRPLRWMEHMKMERLKQVNGALPRLGPLSPT

PPPKGSTLPNILGKENSNITTDLCLNPAVRNKLSPDSIFPVWLLERSGVKTDVVWVLISS

YCVKLNSSGISPCFSSRRSSQASQSEGTAAAAHHHRRLHNLSSSDSYDPISTDASRRSSE

ASHGSRGMLTLTPAQHYHLKAKYAAATGGPPPTPLPNMERMSLKTRMAMMDEGGSNQSLP

PLVRPHCCGDGANGYANGYAGHAGHRRRVLYPGEGPLNGNRRASDPVRTQAGDMGSLPPV

QRFNSLNNLHPLPPLSHHSTPEARSFSLQSYTRSEGNLQRGLQHSPYTPSIAEHAALEAL

AMEDEAGLLLGDEDMLPDDLVQYLHSQAQVDGGSYMHIEEQIASSQRDASHPPMEEMGQI

QAAGLDMGHAGSHGLLQQQQQTAERTSPSKLPIQWNEVSSGSADRSPQREQIHRAQCGRW

PAAEHGPVPFGRFGNMVVQQQVPLDFQNSCVQANQSQGACFVNPAVKLETPPNSCMEMRG

FGRPNFAQIIDGPLQQGFKHPPPGGPPKQSHFQQNHSQMGSNLILSRSSVDILPSVSQGA

AAHHNRQIHVPRPPDGYRNSARIQQYLNAETSGESQAGLNKQQQLQRSGDHCSLAHQVSG

LKLEASDHGYLEQGFGDCLSYAPLDCKASPFPVLEDQCLLNSMVESAGQVVGGGGGGGNS

AALLSPGTDQVTSTMEGAVPGILDDGVGLDFGAMLEDGYDQGSLVSGVLSPSIFQGLSRT

SSRLTTPRASAAFHSVAPGLNNMAIGDMSSLLTTLAEESKFLAILQ

>Tetraodon nigroviridis GLI3

EDDSSGTPFHRERRNAISSHAPTPGGLDRSISEEPSTSTEERPSLLKKELHGSLAHLGDH

ALPYRGTLFAMDPRNGYLDSHYLRATLRCRQPVSAGGFLVCLLPKWHPHRYVSAAPQFFP

TFHPPVPIDDRHTQGRYIYEPSPVPPLHVFWYPASHRERIRPLSAPRDEGHFFILLSNCP

KRIRWNFGGRRPPALAGSPAFSDISLIRISPQRNPSVGAESPFHPPHPYISPYMDYIRSL

HSSPSISVLSATRGLSPADAPHTGLTTAEYYHQMALLAGHRSPYAADLLPSLAATAGASG

AAASHVEYLHAMESSRFSSPRLPSRPSRKRPLPISPLSEPSFDLQTMIRNSPNSLVNILN

NSRSSSSTSGSYGHLSAGAISPALSFAYPPTPVALHVHQQLMGRQPSIVGSAFGHSPPLI

HPSPAFTTQRPVPGIPASGISASERSAISSDSSQTKPTSESAVSSTGDPMHHKRSKMKPE

EELPSPGAVSIQDHPDGMTLVKEEGDKDENKQEPEVVYETNCHWENCCREFDTQEQLVQV

RHKRQVQRRLVGDVGGAALSLFSDEELALHAEAAAHINNDHIHGEKKEFVCRWEDCSREQ

KPFKAQYMLVVHMRRHTGEKPHKCTFEGCAKAYSRLENLKTHLRSHTGEKPYVCEHEGCN

KAFSNASDRAKHQNRTHSNEKPYVCKIPGCTKRYTDPSSLRKHVKTVHGPEAHVTKKQRG

DYPRPPPQPREPGSNGQGRSPGQLPLGGYTDQREYTHSTSKPEECLQVKSIKTEKPMTSQ

PSPGGQSTCSSDQSPVSTHPYGRVQLAVSAGRSPGEGLEESEEKEENEEADEECEEGEQA

PIMDSTVSTASTAMLALQARRSVGRSLRWMEHVKMERLKQVNEALPRLGPLSPTPPPKVS

TLPTILGKGSCLGRQWGVTAPQPPAHGELGSTELTVLNLLRDRRDSSGSATSSAYLSSSR

RSSGISPCFSSRRSSQASQNEATMAAVYHRRLHNLSSTDSYDPISTDASRRSSEASHCGG

GGGGMLSAGGCGGAFGLGVGGGGGSRGMLSLTPAQHYHLKAKYAAATGGPPPTPLPNMER

MMGDALVRPHRCADGSPGSVNGHTGHSGYRRRVLYPGEGSANRNRRASDPVRSQAPDACS

LPPVQRFSSLNNLHPLPPLSHHSIPENCSLSLQNSARSEGNMQRILQHSAYNPSIAEHAA

LEALAMEEDGGAGLLLGDEDMLPDDLVQYLHSQVQSDSCAFMNVEEQLASSQRDPSYPSM

EELEQIQSSGLDMSYSGSHGLQQQIAERRSPSSLPIQWNEVSSGSADRS

>Takifugu rubripes GLI3

METQSQASSAAEKKKKVETIAATKGLVARNDISEKAVASSTTSNEDDSSGTPFHRERRNA

ISSHAPTASGLDRSISEEPSTSTDDRPSLLKKEQHSSLPHLADHALPYRGTLFAMDPRNG

YLDSHYAAPQFFPTFHPPVPIDDRHTQGRYIYDPSPVPPLHVPPALAGSPAFSDISLIRI

SPQRNPSVGAESPFHPPHPYISPYMDYIRSLHSSPSISVLSATRGLSPADAPHAGLTTAE

YYHQMALLAGHRSPYATDLLPSLAATASASGAVAPHVEYLHAMESSRFSSPRLPSRPSRK

RPLPISPLSEHSFDLQTMIRNSPNSLVNILNNSRSSSSTSGSYGHLSAGAISPALSFAYP

PTPVALHVHQQLIGRQPSIVGSAFGHSPPLIHPSPAFATQRPVPGIPPPGISASERSAIS

SDSSQNKPTSESAVSSTGDPMHHKRSKLKPEEELPSPGAVSIQDHPDGMTLVKEEGDKDE

SKQEPEVVYETNCHWENCCREFDTQEQLVQHINNDHIHGEKKEFVCRWEDCSREQKPFKA

QYMLVVHMRRHTGEKPHKCTFEGCAKAYSRLENLKTHLRSHTGEKPYVCEHEGCNKAFSN

ASDRAKHQNRTHSNEKPYVCKIPGCTKRYTDPSSLRKHVKTVHGPEAHVTKKQRGDYPRP

PPQPREPGTNGQGRSPGQLPLGGYTDQREYNHSTSKQEECLQVKSIKTEKPMTSQPSPGG

QSTCSSDQSPVSTYPYSRVQLAPIMDSTVSTASTAMLTLQARRGVGRPLRWMEHVKMERL

KQVNEALPRLGPLSPTPPPKVATLPNILGKGSCLGRQWGVTAPQPPINGELGSTELTVLN

LLRDRRDSSGSAASSAYLSSSRRSSGISPCFSSRRSSQASQNEATTAAAHHRRLHNLSST

DSYDPISTDASRRSSEASHCGGGAGGGGGGPLSGGCGGVFGLGVGGGGGSRGMLSLTPAQ

HYHLKAKYAAATGGPPPTPLPSMERVMDDGGSNQTLPPLVRPHRCSESSWGNTGGYVGHS

GSRRRVLYPGEGALNRNRRASDPVRTQAPDACGLPPVQRFSSLNNLHPLPPLAHHSIPEN

CSLSLQSSTRSEGNMQRFLQHSTYNPSIAEHAALEALAMEEDGGSGFLLGDEDMLPDDLV

QYLHSQVQSDSLSLLNIEDQLASSQRDTSYPSMEEFEQIQSSGLDMSYPGSHSLQHQIAE

QKSPSSLPIQWNEVSSGSADKSPHRKQNQQTAYGRWTGSGNGCASAPFGRFQNMVVQQQV

PLEFQNNCPNASQPQGACSVNPAVKLETLPNSCMELRGTRGTFGRSTFPQGLDGPLPQVF

KQQPANGAPRQSHSQLKHHSTTPSQDGADPGSSLNNLSGLSHGAALSLHRQVHVPHPPNS

YRTFARAQQGHNSSNMLTNTKQQLMRRNGDHCSLAHQVSGLKLEAPDQGYSESGYGQSLS

YGEGDCKASSFLALKDQCLLGSITEPLGQSEGINGTAPVALLSPGADQVTSTVDSLGPPL

DQGTSLDFGAILEDGFDQGSLGTSSRLTTPRASAAFHSVAPGLNNMAIGDMSSLLTTLAE

ESKFLAIMQ

>Danio rerio GLI3

MEPQFQTSSSTEKRMENVVGTNSAPTRTDISEKAVASSTTSNEDDSSGSPYHLERRNTIS

AQTDVVNTSGKVSEEPSTSTDERLPLVKKELQSFLHRLADHTLPYRGTLFAMDLRSGYLD

PHYTAPQFFRAFHHPVPIDDRHTQGRYIYEPSPVPSLHMPPALAASPTFSDISLIRISPH

RNPSMGTDSPFNPSHPYINPYMDYIRSLHSSPSLSMVSAARGLSPADTHHTGLTTAEYYH

QMALLVGHRSPYADLIPSVGSTTGPASSALHMEYLQAMESSRFPSPRLINRPSRKRPIPN

SPSLSDPSFDLQAMIRTSPNSLVTILNNSRSSSSNSGSYGHLSAGTISPALSFAYPPTPV

ALQMHQQLISRQPGIVGSAFGHSPPLMHSALAFSTQRPVPAIQTSSLAPTERSIVSNDSQ

SKPTSESAVSSTGDPMNHKRSKLKPEEEPPSPRAVSMQEQMNGMTLVKEEGDKEEGKQEP

EVVYETNCHWDGCCREFDTQEQLVHHINNDHIHGEKKEFVCRWEECSREQKPFKAQYMLV

VHMRRHTGEKPHKCTFEGCSKAYSRLENLKTHLRSHTGEKPYVCEHEGCNKAFSNASDRA

KHQNRTHSNEKPYVCKIPGCTKRYTDPSSLRKHVKTVHGPEAHVTKKQRGETYPRPPPQP

REPGATTDQKEYTHATSKQDECLQVKSIKTEKPMTSQSSPGSQSSCSSDQSINGPHVSQG

VQLTVNSIGLAGEVAMLEDMENEEEDEENREGEKEDAPIMDSTVSTATSSAATIVLQTHR

SVGRPLRWMEHVKMERLRQVNGAVPRLGFLPPTPPRKGQAMPALTGKGQCLGRMTSVRVP

PCPEPSNTELTILSLLHERRDSSGSNTSSAYLSSSRRSSGISPCFSSRCSSQASLSEYSP

HRHIHNLSATDSYDPISTDASRRSSEASQYDGGSSSAVVFGGSGVFKGSGMGSRGDLNLT

PAQHYSLKAKYAAATGGPPPTPLPSMGHINLKTHFALKEDRKDTGQLTLPPLVRSRRCSD

GAHNSHDSQAVYQRKGTYFGGGPGKNDRRASDPTHHKQNLSSYGLPQVRHFSSLNNMTSS

PNPASYGYSNREDHSLNLQNSTCHVDSFQSGLHSPCPPSIMEQSALENIFMNQDGLLLRV

NENIQSDFMQYLHSQDQESIIIQDNHRPIFNPADDQNFLSPQQQQEGVEQEDTRIDLMPI

QSNEVSSASADVTIPRQPFQRCGRWSDQSGSVSNAFGRFGNMVVQQQPIDFIDQHSALQH

MGGISYGCRNSEQHLQVAKPAQEKSRMKTGIKTESSSTFQLESKHSNFGRPDFSHIFSAP

IHQKCSQHTVVSQSYPQQNQITNQDSSNLFLQRPGNCSLLLAYRPQPAVNTSYSTRQDIN

SHNVQSCHPQSLNNRLGTSCSLSNQVSVVKLDQVNHLQINSCSTIQQQKPVFIDKCFDPE

ESKLSHAIVQSCLMESLMAEKERTSKVLLSSGVGQVTSKVERSPAEKVGLGICSIGGDSY

DQGSVVSSSRLSNSHDSFAFHSVDPGTNNMAISDMNSLLTTLAEESKFLAIIQ

>Branchiostoma floridae GLIA

MASTSPGVTKEPERRPAESSESKSDKSRHHGEAKSAETSIKKEAASSSEYAHPRLEPSRPGFPHPPLISE

HRSTYPYPSFGLDPRNGYVDPHFMSPVMPFHQPIPVDARTNEGRYTFDPHNVYHVPIHSPPFGGSPVLSD

VSMLRLTPHRPHSDPTSPLHGPHPAFRLNPFFSPYHGTPPLSALSQARGLSPHDGMTAAEYYHMAAMSQQ

RMYSDMPPLSGGSGSIAIDLPTDGSRFSTPRTAARHGRKRALSISPLSGECLDLNSMIRTSPNSLVAFIN

GGSRSGSSASTGSYGHLSASAMRTESGAESKPGTQSSANMPFSAPMPMFPYPTSPAMQQFHNRLMRQKSP

FHFGMPHASPFAAPLPAGMAMLAAQGAMPPSSSAATHTETKASLGDAFTMEPSESKRSKVKYEGSQGSPD

DVPSTTFPAESRSETSMAEGTNEDAEPPVYETNCHWDGCSKEFDTQDQLVHHINNDHIHGEKKEFVCRWS

ECTREQKPFKAQYMLVVHMRRHTGEKPHKCTEIMPQISLRTLPVLPFEGCNKAYSRLENLKTHLRSHTGE

KPYVCEHEGCNKAFSNASDRAKHQNRTHSNAKPYVCKIPGCTKRYTDPSSLRKHVKTVHGPEAHQTKKHK

TLGPTPRPRDPPSEKRDQDSVSSPPDSNGVHSSTTNPAASQGSPGQKPTEGHKPTGQTCDAQQSVYGSSP

HHDSGVEMNANSGSLPDLSTLDDQVISDSSISSTVPTSRASGVMVAARPGLVPRAPRIGNKPSNQRRRMR

LSSGTPGPTSPPRSDSVQLPPIEKTGSRGPSAQGSHSSVEAANRRTNELRASDLSQTSRTSSLGSLGSRK

DSASTVSSYYSSRRSSEASPFPESIFSSRRSSQASPFPGINRRTSNGSLYSPNDSYDPISLGSSRKSSDA

SSLSMNVNELGINIEQQQMLRARFIQATGRPPTAVCGNDSRPESRRGDRKEKENVEEPNPRRQSDLGHYN

RLKGTPLPKEVKDGPHRRSSAPQKNDVVTNLPDVPRDHSFNKHTPLPPVTPQPPPQIKKAFSPSKVKQAF

SPKSASTSMQGVAEEFPMDLIENEPDVIIPDEMVQFLNSQTGDDPREMVPNFEQVGTTPTFVEDIPPMQV

NPIQGDGFSNMGSPQQAFSPNRQPMPPIQQQQAFNQSQQVLSPSRQSYKMSQPMGSQPLSPMEVNMAALD

HSPNPPTYNASQMSPAQQFSPASYDPNLNSPSCRVQQNYSQMGMQSPVPNYASPTRQAQNMAPKLPNMQP

QMLSPQQRQQSQQQQLQQQMQSQQQQMQQMQKLQQMQKMQQMQKMQQMQKMQQMQKMQQMQKMQQVQKMQ

QMQQQQQQQQQAQMQNMQVQRQMQNMQKQSQLQQQMQNLQQQQRLQQQNLQQNYNMQQMQNLQSQMQLSP

NVQQQMQNLQLSPSGQQYLPEGAQQMQYQANVQFYNLQNQSGRPLENPTQVNVVHSTIEPHHSPQRPDGA

ADINLENVAMPTQNPMGFPAGNAGYTQDQSTNFVGGVNMSTPMVPPETPGSASNMVINDMSSLMASLAEE

NRYLNMMG

>Branchiostoma floridae GLIB

MASTSPGVTKEPERRPAESSESKSDKSRHHGEAKSAETSIKKEAASSSEYAHPRLEPSRPGFPHPPLISE

HRSTYPYPSFGLDPRNGYVDPHFMSPVMPFHQPIPVDARTNEGRYTFDPHNVYHVPIHSPPFGGSPVLSD

VSMLRLTPHRPHSDPTSPLHGPHPAFRLNPFFSPYHGTPPLSALSQARGLSPHDGMTAAEYYHMAAMSQQ

RMYSDMPPLSGGSGSIAIDLPTDGSRFSTPRTAARHGRKRALSISPLSGECLDLNSMIRTSPNSLVAFIN

GGSRSGSSASTGSYGHLSASAMRTESGAESKPGTQSSANMPFSAPMPMFPYPTSPAMQQFHNRLMRQKSP

FHFGMPHASPFAAPLPAGMAMLAAQGAMPPSSSAATHTETKAGEPSSSIVSSTMEPSESKRSKVKYEGSQ

GSPDDVPSTTFPAESRSETSMAEGTNEDAEPPVYETNCHWDGCSKEFDTQDQLVHHINNDHIHGEKKEFV

CRWSECTREQKPFKAQYMLVVHMRRHTGEKPHKCTEIMPQISLRTLPVLPFEGCNKAYSRLENLKTHLRS

HTGEKPYVCEHEGCNKAFSNASDRAKHQNRTHSNAKPYVCKIPGCTKRYTDPSSLRKHVKTVHGPEAHQT

KKHKTLGPTPRPRDPPSEKRDQDSVSSPPDSNGVHSSTTNPAASQGSPGQKPTEGHKPTGQTCDAQQSVY

GSSPHHDSGVEMNANSGSLPDLSTLDDQVISDSSISSTVPTSRASGVMVAARPGLVPRAPRIGNKPSNQR

RRMRLSSGTPGPTSPPRSDSIQLPPIEKTGSRGPSAQGSHSSVEAANRRTNELRASDLSQTSRTSSLGSL

GSRKDSASTVSSYYSSRRSSEASPFPESIFSSRRSSQASPFPGINRRTSNGSLYSPNDSYDPISLGSSRK

SSDASSLSMNVNELGINIEQQQMLRARFIQATGRPPTAVCGNDSRPESRRGDRKEKENVEEPNPRRQSDL

GHYNRLKGTPLPKEVKDGPHRRSSAPQKNDVVTNLPDVPRDHSFNKHTPLPPVTPQPPPQIKKAFSPSKV

KQAFSPKSASTSMQGVAEEFPMDLIENEPDVIIPDEMVQFLNSQTGDDPREMVPNFEQVRTTPTFVEDIP

PMPVNPIQGDGFSNMGSPQQAFSPNRQPMPPIQQQQQAFNQSQQVLSPSRQSYKMSQPMGSQPLSPMEVS

MAALDHSPNPPTYNASQMSPAQQFSPASYDPNLNSPSCRVQQNYSQMGMQSPVPNYASPTRQAQNMAPKL

PNMQPQMMSPQQRQQSQQQQLQQQMQSMQSQQQQMQQMQKLQQMQKMQQMQKMQQMQKMQQMQKMQQMQK

MQQVQKMQQLQQQQQQQAQMQNMQVQRQMQNMQKQSQLQQQMQNLQQQQRLQQQNLQQNYNMQQMQNLQS

QMQLSPNVQQQMQNLQLSPSGQQYLPEGAQQMQYQANVQFYNMQNQSGRPLENPTQVNVVHSTIEPHHSP

QRPDGAADINLENVALPTQNPMGFPAGNAGYTQDQSTNFVGGVNMSTPMVPPETPGSASNMVINDMSSLM

ASLAEENRYLNMMG

>Ciona savignyi GLI

METAGKNDIDVLHQKCGKDFSREEKATPQTHLPPDHKIEGSEHHSRRSEAPKPGKQSAFF

SSQDENEAREEAVVSSSTSPSDKRTKRSSSPDHNEHLRQQNDELRHQSRHQSVREAATST

TTFEDEFEKQFHGNENLGVTSSDGRPPSCGTPLGKIKEETSSNNRKVMLLGNAHQFTKPS

PSNRNMTSSQAGNTNCNGEDRRYPIGNPHTQVDPHSRDGRYPFEPRTMNNAPGFSGSPIH

SDISLIPMSPQQSANPFNYNSSLEFYMGGLSQGGLRSPTMSILSQARNLSPHHSIQELEK

LCYPYYNAHQSNMSVNGAQQNSNQSLPGNFSQNINPLAYYQLMQQQQRLLAVAARNDRNL

ALSLEKQEDFGRFPSPLCRSRARKRPLSISPCFSDTGLDITAMIRTSPNSLLPFGGLTNS

RSSSVASGGSYGHLAAGGISPTFGYQTPMMTSPQHLHAHLLSRQMQSSFAGQLMPGFPTN

QGAFDKPNSRFDPNISTRNYLPHVGASAASNDMCNPHRDNKNFSKHSVDPQSPNVQFNNN

NNNSNSIPGISPGGHGANEENDVVYDTNCSWVNCSLEFDTQEQLVHHINNDHIHGDKKEF

VCRWIGCARDQRPFKAQYMLVVHMRRHTGEKPHRCTFEGCSKAYSRLENLKTHLRSHTGE

KPYVCEYPSCTKAFSNASDRAKHQNRTHSNEKPYACKQAGCTKRYTDPSSLRKHVKTVHG

PEAHVTKRMKAEREREGKREPNIKQEPGMDKDKRKGDAGGTKRTCHPLSPGSNPPSNDQN

PPSNMNPRSNQSSCTSGSDVSVTPQNSPHMTSQNNDSGVDVNVGANEGDSDGDIVVDENP

QPDSTSGGVGVQSRHRGTVRASLVPRLVNKKMQNLSIGGNIESYVDIGGYDDQRKLGMHK

AESFTEVSSTTAFPAKQKNATFPRKLPLTPHRQVALLNQDRRDSGTVSDGSRKSSLASQN

SRRSSQNTGGINVAGSYDPISLDSSRRSSSNVTSGSSALNPYQLHRLRSRFNENAGLPPP

TPVDREGYTKSQLSRWLKDDTPIPDRGGYLYNPQQRPSLPQMGPPKPPMEDRRRSEPRSS

ARTPLPPHLGGGVAFRRASDPTRKSPQMDPTRLPNVQRHNSWNSIHPLPGIKPQGCAYPG

AVPCNPSNQPPMQQVQRQRSISNSSNYYGGESGYHSHGSNMALHQPSYQVNEQVLMENFN

NDVINGDVTYNGQVYGKSDHWHQQTTPHPHQTMTPASVGSMPPHQRFPQNGHINQNPFPR

SFNNNPNVGPNKGVQDANATRYYGNPHFNPNAQMGGQVHGHPTMRPQKMTQQNVTQQFGN

PDFMQLPYNAPSPYIAQRQSVQNQGRSPNMINSGNVEMHLGHQNRTANQITPRPPVSSTS

SNQNRMRLQRVRQSGSSSVTSYNQAPSVTSQSNGQAGRTNYTPDAQIPDEFLQQEFIDSI

PELHPETHRENEPNENQPYVKMAADSTTGPDLDGITGNFCYQDDDAMSGTNTDVTLPPMS

AFSSRMATPMMGKYPPQIPTNQHPASNMAINSMSSMLSTLEEENKYLNLLPQ

>Strongylocentrotus purpuratus (XP_001195249)

MSSVSKQVTEPNNNQVTSSVDLDNHQSKGMDDPNTVSSTSHMEGSPSAPAPGGDHRDDDGAHGGEEDIPV

VTDCEWDDCHRKFDTLDQLVQYDNHLFFFLLKFVCNLLQHINNDHIHNERKEFICHWRDCIREEKPFKAQ

YMLVVHMRRHTGEKPHKCSFEGCYKAYSRLENLKTHLRSHTGERPYVCEFQGCTKAFSNASDRAKHQNRT

HSNAKPYACKITGCTKRYTDPSSLRKHVKTVHGPEAHVTKRQRGDKRDPPSGSGGEKEGRKGDDMDKKSP

GDVKPITKAISVSSNSTNNNRTKGVPQECHTSAHQSVHLSAPVNDYSSSPHNDSGVEMNATGGSIGDLTS

IDDEKLIQDDQISSNVGDLGDGIGHRGRMGRRGGGGGRGSLTVTTAPTIIGRTTSPFMERSIKIKAGMSR

PTHKAKTGVHILPQMPPPPPPPLQQQLPPVINNNNHGIDYKPKARNIEKLIFEERCDSSQSMTGNLDNLG

NRRGSDHSTVSSYFSGRSSEASPFFMGSQFSSRRSSQASSYMSSMSSRRTSGASQWSGHMGMNSPYDPIS

IGSSRRSSEASSLNGPTGLPGLTLAQQRSLQARYEQTIMQQNSGDERFGMTPSPMPPPGYRRLSGVASMP

PHPPRTPLPNEIPGNEIRRGSEPISSNQNSHMNRRYSSFNNKQPLPLPENMKNFRPAFHNIHHPPTIHEQ

DGGSGREQWQNIPQNHYEHGTIPEESMDTGGSGGPEEFMFPDEMVQFLDDQCKQEMQWTGQRQMPAGGGG

GDGGRGFNQPHGNNHPGHNQMQGGYNMQPLNSDALDHVQQIVPETMESETENKMPYNSQGASAGYPQQEN

VVNPTISNMAVTDMSSMLTSLAEENRFLSIM

>Drosophila melanogaster (Ci)

MDAYALPTYFPLAYSELQFLASRRAAAVAAAATVLPGSPCINQHHPTDVSSSVTVPSIIP

TGGTSDSIKTSIQPQICNENTLLGNAGHQHNHQPQHVHNINVTGQPHDFHPAYRIPGYME

QLYSLQRTNSASSFHDPYVNCASAFHLAGLGLGSADFLGSRGLSSLGELHNAAVAAAAAG

SLASTDFHFSVDGNRRLGSPRPPGGSIRASISRKRALSSSPYSDSFDINSMIRFSPNSLA

TIMNGSRGSSAASGSYGHISATALNPMSHVHSTRLQQIQAHLLRASAGLLNPMTPQQVAA

SGFSIGHMPTSASLRVNDVHPNLSDSHIQITTSPTVTKDVSQVPAAAFSLKNLDDAREKK

GPFKDVVPEQPSSTSGGVAQVEADSASSQLSDRCYNNVVNNITGIPGDVKVNSRLDEYIN

CGSISIPSNEYDCANADTTDIKDEPGDFIETNCHWRSCRIEFITQDELVKHINNDHIQTN

KKAFVCRWEDCTRGEKPFKAQYMLVVHMRRHTGEKPHKCTFEGCFKAYSRLENLKTHLRS

HTGEKPYTCEYPGCSKAFSNASDRAKHQNRTHSNEKPYICKAPGCTKRYTDPSSLRKHVK

TVHGAEFYANKKHKGLPLNDANSRLQQNNSRHNLQEHNIDSSPCSEDSHLGKMLGTSSPS

IKSESDISSSNHHLVNGVRASDSLLTYSPDDLAENLNLDDGWNCDDDVDVADLPIVLRAM

VNIGNGNASASTIGGSVLARQRFRGRLQTKGINSSTIMLCNIPESNRTFGISELNQRITE

LKMEPGTDAEIKIPKLPNTTIGGYTEDPLQNQTSFRNTVSNKQGTVSGSIQGQFRRDSQN

STASTYYGSMQSRRSSQSSQVSSIPTMRPNPSCNSTASFYDPISPGCSRRSSQMSNGANC

NSFTSTSGLPVLNKESNKSLNACINKPNIGVQGVGIYNSSLPPPPSSHLIATNLKRLQRK

DSEYHNFTSGRFSVPSYMHSLHIKNNKPVGENEFDKAIASNARRQTDPVPNINLDPLTNI

SRFSTTPHSFDINVGKTNNIASSINKDNLRKDLFTVSIKADMAMTSDQHPNERINLDEVE

ELILPDEMLQYLNLVKDDTNHLEKEHQAVPVGSNVSETIASNHYREQSNIYYTNKQILTP

PSNVDIQPNTTKFTVQDKFAMTAVGGSFSQRELSTLAVPNEHGHAKCESFHHQSQKYMNT

DIGSKQQSALPSAHQRQTEKSNYNQIIDSSMTSLPELNVDSIYPRNETENIFKVHGDHDN

EIQCGIISQSQMSPSTNLNNDGQFSTVNMQPITTSKLFPPEPQKIVCDTQASNTSVMHLD

TYQRTLEYVQSCQNWMETNNTSTNQIQSLPGMPVNNTLFPDVSSSTHPYHGTNMVINDMT

TSLTSLLEENRYLQMMQ

>Apis mellifera(XP_624136)

LSSSTTAIGTAATGGGGGASSGASGGGGGGSGGGGGGAAGNGTSGGDFLRRSHPLSEHTA

LHPAYRLNYMDHLYHQLQASTHSPNASLHGLGGLGPEYLLHAAGPASTLASSEFPFSIDV

SASSRLGSPRASAIRASRKRALSSSPYSDRFDIDSMIRFSPNSLASIVNGSRSSSASGSY

GHLSAAMSPALGMHPGMAPHLQQLQAHLLRSAAAAALLPHAHPLQRPAPPPPPPHPHPHA

HGLSPHSQLYPGVPPHSTASATLGPHGGGLPPKTESAESCRKASESTTRSVTAEADTSSR

RTSTKVKREPATTTTNATTTTAPPTHPQGLSPSEDLRDEPGDFIETNCHWRDCGLEFPTQ

DDLVKHINNDHIHANKKSFVCGWEECSREEKPFKAQYMLVVHMRRHTGEKPHKCTFEGCF

KAYSRLENLKTHLRSHTGEKPYTCEYPGCSKAFSNASDRAKHQNRTHSNEKPYVCKAPGC

TKRYTDPSSLRKHVKTVHGAEFYANKKHKGGGGDGGGSDEAGAGGHSPSRSEDLHPKTPS

LSSPSVKSESEANSPPSMMQQQGSPLVGGCNDEVAGVGTLTGDGVALAEEPWNEEPDDLD

IADLPVALRAMVGGMESQQQQQPPPPASRNRLKGRLNAKGMPNLPVSVSSMRGTRGMGPQ

GNIGDLNRRITDLKMEGGGSARQTSLSDLQLRLQPLSEPRRDSNSTVSTYYGSMKSTDFG

SRRSSQASGVSAVRMGQTGPGSFYDPISPGTSRRSSQMSTTSGRMNPPNLQGPYSTSNLV

VQTQNMSLQGIQGIPGDWNGPSGHCTQLSGDRRMSEPTRGHQTQRNSPPVPPRPRSAQLP

EHHPNQEVILDEVGEGEMVENKLVIPDEMMQYLNQVQAGGTQVNCRSPLPICQSPICTNP

LHYQRQIQPTCNYNQSHQQTCYPSSQSAQPCTNYQNTSPSPYNQCPSSRPPQSNSQYCPP

PTYPLQPNGGQVLSPAAGQVMSPGSHYAPSHISDQPLTSPAAGALAPQHPPQNLPQNSAQ

LTRNCPQNHMHHGYYPNYNCQGQMNANCTGNPNGQVNHHTNSTCAPPNHNTMTQQQCVQM

RSAGNCQQLSSHCTQQPVIPNQTTMSHPNTQCGQVANQCTRPIVTPNGQMPNLHSVQQTT

VPNQCAQMSPHCSQLNMNNQAKPITNMTNLQGCQQQVQQPTTPCLQMANCAHPNSGHRPI

TDNTLSKRTSPQLCSAQQTNCRMQQQQHTCTHNCQARTNPPNHQYNCNCQWGYDQCYHEQ

TGTAMPEIQCRDISQSQQGSPIKPPQGMRQDSYRRTLEYVQQCRNWSGNAQTHETNVSSS

THPISLPQPLPSSANMVVNDMTSSLSSLLEENRYLQMIQ

>Caenorhabditis elegans (Tra)

MAPSTEDPDTVVEAQRRGSFSKKKNANGWNKVELVDQCAKQMGSEDKQPGGGDVKTENDP

SKNGLGSATSNFIQSSVPPSHQTLSNPLQLSPPAEASVAQQSGASQVFPTFQAALGASSD

ELLQPNATSSSTSSSASTSSIVPVVKFTNQTAPNGSTVATSVGQNVRLTINGKRVGRPPG

TFKRPQNNAANSSNSGNDSDMMGDHDLTCRWKSCNSSFQTLKALVDHVQESHVQSTEQEH

HAWRCEWEGCDRNETFKALYMLIVHVRRHTGEKPNKCEYPGCGKEYSRLENLKTHRRTHT

GEKPYKCEFADCEKAFSNASDRAKHQNRTHSNLKPYSCQIPQCTKSYTDPSSLRKHIKAV

HGDDEYEKAKKSRPANYSNRRRPDHRLAPPTGAMSHPYLATPNSGASVVAHSSVHQQNFI

NMALAQHHHNAQRAQQLMAATGNVMPMMDPASAAAAAQAQAHHQAQAQMLQTHMMQQAQI

QAAAQMQAQVQHQAAMQAHAMQQAQMVLQNNLLGAQSLLSPFSPLLPPSRAPNVMAMLQT

PPTPTSVAPMFDIMTSRAPMAPVVSAPTAPAPLVPAPVPASPVFDELREQMREVEPLQQQ

QQQEPMDQDLQDIRVDGDSDDEDEEEPRTPSGALLLPRGGNNGDGGFGGSGSSRASSGSG

TMELSAAPISQNGSRASGSGERGMRSFLIADILQLAADFQNERLLSDVLDLAIFDTRDVR

SLHNIYQVYIRAHKAIPITRRPLDWNETHQLHNLYHDPRFNRAEHQDSPAIRDRDTRFWR

TIAEANTMRQRQIEPVPLDDDDEGYFDEMVHRVQNGRLNEQFMEGFESDDDDGFEDEDDV

PGLGIAVYRGRRRVRREALKQANLDIQEAETAGRNVGGFGDEEDRNNRGHDQDRSFLDHY

YPPMVVVVETESPQIVRDQEMMRQFEEAKKNVETDEIKKRAEAMQFGTSSSHHHTKTLLI

QRALFDKTSSVRRSLLQFITISVDQEELRQSCHATSAPQGAHVVHNVVDEFDSIMRAQED

SNNRILLSLDIPAPSAVTGVSGSITHADNSALQLQQEQPTSSFSSWFPEDDPIYALPPPP

PPPAPPRRRRSADNKDDSENIPKKPRHQF

**Hedgehog family**

>Homo sapiens SHH

MLLLARCLLLVLVSSLLVCSGLACGPGRGFGKRRHPKKLTPLAYKQFIPNVAEKTLGASG

RYEGKISRNSERFKELTPNYNPDIIFKDEENTGADRLMTQRCKDKLNALAISVMNQWPGV

KLRVTEGWDEDGHHSEESLHYEGRAVDITTSDRDRSKYGMLARLAVEAGFDWVYYESKAH

IHCSVKAENSVAAKSGGCFPGSATVHLEQGGTKLVKDLSPGDRVLAADDQGRLLYSDFLT

FLDRDDGAKKVFYVIETREPRERLLLTAAHLLFVAPHNDSATGEPEASSGSGPPSGGALG

PRALFASRVRPGQRVYVVAERDGDRRLLPAAVHSVTLSEEAAGAYAPLTAQGTILINRVL

ASCYAVIEEHSWAHRAFAPFRLAHALLAALAPARTDRGGDSGGGDRGGGGGRVALTAPGA

ADAPGAGATAGIHWYSQLLYQIGTWLLDSEALHPLGMAVKSS

>Mus musculus SHH

MLLLLARCFLVILASSLLVCPGLACGPGRGFGKRRHPKKLTPLAYKQFIPNVAEKTLGAS

GRYEGKITRNSERFKELTPNYNPDIIFKDEENTGADRLMTQRCKDKLNALAISVMNQWPG

VKLRVTEGWDEDGHHSEESLHYEGRAVDITTSDRDRSKYGMLARLAVEAGFDWVYYESKA

HIHCSVKAENSVAAKSGGCFPGSATVHLEQGGTKLVKDLRPGDRVLAADDQGRLLYSDFL

TFLDRDEGAKKVFYVIETLEPRERLLLTAAHLLFVAPHNDSGPTPGPSALFASRVRPGQR

VYVVAERGGDRRLLPAAVHSVTLREEEAGAYAPLTAHGTILINRVLASCYAVIEEHSWAH

RAFAPFRLAHALLAALAPARTDGGGGGSIPAAQSATEARGAEPTAGIHWYSQLLYHIGTW

LLDSETMHPLGMAVKSS

>Rattus norvegicus SHH

MLLLLARCFLVALASSLLVCPGLACGPGRGFGKRRHPKKLTPLAYKQFIPNVAEKTLGAS

GRYEGKITRNSERFKELTPNYNPDIIFKDEENTGADRLMTQRCKDKLNALAISVMNQWPG

VKLRVTEGWDEDGHHSEESLHYEGRAVDITTSDRDRSKYGMLARLAVEAGFDWVYYESKA

HIHCSVKAENSVAAKSGGCFPGSATVHLEQGGTKLVKDLSPGDRVLAADDQGRLLYSDFL

TFLDRDEGAKKVFYVIETREPRERLLLTAAHLLFVAPHNDSGPTPGPSPLFASRVRPGQR

VYVVAERGGDRRLLPAAVHSVTLREEAAGAYAPLTAHGTILINRVLASCYAVIEEHSWAH

RAFAPFRLAHALLAALAPARTDGGGGGSIPAPQSVAEARGAGPAAGIHWYSQLLYHIGTW

LLDSETLHPLGMAVKSS

>Canis familiaris Shh

DEMLLLARCMLAVLVCALLMCSGLACGPGRGFGKRRHPKKLTPLAYKQFIPNVAEKTLGA

SGRYEGKISRNSERFKELTPNYNPDIIFKDEENTGADRLMTQRCKDKLNALAISVMNQWP

GVKLRVTEGWDEDGHHSEESLHYEGRAVDITTSDRDRSKYGMLARLAVEAGFDWVYYESK

AHIHCSVKAENSVAAKSGGCFPGSATVHLEQGGTKLVKDLRPGDRVLAADDQGRLLYSDF

LTFLDRDDGAKKVFYVIETREPRERLLLTAAHLLFVAPLNDSARVRPGQRVYVVAERGGD

RRLLPAAVHSVTLREEATGAYAPLTAQGTILINRVLASCYAVIEEHGWAHRAFAPFRLAH

ALRAADAPGAAGIHWYSQLLYQIGTWLLDSEALHPLGMAVKSS

>Gallus gallus SHH

MDEMLLLTRILLVGFICALLVSSGLTCGPGRGIGKRRHPKKLTPLAYKQFIPNVAEKTLG

ASGRYEGKITRNSERFKELTPNYNPDIIFKDEENTGADRLMTQRCKDKLNALAISVMNQW

PGVKLRVTEGWDEDGHHSEESLHYEGRAVDITTSDRDRSKYGMLARLAVEAGFDWVYYES

KAHIHCSVKAENSVAAKSGGCFPGSATVHLEHGGTKLVKDLSPGDRVLAADADGRLLYSD

FLTFLDREDSSRKLFYVIETRQPRARLLLTAAHLLFVAPQHNQSEATGSTSGQALFASNV

KPGQRVYVLGEGGQQLLPASVHSVSLREEASGAYAPLTAQGTILINRVLASCYAVIEEHS

WAHWAFAPFRLAQGLLAALCPDGAIPTAATTTTGIHWYSRLLYRIGSWVLDGDALHPLGM

VAPAS

>Xenopus tropicalis SHH

MLVVTRILLLGFICTLVAPPGLACGPGRGIGKRRHPKKLTPLAYKQFIPNVAEKTLGASG

RYEGKITRNSDRFKELTPNYNPDIIFKDEENTGADRLMTQRCKDKLNALAISVMNQWPGV

KLRVTEGWDEDGHHSEESLHYEGRAVDITTSDRDRSKYGMLARLAVEAGFDWVYFESKAH

IHCSVKA

>Tetraodon nigroviridis SHH

MLLWTRIVLVGLLCLSLVSSGMGCGPGRGYGRRRHPKKLTPLAYKQFIPNVAEKTLGASG

RYEGKITRNSERFKELTPNYNTDIIFKDEENTGADRLMTQRRVETAAALWECFEHYRDLK

NGWNSVKAHGDDGFLIARRCKDKLNSLAISVMNQWPGVKLRVTEGWDEDGHHFEESLHYE

GRAVDITTSDRDKSKYGTLSRLAVEAGFDWVYYESKAHIHCSVKAENSVAAKSGGCFPGS

STVTLQDGTKKLVKDLQSGDRVLAADGDGNPTYSDFITFIDRDSATRRLFHVIETDSGQK

ITLTAAHLLFVARNGSGGDGMSAVFASQVRRGQKVMVSDPERGRLAPVTVKRIYTQEHVG

SYAPVTVQGNVVVDEILASCYAVIEDHDLAHWALAPVRLAHWVSSLLSSSQPGGGGQRDG

VHWYSRLLYQVGTWLLDSQAIHPLGMSVSPS

>Danio rerio SHH

MRLLTRVLLVSLLTLSLVVSGLACGPGRGYGRRRHPKKLTPLAYKQFIPNVAEKTLGASG

RYEGKITRNSERFKELTPNYNPDIIFKDEENTGADRLMTQRCKDKLNSLAISVMNHWPGV

KLRVTEGWDEDGHHFEESLHYEGRAVDITTSDRDKSKYGTLSRLAVEAGFDWVYYESKAH

IHCSVKAENSVAAKSGGCFPGSALVSLQDGGQKAVKDLNPGDKVLAADSAGNLVFSDFIM

FTDRDSTTRRVFYVIETQEPVEKITLTAAHLLFVLDNSTEDLHTMTAAYASSVRAGQKVM

VVDDSGQLKSVIVQRIYTEEQRGSFAPVTAHGTIVVDRILASCYAVIEDQGLAHLAFAPA

RLYYYVSSFLFPQNSSSRSNATLQQEGVHWYSRLLYQMGTWLLDSNMLHPLGMSVNSS

>Danio rerio twhh

MDVRLHLKQFALLCFISLLLTPCGLACGPGRGYGKRRHPKKLTPLAYKQFIPNVAEKTLG

ASGKYEGKITRNSERFKELIPNYNPDIIFKDEENTNADRLMTKRCKDKLNSLAISVMNHW

PGVKLRVTEGWDEDGHHLEESLHYEGRAVDITTSDRDKSKYGMLSRLAVEAGFDWVYYES

KAHIHCSVKAENSVAAKSGGCFPGSGTVTLGDGTRKPIKDLKVGDRVLAADEKGNVLISD

FIMFIDHDPTTRRQFIVIETSEPFTKLTLTAAHLVFVGNSSAASGITATFASNVKPGDTV

LVWEDTCESLKSVTVKRIYTEEHEGSFAPVTAHGTIIVDQVLASCYAVIENHKWAHWAFA

PVRLCHKLMTWLFPARESNVNFQEDGIHWYSNMLFHIGSWLLDRDSFHPLGILHLS

>Takifugu rubripes SHH

MLLWTRIVLVGLLCLSLVSSGMGCGPGRGYGRRRHPKKLTPLAYKQFIPNVAEKTLGASG

RYEGKITRNSERFKELTPNYNTDIIFKDEENTGADRLMTQRCKDKLNSLAISVMNQWPGV

KLRVTEGWDEDGHHFEESLHYEGRAVDITTSDRDKSKYGTLSRLAVEAGFDWVYYESKAH

IHCSVKAENSVAAKSGGCFPGSSTVTLQDGTKKLVKHLRSGDRVLAADDDGNPTYTDFIM

FVDRDSTTRRLYHVIETDSGQKITLTAAHLLYVARNGTEGDGMSAVFASQVRRGQKVIVS

DPERSRLEPVTVERIYTQEHVGSYAPVTVQGNVVVDEILASCYAVIEDHDLAHWALAPVR

LAHWVSSLLSRSQPGGGGQKDGVHWYSRLLYQVGTWLLDGHAIHPLGMSVSPS

>Gasterosteus aculeatus SHH

MLLWNRIVLVGLICLSLVSSGMGCGPGRGYGRRRHPKKLTPLAYKQFIPNVAEKTLGASG

RYEGKITRNSERFKELTPNYNTDIIFKDEENTGADRLMTQRCKDKLNSLAISVMNQWPGV

KLRVTEGWDEDGHHFEESLHYEGRAVDITTSDRDKSKYGTLSRLAVEAGFDWVYYESKAH

IHCSVKAENSVAAKSGGCFPGSSTVTLQDGTKKAVKHLQTGDRVLAGDVEGNPTYTDFIM

FIDQDSTTRRLFHVIETDSGQKITLTAAHLLFVGRNATAGERMSAVFASQVRSGQKVFVF

DAERSRLEPVTVRRIYTREHEGSFAPVTAQGTVVVDQVLASCYAVIEDHDLAHWALAPVR

LAHWVSSLLFRSQRGASAQSDGVHWYSKMLYQVGSWLLDSHSIHPLGMSVYSS

>Oryzias latipes SHH

KMLLWAKFVLVGLFCLSLVSSGMGCGPGRGYGRRRHPKKLTPLAYKQFIPNVAEKTLGAS

GRYEGKITRNSERFKELTPNYNTDIIFKDEENTGADRLMTQRCKDKLNSLAISVMNQWPG

VKLRVTEGWDEDGHHFEESLHYEGRAVDITTSDRDKSKYGTLSRLAVEAGFDWVYYESKA

HIHCSVKAENSVAAKSGGCFPGSSTVTLENGTQRPVKDLQPGDRVLAADYDGNPVYTDFI

MFIDRDSSTSRIFYVIETESGQKVTLTAAHLLFIAHNSTQAERRMSAVFASQVRPGQKVF

VFGRGLLAPVTVKRIYTQRHEGSFAPVTAQGTVVVDQVLASCYAVIEDHSLAHWALAPVR

LAHWVSSLLFGSQPRVSAQSEGVHWYSKILYQLGTWLLDSHSIHPLGMSVSPS

>Homo sapiens DHH

MALLTNLLPLCCLALLALPAQSCGPGRGPVGRRRYARKQLVPLLYKQFVPGVPERTLGAS

GPAEGRVARGSERFRDLVPNYNPDIIFKDEENSGADRLMTERCKERVNALAIAVMNMWPG

VRLRVTEGWDEDGHHAQDSLHYEGRALDITTSDRDRNKYGLLARLAVEAGFDWVYYESRN

HVHVSVKADNSLAVRAGGCFPGNATVRLWSGERKGLRELHRGDWVLAADASGRVVPTPVL

LFLDRDLQRRASFVAVETEWPPRKLLLTPWHLVFAARGPAPAPGDFAPVFARRLRAGDSV

LAPGGDALRPARVARVAREEAVGVFAPLTAHGTLLVNDVLASCYAVLESHQWAHRAFAPL

RLLHALGALLPGGAVQPTGMHWYSRLLYRLAEELLG

>Mus musculus DHH

MALPASLLPLCCLALLALSAQSCGPGRGPVGRRRYVRKQLVPLLYKQFVPSMPERTLGAS

GPAEGRVTRGSERFRDLVPNYNPDIIFKDEENSGADRLMTERCKERVNALAIAVMNMWPG

VRLRVTEGWDEDGHHAQDSLHYEGRALDITTSDRDRNKYGLLARLAVEAGFDWVYYESRN

HIHVSVKADNSLAVRAGGCFPGNATVRLRSGERKGLRELHRGDWVLAADAAGRVVPTPVL

LFLDRDLQRRASFVAVETERPPRKLLLTPWHLVFAARGPAPAPGDFAPVFARRLRAGDSV

LAPGGDALQPARVARVAREEAVGVFAPLTAHGTLLVNDVLASCYAVLESHQWAHRAFAPL

RLLHALGALLPGGAVQPTGMHWYSRLLYRLAEELMG

>Rattus norvegicus DHH

MALPASLLPLCCLALLALSVQSCGPGRGPVGRRRYVRKQLVPLLYKQFVPSMPERTLGAS

GPAEGRVTRGSERFRDLVPNYNPDIIFKDEENSGADRLMTERCKERVNALAIAVMNMWPG

VRLRVTEGWDEDGHHAQDSLHYEGRALDITTSDRDRNKYGLLARLAVEAGFDWVYYESRN

HIHVSVKADNSLAVRAGGCFPGNATVRLRSGERKGLRELHRGDWVLAADAAGRVVPTPVL

LFLDRDLQRRASFVAVETERPPRKLLLTPWHLVFAARGPAPAPGDFAPVFARRLRAGDSV

LAPGGDALRPARVARVAREEAVGVFAPLTAHGTLLVNDVLASCYAVLESHQWAHRAFAPL

RLLHALGALLPGGAVQPTGMHWYSRLLYRLAEELMG

>Bos taurus DHH

ASMALPASLVPLCCLALLALPAQSCGPGRGPVGRRRYVRKQLVPLLYKQFVPSVPERTLG

ASGPAEGRVTRGSERFRDLVPNYNPDIIFKDEENSGADRLMTERCKERVNALAIAVMNMW

PGVRLRVTEGWDEDGHHAQDSLHYEGRALDITTSDRDRNKYGLLARLAVEAGFDWVYYES

RNHVHVSVKADNSLAVRAGGCFPGNATVRLRSGERKGLRELHRGDWVLAADAAGRVVPTP

VLLFLDRDLQRRASFVAVETERPPRKLLLTPWHLVFAARGPAPAPGDFAPVFARRLRAGD

SVLAPGGDALRPARVARVAREEAVGVFAPLTAHGTLLVNDVLASCYAVLESHQWAHRAFA

PLRLLHALGALLPGGAVQPTGMHWYSRFLYRLAEELLD

>Danio rerio DHH

MTLAPWLRLARLGLLTVCLYSWLVVDGRGPGPGYGGRHRQRKLTPMSYKQYVPGVSENNL

GASGRAEGRITRSSERFNELVCNYNTDIDFKDEERSNADRFMTKRCKDCLNKLAIAVMNQ

WPGVRLRVTEAWDEDGHHPPGSLHYEGRAVDITTSDRDTKKYGLLAQLAVEAGFDWVHYE

SKYHVHCSVKADHSVAVEKGGCFSASGLVTMADGVQKPMSCLWPGEKVLSVSGSGEVVFS

RVLLFLHLDRESRTSFFIITTENEKRIALTPNHLIFAAHNLKLHHHDYETVFARNVRIGD

YILTTGGDRGIQPSKVVSVSLEERMGVYAPLTEHGNLFVDGVLASNYATFQDHGLAHTVF

WPFRVLFVFFNKEMEEDLQRVAVPYICSTNQTILTSVMHSRLSSVFKWQDATRAEMENAF

LQQKEVYWYARLLHTLGRIFLDPQRFY

>Gasterosteus aculeatus DHH

PQPSPMKQSLWARLAQVGLLAVWTCVWLVQGCGPGPGYGIRSRTRRLTAMHYKQFFPNFS

ENNLGASGRAEGKITRNSERFNELVCNYNTDIVFKDEENTNADRFMTKRCKDCLNRLAIA

VMNQWPGVHLRVTEAWDEDGHHPPGSLHYEGRAVDITTDDRETEKYGLLAQLAVEAGFDW

VHYESKYHIHCSVKADHSVAVEKGGCFPGWARVTVAGGVQKSLSSLAAGDRVMALSGTGQ

VVLSQVLLFLHQDHESWSTFLSLETEDGHRLALTPHHLVFLSSHCRLDSSEYEAQFASRA

KTGDCVLIHTAEAQVRPSRIISVSEEESVGVYAPLTEAGSVFVEGVLASCYALMDDHRLA

HWAFGPVRLLSSFNHLLWEETRHVHWYAELLYSLRWTFLDSNSFYP

>Oryzias latipes DHH

MKQFCWARLVQLCLLAAWTCVWLVEGCGPGPGYGIRSRPRKLTAMHYKQFFPNFSENNLG

ASGRAEGKITRDSERFNELVCNYNPDIVFKDEENTDADRLMTKRCKDCLNRLAIAVMNQW

PGVHLRVTEAWDEDGHHPQGSLHYEGRAVDITTDDRETEKYGLLAQLAVEAGFDWVHYES

KYHVHCSVKADHSVAVERGGCFPGWARVTLAGGRQKTLSSLAPGDRVMALSDTGQVVYTR

VLSFLHQDQESVSTFLSLETEEGYTLVLTPHHLVFLAPNCGHDISEYQARFASRAKTGDC

VLISTADSQTLPSPIVSVSVAESVGVYAPLTEAGTLFVDRVLASSYALVEDHRLAHWAFG

PLRLFYSLKELLWAEAADQTTTGSISLTNIRSHFSTLTIKHNNLTEPFRMKIQEKRLSKI

HWYARLLYRFAWVLLDSNLFYP

>Homo sapiens IHH

MSPARLRPRLHFCLVLLLLLVVPAAWGCGPGRVVGSRRRPPRKLVPLAYKQFSPNVPEKT

LGASGRYEGKIARSSERFKELTPNYNPDIIFKDEENTGADRLMTQRCKDRLNSLAISVMN

QWPGVKLRVTEGWDEDGHHSEESLHYEGRAVDITTSDRDRNKYGLLARLAVEAGFDWVYY

ESKAHVHCSVKSEHSAAAKTGGCFPAGAQVRLESGARVALSAVRPGDRVLAMGEDGSPTF

SDVLIFLDREPHRLRAFQVIETQDPPRRLALTPAHLLFTADNHTEPAARFRATFASHVQP

GQYVLVAGVPGLQPARVAAVSTHVALGAYAPLTKHGTLVVEDVVASCFAAVADHHLAQLA

FWPLRLFHSLAWGSWTPGEGVHWYPQLLYRLGRLLLEEGSFHPLGMSGAGS

>Mus musculus IHH

MESPRATQTPESPKLSQPRAHLSAHQAPSPAALPGYPAMSPAWLRPRLRFCLFLLLLLLV

PAARGCGPGRVVGSRRRPPRKLVPLAYKQFSPNVPEKTLGASGRYEGKIARSSERFKELT

PNYNPDIIFKDEENTGADRLMTQRCKDRLNSLAISVMNQWPGVKLRVTEGWDEDGHHSEE

SLHYEGRAVDITTSDRDRNKYGLLARLAVEAGFDWVYYESKAHVHCSVKSEHSAAAKTGG

CFPAGAQVRLENGERVALSAVKPGDRVLAMGEDGTPTFSDVLIFLDREPNRLRAFQVIET

QDPPRRLALTPAHLLFIADNHTEPAAHFRATFASHVQPGQYVLVSGVPGLQPARVAAVST

HVALGSYAPLTRHGTLVVEDVVASCFAAVADHHLAQLAFWPLRLFPSLAWGSWTPSEGVH

WYPQMLYRLGRLLLEESTFHPLGMSGAGS

>Rattus norvegicus IHH

LESPRATQTPESPKLCQPRTHPSAHQAPSPVALPGYPAMSPAWLRPRLRFCLLLLLLLLV

PAARGCGPGRVVGSRRRPPRKLVPLAYKQFSPNVPEKTLGASGRYEGKIARSSERFKELT

PNYNPDIIFKDEENTGADRLMTQRCKDRLNSLAISVMNQWPGVKLRVTEGWDEDGHHSEE

SLHYEGRAVDITTSDRDRNKYGLLARLAVEAGFDWVYYESKAHVHCSVKSEHSAAAKTGG

CFPAGAQVHLETGERVALSAVKPGDRVLAMGEDGNPTFSDVLIFLDREPNRLRAFQVIET

QDPPRRLALTPAHLLFIADNHTEPAARFRATFASHVQPGQYVLVAGVPGLQPARVAAIST

HVALGSYAPLTRHGTLVVEDVVASCFAAVSDHHLAQLAFWPLRLFPSLPWGSWTPSEGVH

WYPQLLYRLGRLLLEESTFHPLGMSGAGS

>Gallus gallus IHH

MKPARLLLLLSGCALLLAPAVRCCGPGRVVGSRRRPPRKLIPLAYKQFSPNVPEKTLGAS

GRYEGKIARNSERFKELTPNYNPDIIFKDEENTGADRLMTQRCKDRLNSLAISVMNQWPG

VKLRVTEGWDEDGHHSEESLHYEGRAVDITTSDRDRNKYGMLARLAVEAGFDWVYYESKA

HIHCSVKSEHLVAAKTGGCFSGRALATLENGARTPLWALRPGQRVLAMDGAGRPTYSDFL

AFLDKEPRALTAFHVIETRQPPRRLALTPTHLLFVADNASAPAAQFRPTFASHVQPGHFV

LVAVGSGGLQPAEVVGVRGRTDVGAYAPLTRHGTLVVDDVVASCFALVREQQLAQMAFWP

LRLYHSLLGGPGVQGDGVHSYSGLLYRLGRMLLPPDSFHPLGAPRAES

>Bos taurus IHH

LEPPGATRTPEPPQRSGLLVHPSAHQASSPAAPRGSPAMSPARLRPRLRFCLLLLLLLLL

VPAARGCGPGRVVGSRRRPPRKLVPLAYKQFSPNVPEKTLGASGRYEGKIARSSERFKEL

TPNYNPDIIFKDEENTGADRLMTQRCKDRLNSLAISVMNQWPGVKLRVTEGWDEDGHHSE

ESLHYEGRAVDITTSDRDRNKYGLLARLAVEAGFDWVYYESKAHVHCSVKSEHSAAAKTG

GCFPAGAQVRLESGARVALSAVRPGDRVLAMGEDGNPTFSDVLIFLDREPDRLRAFQVIE

TQDPPRRLALTPAHLLFTANNHTEPAAHFRATFASQVQPGQYVLVAGVPGLQPARVAAVS

THVALGAYAPLTRHGTLVVEDVVASCFAAVADHHLAQLAFWPLRLFHSLAWGSWTPGEGV

HWYPQLLYRLGRLLLEEGSFHSLGVAGAGS

>Danio rerio IHH

MRLPVVFGLLVGCALIFAPVNEGCGPGRGHGKRRPPKKLTPLNYKQFSPNVAEKTLGASG

RIEGKITRNSERFKELTPNYNPDIIFKDEENTGADRLMTQRCKDKLNSLAISVMNMWPGV

KLRVTEGWDEDGNHFEDSLHYEGRAVDITTSDRDRNKYGMLARLAVEAGFDWVYYESKAH

IHCSVKSEHSVAAKTGGCFPASALVTVEDGSLKTLDSLQPGEKVLASSESDGSGTLVYSE

VIAFLDRDPSARKQFFTIETDSGAKLSLTAAHLLFVSEGNCSGSAANAELRSVFASDVLP

GQCVVSTQAAGQHGRLSRVSRIQMQEDRGVFAPLTSHGTVVVNGIVSSCYAAVDQHWLAH

WAFGPLRVLYNWGGPVGHQVTGIHWYSSLLHWIGTQVLDPAHFHPWSMMDNDR

>Tetraodon nigroviridis IHH

MRISFLRLTASLCALVLLFLLAPASEGCGPGRGYGKRRLPKKLIPLAYKQFSPNVAEKTL

GASGRPEGKITRNSERFKELTPNYNTDIIFKDEEDTGADRLMTQRCKDKLNSLAISVMNM

WPGVKLRVTEGWDEDGHHSGDSLHYEGRAVDITTSDRDRNKYAMLARLAVEAGFDWVYYE

SKAHIHCSVKSEHSVAAKTGGCFHGDTQVILEGSVTKPMRDLRPGDRVLASSGGGGPLVY

SSVLSFLDRQPNATSVFYTIGTDAGHRITLTAAHLIYVTDCAWSGTHGEPYLDSVQGGRS

SRDRGLRIVFASEVRPGQCVLTSGGKLGSPATFSPVTFVHVRRSTGLYAPLTEHGSIVVN

GVLASCYAVVDSHHLAHWALAPLRFFYALMGPSGSQTDGLHWYPWLLQRLGTTLL

>Takifugu rubripes IHH

VLLFLLAPASEGCGPGRGYGKRRLPKKLIPLAYKQFSPNVAEKTLGASGRPEGKITRNSE

RFKELTPNYNTDIFFKDEEETGADRLMTQKHTHTDLLHVFGSGLSKKTPFLPSLCCLQRC

KDKLNSLAISVMNMWPGVKLRVTEGWDEDGHHSEDSLHYEGRAVDITTSDRDRNKYAMLA

RLAVEAGFDWVYYESKAHIHCSVKSEHSVAAKTGGCFSGDAQVVLEGGVTKPMRDLRPGD

RVLSSSATEGLGPFIYSPVLSFLDRRPNVTSVFYTIGTVAGHSITLTAAHLIYVTDCVWN

ETYGDLYLDPAQEVGFSRDRGLRIVFASEVQPGQCVLTSGGKVGSQATFSPVTFVRAQRS

TGLYAPLTEHGSIVVNGVLASCYAAVDSHRLAHWALAPLRLFYTLLGPSGSQTDGLHWYP

WLLQRLGTMLLDTEHFHPWGV

>Gasterosteus aculeatus IHH

MRIPFHLLTASLCATAPASEGCGPGRGYGKRRPPKKLIPLAYKQFSPNVAEKTLGASGRP

EGKITRNSERFKELTPNYNTDIIFKDEEDTGADRLMTQRCKDKLNSLAISVMNMWPGVKL

RVTEGWDEDGHHSEDSLHYEGRAVDITTSDRDRNKYAMLARLAVEAGFDWVYYQSKAHIH

CSVKSEHSVAAKTGGCFPGDAQVTLSGGVTKRMQDLRPGDHVLASSTTDGHGPLIYSPVS

SFLDYQPNVTKTFCRIGTDTGLIITVTAAHLIFVTDCTGAKSREAGVRTVFASEVRPGQC

VLTSHGKAGSRASLSVVTFVEEQGGTGLYAPLTQHGSIVVNGVLASCYAAVDNHHLAHWV

LTPLRFFYSLMGRSEPQSHGLHWYPRLLQRLGKTLLGAGHFHPWGTEQGHR

>Oryzias latipes IHH_A

MLLTTLVTCLAGCAMLLSPASEGCGPGRGHGKRRSQRKLVPLSYKQFSPNVAEKTLGASG

RYEGKITRNSERFKELTPNYNPDIIFKDEENTGADRLMTQRCKDKLNSLAISVMNLWPKV

RLRVTEGWDEDGHHSEESLHYEGRAVDITTSDRDRNKYAMLARLAVEAGFDWVYYESKAH

IHCSVKSANXTFAKKKGGFVLMVTTALRDGYGLPAGEKWIFCAAHSETIFSHLIIILELD

LPKTKXFYTLQTEAGPELSLTAAHLLFVSEGNCSEGTVPGPGALKTVYASDVQPGQCVLV

LGGQSGCLSRVNRVSVAVKRGVFAPLTQQGTVVVDGVLASCYAAVNQHLLAHWSFSPLRL

MHSWTGSTGGHSEGLHWYAQLLYWLGRKLLSSGHLHPLAVAQDNR

>Oryzias latipes IHH_B

PPGAMRISLFLLTASLCALVLLLAPALEGCGPGRGIGKRRFPKKLIPLAYKQFSPNVAEK

TLGASGRPEGKITRSSERFKELTPNYNTDIIFKDEEDTGADRLMTQRCKDKLNSLAISVM

NMWPGVKLRVTEGWDEDGHHSEDSLHYEGRAVDITTSDRDRNKYNMLARLAVEAGFDWVY

YESKAHVHCSVKSEHSVAAKTGGCFPGDAQVFLEGGGTKQMRHLLPGDRVLSSSTKDGHT

SLLYSPVISFLDRQPNIMKTFYIIGTDAGFNISLTAAHLIFTADCADGGNKTKPQETFFP

SIWTDRGRSGAHLRTVFASQVQPGQCVYTSSEESEPHVRISVVTFVEEQRSTGLYAPLTQ

HGSIVVNGVLSSCYAAVDSHELSHWAFAPLRFLYSMLGSSQAQSDGVHWYPRLLHRLGEL

LLDAGHFHPWGI

>Branchiostoma floridae HH

MAGVLARWMVTLVAISALGTHWGPSEACGPGGRFGRRRHPRKLTPFVYKQQMPAVSENTFGASGLFNGRI

TRDSERFHTLKQNFNTDIIFKDEEKTGADRFMTQTPHSQIRVLGSVLLGLTAEQVCIYLGVDRNTLQRCK

DKLNALAISVMNQWEGVKLRVTEGWDEDGFHTEESLHYEGRAVDITTSDRDRTKYGMLARLAVEAGFDWV

YYESKAHIHCSVKAESDTTATQGGCFPAESWVTRDDGNRIRMRDVRPGDKVLSMDSGGHPVFSEVLTFMD

RDSRGPWVYYTIHTDDRNITVTATPSHLVFVTESRDLSSPRIAKFMSDARPGEFLLTPDSDGGGFRKVKI

VSVTMREEKGAYAPLTVHGTVVVDNVAMSCYALIESQALAHWVFAPFRLYYQLTSSLWDGPSHDQTLQEG

VHWYPSFFYRYGISLVEPTLLHPTATDS

>Strongylocentrotus purpuratus hh

MVRADMVKWLTVQITTVLCLIALTQACHPGRSGKTSHRPRNRTPLQYKQRVPNISEDTFG

ASGPPEGRINRNDERFNTLSPNNNDDIVFKDKEGTGADRLMTQRCKDKLNTLAISVMNEW

PGIKLRVVEAWDEDQPNVEPLHAEGRAVDITTSDRDKNKYGALARLAVEAGFDWVNYESK

AWVHCSVKSESAAAKNSGGCFPGFSQASLENGRTISMLDIRVGDEVAVVNDDGALDYSDV

IMIVHRKLNDSTLFYVIETEDKSVVQLTPQHLIYVSETESSFSQSKAMFASEVRTNQFVY

TTGQNHDRGVRPKRVVSVTTRLGRTAVAPVTRQGSLVIDDVAISSYAVMRDEWIAHASFA

PVRWYSYIRHNMLGIVDTNTGQEQRVHWYTQRLYKLGKYVMSDRLFLGFDV

**IGFBP family**

>Homo sapines IGFBP4

MLPLCLVAALLLAAGPGPSLGDEAIHCPPCSEEKLARCRPPVGCEELVREPGCGCCATCA

LGLGMPCGVYTPRCGSGLRCYPPRGVEKPLHTLMHGQGVCMELAEIEAIQESLQPSDKDE

GDHPNNSFSPCSAHDRRCLQKHFAKIRDRSTSGGKMKVNGAPREDARPVPQGSCQSELHR

ALERLAASQSRTHEDLYIIPIPNCDRNGNFHPKQCHPALDGQRGKCWCVDRKTGVKLPGG

LEPKGELDCHQLADSFRE

>Mus musculus IGFBP4

MLPFGLVAALLLAAGPRPSLGDEAIHCPPCSEEKLARCRPPVGCEELVREPGCGCCATCA

LGLGMPCGVYTPRCGSGMRCYPPRGVEKPLRTLMHGQGVCTELSEIEAIQESLQTSDKDE

SEHPNNSFNPCSAHDHRCLQKHMAKIRDRSKMKIVGTPREEPRPVPQGSCQSELHRALER

LAASQSRTHEDLFIIPIPNCDRNGNFHPKQCHPALDGQRGKCWCVDRKTGVKLPGGLEPK

GELDCHQLADSFQE

>Rattus norvegicus IGFBP4

MLPFGLVAALLLAAGPRPSLGDEAIHCPPCSEEKLARCRPPVGCEELVREPGCGCCATCA

LGLGMPCGVYTPRCGSGMRCYPPRGVEKPLRTLMHGQGVCTELSEIEAIQESLQTSDKDE

SEHPNNSFNPCSAHDHRCLQKHMAKVRDRSKMKVVGTPREEPRPVPQGSCQSELHRALER

LAASQSRTHEDLFIIPIPNCDRNGNFHPKQCHPALDGQRGKCWCVDRKTGVKLPGGLEPK

GELDCHQLADSLQE

>Xenopus tropicalis IGFBP4

LHMMSGNCHPALLLLVLATFGMAEDAAIQCPPCSQEKLIRCTDPVGCQELVKEPGCGCCA

TCALPKGAPCGVYTARCGTGLRCYPPRGSEKPLHTLMHGQGLCTEIGEIESITETFPKTG

EYIAVGRGQTSQTVWMHHNQIAVFVYFTLGPMPNGCLGFSLVLIILSIRLSSHLHHHKKG

EDEGVCQKDLNRALEKLAAYQTRTQEDFLSIPIPNCDRNGNYNPKQCHPALDGQRGKCWC

VDRKTGVKLHIPYDPVLDADCQVASERGK

>Tetraodon nigroviridis IGFBP4

MIRGSAVALASSCWGFWAFSALSLAALCLSDQAIRCPVCSEERLARCRLPEGCEETVREP

GCGCCPTCALAKGAHCGVYSPRCGTGLRCYPPRGVERPLHSLMHGQGVCTDEREVEENSA

TDRQDEIIPEHPNNSNIRCSQQDKRCIQKTLARHLSKSTNQRSNSGREETKAVLAPCRAE

LQRALDRLASNTRTHDDLFTIPIPNCDKNGDFHIKQCHPARDGQRGKCWCVDQKTGMRLP

GPLEPRGDLDCHQLT

>Takifugu rubripes IGFBP4

MIPGRAVALASSCWGFWAFSALSLVALCLSDQAIRCPVCSEERLARCRLPEGCEETVREP

GCGCCPTCALAKGAHCGVYSPRCGTGLRCYPPRGVERPLHSLMHGQGVCTDEREVEENSA

MDRQDEIIPEHPNNSNIRCSPQDKRCIQKTLARHPPKSTNQRSNTAREETKAVLAPCRAE

LQRALDRLASNTRTHDDLFTIPIPNCDKNGDFHIKQCHPARDGQRGKCWCVDQKTGMRLP

GPLELRGDLDCHQL

>Homo sapines IGFBP1

MSEVPVARVWLVLLLLTVQVGVTAGAPWQCAPCSAEKLALCPPVSASCSEVTRSAGCGCC

PMCALPLGAACGVATARCARGLSCRALPGEQQPLHALTRGQGACVQESDASAPHAAEAGS

PESPESTEITEEELLDNFHLMAPSEEDHSILWDAISTYDGSKALHVTNIKKWKEPCRIEL

YRVVESLAKAQETSGEEISKFYLPNCNKNGFYHSRQCETSMDGEAGLCWCVYPWNGKRIP

GSPEIRGDPNCQIYFNVQN

>Mus musculus IGFBP1

MPEFLTVVSWPFLILLSFQIGVAAGAPQPWHCAPCTAERLGLCPPVPASCPEISRPAGCG

CCPTCALPMGAACGVATARCAQGLSCRALPGEPRPLHALTRGQGACVPEPAAPATSTLFS

SQHEEAKAAVVSADELSESPEMTEEQLLDSFHLMAPSREDQPILWNAISTYSSMRAREIA

DLKKWKEPCQRELYKVLERLAAAQQKAGDEIYKFYLPNCNKNGFYHSKQCETSLDGEAGL

CWCVYPWSGKKIPGSLETRGDPNCHQYFNVHN

>Rattus norvegicus IGFBP1

MPEFLTVVSWPFLILLSFQVRVVAGAPQPWHCAPCTAERLELCPPVPASCPEISRPAGCG

CCPTCALPLGAACGVATARCAQGLSCRALPGEPRPLHALTRGQGACVLEPAAPATSSLSG

SQHEEAKAAVASEDELAESPEMTEEQLLDSFHLMAPSREDQPILWNAISTYSSMRAREIT

DLKKWKEPCQRELYKVLERLAAAQQKAGDEIYKFYLPNCNKNGFYHSKQCETSLDGEAGL

CWCVYPWSGKKIPGSLETRGDPNCHQYFNVQN

>Xenopus tropicalis IGFBP1

MARENISCCSLLATLLFIAAAIVVAEPLHCAQCTEERLASCPSVPNDCPELAREPGCGCC

LTCALKRGEPCGVYTARCGKALSCHPKAGESRPLYALTRGQGICMDAEDLKKLRASDAAD

GKDYTEHENVAPEVSDISHDQIPPFLRLFPEGLDKLDPWNAITVYEKARKLLERRKWKEH

QGPCQKDLYKTMDKLVKAQQRPGEDVFKFHIPNCNRNGFYHSKQCEASLDGERGKCWCVF

PLTGKRIPGSPENRGELNCQQYLNVQE

>Takifugu rubripes IGFBP1

ATGALGSPVVGPEPIRCAPCSPERLSQCPAVAPGCAEVLREPGCGCCLACALPAGELCGI

YTAPCGSGLRCTPRPGDPRPLHSLTRGQAVCTASAEPQSTPEPQQTQDQAEPEPEIENTV

IVTDLSSSHYLPGHSTPYDPRASADAQESMKAKVIAIRKKLVEQGPCHLELQRALDKIAT

SQQRPGEKLTRFYLPNCDKHGLYKPKQCESSLDGQRGRCWCVNSWNGKKTSGVSEVPVDA

ECP

>Homo sapines IGFBP2

MLPRVGCPALPLPPPPLLPLLLLLLGASGGGGGARAEVLFRCPPCTPERLAACGPPPVAP

PAAVAAVAGGARMPCAELVREPGCGCCSVCARLEGEACGVYTPRCGQGLRCYPHPGSELP

LQALVMGEGTCEKRRDAEYGASPEQVADNGDDHSEGGLVENHVDSTMNMLGGGGSAGRKP

LKSGMKELAVFREKVTEQHRQMGKGGKHHLGLEEPKKLRPPPARTPCQQELDQVLERIST

MRLPDERGPLEHLYSLHIPNCDKHGLYNLKQCKMSLNGQRGECWCVNPNTGKLIQGAPTI

RGDPECHLFYNEQQEARGVHTQRMQ

>Mus musculus IGFBP2

MLPRLGGPALPLLLPSLLLLLLLGAGGCGPGVRAEVLFRCPPCTPERLAACGPPPDAPCA

ELVREPGCGCCSVCARQEGEACGVYIPRCAQTLRCYPNPGSELPLKALVTGAGTCEKRRV

GTTPQQVADSDDDHSEGGLVENHVDGTMNMLGGGSSAGRKPLKSGMKELAVFREKVNEQH

RQMGKGAKHLSLEEPKKLRPPPARTPCQQELDQVLERISTMRLPDDRGPLEHLYSLHIPN

CDKHGRYNLKQCKMSLNGQRGECWCVNPNTGKPIQGAPTIRGDPECHLFYNEQQETGGAH

AQSVQ

> Rattus norvegicus IGFBP2

MLPRLGGPALPLLLPSLLLLLLLGAGGCGPGVRAEVLFRCPPCTPERLAACGPPPDAPCA

ELVREPGCGCCSVCARQEGEACGVYIPRCAQTLRCYPNPGSELPLKALVTGAGTCEKRRV

GATPQQVADSEDDHSEGGLVENHVDGTMNMLGGSSAGRKPPKSGMKELAVFREKVNEQHR

QMGKGAKHLSLEEPKKLRPPPARTPCQQELDQVLERISTMRLPDDRGPLEHLYSLHIPNC

DKHGLYNLKQCKMSLNGQRGECWCVNPNTGKPIQGAPTIRGDPECHLFYNEQQENDGAHA

QRVQ

> Xenopus tropicalis IGFBP2

RCWIWKPRQRRGTACPNWPVXLPLQALVLGLGTCGKRRDTEYGSSQERGTELPEERSDNM

LVDNKLEAGPAVAGEAAPRKPSKKEMKEIAVTRERANEQQRSKSNKSEDKKRPARSLCQL

QLDQVLERISGMHLPDDRGPLEHLYALHIPNCDKNGFFNLKQCKMSVNGQRGECWCVNPI

TGKALPGSPTIRGDPECHLYYTSPEEGRAHTQRAP

> Takifugu rubripes IGFBP2

MLLYVSCNLLIISASLAGASLAEMVFRCPGCTAERQALCPKLTETCAEIVREPGCGCCPV

CARQEGEMCGVYTPRCATGLRCYPTPNSELPLEQLVQGQGQCRHKVDTEAATYSQEQREQ

TSGEAVEAPPEQGMSDIPAIRKPSKDATWMSPKESAVRQHRQDLKTKMKTNKVEEVKPAQ

PKQTLCQQELDQILERISKMPFRDNRGPLEDLYALHIPNCDKRGQYNLKQCKMSLHGQRG

ECWCVNPHTGRPIPSAPTVRGDPNCSQYLAE

>Tetraodon nigroviridis IGFBP2

MLLYAGCSLLILSASLAGASLAEMVFRCPGCTAERQALCPKLTETCAEIVREPGCGCCPV

CARQEGEMCGVYTPRCATGLRCYPTPNSELPLEQLVQGQGQCRRKVDTEAATYSQEQREQ

TSGEVVEPPPELGVSDLPAIRKPSKDAAWMGPKESAVRQHRQELKTKMKTNKVEEVKTSR

PKQTLCQQELDQILERISKMPFRDNRGPLEDLYALHIPNCDKRGQYNLKQCKMSLHGQRG

ECWCVNPHTGRPIPSAPTVRGDPNCSQYLTE

>Homo sapines IGFBP3

MQRARPTLWAAALTLLVLLRGPPVARAGASSAGLGPVVRCEPCDARALAQCAPPPAVCAE

LVREPGCGCCLTCALSEGQPCGIYTERCGSGLRCQPSPDEARPLQALLDGRGLCVNASAV

SRLRAYLLPAPPAPGNASESEEDRSAGSVESPSVSSTHRVSDPKFHPLHSKIIIIKKGHA

KDSQRYKVDYESQSTDTQNFSSESKRETEYGPCRREMEDTLNHLKFLNVLSPRGVHIPNC

DKKGFYKKKQCRPSKGRKRGFCWCVDKYGQPLPGYTTKGKEDVHCYSMQSK

>Mus musculus IGFBP3

MHPARPALWAAALTALTLLRGPPVAELAAGAVGGPVVRCEPCDARAVSQCAPPPTAPACT

ELVREPGCGCCLTCALREGDACGVYTERCGTGLRCQPRPAEQYPLRALLNGRGFCANASA

AGSLSTYLPSQPAPGNISESEEEHNAGSVESQVVPSTHRVTDSKFHPLHAKMDVIKKGHA

RDSQRYKVDYESQSTDTQNFSSESKRETEYGPCRREMEDTLNHLKFLNVLSPRGVHIPNC

DKKGFYKKKRCRPSKGRKQSFCWCVDKYGQRLPGYDTKGKDDVHCLSVQSQ

>Rattus norvegicus IGFBP3

VRCEPCDARALAQCAPPPTAPACTELVREPGCGCCLTCALREGDACGVYTERCGTGLRCQ

PRPAEQYPLKALLNGRGFCANASAASSLSAYLPSQPSPGNTTESEEDHNAGSVESQVVPS

THRVTDSKFHPLHSKMEVIIKGQARDSQRYKVDYESQSTDTQNFSSESKRETEYGPCRRE

MEDTLNHLKFLNVLSPRGVHIPNCDKKGFYKKKQCRPSKGRKRGFCWCVDKYGQPLPGYD

TKGKDDVHCLSVQSQ

>Danio rerio IGFBP3

MTGLCALCLTALLAAFARLAESVSPVVRCEPCDDGAMVLCKPLPWDCDEPVKEPGCGCCL

TCPLTEGQACGVYTGRCGTGLSCQHRPGESKPLQALLEGRGVCAKAPDKKQSGSPSHGHD

KPETEGKEQNGTRTAGTGEAETVHHTTDISRDVQGGSRTPSEPSDPMMHSNKLEMIQKEQ

VKKSQVHKVVPFSGWIVQDIHNFSLESKRENEYGPCRREMESVMKQLKFTNVLNPRRFRI

PNCDQKGFYKKKQCSPSKGRKRGHCWCVDKYGQPLPGYDGKEKVHCYNMETK

> Takifugu rubripes IGFBP3

SGAVGPVIKCEPCDAGARLLCKPLPKDCVEKVREPGCGCCLTCALRFGQPCGVYTSRCGS

GLTCQHQPGETKPLQALLEGRGICVNATNKRLTPRPTAPVNELPAENIETQDEERNSTSS

GLQTPDSTHRPMGSPRPPLNPFLPSAKSDVLRREQKRIQSFKMEELPGPLITDQQNFSVE

TKQEPDYGPCRREIESILSGLKITDILNPRGFRIPNCDKKGFYKKKQCRPSKGRKRGFCW

CVDKYGQPLPGFDGKERGDAQYYNSETQ

>Tetraodon nigroviridis IGFBP3

SGAVGPVIKCEPCDAGARLLCKPLPKDCVEKVREPGCGCCVTCALPFGQPCGIYTSRCGS

GLTCQHQPGETKPLQALLEGRGICVNATNKRLTPKPTPPVNELPAENIETQEEERNSTSS

GLQTPDSTHRPMGSPRPPLNPFLPSAKSDVLRREQKRTQSFKMEELPGPLITDQQNFSVE

TEQEPDYGPCRREIESILSGLKITDILNPRGFRIPNCDKKGFYKKKQCRPSKGRKRGFCW

CVDKYGQPLPGFHGKERADAQYYNSETQ

>Homo sapines IGFBP5

MVLLTAVLLLLAAYAGPAQSLGSFVHCEPCDEKALSMCPPSPLGCELVKEPGCGCCMTCA

LAEGQSCGVYTERCAQGLRCLPRQDEEKPLHALLHGRGVCLNEKSYREQVKIERDSREHE

EPTTSEMAEETYSPKIFRPKHTRISELKAEAVKKDRRKKLTQSKFVGGAENTAHPRIISA

PEMRQESEQGPCRRHMEASLQELKASPRMVPRAVYLPNCDRKGFYKRKQCKPSRGRKRGI

CWCVDKYGMKLPGMEYVDGDFQCHTFDSSNVE

>Mus musculus IGFBP5

MVISVVLLLLAAYAVPAQGLGSFVHCEPCDEKALSMCPPSPLGCELVKEPGCGCCMTCAL

AEGQSCGVYTERCAQGLRCLPRQDEEKPLHALLHGRGVCLNEKSYGEQTKIERDSREHEE

PTTSEMAEETYSPKVFRPKHTRISELKAEAVKKDRRKKLTQSKFVGGAENTAHPRVIPAP

EMRQESEQGPCRRHMEASLQEFKASPRMVPRAVYLPNCDRKGFYKRKQCKPSRGRKRGIC

WCVDKYGMKLPGMEYVDGDFQCHAFDSSNVE

>Rattus norvegicus IGFBP5

MVISVVLLLLAACAVPAQGLGSFVHCEPCDEKALSMCPPSPLGCELVKEPGCGCCMTCAL

AEGQSCGVYTERCAQGLRCLPRQDEEKPLHALLHGRGVCLNEKSYGEQTKIERDSREHEE

PTTSEMAEETYSPKVFRPKHTRISELKAEAVKKDRRKKLTQSKFVGGAENTAHPRVIPAP

EMRQESDQGPCRRHMEASLQEFKASPRMVPRAVYLPNCDRKGFYKRKQCKPSRGRKRGIC

WCVDKYGMKLPGMEYVDGDFQCHAFDSSNVE

>Xenopus tropicalis IGFBP5

MEMLVPALLLVSLCLGQCQALGSFVHCEPCDDKAMSMCPPTPVGCELVKEPGCGCCMTCA

LAEGHRCGVYTEHCAKGLRCLPEQGEEKPLHALLHGRGVCLNLKNHRDQSKIGSQNSIEE

PTTSETDDLYPSKHRGKMRLTDQKAIALNTFRQKKQSQSRIVSVEKVQSPSTPEHKMVPI

VAGPCRRQVETLMQEMKLSHRVYPRAFYLPNCDRKGFFKRKQCKPSRGRKRGLCWCVDKY

GLKLPGIDYVNGDLQCHSFDSSNTE

>Tetraodon nigroviridis IGFBP5

MLLRVSLLLPLLCLRGGGASFVPCQPCDQKALSMCPPVPVGCQLRARSKRASRAACTPGP

CTRGLRCLPKNGEEKPLHALLHGRGACRNEKLYKLMHPSKEGDPRDESMLPVPDFQTKVP

LYGRDHVNSRKVQAMKQAKDRRKQLARPGTGSLDFSPLSLDKLEPDFSVVQGPCRRRLDN

LLQSMKDTSRVLALSLYIPNCDKKGFFKRRQCKPSRGRRRGICWCVDRFGVKIPGINYAG

GDLQCKEHESSSN

> Danio rerio IGFBP5

MALLVLGTFLTVLSVSGGSFVPCEPCDQKALSMCPPVPVGCQLVKEPGCGCCYTCALAEG

QACGVYTGTCTHGLRCLPRNGEEKPLHALLHGRGVCTNEKGYKPPHPPIDRESIEHDDTV

KPDTTEDQIPKIPLYPKPDVINSKKQAALFKDKKKQQEKLRSVGSLDYSPLPIDKHEPEF

GPCRRKLDGIIQSMKDTSRVMALSLYLPNCDRKGFFKRKQCKPSRGRKRGICWCVDKYGV

QLPGTDYSGGNIQCKDLENSNNNNE

>Takifugu rubripes IGFBP5

MLLRVSLLLPLLCLRGCGASFVPCQPCDQKAQSMCPPVPLGCQLRARSKRASRAACTPPC

TRGLRCLPKNGEEKPLHALLHGRGACRNEKLYKLMHPSKDGEARDASMLPVPDFQTKVPL

YGRDHINSRKVQAMKQAKDRKKQLARLGTGNLDFSPLSVDKLEPDFVSEFGGPCRIRLDN

LLQSMKDTSRVLALSLYIPNCDKKGFFKRRQCKPSRGRRRGICWCVDRFGVKIPGINYAG

GDLQCKEHESSSN

>Homo sapines IGFBP6

MTPHRLLPPLLLLLALLLAASPGGALARCPGCGQGVQAGCPGGCVEEEDGGSPAEGCAEA

EGCLRREGQECGVYTPNCAPGLQCHPPKDDEAPLRALLLGRGRCLPARAPAVAEENPKES

KPQAGTARPQDVNRRDQQRNPGTSTTPSQPNSAGVQDTEMGPCRRHLDSVLQQLQTEVYR

GAQTLYVPNCDHRGFYRKRQCRSSQGQRRGPCWCVDRMGKSLPGSPDGNGSSSCPTGSSG

>Mus musculus IGFBP6

MTWDGLPTQPLLMLLMLLFAAGSGSALAGCPGCGPGMQTGCRGGCVEEEDAGSPADGCTE

AGGCLRREGQPCGVYSPKCAPGLQCQPRENEETPLRALLIGQGRCQRARGPSEETTKESK

PQGGASRSRDTNHRDRQKNPRTSAAPIRPNPVQDSEMGPCRRHLDSVLQQLQTEVFRGGA

RGLYVPNCDLRGFYRKQQCRSSQGNRRGPCWCVDPMGQPLPVSPDGQGSTQCSARSSG

>Rattus norvegicus IGFBP6

MTWDGLPTQPLLMLLMLLFAAGSESALAGCPGCGPGVQEEDAGSPADGCAETGGCFRREG

QPCGVYIPKCAPGLQCQPRENEETPLRALLIGQGRCQRARGPSEETTKESKPHGGASRPR

DRDRQKNPRTSAAPIRPSPVQDGEMGPCRRHLDSVLQQLQTEVFRGGANGLYVPNCDLRG

FYRKQQCRSSQGNRRGPCWCVDPMGQPLPVSPDGQGSSQCSARSSG

>Ciona intestinalis IGFBP MVHQNIISSLLFMLWTVIVKCHDVSICVDLCPACFTQRMQNCPPLPDCELKVRDPGCGCC

FTCAAGAGDRCGINKPRCGPGLKCLAVGAESNLIAILENRGACQLDPAHQETTEKASRLR

LIHTRHLKRRRRKKHRRFPRHPHRGLLSPLNHDGPCSRHYEEMMTSCITMAHKVWLPACD

ARGYYAAKQCMSSNGLERGKCWCVDETGIALEDTPYTRDSITCHR

>Ciona savignyi IGFBP

MYILFAIVAVALIGDPAASAPASRFAMKCPVCSAEELNACPVLEAGPTCEIVAEPNCGCC

SMCAKLESEECGVAKGYCGSGLTCAPLIYEYEGEVYFSEHVCMTDADLDRMFGEVITPPL

EDPESHQNGEAQDTTLSPTTTTVIPTQEAHVPEGPCDQHWERVWSKTFFAKHEWVPECDR

EGYYSPQQCEVAYGLELGKCWCVDKLGNRQTDKMVDSPSFQC

>Strongylocentrotus purpuratus (XP_793433)

MAAIDSSCFLEHKLRQNSRAITGTLLAILIMLSDASVSFATRLNPENTQTQNPECNMDCS

NQRPKPFCGTDGRTYLSKCEVKKARCQGWNVRKEHNGPCAVELTKCQLERAQNLQQPDSS

SLFVPQCNDDGSYAMIQCHKSFGYCWCVTEEGKPIAGSSVREGNPTCNRQADEPIVDNPP

TFKGTSSSSGGSRCRQDKREEFNKNLKRIFKEEYARLPRADIAPIPFSSSRATLEWEKQV

IEWKFGSLDENEDANLDSKELKPLLRLMKKIIKPKVCAKDFVKICDLDADDRLTEMEWSL

CLGVDNNEQPEEPTVDSGGPQDASPPLLVTRQIPHGDNADSDNQNVPVLSCETERLRALE

EQERTSASVVFVPQCTITGDYMAVQCHEQMQYCWCVYVDTGKPIPGTSVRDSTKEDCSPL

ATSRGPITPRVFVDCPDDKKKRFLIKLIEKLYAEMTVTDALLIETTTQAAEDLSPNHKDE

SIVWKFGQLDRNGNNYLEAKETATFATEIDLIKSSKKCRNNFLDFCDANQDSRLALKEWL

HCFDITPPVATEAPPRRGRNPFIDRLT

>Drosophila melanogaster(CG13830-PA)

MKHSPLIASACLALVLMSSSLIGSTEARNKKKYVGETGGDFEFIDEINKNTQSNKNLGEH

KRWIHDPSSDLCRPLNCKKREICLLEDEFSAVCVSKKELHKNRDEIITKAKYLEEEAKRR

VNQQDNQDSQDAEDINNDDEDNSSDGGSSNSSPTGTNNAQASVQGNEETDDEDKSLSLGD

DDESKEDDVFYENSIAAGDKQQQQQQLSQPAAGPSVIQQDDDEELDNCKPCPVAKPTFLC

GADNRTYSSLCRLDYHNCIHSTSIRIACKGFCPCKEIVDGKRLQRISGYNNKYNKKISLD

QQQQQQQQQQQQQQQQQAYKDSNNNNIMMNSGNIMGGNNNDFNTIMNLNFWYWYCRTKRT

ITGTTTTSMPSTLSRPRRSNMTTSTTSTSSTRPTRRTPSTRRTSTRCATITRWSRSSSRT

YPSKSAECKPQQLTAIGNRLLDWFSVIMADSKKRRQHSQKSKAHFPPACKTEAKWMFGHL

DLNNDGQLSLQEMYDLEHDQNERCIKPFIDTCDLDTDSSINTREWCRCFEKTDRPCAAVR

RRIAGDFAGAYAPDCDIQGFYKPTQCHNSVGVCWCVDKHGVEFANTRTRGKPNCAFKPAF

MAQCWHNSAAAMLEPANGSENTSHSESVVNNAASLTSDDEDEGADDEDSAEGSADQMLVF

>Appis mellifera (XP_393267)

MTLLTLHVLRCRGLNKLANPLRLTITGSLIKSRSCSVFKKYAKKMSYTTIERGALNSTDY

RIYFRNDVGPISPMHDIPLYADESNKILNMVVEIPRWTNAKMEINLKETLNPIKQDVKKG

KLRYVANCFPHHGYIWNYGALPQTWENPEVLDEATGCKGDNDPIDVLEIGYKVAKRGEIL

KVKVLGCVALIDEGETDWKIIVIDVNDPLAEQMNDVSDIEKHYPGLMKATIEWFKIYKIP

DGKPENQFAFNGEAKSRDFALHIVEEVHQHWQNLIKREAPAGGIACTNTTVSGSPFKTTI

ETAEEVLEKAAELSEPQSVDPIGELCRPLNCKKKDLCLLKDTFTAVCVSKKELEKSGDIV

IPKSRAVQQNRRMRTETSVDTEDDDAFFDSEDDDEDQDESKCQECPVVKPTFLCGSDNRT

YSSPCRLEYHNCIHHTSVRIACKGFCPCKASDLRAHNKKMQLQRQKALSGKMRNRDITLT

PHVFNYDNHHYQYLKYSKRAKALAGSKYDDKHLMSNEVMEKTPSPLKSTYNSQSSRNSDC

PPSSKPAMANRLLDWFSVVMADSKHRRPHPKPKGHFPIGCQSEVRWMFGHLDSDNDGRLS

LSELYGLEHDQNEPCLKPFLDGCDTDRDIFVSGPEWCGCFSKAERPCAAVRKRSSPDVAP

ACDSRGYYRSTQCHRGLGLCWCVDPHGVEFAGTRTRGSKPDCDTIVNKISNGGLKTNSVD

LDDDEDGGTDSAQDLEGSADQPLDF

**Inhibin family**

>Homo sapiensINHBA

MPLLWLRGFLLASCWIIVRSSPTPGSEGHSAAPDCPSCALAALPKDVPNSQPEMVEAVKK

HILNMLHLKKRPDVTQPVPKAALLNAIRKLHVGKVGENGYVEIEDDIGRRAEMNELMEQT

SEIITFAESGTARKTLHFEISKEGSDLSVVERAEVWLFLKVPKANRTRTKVTIRLFQQQK

HPQGSLDTGEEAEEVGLKGERSELLLSEKVVDARKSTWHVFPVSSSIQRLLDQGKSSLDV

RIACEQCQESGASLVLLGKKKKKEEEGEGKKKGGGEGGAGADEEKEQSHRPFLMLQARQS

EDHPHRRRRRGLECDGKVNICCKKQFFVSFKDIGWNDWIIAPSGYHANYCEGECPSHIAG

TSGSSLSFHSTVINHYRMRGHSPFANLKSCCVPTKLRPMSMLYYDDGQNIIKKDIQNMIV

EECGCS

>Mus musculusINHBA

MPLLWLRGFLLASCWIIVRSSPTPGSEGHGSAPDCPSCALATLPKDGPNSQPEMVEAVKK

HILNMLHLKKRPDVTQPVPKAALLNAIRKLHVGKVGENGYVEIEDDIGRRAEMNELMEQT

SEIITFAESGTARKTLHFEISKEGSDLSVVERAEVWLFLKVPKANRTRTKVTIRLFQQQK

HPQGSLDTGDEAEEMGLKGERSELLLSEKVVDARKSTWHIFPVSSSIQRLLDQGKSSLDV

RIACEQCQESGASLVLLGKKKKKEVDGDGKKKDGSDGGLEEEKEQSHRPFLMLQARQSED

HPHRRRRRGLECDGKVNICCKKQFFVSFKDIGWNDWIIAPSGYHANYCEGECPSHIAGTS

GSSLSFHSTVINHYRMRGHSPFANLKSCCVPTKLRPMSMLYYDDGQNIIKKDIQNMIVEE

CGCS

>Rattus norvegicus INHBA

MPLLWLRGFLLASCWIIVRSSPTPGSEGHGAAPDCPSCALATLPKDGPNSQPEMVEAVKK

HILNMLHLKKRPDVTQPVPKAALLNAIRKLHVGKVGENGYVEIEDDIGRRAEMNELMEQT

SEIITFAESGTARKTLHFEISKEGSDLSVVERAEVWLFLKVPKANRTRTKVTIRLFQQQK

HPQGSLDMGDEAEEMGLKGERSELLLSEKVVDARKSTWHIFPVSSSIQRLLDQGKSSLDV

RIACEQCQESGASLVLLGKKKKKEVDGDGKKKDGSDGGLEEEKEQSHRPFLMLQARQSED

HPHRRRRRGLECDGKVNICCKKQFFVSFKDIGWNDWIIAPSGYHANYCEGECPSHIAGTS

GSSLSFHSTVINHYRMRGHSPFANLKSCCVPTKLRPMSMLYYDDGQNIIKKDIQNMIVEE

CGCS

>Canis familiaris INHBA

CALTALPRDAPNSQPEMVEAVKKHILNMLHLKKRPEVTQPVPKAALLNAIRKLHVGKVGE

NGFVEIEDDIGRRAEMNELMEQTSEIITFAESGTARKTLHFEISKEGSDLSVVERAEVWL

FLKVPKANRTRTKVTIRLLQKHPQGSLDAGEEAEDMGFPEERNEVLISEKVVDARKSTWH

IFPVSSSIQRLLDQGRSSLDVRIACEQCHETGASLVLLGRAGGDEDKEQSHRPFLMLQAR

QSEDHPHRRRRRGLECDGKVNICCKKQFFVSFKDIGWNDWIIAPSGYHANYCEGGCPSHI

AGTSGSSLSFHSTVINHYRLRGHSPFTNLKSCCVPTKLRPMSMLYYDDGQNIIKKDIQNM

IVEECGCS

>Bos taurus INHBA

MPLLWLRGFLLASCWIIVRSSPTPGSEGHSAAPDCPSCALAALPKDVPNSQPEMVEAVKK

HILNMLHLKKRPDVTQPVPKAALLNAIRKLHVGKVGENGYVEIEDDIGRRAEMNELMEQT

SEIITFAESGTARKTLHFEISKEGSDLSVVERAEVWLFLKVPKANRTRTKVTIRLFQQQK

HPQGSLDTGEEAEEVGLKGERSELLLSEKVVDARKSTWHVFPVSSSIQRLLDQGKSSLDV

RIACEQCQESGASLVLLGKKKKKEEEGEGKKKGGGEGGAGADEEKEQSHRPFLMLQARQS

EDHPHRRRRRGLECDGKVNICCKKQFFVSFKDIGWNDWIIAPSGYHANYCEGECPSHIAG

TSGSSLSFHSTVINHYRMRGHSPFANLKSCCVPTKLRPMSMLYYDDGQNIIKKDIQNMIV

EECGCS

>Monodelphis domestica INHBA

MPGLSLRGFLLALCWIRVRSSPTPGSEGASPGPVSECPSCALSSLPKEMPSSQPEMVEAV

KKHILNMLHLKKRPDVTQPVPKAALLNAIKKLHVGKVGENGYVEIEDDIGRRAEMNELLE

QTSEIITFAESGTPKKTLHFEISKEGSDLSVVERAEIWLFLKVPKANRTRTKVTIRLYQN

QKQSQSSLSDEAEVVGIKGERSEQLISEKAVDARKSTWHIFPVSSSIQRLLDQGKSSLDV

RIVCDQCQETGANLVLSQTHRPFLMMQARQSEDHPHRRRRRGLECDGKVNICCKKQFFVS

FKDIGWNDWIIAPTGYHANYCEGECPSHIAGTSGSSLSFHSTVINHYRMRGHSPFSNLKS

CCVPTKLRPMSMLYYDDGQNIIKKDIQNMIVEECGCS

>Erinaceus europaeus INHBA

MPLLWLRGFLLASCWIIGRSSPTPGSEGQGAAPDCPSCALATLPKDGPNSQPEMVEAVKK

HILNMLHLKKRPDVTQPVPKAALLNAIRKLHVGKVGENGFVEIEDDIGRRAEMNELMEQT

SEIITFAESGSAKKTLHFEISKEGSDLSVVERAEIWLFLKVPKAHRNRTKVTIRLFQQQK

QLQGSLDTGEEAEEVGFMERSELLISEKVVDARKSTWHIFPVSNSIQRLLDQGRSSLDIR

IACEQCHETGASLVLLGKKKKKEEEGEGKRKDKKDGEGGAGGEEDKEQSHRPFLMLQARQ

SEDHPHRRRRRGLECDGRVNICCKKQFYVSFKDIGWNDWIIAPSGYHANYCEGECPSHIA

GTSGSSLSFHSTVINHYRMRGHSPFANLKSCCVPTKLRPMSMLYYDDGQNIIKKDIQNMI

VEECGCS

>Gasterosteus aculeatus INHBA

ILNIFPQSNSPLLYITCTTKCLLHVDVASRLQEVAPSSDCPSCSLLQMRINSSSPGGQSD

MVEAVKRHILNMLHLSTRPNLTQPVPRAALLNAIKKLHVGRVAKDGSVEIQEMGRGPWAQ

TPPHPPSEIITFAEPGDLVSTVTFDISKEGTSLSVVEQANVWIFLKISTANRGKGKVMLR

LLQLPYEDVGEKEECVSEKMVDTRRSGWHTLTVSRTVQALLEGGRSLLSLRVSCTLCTEA

GASPVLMPAMGEHPTGRYQSHRPFLMVVLRAEEEATQRRVKRGLECDGKIRVCCKRQFYV

NFKDIGWNDWIIAPSGYHANYCEGECPSHMASFSGSSLSFHSTVINHYRMRGYSPFQNMK

SCCVPTRLRAMSMLYYNEEQKIIKKDIQDMIVDECSCS

>Oryzias latipes INHBA

LLSWALLLLVQVRGSGSTSDPASMLQPLSLSVHPHQKLQLDTSSSSHCPSCALARMRRNE

GVMAGDEDVVEAVKRHILNMLHLQERPNITRPVPRAALLNALRKLHVGRVAEDGSVQIGG

EDESISEPSSEPFSSPPSQFVSGLLPGLSPHTETFEVQEGGDMSLVEQATVWLFLRMVKT

NRSRAKVTIRLFQQRRLPNGRLSQPQDDILLAEKKVDTRRSGWHTFAVSAAVQALLESSE

GSTLSLRMSCPLCADAGVTVVLVSGSTETLQRNNQREQSHRPFLMAVVRQGDSNDSRRRR

KRGLECDGKVRVCCKKQFYVNFKDIAWADWIIAPSGYHANYCEGDCPSHAASLPGSSLSF

HSTVISHYRMKENSPFQSLKSCCTPTKLRAMSMLYYDEKQRIVKKDIPNMIVEECGCS

>Tetraodon nigroviridis INHBA

MSPLAVLSWTLLLLVHSSVPLKSPPQDAPSCPSCALARVGRRGVQEGWEEAPQQDVVEAA

KRHILNMLHLQERPNVTRAVPRAALLHALRKLHVGHVARDGSVRIGGEEEEWRGWGGQTH

GGSTREAAGPGGADGAGDETEARETSEVIAFAEAGDSPGTVNFMLSKEGGALSLVKQADV

WLFLRLAKTNRSRTRVTIRLSQRGNFTDRPQEIFLAEKSVHVRRSGWHTFSVLAAVQLLL

ESQEKAVLSLRVSCPLCAGAGATPFLMCGNEREQSHRPFLAVVVQQEDGGDPRQRKKRGL

ECDEDVQVCCKRQLYIDFKDIGWNDWIIAPSGYHANYCEGECPSHAASLVGSTLSFHSTV

ISHYRMRGYSPFQNLRSCCVPARLRAMSMLYYNEEQKIIKKDIQNMVVEECGCS

>Danio rerio INHBA

MSPLPLLSGILLLLIRSCSLSAMVTKGSLPMSEQQAGATVCPSCALARFRKGVSESEDEG

AQQDVVEAVKRHILNMLHLQERPNITHPVPRAALLNAIRKVHVGRVAKDGSVLIEDEASN

RAETEQAEQTEIITFAETGEAPGIVNFLISKEGGEMSVVDQANVWIFLRLPKGNRTRANV

NIRLLLQQGAGEKILAEKSVDTRRSGWHTFPASESVQSLLQRGGSTLSLRVSCPLCADAR

ATPVLVSPGGSEREQSHRPFLMAVVRQMDELSLRRRRKRGLECDGKARVCCKRQFYVNFK

DIGWNDWIIAPSGYHANYCEGDCASNVASITGNSLSFHSTVISHYRIRGYSPFTNIKSCC

VPTRLRAMSMLYYNEEQKIVKKDIQNMIVEECGCS

>Takifugu rubripes INHBA

MAPLVVLSWTILLLVHTSVPLKSRPQDASSCPACALAQLGRSILEEESVVEPHQEMVEAV

KRHILNMLHLQNRPNITRPVPRAALLNALRKLHVGHVAGDGSVQISGEKEESQEWGRRTQ

GDIRGEAGGPGGAEERRGDKREAQETAEIITFAETGDSPGTLNFVLSQEGGAVSQLEQAN

VWLFLRLAKTNSSRTKVTIRLFTQRTRPDRPPPYEETFLVEKSVHARRSGWHTFSVLAAA

RAALQEAEDAVLRLRVSCPLCASAGASLLLASSGLEGNQQHQSHRPFLVALVQQDSGDWR

RRRKRGLECDEEVQVCCKRQLYINFKDIGWNDWIIAPSGYHANYCEGECPSHVAGVASST

LSFHATVISHYRMRGYSPFQNLHSCCIPTRLRAMSMLYYNEEQKIIKKDIQNMVVEECGC

S

>Homo sapiens INHBB

MDGLPGRALGAACLLLLAAGWLGPEAWGSPTPPPTPAAPPPPPPPGSPGGSQDTCTSCGG

FRRPEELGRVDGDFLEAVKRHILSRLQMRGRPNITHAVPKAAMVTALRKLHAGKVREDGR

VEIPHLDGHASPGADGQERVSEIISFAETDGLASSRVRLYFFISNEGNQNLFVVQASLWL

YLKLLPYVLEKGSRRKVRVKVYFQEQGHGDRWNMVEKRVDLKRSGWHTFPLTEAIQALFE

RGERRLNLDVQCDSCQELAVVPVFVDPGEESHRPFVVVQARLGDSRHRIRKRGLECDGRT

NLCCRQQFFIDFRLIGWNDWIIAPTGYYGNYCEGSCPAYLAGVPGSASSFHTAVVNQYRM

RGLNPGTVNSCCIPTKLSTMSMLYFDDEYNIVKRDVPNMIVEECGCA

>Mus musculus INHBB

PGAPGGSQDTCTSCGGGGGGFRRPEELGRVDGDFLEAVKRHILSRLQLRGRPNITHAVPK

AAMVTALRKLHAGKVREDGRVEIPHLDGHASPGADGQERVSEIISFAETDGLASSRVRLY

FFVSNEGNQNLFVVQASLWLYLKLLPYVLEKGSRRKVRVKVYFQEQGHGDRWNVVEKKVD

LKRSGWHTFPITEAIQALFERGERRLNLDVQCDSCQELAVVPVFVDPGEESHRPFVVVQA

RLGDSRHRIRKRGLECDGRTSLCCRQQFFIDFRLIGWNDWIIAPTGYYGNYCEGSCPAYL

AGVPGSASSFHTAVVNQYRMRGLNPGPVNSCCIPTKLSSMSMLYFDDEYNIVKRDVPNMI

VEECGCA

> Rattus norvegicus INHBB

MDGLPGRALGAACLLLLAAGWLGPEAWGSPTPPPSPAAPPPPPPPGAPGGSQDTCTSCGG

GGGGFRRPEELGRVDGDFLEAVKRHILSRLQLRGRPNITHAVPKAAMVTALRKLHAGKVR

EDGRVEIPHLDGHASPGADGQERVSEIISFAETDGLASSRVRLYFFVSNEGNQNLFVVQA

SLWLYLKLLPYVLEKGSRRKVRVKVYFQEQGHGDRWNVVEKKVDLKRSGWHTFPITEAIQ

ALFERGERRLNLDVQCDSCQELAVVPVFVDPGEESHRPFVVVQARLGDSRHRIRKRGLEC

DGRTSLCCRQQFFIDFRLIGWNDWIIAPTGYYGNYCEGSCPAYLAGVPGSASSFHTAVVN

QYRMRGLNPGPVNSCCIPTKLSSMSMLYFDDEYNIVKRDVPNMIVEECGCA

>Monodelphis domestica INHBB

MDGLPGRALGAACLLLLVACWLGPEAWGSPTPLPSPAAPPGAEGAPPDTCTSCGFRRPEE

PGKVDGDFLEAVKRHILSRLQMRGRPNITHAVPKAAMVTALRKLQAGKVRKTAEWRSLRP

DNLSPGIPGPREVSVFPPFSTDDLASSRVRLYFFISNEGNQNLFVVQASLWLYLKLLPYV

LEKGTRRKVRVRVYFQDLDRSDKWNVVEKKVDLKRSGWHTFPLTEAIQALFEKGERRLNL

ELQCEGCQELAVVPVFVDPGEESHRPFLVVQARLADNKHRIRKRGLECDGRTNLCCRQQF

FIDFRLIGWNDWIIAPTGYYGNYCEGSCPAYLAGVPGSASSFHTAVVNQYRMRGLNPGTV

NSCCIPTKLSTMSMLYFDDEYNIVKRDVPNMIVEECGCA

>Erinaceus europaeus INHBB

GLTSSRVRLYFFISNEGNQNLFVVQASLWLYLKLLPYVLEKGTRRKVRVKVYFQEQGHSE

RWKVVEKKVDLKRSGWHTFPLTEAIQSLFERGERRLNLDVQCDGCPDLAVVPVFVDPGEE

SHRPFVVVQARLGDSRHRIRKRGLECDGRTNLCCRQQFFIDFRLIGWNDWIIAPTGYYGN

YCEGSCPAYLAGVPGSASSFHTAVVNQYRMRGLNPGTVNSCCIPTKLSTMSMLYFDDEYN

IVKRDVPNMIVEECGCA

>Tetraodon nigroviridis INHBB

MSSCLFRLALCVACLLSIRCTSAPPTQAGTHPASRDTCASCGIAQPQDPESGQLNVDFLE

AVKRHILSRLQMRERPNITQPVSRAAMVTALRKLHAGKMRADGRMEIPNLDGHPMGDDVL

EESSEIISFAEKDEMVTSKSSLFFLITNEGNQNLHVMQATLWLYFKVLPAPAEARSRRKV

TVKVYYQEPGLGSRWDLVEKRVELKRSSWHTFVLTDAVRLVFQKGDRRQNLDVRCEGCEA

EGVVPVLLHQKDESHRPFLVVQARQADSKHRIRKRGLECDGSSSLCCRQQFYIDFRLIGW

NDWIIAPSGYFGNYCEGNCPAYMPGVPGSASSFHTVVVNQYRLRGMGPGSMNSCCIPTKL

STMSMLYFDDEYNIVKRDVPNMIVDECGCA

>Danio rerio INHBB

MDTIFMKMSVSILSVTCLMACLLSVQCSSLGAETGSQESQCVSCGLGHQEDSGRMDTDFL

EAVKRHILNRLQMRERPNITHPIPKAAMVTALRKLHAGKVREDGRVEIPNLDGHAAHNEV

QEETSEIISFAESDDVTPSKSSLYFLISNEGNQNLYVLQANLWLYFKLMPGTLEKGLRRK

VTVRVHSYEPGGQNVHWPMMEKRVELKRSGWHTFPVSEAIREMLAKGGRRQDLDIHCEGC

EAANVLPILVDPSDPSHRPFLVVRAQQADGKHRIRKRGLECDGNNGGLCCRQQFYIDFRL

IGWNDWIIAPAGYYGNYCEGSCPAYMAGVPGSASSFHTAVVNQYRMRGMSPGSVNSCCIP

TKLSTMSMLYFDDEYNIVKRDVPNMIVEECGCA

>Gasterosteus aculeatus INHBB

AEKGLRRKVTVKIHYQEPGAAGGAEAGPGGGGGGGGGTGRWAVVEKRVDLKRSGWHTFPL

SEAVRAVFGKGNRRQDLEVHCEGCETAGVSPIMVDPSDPSHRPFLVVRVRQVDGKHRIRK

RGLECDGTSGGLCCRQQFYIDFRLIGWNDWIIAPAGYYGNYCEGSCPAYMAGVPGSASSF

HTAVVNQYRMRGMSPGSVNSCCIPTKLSTMSMLYFDDEYNIVKRDVPNMIVEECGCA

>Oryzias latipes INHBB

LECDGSSDLCCRQQFYIDFRLIGWNDWIIAPSGYFGNYCEGDCPPYMAGVPGSASSFHTA

VVNQYRMRGKSPVSMNSCCIPTKLSTMSMLYFDDEYNIVKRDVPNMIVDECGC

>Homo sapiens INHBC

MTSSLLLAFLLLAPTTVATPRAGGQCPACGGPTLELESQRELLLDLAKRSILDKLHLTQR

PTLNRPVSRAALRTALQHLHGVPQGALLEDNREQECEIISFAETGLSTINQTRLDFHFSS

DRTAGDREVQQASLMFFVQLPSNTTWTLKVRVLVLGPHNTNLTLATQYLLEVDASGWHQL

PLGPEAQAACSQGHLTLELVLEGQVAQSSVILGGAAHRPFVAARVRVGGKHQIHRRGIDC

QGGSRMCCRQEFFVDFREIGWHDWIIQPEGYAMNFCIGQCPLHIAGMPGIAASFHTAVLN

LLKANTAAGTTGGGSCCVPTARRPLSLLYYDRDSNIVKTDIPDMVVEACGCS

>Mus musculus INHBC

MASSLLLALLFLTPTTVVNPKTEGPCPACWGAIFDLESQRELLLDLAKKSILDKLHLSQR

PILSRPVSRGALKTALQRLRGPRRETLLEHDQRQEEYEIISFADTDLSSINQTRLEFHFS

GRMASGMEVRQTRFMFFVQFPHNATQTMNIRVLVLRPYDTNLTLTSQYVVQVNASGWYQL

LLGPEAQAACSQGHLTLELVPESQVAHSSLILGWFSHRPFVAAQVRVEGKHRVRRRGIDC

QGASRMCCRQEFFVDFREIGWNDWIIQPEGYAMNFCTGQCPLHVAGMPGISASFHTAVLN

LLKANAAAGTTGRGSCCVPTSRRPLSLLYYDRDSNIVKTDIPDMVVEACGCS

> Rattus norvegicus INHBC

MASSLLLALLFLTLATVVNLKTDGPCPACWGATFDLESHRELLLDLAKKSILDKLHLSQR

PILSRPVSREALKTALRRLRGTRAETLLEHDQRQEYEIISFADTGLSNINQTRLEFHFSD

RTTGGVEVLQTRFMFFMQLPPNTTQTMNIRVLVLRPYDTNLTLTSQYMLQVDASGWYQLL

LGPEAQAACSQGHLTLELVPESQLAHSSLILDGVSHRPFVAAQVRVEGKHRVRRRGINCQ

GLSRMCCRQEFFVDFREIGWHDWIIQPEGYAMNFCTGQCPLHVAGMPGISASFHTAVLNL

LKANTDAGTARRGSCCVPTSRRPLSLLYYDRDSNIVKTDIPDMVVEACGCS

>Canis familiaris INHBC

MTSSLLLAFLLLAPTTVATPRAGGQCPACGGPTLELESQRELLLDLAKRSILDKLHLTQR

PTLNRPVSRAALRTALQHLHGVPQGALLEDNREQECEIISFAETGLSTINQTRLDFHFSS

DRTAGDREVQQASLMFFVQLPSNTTWTLKVRVLVLGPHNTNLTLATQYLLEVDASGWHQL

PLGPEAQAACSQGHLTLELVLEGQVAQSSVILGGAAHRPFVAARVRVGGKHQIHRRGIDC

QGGSRMCCRQEFFVDFREIGWHDWIIQPEGYAMNFCIGQCPLHIAGMPGIAASFHTAVLN

LLKANTAAGTTGGGSCCVPTARRPLSLLYYDRDSNIVKTDIPDMVVEACGCS

>Bos taurus INHBC

MICSLFLAFLVLAAAMVATPRADRQCPACGEPALDVESHRELLLNLAKRSILDKLHLSQR

PTLGRPVSGVALRAALHRLHGPPQGALPEADEGQEYEIISFAETGSKPPPAQALPYKFRL

DQWAHENLEVQQASLMFFVQLPPNTTCPLKVRVLELSPRDTNLTSATQHLLQVDDTGWHQ

LLLGPEAQTAYSQGHLALELAPEEQVDWSPVVLARAAHRPFVTARVRVGGQHRVRRRGID

CQGRSKMCCRQEFFVDFREIGWHDWIIQPEGYAMNFCTGQCPLHVAGMPGIAASFYTSVL

NLLKVNTAAGTTRGSSCCVPTVRRPLSLLYYDRDSNIVKTDIPDMVVEACGCS

>Monodelphis domestica INHBC

LKMVSTLLLSLLLLIPAAAGTEWIDGQCPACGVPGSDPERQREILLNLAKQNILDKLHLT

QRPTLIQPVSKATLRTALRRLHGLQGGRIVPGSTLGGSGTGVEEQEYEIISFAETGYSTT

NRTVLDFQLSPDQSSGSLEVLQARLGFFLKIPPNGTWTMHVRVLGPGPRDTNLTLTTQHQ

LEVDASGWHQFLLGPEAQAAYGQGHLTLELEAKGWGAQGPVFLEKDAHQPFIVAQVRIGE

KHRIHRRGIECQGGSQMCCRQEFFVDFREIGWNDWIIQPEGYAMNFCTGQCPLHVAGMPG

IAASFHTAVLNLLKANGAPGTTGGGSCCVPTARRPLSLLYYDRDSNIVKTDIPDMVVEAC

GCS

>Erinaceus europaeus INHBC

MMSSLLPTFLCLALATLATSRADHQCPACGGPSLDLKSQRELLLDLAKRSILDKLHLTQR

PDLSQPVSGAALRTALQRLQGPGRGTLPETDRGQEYEIISFAETALSNVNQNRLDHFSDR

AADSMKQQASLMFFVQLPPNATRTLQVKVLGLDLHDTNLSLATQHLLEVDASGWHQLLLG

PEAQNACSQGHLTLELIPEGQGAQSSVILGGAAHTPFVAARVRDSGKHRVRRRGLDCQGA

SRMCCRQEFFVDFREIGWHDWIIQPEGYAMNFCAGQCPLHVAGMPGIAASFHTAVLNLLK

ANTDSGTAGGGSCCVPTARRPLSLLYYDRDSNIVKTDIPDMVVEACGCS

>Oryzias latipes INHBC

LFLAPLLLKSLWVTGSPGCPSCGLPAMERDAEEKMMIEFAKQQLLEKLHLKERPNITQSV

PRVALLTALRKLHAGRVRQDGTLELENSSPTKDQGYEIVSFADIKRGDKASLSLTFQFLQ

ERGKSIQVLQSSLWIYSRSSQDPHRNSRLLARVFLSAEGGFSGSNRTLVIEKMLEVQENN

WHTFPITRTLQSFLDGGQRRLRLEVSCEEDGKNLCSLDTSDSPNQPFLVAQVRLRDDHSK

HSVRKRSLRCGNEVTVCCKRDFYIKFKDIQWDEWIIAPEGYHMNYCMGQCPQHLSGSPGI

ASSFHATIFSQLKVNGINTATASCCIPTERQPLSMVYFNSQHSIVKTDVPDMIVESCGCT

>Tertaodon nigroviridis INHBC

MVINSSFPLQGTSHVTPKDGQCPYHACTTVSVAIHNPAGLSVWVYQVDDSSHLEQLVDSC

CRQRKEASRLSVNVGQFQRAVDEGDLGGQAVLSVDLRPLLRGPSPGATLTAQVFLSAEEG

VSGLNRTLVMEKMLEVHKANWHTFPVTRTLQAFLDSNQQLVRLEVTCHQDGKNLCSLDGP

VDSPYQPFLVAQVRRDNPSKHLLRKRSLRCGEDVTVCCKREFYIKFKDIQWHDWIIAPEG

YHMNYCMGQCPQHLSGSPGIASSFHATIFSQLKVNGINTAVSSCCVPTERRPLSMVYFNS

QHNIIKTDVPDMIVESCGAQTALHHSAIGLFFLVLCLCCLDDSGAPWQTRDLQRHR

>Takifugu rubripes INHBC

LWVSGSPRCASCGFPALTKDAEEKLMIEVAKQQLLDKLHLKERPNITQAVPRAALLTALR

KLHSGRVRPNRTLEQENNLSKKDQSYEIVSFADIHEGDGGAQASLGLAFRFLQESGQSIQ

VLQSSLWIYARSSENPHRAPRLTAQVFLSADGGASGLNRTLVMEKMLEVHKGNWHTFPIT

RTLQAFLDSNQHQLRLEVTCDEDGKNLCSLDGPADSPYQPFLVAQVRLRDNHSKHLVRKR

SLRCGDDVTVCCKREFYIKFKDIQWHDWIIAPEGYHMNYCMGQCPQHLSGSPGIASSFHA

TIFSQLKVNGINTAVSSCCVPTERRPLSMVYFNSQHNIVKTDVPDMIVESCGCT

>Homo sapiens INHBE

MRLPDVQLWLVLLWALVRAQGTGSVCPSCGGSKLAPQAERALVLELAKQQILDGLHLTSR

PRITHPPPQAALTRALRRLQPGSVAPGNGEEVISFATVTDSTSAYSSLLTFHLSTPRSHH

LYHARLWLHVLPTLPGTLCLRIFRWGPRRRRQGSRTLLAEHHITNLGWHTLTLPSSGLRG

EKSGVLKLQLDCRPLEGNSTVTGQPRRLLDTAGHQQPFLELKIRANEPGAGRARRRTPTC

EPATPLCCRRDHYVDFQELGWRDWILQPEGYQLNYCSGQCPPHLAGSPGIAASFHSAVFS

LLKANNPWPASTSCCVPTARRPLSLLYLDHNGNVVKTDVPDMVVEACGCS

>Mus musculus INHBE

MKLPKAQLWLILLWALVWVQSTRSACPSCGGPTLAPQGERALVLELAKQQILEGLHLTSR

PRITRPLPQAALTRALRRLQPKSMVPGNREKVISFATIIDKSTSTYRSMLTFQLSPLWSH

HLYHARLWLHVPPSFPGTLYLRIFRCGTTRCRGFRTFLAEHQTTSSGWHALTLPSSGLRS

EDSGVVKLQLEFRPLDLNSTAAGLPRLLLDTAGQQRPFLELKIRANEPGAGRARRRTPTC

EPETPLCCRRDHYVDFQELGWRDWILQPEGYQLNYCSGQCPPHLAGSPGIAASFHSAVFS

LLKANNPWPAGSSCCVPTARRPLSLLYLDHNGNVVKTDVPDMVVEACGCS

>Rattus norvegicus INHBE

MGLSNVQLWTILLWALAWVQSTRSACPSCGAPTLTPQGERALVLELAKQQILEGLHLTSR

PRITRPLPQAALTRALRRLQPRSMVPGNREKVISFATSIDKSTSTYRSVLTFQLSPLWSH

HLYHARLWLHVPPSFPATLYLRIFGCGTTRCRGSRTFLADYQTTSSGWHALTLPSSGLRS

EESGVTKLQLEFRPLDLNSTTARLPRLLLDTAGQQRPFLELKIRANEPGAGRARRRTPTC

ESETPLCCRRDHYVDFQELGWRDWILQPEGYQLNYCSGQCPPHLAGSPGIAASFHSAVFS

LLKANNPWPAGSSCCVPTARRPLSLLYLDHNGNVVKTDVPDMVVEACGCS

>Canis familiaris INHBE

MGLPDVQPQLVLLWALVWAQGAGSGCPSCGGPTLAPQAERALVLELAKQQILEGLHLTSR

PRITHPPPQAALARALRRLQPRSMPIADGDEVISFATVAGGAGDTRRSPILADERVAGWA

CRAWSSATPLTCLPLAVSRADSTSACSSVLTFDLSTAQPHQLTAAGWHALALPSGGLRAE

ESAVLQLQLKCRLLPGNRTSAQQLGRRLLDTAGDRRPFLQLQIWPREPGAGRARRRTPTC

EPETPLCCRRDHYVDFQELGWRDWILQPEGYQLNYCSGQCPPHLAGSPGIAASFHSAVFS

LLKANNPWPLGTSCCVPTARRPLSLLYLDRDGNVVKTDVPDMVVEACGCS

>Erinaceus europaeus INHBE

MGLPDVQLPLMLLWALVWAQGAESACSSCGNPTLTPHVERALVLELAKQQILKGLQLPGR

PRVTHPPPQAVLSRALRRLRLGSAAPAHREEVLSFANRTDPAASTCSSALTFPLVTARSR

HLRRARLWLHVLPTLPATLHLRIFRRGPRSRRHRHRPLLAQHRVTAPGWHALTLPSSGLW

DEQPGVLKLQLDCSLEGHSTAFLQPLPLLDMAGDQRPFLELKTRPSGSRAGRVKRRKPTC

ESETPLCCRRDHYVDFRELGWRDWILQPEGYQLNYCSGQCPPHLAGSPGIAASFHSAVFN

LLKANNPWPKTTSCCVPTARRPLSLLYLDGNGNVVKTDVPDMVVEACGCS

>Strongylocentrotus purpuratus (NP_999793)

MDVSKVLILTLIWLLTADSAPPDYVTLTRKMESKILRVMGLSERPRPKPNATAPQYMWDL

YRQQMAATEGAAASRGGETEIGKEEEEDGRPCSETKLSSNIIRSVSHTGGDLRASNSTSL

QQILLFDVASIPHAETIEAADLRLEIPALPSATDVPSLAVRIYQLESRTRLNSIVSLKDK

RLRLLDVVLADLSQGYAGTIDILSTVNSWRSKKTSNHGLLLHVELMSTSGNNRRGSQVIK

ELGAISKKCTANLIVTSSEYRQCSKRNRRNKRQAESEAPADISSFPTASLTNLCQRHRLF

VSFRDVGWENWIIAPMGYQAYYCDGECPFPLGERLNGTNHAIIQTLVNSIDNRAVPKVCC

APTKLSGISMLYFDNNENVVLRQYEDMVVEACGCR

>Branchiostoma floridae(inhibinbeta)

DGRSNECCREKFYVDFKDIAWDDWIISPKGYYANFCTGSCQGTILPRYHHTSVLQRVALS

QKDRETRLKLTPCCTPTKMSALSMLYFDNDGYIFNKNLPNMKVDACGCS

>Ciona intestinalis (inhibin beta)

MISMFNIAAFVFTMTSIFVNGESVTSSNPTNAEALLRHMQQRSNAVRSKYMMELYRAKNT

GMPTNNALKRSDIVRSFRSIESYKVGSKLVVVFDLSSVPRERIRGSELQVDRSLSDVTMI

ELWEGINAESRRKIGTMRYIRTNKSFDTTSQIRRWHRGDSPVTPFHASGAILPLTPLLVV

YSNNHHYDLIEASESRMNRQRRSSRGRHSNKYRKRRYRVNKNKTRKSTLRHNDVEITSHA

QQKSRRISNDFELSTSSADDVTIQSGNEYDVTDEWRKQQSDDVKVVKAKHPVSPQRKHPF

IISTPAPTVGATHCRKVSFEIDFEKIGWGSWIVYPKKYNAYRCEGQCRTPMRSDSKPTNH

AYMQSIIATKRPDLHIPQPCCVPTKLKPLSILYYEDGEVKQRDHEDMVVDECGCR

**Integrin beta chain family**

>Homo sapiens ITGB3

MRARPRPRPLWATVLALGALAGVGVGGPNICTTRGVSSCQQCLAVSPMCAWCSDEALPLG

SPRCDLKENLLKDNCAPESIEFPVSEARVLEDRPLSDKGSGDSSQVTQVSPQRIALRLRP

DDSKNFSIQVRQVEDYPVDIYYLMDLSYSMKDDLWSIQNLGTKLATQMRKLTSNLRIGFG

AFVDKPVSPYMYISPPEALENPCYDMKTTCLPMFGYKHVLTLTDQVTRFNEEVKKQSVSR

NRDAPEGGFDAIMQATVCDEKIGWRNDASHLLVFTTDAKTHIALDGRLAGIVQPNDGQCH

VGSDNHYSASTTMDYPSLGLMTEKLSQKNINLIFAVTENVVNLYQNYSELIPGTTVGVLS

MDSSNVLQLIVDAYGKIRSKVELEVRDLPEELSLSFNATCLNNEVIPGLKSCMGLKIGDT

VSFSIEAKVRGCPQEKEKSFTIKPVGFKDSLIVQVTFDCDCACQAQAEPNSHRCNNGNGT

FECGVCRCGPGWLGSQCECSEEDYRPSQQDECSPREGQPVCSQRGECLCGQCVCHSSDFG

KITGKYCECDDFSCVRYKGEMCSGHGQCSCGDCLCDSDWTGYYCNCTTRTDTCMSSNGLL

CSGRGKCECGSCVCIQPGSYGDTCEKCPTCPDACTFKKECVECKKFDRGALHDENTCNRY

CRDEIESVKELKDTGKDAVNCTYKNEDDCVVRFQYYEDSSGKSILYVVEEPECPKGPDIL

VVLLSVMGAILLIGLAALLIWKLLITIHDRKEFAKFEEERARAKWDTANNPLYKEATSTF

TNITYRGT

>Mus musculus ITGB3

MRAQWPGQLWAALLALGALAGVVVGESNICTTRGVNSCQQCLAVSPVCAWCSDETLSQGS

PRCNLKENLLKDNCAPESIEFPVSEAQILEARPLSSKGSGSSAQITQVSPQRIALRLRPD

DSKIFSLQVRQVEDYPVDIYYLMDLSFSMKDDLSSIQTLGTKLASQMRKLTSNLRIGFGA

FVDKPVSPYMYISPPQAIKNPCYNMKNACLPMFGYKHVLTLTDQVSRFNEEVKKQSVSRN

RDAPEGGFDAIMQATVCDEKIGWRNDASHLLVFTTDAKTHIALDGRLAGIVLPNDGHCHI

GTDNHYSASTTMDYPSLGLMTEKLSQKNINLIFAVTENVVSLYQNYSELIPGTTVGVLSD

DSSNVLQLIVDAYGKIRSKVELEVRDLPEELSLSFNATCLNNEVIPGLKSCVGLKIGDTV

SFSIEAKVRGCPQEKEQSFTIKPVGFKDSLTVQVTFDCDCACQAFAQPSSPRCNNGNGTF

ECGVCRCDQGWLGSMCECSEEDYRPSQQEECSPKEGQPICSQRGECLCGQCVCHSSDFGK

ITGKYCECDDFSCVRYKGEMCSGHGQCNCGDCVCDSDWTGYYCNCTTRTDTCMSTNGLLC

SGRGNCECGSCVCVQPGSYGDTCEKCPTCPDACSFKKECVECKKFNRGTLHEENTCSRYC

RDDIEQVKELTDTGKNAVNCTYKNEDDCVVRFQYYEDTSGRAVLYVVEEPECPKGPDILV

VLLSVMGAILLIGLATLLIWKLLITIHDRKEFAKFEEERARAKWDTANNPLYKEATSTFT

NITYRGT

>Tetraodon nigroviridis ITGB3

VPGSNVCTTRGASTCKQCLAVHPSCAWCFQEVRFFLLCFSSPRCDLKRNLVAAGCAASAV

ESPTSKLQVVEDRPLSNKATLATQDITQIKPQRLHINLRPDDAKRFTVKVRQVEDYPVDL

YYLMDLSYSMNDDLFRLRTLGKGLAEAMSRTTSNLRMGFGAFVDKPLSPYMYISPKEAVK

NPCYSINMTCLPQFGYKHVLTLTEEVGRFTEEVKKQMVSRNRDAPEGGFDAIIQAAVCKE

QIGWRPGASHLLIFTSDAKTHVALDGRLAGIVQPNDGKCHLNSENMYSMSTTMDYPSLAL

ITEKMSENNINLIFAVTNPVVRLYQPLQELKGRFCVQNYSELIPGTTVGTLNNDSGNVIQ

LILKAYAKIRSKVELELQGVPEELALSFNASCLNGELIPGLKSCSGLKIGDTFLIMVASL

ALFPTISSCQVSFTVEARARGCPKEKKKTFVIKPVGFKDTLSITVTFECDCKCQARAQPS

SPKCNHGNGTYECGICMCHPGRLGPHCECAEGDYDPTEQDSCSGPTGSGGRASVCSGRGD

CVCGQCVCHSSDFGKVWGKLCECDDFNCLHYKGELCSGHGVCNCGFCQCAPDWQGDNCNC

SRRTDTCMSGMGLLCSGRGQCVCGACECTQPGAYGATCDKCPTCPDACTMKKECVECKHF

KRGKLFEGSTCSRICKDEIVLVDELAGSEVTNAVNCSYKDEDDCVQRFQYYEEASGKSIL

YVVKEPDCPKGPDILVVLLSVAGAILFLGLAALLIWKLLITIHDRREFAKFEEERARAKW

DTGHNPLYKGATSTFTNITFRG

>Takifugu rubripes ITGB3

VPGSNVCTTRGASTCKECLAVHPSCAWCLQEVGLFLLCFRMDKMLNGLSPRCDLKRNLVA

AGCTPSAVESPTSKLQVMEDRPLSNKATVATQDITQIKPQRLHINLRPDDAKRFTVKVRQ

VEDYPVDLYYLMDLSYSMNDDLFRLRSLGKGLAEAMNRTTSNLRMGFGAFVDKPLSPYMY

ISPKEAVKNPCYSINMTCLPQFGYKHVLTLTEEVSRFTEEVKKQMVSRNRDAPEGGFDAI

IQAAVCKKKIGWRPGASHLLIFTSDAKTHVALDGRLAGIVQPNDGKCHLNAENMYSMSTT

MDYPSLALITEKMSENNINLIFAVTNPVVPLYQVGTFTGQNYSELIPGTTVGTLNNDSGN

VIQLILQAYAKIRSKVELELQGVPDGLSLSFNATCLNGELIPGLRSCSGLKIGDTFLIIV

ASLALFPTISSHQVSFTVEARARGCPKEKKKTFFIKPVGFKDTLSITVTFECDCKCQAKA

QPSSPKCNHGNGTYECGICMCHPGRLGPHCECAEGDYDPTEQDSCSGPTGSGGRGSICSG

RGDCVCGQCVCHSSDFGKVWGKLCECDDFNCLHYKGELCSGHGVCNCGFCQCAPDWQGEN

CNCSRRTDTCMSSMGLLCSGRGQCVCGACECTQPGAYGATCDKCPTCPDACTMKKECVEC

KHFKRGKLFQDNTCSRICKDEIVLVDELGSGVTNAVNCSYKDEDDCVERFQYYEEASGKS

ILYVVKEPDCPKGADILVVLLSVAGAILFLGLAALLIWKLLITIHDRREFAKFEEERARA

KWDTGHNPLYKEVTSTFTNITFRG

>Homo sapiens ITGB6

MGIELLCLFFLFLGRNDHVQGGCALGGAETCEDCLLIGPQCAWCAQENFTHPSGVGERCD

TPANLLAKGCQLNFIENPVSQVEILKNKPLSVGRQKNSSDIVQIAPQSLILKLRPGGAQT

LQVHVRQTEDYPVDLYYLMDLSASMDDDLNTIKELGSRLSKEMSKLTSNFRLGFGSFVEK

PVSPFVKTTPEEIANPCSSIPYFCLPTFGFKHILPLTNDAERFNEIVKNQKISANIDTPE

GGFDAIMQAAVCKEKIGWRNDSLHLLVFVSDADSHFGMDSKLAGIVIPNDGLCHLDSKNE

YSMSTVLEYPTIGQLIDKLVQNNVLLIFAVTQEQVHLYENYAKLIPGATVGLLQKDSGNI

LQLIISAYEELRSEVELEVLGDTEGLNLSFTAICNNGTLFQHQKKCSHMKVGDTASFSVT

VNIPHCERRSRHIIIKPVGLGDALELLVSPECNCDCQKEVEVNSSKCHHGNGSFQCGVCA

CHPGHMGPRCECGEDMLSTDSCKEAPDHPSCSGRGDCYCGQCICHLSPYGNIYGPYCQCD

NFSCVRHKGLLCGGNGDCDCGECVCRSGWTGEYCNCTTSTDSCVSEDGVLCSGRGDCVCG

KCVCTNPGASGPTCERCPTCGDPCNSKRSCIECHLSAAGQAREECVDKCKLAGATISEEE

DFSKDGSVSCSLQGENECLITFLITTDNEGKTIIHSINEKDCPKPPNIPMIMLGVSLAIL

LIGVVLLCIWKLLVSFHDRKEVAKFEAERSKAKWQTGTNPLYRGSTSTFKNVTYKHREKQ

KVDLSTDC

>Mus musculus ITGB6

MGIELVCLFLLLLGRNDHVQGGCAWGGAESCSDCLLTGPHCAWCSQENFTHLSGAGERCD

TPANLLAKGCQLPFIENPVSRIEVLQNKPLSVGRQKNSSDIVQIAPQSLVLKLRPGREQT

LQVQVRQTEDYPVDLYYLMDLSASMDDDLNTIKELGSRLAKEMSKLTSNFRLGFGSFVEK

PVSPFMKTTPEEITNPCSSIPYFCLPTFGFKHILPLTDDAERFNEIVRKQKISANIDTPE

GGFDAIMQAAVCKEKIGWRNDSLHLLVFVSDADSHFGMDSKLAGIVIPNDGLCHLDHRNE

YSMSTVLEYPTIGQLIDKLVQNNVLLIFAVTQEQVHLYENYAKLIPGATVGLLQKDSGNI

LQLIISAYEELRSEVELEVLGDTEGLNLSFTALCNNGVLFPHQKKCSHMKVGDTASFNVT

VSVSNCEKRSRNLIIKPVGLGDTLEILVSAECDCDCQREIETNSSKCHNGNGSFQCGVCT

CNPGHMGPHCECGEDMVSTDSCKESPGHPSCSGRGDCYCGQCICHLSPYGSIYGPYCQCD

NFSCLRHKGLLCGDNGDCDCGECVCRDGWTGEYCNCTTNRDSCTSEDGVLCSGRGDCVCG

KCVCRNPGASGPTCERCPTCGDPCNSKRSCIECYLSADGQAQEECADKCKAIGATISEED

FSKDTSVSCSLQGENECLITFLITTDNEGKTIIHNINEKDCPKPPNIPMIMLGVSLAILL

IGVVLLCIWKLLVSFHDRKEVAKFEAERSKAKWQTGTNPLYRGSTSTFKNVTYKHREKHK

AGLSSDG

>Rattus norvegicus ITGB6

MGIELVCLFLLLLGRNDHVQGGCAWSGAETCSDCLLTGPHCAWCSQENFTHLSGAGERCD

TPENLLAKGCQLPFIENPVSQVEILQNKPLSVGRQKNSSDIVQIAPQSLVLKLRPGGEQT

LQVQVRQTEDYPVDLYYLMDLSASMDDDLNTIKELGSRLAKEMSKLTSNFRLGFGSFVEK

PVSPFMKTTPEEITNPCSSIPYFCLPTFGFKHILPLTDDAERFNEIVRKQKISANIDTPE

GGFDAIMQAAVCKEKIGWRNDSLHLLVFVSDADSHFGMDSKLAGIVIPNDGLCHLDNRNE

YSMSTVLEYPTIGQLIDKLVQNNVLLIFAVTQEQVHLYENYAKLIPGATVGLLQKDSGNI

LQLIISAYEELRSEVELEVLGDTEGLNLSFTALCSNGILFPHQKKCSHMKVGDTASFNVS

VSITNCEKRSRKLIIKPVGLGDTLEILVSAECDCDCQREVEANSSKCHHGNGSFQCGVCA

CNPGHMGPRCECGEDMVSTDSCKESPGHPSCSGRGDCYCGQCVCHLSPYGSIYGPYCQCD

NFSCLRHKGLLCGDNGDCDCGECVCRDGWTGEYCNCTTSRDACASEDGVLCSGRGDCVCG

KCVCRNPGASGPTCERCPTCGDPCNSRRYCIECYLSADGQAQEECEDKCKATGATISEEE

FSKDTSVPCSLQGENECLITFLITADNEGKTIIHNISEKDCPKPPNIPMIMLGVSLAILL

IGVVLLCIWKLLVSFHDRKEVAKFEAERSKAKWQTGTNPLYRGSTSTFKNVTYKHREKHK

VGLSSDG

>Tetraodon nigroviridis ITGB6

MGLLLLSLVLRYWINSVEGSCSVGSAATCDECLQLSSHCAWCTQENFTDWFSVGQRCDTL

EVLLEKGCARSYLEFPVSEARVLENSPLGKRTESDNSTQISPQKIALKLRPGSQVTFQVK

VQHTEHYPVDLYYLMDLSASMVDDLQRIKDLGSTLSKEMANLTNKFRMGFGSFVEKPTLP

FIKITEEELANPCSSVSPHITCLPAFGYKHVLSLTSDANQFNQIIAKQRVSANIDLPECG

FDAIMQAAVCGDKIGWRNDSMRLLVFVSDADSHFGMDSKMAGIVVPNDGRCHLDANNEYS

MSTMLEYPTLGQLIDKVVENNILLIFAVTEQQIHNYKNYANFIPGATVGVLANDSINILE

LIVTAYKELRSEIELEVLGDTEELQMSFTATCPNGIVLPDLKRCSNVKPGEMVVFNVSVA

LPGCLSGLRHFSLKPVGLQDTLEVELESLCSCDCPSPPEANSSQCVEGKGAFHCGVCVCQ

PGFEDSAFSTDCLASNESEVCSGQGRCFCGQCVCHASNFGHIYGRHCECDDYSCGRFRGE

LCGGHGVCDCGECRCEDGWDGSYCNCSTGTEACTAEDGALCSGRGRCECGRCVCSVPGAS

GDRCEKCPTCGDACTSARICVECHLEEEDAELCQEKCSIPKISINTTADYDKSTSLQCTV

MTENECQISFNVVASETQTTAYNLRIYGCPKPPKIFMIVLGLSLSVVCIGLVLLVVWKVL

VSFHDRKEVARFEAERSKAKWQTGTNPLFRSSTSTFKNVTYKETHK

>Takifugu rubripes ITGB6

GSCSAGSAATCDECLQLSSHCAWCTQENFTDWFSVGERCDAPDALLVKGCARSYLEFPVS

KAQVLQDLPLGKKSETHNSTQISPQKIALKMRPGSQVTFQVKVQHTEDYPVDLYYLMDLS

ASMMDDLQKIKDLGSSLSKEMANLTSKFHMGFGSFVEKPTLPFIKITEEELADPCSGISS

SLTCLPTFGYKHMLSLTSDTDKFNQIIAKQRVSANIDLPECGFDAIMQAAVCGDKIGWRN

DSMRLLVFVSDADSHFGMDSKMAGIVIPNDGHCHLDANNEYSMSTMLEYPTLGQLIDKVV

ENNILLIFAVTEPQIHNYKNYANFIPGATVGVLATDSRNILELIVTAYKLYHPAHLEHDS

SKNYRCRVLPPHRSLRGESVIPRKSPFISVFQGDVWFSSVADNCISTLRNLCAVLQELRS

EIELEVLGDTEELQMSFTTICPNGTVLPDLKRCTNIKPGETVVFNVSVALPECLSGLRHL

FLKPVGLQDSLEVELKSLCSCDCQSPPNANSSQCAEGQGTFHCGVCVCQPGFMGAECECS

EESTLSSNCLANNGSEVCSGQGQCYCGQCVCHTSNFGRIYGHHCECDNYSCARFRGELCG

GHGVCDCGECHCESGWEGHYCNCSTGTEACVSEDGTLCSGRGRCECGHCVCSIPGASGDR

CEKCPTCGDACNSARTCVECHLEEEDAELCDQQCSIPKISINSTADYDKSASLQCTVMTE

NECWISFNVVASETRTIAYNLQIYGCPEAPGMLVIILGLSLSVVCIGLILLVVWKVLVSV

HDRKEVAKFEAERSKAKWQTGTNPLFRSSTSTFKNVTYRETHRDK

>Homo sapiens ITGB8

MCGSALAFFTAAFVCLQNDRRGPASFLWAAWVFSLVLGLGQGEDNRCASSNAASCARCLA

LGPECGWCVQEDFISGGSRSERCDIVSNLISKGCSVDSIEYPSVHVIIPTENEINTQVTP

GEVSIQLRPGAEANFMLKVHPLKKYPVDLYYLVDVSASMHNNIEKLNSVGNDLSRKMAFF

SRDFRLGFGSYVDKTVSPYISIHPERIHNQCSDYNLDCMPPHGYIHVLSLTENITEFEKA

VHRQKISGNIDTPEGGFDAMLQAAVCESHIGWRKEAKRLLLVMTDQTSHLALDSKLAGIV

VPNDGNCHLKNNVYVKSTTMEHPSLGQLSEKLIDNNINVIFAVQGKQFHWYKDLLPLLPG

TIAGEIESKAANLNNLVVEAYQKLISEVKVQVENQVQGIYFNITAICPDGSRKPGMEGCR

NVTSNDEVLFNVTVTMKKCDVTGGKNYAIIKPIGFNETAKIHIHRNCSCQCEDNRGPKGK

CVDETFLDSKCFQCDENKCHFDEDQFSSESCKSHKDQPVCSGRGVCVCGKCSCHKIKLGK

VYGKYCEKDDFSCPYHHGNLCAGHGECEAGRCQCFSGWEGDRCQCPSAAAQHCVNSKGQV

CSGRGTCVCGRCECTDPRSIGRFCEHCPTCYTACKENWNCMQCLHPHNLSQAILDQCKTS

CALMEQQHYVDQTSECFSSPSYLRIFFIIFIVTFLIGLLKVLIIRQVILQWNSNKIKSSS

DYRVSASKKDKLILQSVCTRAVTYRREKPEEIKMDISKLNAHETFRCNF

>Mus musculus ITGB8

MCGSALAFLTAALLSLHNCQRGPALVLGAAWVFSLVLGLGQSEHNRCGSANVVSCARCLQ

LGPECGWCVQEDFVSGGSGSERCDTVSSLISKGCPVDSIEYLSVHVVTSSENEINTQVTP

GEVSVQLHPGAEANFMLKVHPLKKYPVDLYYLVDVSASMHNNIEKLNSVGNDLSKKMALY

SRDFRLGFGSYVDKTVSPYISIHPERIHNQCSDYNLDCMPPHGYIHVLSLTENITEFEKA

VHRQKISGNIDTPEGGFDAMLQAAVCESHIGWRKEAKRLLLVMTDQTSHLALDSKLAGIV

VPNDGNCHLKNNVYVKSTTMEHPSLGQLSEKLIDNNINVIFAVQGKQFHWYKDLLPLLPG

AIAGEIESKAANLNNLVVEAYKKIISEVKVQLENQVHGVHFNITAICPDGARKPGISGCG

NVTSNDEVLFNVTVVMKTCDIMGGKNYAIIKPIGFNETTKVHIHRSCSCQCENHRGLKGQ

CAEAAPDPKCPQCDDSRCHFDEDQFPSETCKPQEDQPVCSGRGVCICGKCLCHKTKLGRV

YGQYCEKDDFSCPYLHGDVCAGHGECEGGRCQCFSGWEGDRCQCPSASAQHCVNSKGQVC

SGRGTCVCGRCECTDPRSIGRLCEHCPTCHLSCSENWNCLQCLHPHNLSQAVLDQCKSSC

AVMEQHRMDQTSECLSGPSYLRIFFIIFIVTFLIGLLKVLIIRQVILQWNNNKIKSSSDY

RMSASKKDKLILQSVCTRAVTYRREKPEEIKMDISKLNAQEAFRCNF

>Rattus norvegicus ITGB8

MCGSALAFLTAALLCLHNCQRGPALVLGAAWVFSLVLGLGQSEHNRCDSANVVSCARCLQ

LGPECGWCVQEDFVSGGSRSERCDTVSNLISKGCPVDSIEYLSVHVVTSSENEINTQVTP

GDVSIQLHPGAEANFMLKIRPLKKYPVDLYYLVDVSASMHNNIEKLNSVGNDLSKKMALF

SHDFRLGFGSYVDKTVSPYISIHPERIHNQCSDYNLDCMPPHGYIHVLSLTENITEFEKA

VHRQKISGNIDTPEGGFDAMLQAAVCESHIGWRKEAKRLLLVMTDQTSHLALDSKLAGIV

VPNDGNCHLKNNVYVKSTTMEHPSLGQLSEKLIDNNINVIFAVQGKQFHWYKDLLPLLPG

AIAGEIESKAANLNNLVVEAYKKLISEVKVQLESQVHGVHFNITAICPDGARKPGTSGCG

NVTSNDEVLFNVTVVMKTCDIIGGKNYAIIKPIGFNETTKVHIHRSCSCQCEDHRGLKGQ

CAEAAPDPKCPQCDDGKCHFDEDQFPSEACKPQEDQPVCSGRGVCICGKCLCHKTKLGRV

YGQYCEKDDFSCPYLHGDVCSGHGECEGGRCQCFNGWEGDRCQCPSASAQHCVNSKGQVC

SGRGTCVCGRCECTDPRSIGRLCEHCPTCHLSCSENWNCLQCLHPHNLSQAVLDQCKASC

AVMEQHPVAQTSECLSGPSYLRIFFIIFIVTFLIGLLKVLIIRQVILQWNSNKIKSSSDY

RVSASKKDKLILQSVCTRAVTYRREKPEEIKMDISKLNAQEAFRCNF

>Takifugu rubripes ITGB8

MCLSASITSCAECLSHGPQCAWCFKEDFLNGADLGWRCDLPENLLNRGCEAESMERWETK

VVVNTTISSTQVSPGDISITLTPGRQTQVQCEMVKQLQRYPVDLYYLVDVSASMQENLNH

LKTVGVALTLRMTEHSSDLWLGFGSFVDKPVSPYIDVHPSKINNPCSDYEIRCRPAHGFH

HVLSMTGNMSEFTRVIKRQHISGNMDTPEGGLDAMLQAAVCQVCSARANLLAPGSHALHA

LEAVGWRSEAKRLLLLMTDQPSHLALDSRLAGIVVPHDGLCHLENNVYTGSTRMDHPSVG

QLSDKLLENHIYSVFAVEKQQYQWYEVMSPTHVHTSVYRSAHICGWENKYFRPSCSSEDR

DQTPALHSLYYSLGTLVALEFDRNLLTCFPSQTYFKITFGLRSCPDDGGDEDVTVLIHPV

GYNESTVVRIHSKCVCSCGVTKRCSNDGQSPCSGMQDEDRGPNHELMDSNKGSNRNCRPD

VADVDCSGRGVCECGRCVCDRSKLGAVYGKYCEIDDFSCPYHEGLSCGGRGACVSSECVC

TDGWTGESCSCPISTATCQSASGSLCSGRGRCVCGKCACDDPQYSGDFCETCPACQSPCQ

S

>Tetraodon nigroviridis ITGB8

DFLNGAASGRRCDLPENLLARGCEVEVMERWETRVEVNTTVSGTQVSPRDISVTLRPGSE

ASIVVEVKQLHRYPVDLYYLVDVSASMQENLDHLKTVGVALTLRMTEHTSDLWLGFGSFV

DKPVSPYINVHPSKISNPCSDYEIRCRPAHGFHHVLSMTGNMSEFTRVIKRQRISGNMDT

PEGGLDAMLQAAVCQV

>Danio rerio ITGB8

NRCLSALISTCEDCLRRGPECAWCSQERFLDGAHVSQRCGSVSDLRVRGCEEEFIENPKV

KVEVNATISSSQVAPREITIHLRPGSEARFVIEVQQLERYPVDLYYLVDVSASMQDNLDR

LKTVGLALSHQMKQHSSDFRVGFGSFVDKPVSPYIDVHPSKLLNPCRHYVVTALMIILFD

YSSDVKLSLKITSHQSYESAQMISFSSVESVSAHPTDLCSCSCCVQKDIGWRPEAKHLLL

VMTDQPSHLALDSKLAGIVVPHDGRCHLEDNVYSQTSTMEHPTVAQLAEKLLENSIYSIF

AVDHLQYQWYEDLVDLIPGSYVGRLFPKASNLKDLVVQAYKKLLSDVEVQMELQDPQQAE

RFWVNVSAICPGGSSAAGNSKCSGVQPKQTVLFQLTVGMLSCPAGSADQDVLMLVRPVGF

NESVRVRIRQLCGSGCTDPKPEPSSCTTNASDSCREHRNTPVCSSRGVCRCGTCVCDSSR

FGNIYGKFCEMDDFSCPYDVTFLVSGHGRCVRGECVCFSGWTGEACACRESTDSCVSDDG

FICGDRGRCVCGKCVCDDPRRSGAFCEKCPMCHSSCQSHWNCVNCHVWNGLGSDGFQLCN

QSC

>Homo sapiens ITGB7

MVALPMVLVLLLVLSRGESELDAKIPSTGDATEWRNPHLSMLGSCQPAPSCQKCILSHPS

CAWCKQLNFTASGEAEARRCARREELLARGCPLEELEEPRGQQEVLQDQPLSQGARGEGA

TQLAPQRVRVTLRPGEPQQLQVRFLRAEGYPVDLYYLMDLSYSMKDDLERVRQLGHALLV

RLQEVTHSVRIGFGSFVDKTVLPFVSTVPSKLRHPCPTRLERCQSPFSFHHVLSLTGDAQ

AFEREVGRQSVSGNLDSPEGGFDAILQAALCQEQIGWRNVSRLLVFTSDDTFHTAGDGKL

GGIFMPSDGHCHLDSNGLYSRSTEFDYPSVGQVAQALSAANIQPIFAVTSAALPVYQELS

KLIPKSAVGELSEDSSNVVQLIMDAYNSLSSTVTLEHSSLPPGVHISYESQCEGPEKREG

KAEDRGQCNHVRINQTVTFWVSLQATHCLPEPHLLRLRALGFSEELIVELHTLCDCNCSD

TQPQAPHCSDGQGHLQCGVCSCAPGRLGRLCECSVAELSSPDLESGCRAPNGTGPLCSGK

GHCQCGRCSCSGQSSGHLCECDDASCERHEGILCGGFGRCQCGVCHCHANRTGRACECSG

DMDSCISPEGGLCSGHGRCKCNRCQCLDGYYGALCDQCPGCKTPCERHRDCAECGAFRTG

PLATNCSTACAHTNVTLALAPILDDGWCKERTLDNQLFFFLVEDDARGTVVLRVRPQEKG

ADHTQAIVLGCVGGIVAVGLGLVLAYRLSVEIYDRREYSRFEKEQQQLNWKQDSNPLYKS

AITTTINPRFQEADSPTL

>Mus musculus ITGB7

MVDSSTVLIFLLVLGGGQSELDTKITSSGEAAEWEDPDLSLQGSCQPVPSCQKCILSHPS

CAWCKQLNFTASGEAEARRCARREELLARGCPAQELEEPRGRQEVLQDKPLSQGDRGEGA

TQLAPQRIRVTLRPGEPQKFRVRFLRAAGYPVDLYYLMDLSYSMKDDLERVRQLGHALLV

RLQEVTHSVRIGFGSFVDKTVLPFVSTVPSKLHHPCPSRLERCQPPFSFHHVLSLTGDAQ

AFEREVGRQNVSGNLDSPEGGFDAILQAALCQEQIGWRNVSRLLVFTSDDTFHTAGDGKL

GGIFMPSDGRCHLDSNGVYTNSAEFDYPSVGQVAQALTAANIQPIFAVTGATLPVYQELR

QLIPKSAVGELSEDSSNVVQLIMDAYDSLSSTVTLEHSPLPPGVSISFESHCKGPEKTEG

EAGDRGQCNDVRVNQTVDFWVTLQATHCLPEAHVLRLWALGFSEELTVELHTVCDCNCGD

AQPHAPYCSDGQGDLQCGICSCAPGRLGQLCECSEADLSSPDLESGCRAPNGTGPLCSGK

GRCQCGRCSCSGQSSGRLCECDDASCERHEGILCGGFGHCQCGVCHCHANHTGRACECSK

SVDSCVSPEGGLCSGHGYCKCNRCQCLDGYYGALCDQCLGCKSPCEQYRDCAECGAFGTG

PLAANCSVVCADVNVTLTLAPNLDDGWCKERTIDNQLFFFLVEHAASGIVLRVRPQEKGV

DHTRAIILGCTGGIVAVGLGLVLAYRLSVEIYDRREYRRFEKEQQQLNWKQDNNPLYKSA

ITTTVNPRFQGTNGRSPSLSLTREAD

> Rattus norvegicus ITGB7

MVDSSTVLIFLLVLGGGQSELDTKITSSGETTEWEDPDLSPQGSCQPAPSCQKCILSHPS

CAWCKQLNFTASGEAEARRCARREELLARGCPEQELEEPRGHQEVLQDRPLSQGDRGEGA

TQLAPQRIRVTLRPGEPQKFRVRFLRAAGYPVDLYYLMDLSYSMKDDLERVRQLGQALLL

RLREVTHSVRIGFGSFVDKTVLPFVSTVPSKLNHPCPSRLERCQAPFSYHHVLPLTRDAQ

AFEREVGRQNVSGNLDSPEGGFDAILQAALCQEQIGWRNVSRLLVFTSDDTFHTAGDGKL

GGIFMPSDGRCHLDSNGVYTNSAEFDYPSVGQVAQALTAANIQPIFAVTGATLPVYQELS

QLIPKSAVGELSEDSSNVVQLIMDAYDNLSSTVTLEHSSLPPGVSISFESHCRSPEKSEG

EAGDRRGQCNHVRVNQMVDFWVTLQASHCLPEAHVLRLWALGFSEELTVELHTLCDCNCS

DAQPRAPYCSDGQGDLQCGICSCAPGRLGQLCECSEADLSSPDLESGCRAPNGTGPLCSG

KGRCQCGRCSCSGQSSGRLCECDDASCERHEGILCGGFGHCQCGVCHCHANRTGRACECS

ESVDSCVSPEGGLCSGHGDCKCNRCQCLDGYYGALCDQCLGCKSPCEQYRDCAECGAFGT

GPLAANCSAACADVNVTLALAPNLDDGWCKERTLDNQLFFFLVENSARGVILRVRPQEKG

ADHTRAIILGCVGGIVAVGLGLVLAYRLSVEVYDRLEYSRFEKERQQLNWKQDSNPLYKS

AVTTTVNPRFQGGNKQSLSLPLTQEAD

>Danio rerio ITGB7

MDLKLLFISALLGIISCSRAQQEGNECIKANALSCGECIQVGDKCGWCTDAEFLKQGEPT

SARCDELESLKKRGCAEAKIENPHGSQRILKNTPVTNRKKGAEKLRPEDITQIQPQKLSL

QLRSGEPQNIKLKFKRAEDYPIDLYYLMDLSYSMKDDLENVKNLGTSLMKEMSKITSDFR

IGFGSFVEKTVMPYISTTPAKLLNPCTGDQNCTSPFSYKNVLKLTSNGQRFNSLVGQQQI

SGNLDSPEGGFDAIMQVAVCGEHIGWRNVTRLLVFSTDAGFHFAGDGKLGGIVLPNDGRC

HLENDMYTMSHYYDYPSIAHLVQKLSENNIQTIFAVTEEFQPVYKELKNLIPKSAVGTLS

ANSSNVINLIVDAYNSLSSEVILENSKLPEGVTITYQSRCKNGVVNEGESGRKCSNISIG

DEVSFNINITAQGCPKQGKTETIKIKPLGFTEEVEITLSFICECECHKHAMKNSPLCHNG

NGSFECGACRCNKGRVGRQCECRKDEVSTEDLDKNCRKDNGTDICSNNGECVCGTCECKK

RENPEERYSGKYCECDNFNCDRSNNKLCGGHGRCECRVCVCDANYTGSACDCSLDTSTCL

ASNKQICNGRGICECGTCRCTDPKFQGPTCEICPTCPGVCTEHKECVQCRAFGTGEKKDT

CKRDCSYFNLIEVEDRDKLPQPVQAFPLMHCKERDARDCWFYYTYAVNNNTEKEVHVVKT

MECPPGPDIIPIVAGVVAGIVLIGLALLLIWKLLMIIHDRREFAKFEKEKMNAKWDTGEN

PIYKSAVTTVINPKYEGK

>Takifugu rubripes ITGB7

EGSVCIKANAQSCGECIQVSESCGWCTDVSFLSEGESKSARCDDLESLKKRKCTAESIENPRGSVVVDKD

KPVTNRRNEPGKLKPEQITQIQPQKLTLTLRSGEPQTFKLKFKRAEDYPIDLYYLMDLSFSMKDDLENVK

NLGTDLMKEMQKITSDFRIGFGSFVEKTVMPYISTTPARLANPCTGNQNCTSPFSYKNVLKLTNKGDEFN

RLVSQQQISGNLDSPEGGFDAIMQVAVCQNEIGWRNVTRLLVFSTDAGFHFAGDGKLGGIVLPNDGKCHL

EKDMYTMSHYYDYPSIAHLVQKLSDHNIQTIFAVTEEFQPVYKELKNLIPKSAVGTLSSNSSNVIKLIID

AYNSLSSEVILENGKLPEGVSINYKSICKNGVEGTGENGRKCSNISIGDEVFFQISVQADKCPADSRSET

IRIKPLGFTEEVEIVVNFICDCECAAKGEPQSSKCDNGNGIFQCGACKCNEGRIGRVCECSKDDVHTEDL

DANCRKDNGTDICSNNGDCVCGTCECKKRQNPAEIYSGKYCECDNFNCDRSNNMLCGGHGRCECRKCICD

ANYTGSACDCSMDKSTCMAKNGQLCNGRGLCECGICKCTDVKFQGPTCEICPTCPGVCQEHKDCVLCRAF

PSEKPCVNCSYFDLVKVADRDKLPQPTDQSFPLTHCKVRDENDCWVYYTFAIQNQTRLVHVVEKPGCTEG

PDIVPIVAGVVAGIVLIGLALLLIWKLLMIIHDRREFAKFEKEKMNAKWDTGENPIYKSAVTTVVNPKYE

GK

>Homo sapiens ITGB5

MPRAPAPLYACLLGLCALLPRLAGLNICTSGSATSCEECLLIHPKCAWCSKEDFGSPRSI

TSRCDLRANLVKNGCGGEIESPASSFHVLRSLPLSSKGSGSAGWDVIQMTPQEIAVNLRP

GDKTTFQLQVRQVEDYPVDLYYLMDLSLSMKDDLDNIRSLGTKLAEEMRKLTSNFRLGFG

SFVDKDISPFSYTAPRYQTNPCIGYKLFPNCVPSFGFRHLLPLTDRVDSFNEEVRKQRVS

RNRDAPEGGFDAVLQAAVCKEKIGWRKDALHLLVFTTDDVPHIALDGKLGGLVQPHDGQC

HLNEANEYTASNQMDYPSLALLGEKLAENNINLIFAVTKNHYMLYKNFTALIPGTTVEIL

DGDSKNIIQLIINAYNSIRSKVELSVWDQPEDLNLFFTATCQDGVSYPGQRKCEGLKIGD

TASFEVSLEARSCPSRHTEHVFALRPVGFRDSLEVGVTYNCTCGCSVGLEPNSARCNGSG

TYVCGLCECSPGYLGTRCECQDGENQSVYQNLCREAEGKPLCSGRGDCSCNQCSCFESEF

GKIYGPFCECDNFSCARNKGVLCSGHGECHCGECKCHAGYIGDNCNCSTDISTCRGRDGQ

ICSERGHCLCGQCQCTEPGAFGEMCEKCPTCPDACSTKRDCVECLLLHSGKPDNQTCHSL

CRDEVITWVDTIVKDDQEAVLCFYKTAKDCVMMFTYVELPSGKSNLTVLREPECGNTPNA

MTILLAVVGSILLVGLALLAIWKLLVTIHDRREFAKFQSERSRARYEMASNPLYRKPIST

HTVDFTFNKFNKSYNGTVD

>Mus musculus ITGB5

MPRVPATLYACLLGLCALVPRLAGLNICTSGSATSCEECLLIHPKCAWCSKEYFGNPRSI

TSRCDLKANLIRNGCEGEIESPASSTHVLRNLPLSSKGSSATGSDVIQMTPQEIAVSLRP

GEQTTFQLQVRQVEDYPVDLYYLMDLSLSMKDDLENIRSLGTKLAEEMRKLTSNFRLGFG

SFVDKDISPFSYTAPRYQTNPCIGYKLFPNCVPSFGFRHLLPLTDRVDSFNEEVRKQRVS

RNRDAPEGGFDAVLQAAVCKEKIGWRKDALHLLVFTTDDVPHIALDGKLGGLVQPHDGQC

HLNEANEYTASNQMDYPSLALLGEKLAENNINLIFAVTKNHYMLYKNFTALIPGTTVEIL

HGDSKNIIQLIINAYSSIRAKVELSVWDQPEDLNLFFTATCQDGISYPGQRKCEGLKIGD

TASFEVSVEARSCPGRQAAQSFTLRPVGFRDSLQVEVAYNCTCGCSTGLEPNSARCSGNG

TYTCGLCECDPGYLGTRCECQEGENQSGYQNLCREAEGKPLCSGRGECSCNQCSCFESEF

GRIYGPFCECDSFSCARNKGVLCSGHGECHCGECKCHAGYIGDNCNCSTDVSTCRAKDGQ

ICSDRGRCVCGQCQCTEPGAFGETCEKCPTCPDACSSKRDCVECLLLHQGKPDNQTCHHQ

CKDEVITWVDTIVKDDQEAVLCFYKTAKDCVMMFSYTELPNGRSNLTVLREPECGSAPNA

MTILLAVVGSILLIGMALLAIWKLLVTIHDRREFAKFQSERSRARYEMASNPLYRKPIST

HTVDFAFNKFNKSYNGSVD

>Tetraodon nigroviridis ITGB5

GLNICTGGSATSCQECLLIHPSCAWCAQDVRLPNLALVIKSQPIVFGVSEVPANANIRIY

LSFQDFGQRKTLTSRCDFEQNLVKRGCDQRFVESPSSSISVLQNVPLSSKGVSQYDVVQI

MPQKMALSLRPGRCPVREDWCLFPEKPEIKAAKMVSKRLLRVFAPSGGQTWFQLQVRQVE

DYPVDLYYLMDLSLSMKDDLDTIRNLGTKLAYEMGKLTSNFRLGFGSFVDKNMSPFSYTA

EKYRENPCNGYKPFPNCVPTFGFRHILPLTDKVDSFNVEVQKQMVSRNRDAPEGGFDAIL

QAAVCKEKIGWRKEAYHLLVFATDDVPHLALDGRLGGLVEPHDGQCHLDDGSEYSASTKM

DYPSLALLGEKLAENNIFLIFAVTKQLYIIYKNFTALIPGTTVEILDQDSKNVIQLIIAA

YNNIRSKVELSVWDHPEDLSLSFTATCQDGMPLPGLKKCADLKIGDTVSFNVSVEARSCP

PRGANHSFTIKPVGFKDRLEVSVDYRCDCGCSEVAQVNSSVCNSTGTYNCGICHCEPGYL

GAHCECQESEASTIYLNACRGAEGKQVCSGRGKCSCNQCLCYESEFGKIYGSFCECDDFS

CARHKGILCSGHGECHCGECKCHAGYVGDNCNCSTETSSCISDDGQMCSGRGSCVCGRCQ

CTEPGSFGDTCEKCPTCPDACDTKRKCIECRLFNSGGFTDNQTCQRLCKDEIITVETLNT

DDRNAVLCLYKTDDNCVMKFTYSEHTSGQSILTALKEPECGTGTNVLMVLLAVVGSILLV

GVVLLAAWKLVITVHDLREFSRFQSARSRARYEMVRMDNPGVI

>Takifugu rubripes ITGB5

GLNICTSGSATSCRDCLLIHPSCAWCAQEVRLYCCRLCHVSVPTNHVRRFRSHRGRSFFF

SVLQDFGRGKTLTSRCDFIQNLQKRGCEPRFVENPSSSISVLQNIPLSSKGLSQYDVIQI

MPQKLALSLRPGRFPSHSVRDNRCLFSRPTEILPKHFLNVFARSGGQTWFELQVRQVEDY

PVDVYYLMDLSLSMKDDLETIRNLGTKLAHEMGKLTSNFRLGFGTFVDKNMSPFSYTAEK

YQENPCSGYKLFPNCVPTFGFRHILPLTDKVDRFNEEVQKQMVSRNRDAPEGGFDAILQA

AVCKEKIGWRKEAYHLLVFTTDDVPHLALDGRLGGLVEPHDGQCHLDDNSEYSASTKMDY

PSLALLGEKLAENNIFLIFAVTKQLYIIYKNFTALIPGTTVEILDQDSKNVIQLIVAAYN

NIRSKVELSVWDHPEDISLSFTATCQDGMPLPGFRKCADLKIGDTVSFNVSVEARSCPPH

GANHSFTIKPVGFKDHLEVSVDYQCDCGCSKTAQVNSSICSSTGTYNCGTCHCEPGYLGA

RCECQEGEASSIYLNACREAEGKQVCSGRGECSCNQCLCYESEFGKIYGSFCECDDFSCA

RHKGILCSGHGECHCGECKCHAGYVGDNCNCSTETSSCISDDGRMCSGRGSCACGRCQCT

EPGSFGETCEKCPTCPDACATKRECIECRLFNSGQLTDNQTCQRLCKDEIITVETLKTDD

PGAVLCLYKTDNNCVMKFTYSEHTSGQSVLTTLKEPECSTGTDALMVLLAVVGSILLVGV

VLLAVWKLVITIHDWREFSRFQSARSRARYEMVRSDNPSLL

>Danio rerio ITGB5

KSCKINQLLKVNEFRFQQRGLNVCMSGSATSCEECLLIHPSCAWCAQEDFGQARTLTSRC

DFSQNLQKRGCDAPFIENPRSGSSVLQSKPLSYKGSGVTQYDVIQIYPQKISLSLRPGDQ

ASFEVQVRPVEDYPVDLYYLMDLSLSMKDDLDTIRNLGTKLAEEMRKLTSNFRLGFGSFV

DKNISPFSYTASKYQDNPCNGYKLFPNCVPTFGFRHLLSLTDKVDRFNEEVQKQMVSRNR

DAPEGGFDAILQAAVCREKIGWRKEAYHLLVFATDDVPHLALDGKLGGLVHPHDGQCHLN

DKNEYSASTKMDYPSLALLGEKLAENNIFLIFAVIKRHYVLYKNLTALIPGTTVEILDQD

SKNIIQLIVNAYNNIRSKVELSVWDHPEDLSLAFSATCQDGQPLTGLRKCADLKIGDTVS

FNVTVDARGCPPKGTRKSFTIRPVGFKDRLEVSVDYRCDCRCTYHTKPNSSRCNSAGTYN

CGLCRCEPGYLGARCECQEGEVDHQHLSACREAEGKQVCSGRGECSCNQCLCYESEFGKI

YGTFCECDDFSCARHKGILCSGHGECHCGECKCHAGYIGDNCNCSTETASCVSDDGKICS

GRGSCVCGRCQCTEPGAFGDTCEKCPTCPDACGTKRECIECRLFNTGRLADNQTCQRMCK

DEITTVETLILVNCIFENIPYLRSIMSNNGVMHLCNVCVFTECAVAPNAMTVLLAVVGSI

LLMGIVLLALWKLVITVHDRREFARFQSARSRARY

>Homo sapiens ITGB4

MAGPRPSPWARLLLAALISVSLSGTLANRCKKAPVKSCTECVRVDKDCAYCTDEMFRDRR

CNTQAELLAAGCQRESIVVMESSFQITEETQIDTTLRRSQMSPQGLRVRLRPGEERHFEL

EVFEPLESPVDLYILMDFSNSMSDDLDNLKKMGQNLARVLSQLTSDYTIGFGKFVDKVSV

PQTDMRPEKLKEPWPNSDPPFSFKNVISLTEDVDEFRNKLQGERISGNLDAPEGGFDAIL

QTAVCTRDIGWRPDSTHLLVFSTESAFHYEADGANVLAGIMSRNDERCHLDTTGTYTQYR

TQDYPSVPTLVRLLAKHNIIPIFAVTNYSYSYYEKLHTYFPVSSLGVLQEDSSNIVELLE

EAFNRIRSNLDIRALDSPRGLRTEVTSKMFQKTRTGSFHIRRGEVGIYQVQLRALEHVDG

THVCQLPEDQKGNIHLKPSFSDGLKMDAGIICDVCTCELQKEVRSARCSFNGDFVCGQCV

CSEGWSGQTCNCSTGSLSDIQPCLREGEDKPCSGRGECQCGHCVCYGEGRYEGQFCEYDN

FQCPRTSGFLCNDRGRCSMGQCVCEPGWTGPSCDCPLSNATCIDSNGGICNGRGHCECGR

CHCHQQSLYTDTICEINYSAIHPGLCEDLRSCVQCQAWGTGEKKGRTCEECNFKVKMVDE

LKRAEEVVVRCSFRDEDDDCTYSYTMEGDGAPGPNSTVLVHKKKDCPPGSFWWLIPLLLL

LLPLLALLLLLCWKYCACCKACLALLPCCNRGHMVGFKEDHYMLRENLMASDHLDTPMLR

SGNLKGRDVVRWKVTNNMQRPGFATHAASINPTELVPYGLSLRLARLCTENLLKPDTREC

AQLRQEVEENLNEVYRQISGVHKLQQTKFRQQPNAGKKQDHTIVDTVLMAPRSAKPALLK

LTEKQVEQRAFHDLKVAPGYYTLTADQDARGMVEFQEGVELVDVRVPLFIRPEDDDEKQL

LVEAIDVPAGTATLGRRLVNITIIKEQARDVVSFEQPEFSVSRGDQVARIPVIRRVLDGG

KSQVSYRTQDGTAQGNRDYIPVEGELLFQPGEAWKELQVKLLELQEVDSLLRGRQVRRFH

VQLSNPKFGAHLGQPHSTTIIIRDPDELDRSFTSQMLSSQPPPHGDLGAPQNPNAKAAGS

RKIHFNWLPPSGKPMGYRVKYWIQGDSESEAHLLDSKVPSVELTNLYPYCDYEMKVCAYG

AQGEGPYSSLVSCRTHQEVPSEPGRLAFNVVSSTVTQLSWAEPAETNGEITAYEVCYGLV

NDDNRPIGPMKKVLVDNPKNRMLLIENLRESQPYRYTVKARNGAGWGPEREAIINLATQP

KRPMSIPIIPDIPIVDAQSGEDYDSFLMYSDDVLRSPSGSQRPSVSDDTGCGWKFEPLLG

EELDLRRVTWRLPPELIPRLSASSGRSSDAEAPHGPPDDGGAGGKGGSLPRSATPGPPGE

HLVNGRMDFAFPGSTNSLHRMTTTSAAAYGTHLSPHVPHRVLSTSSTLTRDYNSLTRSEH

SHSTTLPRDYSTLTSVSSHDSRLTAGVPDTPTRLVFSALGPTSLRVSWQEPRCERPLQGY

SVEYQLLNGGELHRLNIPNPAQTSVVVEDLLPNHSYVFRVRAQSQEGWGREREGVITIES

QVHPQSPLCPLPGSAFTLSTPSAPGPLVFTALSPDSLQLSWERPRRPNGDIVGYLVTCEM

AQGGGPATAFRVDGDSPESRLTVPGLSENVPYKFKVQARTTEGFGPEREGIITIESQDGG

PFPQLGSRAGLFQHPLQSEYSSITTTHTSATEPFLVDGLTLGAQHLEAGGSLTRHVTQEF

VSRTLTTSGTLSTHMDQQFFQT

>Mus musculus ITGB4

MAGPCCSPWVKLLLLAAMLSASLPGDLANRCKKAQVKSCTECIRVDKSCAYCTDELFKER

RCNTQAELLAAGCRGESILVMESSLEITENTQIDTSLHRSQVSPQGLQVRLRPGEERSFV

FQVFEPLESPVDLYILMDFSNSMSDDLDNLKQMGQNLAKILRQLTSDYTIGFGKFVDKVS

VPQTDMRPEKLKEPWPNSDPPFSFKNVISLTENVEEFWNKLQGERISGNLDAPEGGFDAI

LQTAVCTRDIGWRADSTHLLVFSTESAFHYEADGANVLAGIMNRNDEKCHLDASGAYTQY

KTQDYPSVPTLVRLLAKHNIIPIFAVTNYSYSYYEKLHKYFPVSSLGVLQEDSSNIVELL

EEAFYRIRSNLDIRALDSPRGLRTEVTSDTLQKTETGSFHIKRGEVGTYNVHLRAVEDID

GTHVCQLAKEDQGGNIHLKPSFSDGLRMDASVICDVCPCELQKEVRSARCHFRGDFMCGH

CVCNEGWSGKTCNCSTGSLSDTQPCLREGEDKPCSGHGECQCGRCVCYGEGRYEGHFCEY

DNFQCPRTSGFLCNDRGRCSMGECVCEPGWTGRSCDCPLSNATCIDSNGGICNGRGYCEC

GRCHCNQQSLYTDTTCEINYSAIRLGLCEDLRSCVQCQAWGTGEKKGRACDDCPFKVKMV

DELKKAEEVVEYCSFRDEDDDCTYSYNVEGDGSPGPNSTVLVHKKKDCPPGSFWWLIPLL

IFLLLLLALLLLLCWKYCACCKACLGLLPCCNRGHMVGFKEDHYMLRENLMASDHLDTPM

LRSGNLKGRDTVRWKITNNVQRPGFATHAASTSPTELVPYGLSLRLGRLCTENLMKPGTR

ECDQLRQEVEENLNEVYRQVSGAHKLQQTKFRQQPNTGKKQDHTIVDTVLLAPRSAKQML

LKLTEKQVEQGSFHELKVAPGYYTVTAEQDARGMVEFQEGVELVDVRVPLFIRPEDDDEK

QLLVEAIDVPVGTATLGRRLVNITIIKEQASGVVSFEQPEYSVSRGDQVARIPVIRHILD

NGKSQVSYSTQDNTAHGHRDYVPVEGELLFHPGETWKELQVKLLELQEVDSLLRGRQVRR

FQVQLSNPKFGARLGQPSTTTVILDETDRSLINQTLSSPPPPHGDLGAPQNPNAKAAGSR

KIHFNWLPPPGKPMGYRVKYWIQGDSESEAHLLDSKVPSVELTNLYPYCDYEMKVCAYGA

QGEGPYSSLVSCRTHQEVPSEPGRLAFNVVSSTVTQLSWAEPAETNGEITAYEVCYGLVN

EDNRPIGPMKKVLVDNPKNRMLLIENLRESQPYRYTVKARNGAGWGPEREAIINLATQPK

RPMSIPIIPDIPIVDAQGGEDYENFLMYSDDVLRSPASSQRPSVSDDTEHLVNGRMDFAY

PGSANSLHRMTAANVAYGTHLSPHLSHRVLSTSSTLTRDYHSLTRTEHSHSGTLPRDYST

LTSLSSQGLPPIWEDGRSRLPLSWTLGSLSRAHMKGVPASRGSPDSIILAGQSAAPSWGT

DSRGAVGVPDTPTRLVFSALGPTSLKVSWQEPQCDRMLLGYSVEYQLLNGGEMHRLNIPN

PGQTSVVVEDLLPNHSYVFRVRAQSQEGWGREREGVITIESQVHPQSPLCPLPGSAFTLS

TPSAPGPLVFTALSPDSLQLSWERPRRPNGDILGYLVTCEMAQGGAPARTFRVDGDNPES

RLTVPGLSENVPYKFKVQARTTEGFGPEREGIITIESQVGGPFPQLGSHSGLFQNPVQSE

FSSVTSTHSTTTEPFLMDGLTLGTQRLEAGGSLTRHVTQEFVTRTLTASGSLSTHMDQQF

FQT

>Rattus norvegicus ITGB4

MAGLCSSPWVKLLLAVVLSAGLPGNMANRCKKAQVKSCTECIRVDKSCAYCTDELFKERR

CNTQAELLAAGCRGESVLVMESSLEITENIQIDTSLHRSQVSPQGLQVRLRPGEERNFVF

KVFEPLESPVDLYILMDFSNSMSDDLDNLKQMGQNLAKILRQLTSDYTIGFGKFVDKVSV

PQTDMRPEKLKEPWPNSDPPFSFKNVISLTENVEEFWDKLQGERISGNLDAPEGGFDAIL

QTAVCTRDIGWRADSTHLLVFSTESAFHYEADGANVLAGIMNRNDEKCHLDATGAYTQYK

TQDYPSVPTLVRLLAKHNIIPIFAVTNYSYSYYEKLHKYFPVSSLGVLQEDSSNIVELLE

EAFYRIRSNLDIRALDSPRGLRTEVTSDTLQKTETGSFHIKRGEVGTYNVHLRAVEDIDG

THVCQMAKEDQRGNIHLKPSFSDGLRMDASVICDVCACELQKEVQSARCHYRGDFMCGHC

VCNEGWSGKTCNCSTGSLSDTQPCLREGEDKPCSGHGECQCGRCVCYGEGRYEGHFCEYD

NFQCPRTSGFLCNDRGRCSMGECVCEPGWTGRSCDCPLSNATCIDSNGGICNGLGFCECG

RCHCNQRSSLYTDTTCEINYSAIRLGLCEDLRSCVQCQAWGTGEKKGRTCEECNFKVKMV

DELKKAEEVVEYCSFRDEDDDCTYSYTVEGDGSPGPNSTVLVHKKKDCPPGSFWWLIPLL

IFLLLLLVLLLLLCWKYCACCKACLGLLPCCNQGHMVGFKEDHYMLRENLMASDHLDTPM

LRSGNLKGRDTVRWKITNNVQRPGFATHAASISPTELVPYGLSLRLGRLCTENLMKPGTR

ECDQLRQEVEENLNEVYRQVNGVHKLQQTKFRQQPNAGKKQDHTIVDTVLLAPRSAKQSL

LKLTEKQVEQGSFHELKVAPGYYTLTAEQDARGMVEFQEGVELVDVRVPLFIRPEDDDEK

QLLVEAIDVPVGTATLGRRLVNITIIKEQASGIVSFEQPEYSVSRGDQVARIPVIRHILD

NGKSQVSYSTQDNTAHGHRDYVPVEGELLFYPGETWKELQVKLLELQEVDSLLRGRQVRR

FQVQLSNPKFGARLGQPNTATVIIGEQDETDRSLINEISASPPLPRGDLGAPQNPNAKAA

GSRKIHFNWLPPPGKPMGYRVKYWVQGDSESEAHLLDSKVPSVELTNLYPYCDYEMKVCA

YGAHGEGPYSSLVSCRTHQEVPSEPGRLAFNVVSSTVTQLSWAEPAETNGEITAYEVCYG

LVNEDNRPIGPMKKVLVDNPKNRMLLIENLRESQPYRYTVKARNGAGWGPEREAIINLAT

QPKRPMSIPIIPDIPIVDAQGGEDYENFLMYSDDVLRSPASSQRPSVSDDTEHLVNGRMD

FAYPGSANSLHRMTAANVAYGTHLSPHQTHRMLSTSSTLTRDYHSLTRTDHSQSGTLPRD

YSTLTSLSSQGLPPIWEDGRSRLPLSWTLGSWSRAQMKGVPASRGSPDSIILAGQSAAPS

WGTDSRGAMGVPDTPTRLVFSALGPTSLKVSWQEPQCDRALLGYSVEYQLLNGGEMHRLN

IPNPGQTSVVVEDLLPNHSYVFRVRAQSQEGWGREREGVITIESQVHPQSPLCPLPGSAF

TLSTPSAPGPLVFTALSPDSLQLSWERPRRPNGDILGYLVTCEMAQGGGPARTFRVDGDN

PESRLTVPGLSENVPYKFKVQARTTEGFGPEREGIITIESQDGGPFPQLGSHSGLFQNPL

QSEYSTVTSTHSTTTTEPFLIDGLTLGTQRLEAGGSLTRHVTQEFVSRTLTTSGSLSTHM

DQQFFQT

>Tetraodon nigroviridis ITGB4

RLAKGCAYCTEETFNGPRCDLQENILAHGCGAAAIITAQSTMEIVKKEQINMKLQQSQVS

PQQIRMNFLPGEEKMVDVEVFAPTKGPLDLYILMDFSNSMADDLDNLKRMGNELATLVEQ

LSDDYTIGFGKFVDKVIEPQTDMRPEKLKKPWPDSDPPFSFQNVIKLTNDLNFFTAKLND

ETISGNLDAPEGGFDAILQAAVCGEKIGWRSDSTHLLVFSTESAFHYETDGVNVLSGILK

RNDEQCHLDSAGKYTEDIHQDYPSIPTLVRLLGKHNIIPIFAVTNHSYTYYQKLKEYFPI

AEVGLLQEDSSNILQVMETAFKSIRSKMSIRAENRPKAFEAQFLSAGGKLSEYGDFDFTP

GAIGKFKMRLKAQRTIDDTPVCDAAQAEKYGTMRVKPTTFTKAVNVNADVLCPACDCEKI

RVENAPRCHGNGRLVCGKCECYSGWLSTFCNCSASAALVTGQCIGPDMKEPCSGRGDCME

CGTCVCYNPEQFEGPFCEYDKTQCQRYGGFLCNDRGSCVMGKCACTEGWEGNACECPKSK

QTCLDSKGGICNGRGKCVCGLCECPNSGIELTSTCEPNFQAQLGACEATRSCVQCQAWKT

GEKKDKKDCDTCPFTVVMVDELKESDKVLDSCSFRDEDDDCTYHYTVENPKDKDLEVQVL

KKKDCPPASLLWLLPLLLFLLLLLALFLLCCWKYCAGCKPCCQGCLALLPCCRRGRTVGW

KEDEYLFRQSLLTSDHLDTPMVRSGPPKGTDVVRWRVTDNVHRGLNHPQAVAKPNPKEMI

PFPLSVRLDKLFFDNLARPESKEAEQLRSEVADNLNELYKQIPGAQKVQKTSFRMQRNAG

KRQDYTIADTVLSAPRSSFPDIVKLTEKNVQSGRMNNLKVVPGYYTIETDREAMGAIEFQ

EGVESLDVHVPLFVKEEDDDKKKLQLEAVDVPLGIAKIAKRFVNITIIKDRANSIFSFPQ

PAYTYSRRDGVINIPVSREIIEDGRTQVTYRTRDLTAKDKLDYVTVEGDLTYLPGETQKN

VSVRLLELGEKDGLLEDKQPKQFVMDLSNPLVGAKLGRYQRTTITIADQPEPSVVMFKKS

THNFPTTHPTFTIPVVRSRNQDSPATVKWRTKKAQRFDLSGPEISQKQQKGYVSPKATGP

GGLLYAPSNPQAKATGPRSIQLNWEPPPGNPMGYKVKYWIYGDPEKDAPVLDVKSPRADL

TNLYPYCDYEMRVCAYNENGDGYDTDIVTCQTPEDAPGKPGRLAFNVISPTVTQLSWAEP

AETNGNITAYEVIYTPIDDELILISSNVPAEPVGAPKKVQIDNAKKRMLLIENLQNAQTY

QYKVRAKNSVGWGPFRDASINLASQPNRPLSIPIIPDIPIVDAEAGDEYDSYLMYSNEVL

RSPTSSKSPSVSGDDFFVNTKWEQSLLSPGGSGPRNLSASSSPMSTLSSNYRAAGGSYNT

EVTTTYLSGPGSSHRPNVSGGLHTSEVTMRKRSENRVYTDENVRDSIVMGDLSSNFPDFG

GFSFSRMQSSPKSPLSYSQGPRARTQSSDINEALYNLDRVLHDARLSPGVPDTPSRLVFS

ALGPTALKVSWQEPHCERDIMGYCVIYQLLSGGEVKRINVTNPAENSVIIQDLLPNQSYL

FKVKAQSQEGWGPEREGVITIESAVDPRSPLCPMPGSPFTLSTPSAPGPLIFTALSPESL

QLSWEKPRKPNGEIRGYVVTCEQLHGGGDVRTFQVHGNSAETSLTVPDLVENVPYKFKVQ

ARTTEGFGPEREGIITIESQDGS

>Takifugu rubripes ITGB4

PTPTENYCFSSRAKSCSECLQAGKGCAYCSEETFNGPRCDLQENILAHGCSAAAMITAQS

SRNVEKGEQINMQLLKSQVSPQLMSMSFLPGEEKMVDVEVFAPTKGPLDLYILMDFSNSM

SDDLDNLKRMGKELASLVEQLSDDYTIGFGKFVDKVIEPQTDMRPAKLAKPWANSDPPFS

FQNVINLTSNLEFFNTKLKNETISGNLDAPEGGFDAILQAAVCGDRIGWRSNSTHLLVFS

TESAFHYETDGANVLSGIVKRNDEGCHLDPAGKYTQDIYQDYPSIPTLVRLLGKHNIIPI

FAVTNHSYTYYQKLKEYFPIAEVGLLQEDSSNILQVMETAFESIRSKMSIRAEDRPKAFE

AQFFSSSKKLSKYGDFDFKPGEIGKFKMRLKAQRMIDDGPVCQTEQDEREGTIRVKPTTF

NDAVNVKASLLCPTCECEKTVVQNAPRCHGQGDLVCGKCQCHDGWLSTFCNCSASASALV

TGQCIGPGMTEPCSGRGDCMECGTCVCYNPEQFEGPYCEYDKTQCQRYGGFLCNDRGSCV

MGKCTCTEGWEGNACECPKSNQTCLDSKGGVCNDRGKCLCGRCACPSSGIEMTSTCIFGV

IAQLGACEATRSCVQCQAWKTGEKKDKKDCDACPFTVVMVDELKKSDQVLDSCSFRDEDD

DCTYHYTVENPKDKDLEVQVLKKKDCPPASLLWLLPLLLFLLLLLALLLLCCWKYCACCK

TCWQSCLALLPCCRRGRMVGWKEDEYLFRQSLLTSDHLDTPMVRTGPPKGTDVVRWRVTD

NVHRGPNHPQAIVKPNPKEMIPFPLSLRLDKLFFDNLARPESKDAEQLRSEVADNLNELY

KQIPSAQKVQKTAFRMQRNAGKRQDYTIVDTVLSAPRSSFPDIVKLTEKNVQSGRMHNLK

VVPGYYTVETDREAMGAIEFQEGVESLDVHVPLFVKEEDDDKKKLQLEAVDVPLGIADIA

KRFVNITIIKDHASSIFSFLQPAYTYSRQDGLVNIPVSREIIEDGRTQVTYRTRDLTAKD

KLDYVTVEGDLNYGPGETQKTVPVRLLELGEKDGLMEDKQIKQFVMDLSNPRVGAKLGRY

PRTTITIADEPEISQKQQKGYISPKASLPGGFLSAPSNPKAKATGPRSIHLNWDPPPGNP

MGYKVKYWIYGDPEKDAQVLDVKSPRADLTNLYPFCDYEMRVCAYNTKGDGYETDIVTCQ

TLEDVPGEPGRLAFNVISPTVTQLSWAEPAETNGNITAYEVIYTPIDDELSPVGPPKKVQ

IDNPKKRMLLIENLQNAQTYQYKVRAKNSVGWGPFRDASINLASQPNRPLSIPIIPDIPI

VDAEAGDEYDSYLMYSNEVLKSPTGSKTPSVSGDGGYPETKPHCDRLLDLYWNFLEPLGL

ERLALSLPAFLISNSSVKHISMQHQVVAAILLWCFESSSGVSLNTVICNLVLNVSRLKLG

VYILFLPISKINFAYYLLNYLFTPYLLDARLSPGVPDTPSRLVFSALGPTALKVSWQEPH

CERDILGYCIIYQLLNGGEVKRINVTNPAENSVIIQDLLPNHSYLFKVKAQSQEGWGPER

EGVITIESAVDPRSPLSPMPGSPFTLSTPSAPGPLVFTALSPDSLQLSWEKPRKPNGDIL

GYVVTCEQLHGGGDVRTFQVNGDSTEMSLTVPDLVENVPYKFKVQARTTQGFGPEREGII

TIESQDGSALSQYNNQSMVRRDVFHMPTEVSTRTNVSHTVVNDPFFSEGMMMTTQHMETS

GTVTRQVTKEVVQRSVMGGTTITKKM

>Danio rerio ITGB4

NHCTAARTKTCSECIQVGAGCAYCPDEVFEHERCDLKENLEAQKCSKIVNFESSRQIQEN

VKINEQLKQSQVSPQLMSMTLLPGEEKEVEMEVFEPTRGPLDLYILMDFSNSMSDDLDNL

KRMGEKLARLVGDLSDDYTIGFGKFVDKVTEPQTDMRPAKLAEPWSNSDPPFSFRNVIKL

TSNITSFRQKLQKERISGNLDAPEGGFDAILQTAVCQDQIGWRKDSTHLLVFSTESAFHY

EADGANVLAGILDRNDEQCHLNVDGNYTHDVRQDYPSIPTLVRLLVKHNIIPIFAVTNHS

YSYYEKLHEYFPIAELGQLQEDSSNILSILEKANVKTLPHCFQGKFKVRMKALNQVGEEP

VCKVNQADRAGTLRVKPTTFSSAFKINAEVLCPTCDCEKTPVKNAVRCTGHGDLVCGKCQ

CYAGWYQLGPLCNCSAGASTDSVMCIERGKTEPCSGRGDCMCGTCVCYNPNQFEGPYCQF

DKSQCQRFGGFLCNVTERGSCSMGRCVCSPGWSGEACECPTSNDSCRDSKGGICNDRGVC

KCGRCECEHSGLPLSPTCEANFQ

>Strongylocentrotus purpuratus(integrin beta1)

MPSVRSPHRLNRLGSVVVFFLTFVLGVFTVHANEELSSICQGAQNCGECITVNPECTWCT

EDTFQGRRCDLESRLQDAGCSNITNPLSSADATQDDPLSEANADLDEIVQVKPQRMKIKV

RPNEPIDIRLYVRQAADYPVDLYYAMDLSNSMRDDLENLKGLGTTLSKVLSNITGDFRLG

FGSFVDKRILPFVNTLPDRLIFPCPESSESCARTHGFFNALPLNEDSTLFANLIANTIIS

GNLDTPEGGFDALMQIAVCGNIIGWRPKARHLVIFTTDAPFHIAGDGRLGGLVEPNDGQC

HTDPNTNMYTFSTLQDYPSIGHLSAKLRENNVIPIFAVTSRRTNLYMKLENYIEGATVGT

LDVDSGNIVQLIQSNYDRITSQVRLTSTAPDDVTLSYRANCIDQTYEDTNECSGLKLEDT

VSFDITITAERCVEGGMTSFEIGPVGFNEELKIELEVTCECDCQGLGEANSNVCSNGNGT

LVCGECACNPGRYGVKCECSGNEISMESTDTSQCRTDNTTRTCSGRGECICGKCVCDNTG

IPGEVISGQFCECDNFNCPYSRGLRCGGPDQGMCVCDVATRQPKCRCEPGFKGDSCDCPT

RKDTCRASNGLECNARGTCECGVCECIEDSQFQGKTCERCTACPSGNCHIHRDCVQCTVF

ESGRLTPEQCGMCSIDIINVTSIDEYTKDNPICDFMLNGICTFRFVVVSENETVTVYVEG

EPTCITPNPRKPRLTPTEIRWIIIGIILGIVLIGIILVCAWRLYINIQDRREYAQWKKES

QKPKWAQVRLEPLSLFAVCP

>Drosophila melanogaster(CG1560-RA)

MILERNRRCQLALLMIAILAAIAGQTDAQKAAKLTAVSTCASKEKCHTCIQTEGCAWCMQ

PDFKGQSRCYQNTSSLCPEEFAYSPITVEQILVNNKLTNQYKAELAAGGGGSAMSGSSSS

SYSSSSSSSSFYSQSSSGSSSASGYEEYSAGEIVQIQPQSMRLALRVNEKHNIKISYSQA

EGYPVDLYYLMDLSKSMEDDKAKLSTLGDKLSETMKRITNNFHLGFGSFVDKVLMPYVST

IPKKLEHPCENCKAPYGYQNHMPLNNNTESFSNEVKNATVSGNLDAPEGGFDAIMQAIAC

RSQIGWREQARRLLVFSTDAGFHYAGDGKLGGVIAPNDGECHLSPKGEYTHSTLQDYPSI

SQINQKVKDNAINIIFAVTASQLSVYEKLVEHIQGSSAAKLDNDSSNVVELVKEEYRKIS

SSVEMKDNATGDVKITYFSSCLSNGPEVQTSKCDNLKEGQQVSFTAQIQLLKCPEDPRDW

TQTIHISPVGINEVMQIQLTMLCSCPCENPGSIGYQVQANSCSGHGTSMCGICNCDDSYF

GNKCECSATDLTSKFANDTSCRADSTSTTDCSGRGHCVCGACECHKRPNPIEIISGKHCE

CDNFSCERNRNQLCSGPDHGTCECGRCKCKPGWTGSNCGCQESNDTCMPPGGGEICSGHG

TCECGVCKCTVNDQGRFSGRHCEKCPTCSGRCQELKDCVQCQMYKTGELKNGDDCARNCT

QFVPVGVEKVEIDETKDEQMCKFFDEDDCKFMFKYSEQGELHVYAQENKECPAKVFMLGI

VMGVIAAIVLVGLAILLLWKLLTTIHDRREFARFEKERMNAKWDTGENPIYKQATSTFKN

PMYAGK

**Myosin light chain family**

>Homo sapiens MYL7

MASRKAGTRGKVAATKQAQRGSSNVFSMFEQAQIQEFKEAFSCIDQNRDGIICKADLRET

YSQLGKVSVPEEELDAMLQEGKGPINFTVFLTLFGEKLNGTDPEEAILSAFRMFDPSGKG

VVNKDEFKQLLLTQADKFSPAEVEQMFALTPMDLAGNIDYKSLCYIITHGDEKEE

>Mus musculus MYL7

MASRKAGTRGKAAATKQAQRGSSNVFSMFEQAQIQEFKEAFSCIDQNRDGIICKSDLKET

YSQLGEVSVPEEELDAMLQEGKGPINFTVFLTLFGEKLNGTDPEEAILSAFRMFDPSGQG

VVNKEEFKQLLMTQADKFSPAEVEQLFALTPMDLAGNIDYKSLCYIITHGDEKEE

>Rattus norvegicus MYL7

ASRKAGTRGKAAATKQAQRGSSNVFSMFEQAQIQEFKEAFSCIDQNRDGIICKSDLKETY

SQLGRVSVPEEELDAMLQEGKGPINFTVFLTLFGEKLNGTDPEEAILSAFRMFDPSGQGV

VNKEEFKQLLMTQADKFSPAEVEQLFALTPMDLTGNIDYKSLCYIITHGDEKEE

>Bos taurus MYL7

QASRKAGTRGKAAATKQAQRGSSNVFSMFEQAQIQEFKEAFSCIDQNRDGIICKSDLRET

YSQLGKVNVPEEELDAMLQEGKGPINFTVFLTLFGEKLNGTDPEEAILSAFRLFDPSGKG

VVNKDEFRQLLLTQADKFSPAEVEQMFALTPMDLAGNIDYKSLCYIITHGDEKEE

>Macaca mulatta MYL7

MASRKAGTRGKVAATKQAQRGSSNVFSMFEQAQIQEFKEAFSCIDQNRDGIICKADLRET

YSQLGKVSVPEEELDAMLQEGKGPINFTVFLTLFGEKLNGTDPEEAILSAFRMFDPSGKG

VVNKEEFKQLLLTQADKFSPAEVEQMFALTPMDLAGNIDYKSLCYIITHGDEKEE

>Canis familiaris MYL7

FSCIDQNRDGIICKSDLRETYSQLGKVSIPEEELDAMLQEGKGPINFTVFLTLFGEKLNG

TDPEEAILSAFRMFDPSGKGVVNKDEFKQLLLTQADKFSPAEVEQMFALTPMDLAGDIDY

KSLCYIITHGDEKEE

>Tetraodon nigroviridis MYL7

QKSCSNVFSMFEQSQIQEFKEAFGCIDQDRDGVIKKQDLKETYAQLGKLNVKDEELDEML

NEGKGPINFTVFLSLFGEKLNGTDPEDTILAAFKLFDPNGTGFVNKDEFRRLLMNQADKF

TAEEVDQAFAVAPIDPTGNIDYKSLCYIITHGDEKEES

>Takifugu rubripes MYL7

ASKKASNKRQRGQKSCSNVFSMFEQSQIQEFKEAFGCIDQDRDGVIKKQDLKETYAQLGK

LNVKDEELDEMLNEGKGPINFTVFLSLFGEKLNGTDPEDTILAAFKLFDPNGTGFVNKDE

FRRLLMNQADKFTAEEVDQAFAVAPIDPTGNIDYKSLCYIITHGDEKEE

>Danio rerio MYL7

MASKKAAAKRGKTAQRGSSNVFSMFEQSQIQEFKEAFGCIDQNRDGVINKSDLKETYAQL

GKLNVSDEELESMLTEGKGPINFTVFLTLFGEKLNGTDPEETILAAFKLFDPNATGVVNK

DEFKRLLMTQADKFTAEEVDQAFAVAPIDVAGNIDYKSLCYIITHGDEKEES

>Homo sapiens MYL2

MAPKKAKKRAGGANSNVFSMFEQTQIQEFKEAFTIMDQNRDGFIDKNDLRDTFAALGRVN

VKNEEIDEMIKEAPGPINFTVFLTMFGEKLKGADPEETILNAFKVFDPEGKGVLKADYVR

EMLTTQAERFSKEEVDQMFAAFPPDVTGNLDYKNLVHIITHGEEKD

>Mus musculus MYL2

MAPKKAKKRIEGGSSNVFSMFEQTQIQEFKEAFTIMDQNRDGFIDKNDLRDTFAALGRVN

VKNEEIDEMIKEAPGPINFTVFLTMFGEKLKGADPEETILNAFKVFDPEGKGSLKADYVR

EMLTTQAERFSKEEIDQMFAAFPPDVTGNLDYKNLVHIITHGEEKD

> Canis familiaris MYL2

LLGKQFCVLSTMAPKKAKKRAEGANSNVFSMFEQTQIQEFKEAFTIMDQNRDGFIDKNDL

RDTFAALGRVNVKNEEIDEMLKEAPGPINFTVFLTMFGEKLKGADPEETILNAFKVFDPE

GKGVLRADYVREMLTTQAERFSKEEIDQMFAAFPPDVTGNLDYKNLVHIITHGEEKD

> Monodelphis domestica MYL2

QAPKKAKKRAEGANSNVFSMFEQTQIQEFKEAFTIMDQNRDGFIDKADLRDTFAALGRVN

VKNEEIDEMIKEAPGPINFTVFLTMFGEKLKGADPEETILNAFKVFDPEGKGVLKSDYIQ

EMLTTQAERFSKEEVDQMFAAFPPDVTGNLDYKNLVHIITHGEEKD

> Macaca mulatta MYL2

MAPKKAKKRAQGANSNVFSMFEQTQIQEFKEAFTIMDQNRDGFIDKNDLRDTFAALGRVN

VKNEEIDEMIKEAPGPINFTVFLTMFGEKLKGADPEETILNAFKVFDPEGKGVLKADYVR

DMLTTQAERFSKEEIDQMFAAFPPDVTGNLNYKNLVHIITHGEEKD

>Gallus gallus MYL2

TPQAPKKAKKRIEGANSNVFSMFEQAQIQEFKEAFTIMDQNRDGFIDKADLRDTFAALGR

LNVKNEEIDEMIKEAPGPINFTVFLTMFGEKLKGADPEETILNAFKVFDPEGKGLKSAYI

KEMLMTQGERFSQEEIDQMFAAFPPDVSGNLDYKNLVHVITHGEEKD

>Tetraodon nigroviridis MYL2

APKKAKKRTAEGANSNVFSMFEQAQIQEFKEAFTIMDQNRDGFIDKNDLRDTFAALGRLN

VKQEELDEMLKEAPGPINFTVFLTMFGEKLKGADPEETILNAFKVFDPEGKGVLRKDYVT

QMLTTQADRFSSEEMEQMFTAFPPDVAGNLDYKNLVHIITHGEEKD

> Takifugu rubripes MYL2

APKKAKRRTAEGANSNVFSMFEQAQIQEFKEAFTIMDQNRDGFIDKNDLRDTFAALGRLN

VKQEELDEMLKEAPGPINFTVFLTMFGEKLKGADPEETILNAFKVFDPEGKGVLRKDYVT

QMLTTQADRFSKEEMDQMFTAFPPDVAGNLDYKNLVHIITHGEEKD

>Danio rerio MYL2

QAPKKAKKRAEGANSNVFSMFEQAQIQEFKEAFTIMDQNRDGFIDKNDLRDTFAALGRLN

VKQEELDEMLKEAPGPINFTVFLTMFGEKLKETHAGADAEETILNAFKVFDPEGKGTLRK

DFLSRMLTTQADRFSPEEMEQMFSAFPPDAAGNLDYKNLVYIITHGEEKDQE

>Homo sapiens MYL1

MAPKKDVKKPVAAAAAAPAPAPAPAPAPAPAKPKEEKIDLSAIKIEFSKEQQDEFKEAFL

LFDRTGDSKITLSQVGDVLRALGTNPTNAEVRKVLGNPSNEELNAKKIEFEQFLPMMQAI

SNNKDQATYEDFVEGLRVFDKEGNGTVMGAELRHVLATLGEKMKEEEVEALMAGQEDSNG

CINYEAFVKHIMSI

>Mus musculus MYL1

MAPKKDVKKPAAAPAPAPAPAPAPAKPKEEKIDLSAIKIEFSKEQQEDFKEAFLLFDRTG

ECKITLSQVGDVLRALGTNPTNAEVKKVLGNPSNEEMNAKKIEFEQFLPMMQAISNNKDQ

GGYEDFVEGLRVFDKEGNGTVMGAELRHVLATLGEKMKEEEVEALLAGQEDSNGCINYEA

FVKHIMSV

>Rattus norvegicus MYL1

MAPKKDVKKPAAAAPAPAPAPAPAPAKPKEEKIDLSAIKIEFSKEQQEEFKEAFLLFDRT

GECKITLSQVGDVLRALGTNPTNAEVKKVLGNPSNEEMNAKKIEFEQFLPMMQAISNNKD

QGGYEDFVEGLRVFDKEGNGTVMGAELRHVLATLGEKMKEEEVEALLAGQEDSNGCINYE

AFVKHIMSV

>Gallus gallus MYL1

MAPKKDVKKPAAAAAPAPAPAPAPAPAPAKPKEPAIDLKSIKIEFSKEQQDDFKEAFLLF

DRTGDAKITLSQVGDIVRALGQNPTNAEINKILGNPSKEEMNAKKITFEEFLPMLQAAAN

NKDQGTFEDFVEGLRVFDKEGNGTVMGAELRHVLATLGEKMTEEEVEELMKGQEDSNGCI

NYEAFVKHIMSV

>Macaca mulatta MYL1

MAPKKDVKKPAAAAAPAPAPAPAPAPAPAKPKEEKIDLSAIKIEFSKEQQDXXXXXXXXX

XXXXXXXXXXXXXXXXXXXXXXXXXXXXXXXXXXXXXXXXLNAKKIEFEQFLPMMQAISN

NKDQGTYEDFVEGLRVFDKEGNGTVMGAELRHVLATLGEKMKEEEVEALMAGQEDSNGCI

NYEAFVKHIMSI

>Takifugu rubripes MYL1

MAPKKDPKAPVKKEAPAKKAEAAPAPAPAPEPAPAPKPAAVDLSAVKIEFSPDQIEDYRE

AFGLFDRVGDNKVAYNQIADIMRALGQNPTNKEVTKMLGNPNTEDMANKRVEFEGFLPML

QTIINSPNKAGFEDYVEGLRVFDKEGNGTVMGAELRIVLSTLGEKMNEAEIDALMAGQED

ENGCVNYEAFVKHIMSV

>Danio rerio MYL1

MAPKKDAKKPEPPKKAEPAPAPAPAPEPPKADAVDLSGVKLDFTQDQMEDYREAFLLFDR

VGDSKVAYNQIADIMRALGQNPTNKEVTKILGNPTADDMVNKRVDFEGFLPMLQVVINNP

NKATYDDYVEGLRVFDKEGNGTVMGAELRIVLSTLGEKMSEAEIDALMQGQEDENGCVNY

EAFVKHIMSV

>Homo sapiens MYL4

MAPKKPEPKKEAAKPAPAPAPAPAPAPAPAPEAPKEPAFDPKSVKIDFTADQIEEFKEAF

SLFDRTPTGEMKITYGQCGDVLRALGQNPTNAEVLRVLGKPKPEEMNVKMLDFETFLPIL

QHISRNKEQGTYEDFVEGLRVFDKESNGTVMGAELRHVLATLGEKMTEAEVEQLLAGQED

ANGCINYEAFVKHIMSG

>Mus musculus MYL4

MPPKKPEPKKETAKPAAAPAPAASAAPEPLKDSAFDPKSVKIDFSADQIEEFKEAFSLFD

RTPTGEMKITYGQCGDVLRALGQNPTNAEVLRVLGKPKPEEMSSKTLDFEMFLPILQHIS

RNKEQGTYEDFVEGLRVFDKESNGTVMGAELRHVLATLGEKMSEAEVEQLLSGQEDANGC

INYEAFVKHIMSG

>Rattus norvegicus MYL4

MPPKKPEPKKETAKVAAAPAPAPAPAPEPLRDSAFDPKSVKIDFSADQIEEFKEAFSLFD

RTPTGEMKITYGQCGDVLRALGQNPTNAEVLRVLGKPKPEEMNSKTLDFEMFLPILQHIS

RNKEQGTYEDFVEGLRVFDKESNGTVMGAELRHVLATLGEKMSEAEVEQLLTGQEDANGC

INYEAFVKHVMSG

>Gallus gallus MYL4

QQPPPRIAPPGRRSHPRAMPLKKPDPKKDAAKAAAAPEVPKEFTFDPKSVKIEFAAEQIE

EFKEAFSLFDRTPTGAMQITYAQCGDVLRALGHNPTNAEVLKVLGKPKPEDMNTKMLDFE

TFLPILQHFTRNREQGTFEDFVEGLRVFDKEGNGLVMGAELRHVLVTLGEKMTESEVEQL

MAGQEDANGCINYEAFVKHIMSG

>Bos taurus MYL4

TCSSTSLFSAEFKEAFSLFDRTPTGELKIAYGQCGDVLRALGQNPTNAEVLRVLGKPKPE

GPLPEEMNSKMLDFETFLPILQHISRNKEQGTYEDFVEGLRVFDKESNGTVMGAELRHVL

ATLGEKMSEAEVEQLLAGQEDANGCINYEAFVKHIMSG

>Takifugu rubripes MYL4

MPPKKIEPKKPEPKKPELKKDVAPAAKPAPPPEPLPEQPKEPDFDPKSITLEFSSDQIEE

FKEAFTLFDRTPTGEMKITYAQCGDVMRALGQNPTNADVLKVLGKPRPEDMSTKMVDFET

FLPMLQHISRKKDQGNFEDFVEGLRVFDKEGNGTIMGAELRHVLATLGERMTEDEVDRLM

AGQEDANGCINYASFVKHILSG

>Tetraodon nigroviridis MYL4

MKNPRAPEVDTEDISIRRRYLGEAAPPPEPLPEQPKEPDFDPKSITLEFTSDQIEEFKEA

FTLFDRTPTGEMKITYAQCGDVMRALGQNPTNADVLKVLGKPRPEDMSTKMVDFETFLPM

LQHISRKKDQGNFEDFVEGLRVFDKEGNGTIMGAELRHVLATLGERMTEDEVDRLMAGQE

DANGCINYASFVKHILSG

>Danio rerio MYL4

MAPKKVEPKKPEPKKPEAKKEEAPAPAPVPETPKEPEVDLKSVPLDFSPDQIEEFRDAFT

LFDETPTGEMKIRYAQCGDVMRALGHNPTNADVLTVLGKPKAEEMNTKYLDFETFLPMLQ

HISRAKDQGTFEDFVEGLRVFDKEGNGTVMGAELRHVLATLGEKMTEDEVDRLMAGQEDA

NGCINYTSFIKHILSG

>Homo sapiens MYL6

MCDFTEDQTAEFKEAFQLFDRTGDGKILYSQCGDVMRALGQNPTNAEVLKVLGNPKSDEM

NVKVLDFEHFLPMLQTVAKNKDQGTYEDYVEGLRVFDKEGNGTVMGAEIRHVLVTLGEKM

TEEEVEMLVAGHEDSNGCINYEAFVRHILSG

>Mus musculus MYL6

CDFTEDQTAEFKEAFQLFDRTGDGKILYSQRGDVMRALGQNPTNAEVLKVLGNPKSDEMN

VKVLDFEHFLPMLQTVAKNKDQGTYEDYVEGLRVFDKEGNGTVMGAEIRHVLVTLGEKMT

EEEVEMLVAGHEDSNGCINYEELVWTVLNG

>Rattus norvegicus MYL6

MCDFTEDQTAEFKEAFQLFDRTGDGKILYSQCGDVMRALGQNPTNAEVLKVLGNPKSDEM

NVKVLDFEHFLPMLQTVAKNKDQGTYEDYVEGLRVFDKEGNGTVMGAEIRHVLVTLGEKM

TEEEVEMLVAGHEDSNGCINYEELVRMVLNG

>Xenopus tropicalis MYL6

MCDYSDDQIADYKESFQLFDRVGDGKILFGQCGDVMRALGQNPTNAEVMKVLGNPKPEDM

NIKTLDFEQFLPMMQTVAKNRDVPGLEDIIEGLRVFDKEGNGTVMGSELRHVLVSLGEKM

TDDEVETLLSNHEDANGCINYEELIRAILNG

>Gallus gallus MYL6

MCDFSEEQTAEFKEAFQLFDRTGDGKILYSQCGDVMRALGQNPTNAEVMKVLGNPKSDEM

NLKTLKFEQFLPMMQTIAKNKDQGCFEDYVEGLRVFDKEGNGTVMGAEIRHVLVTLGEKM

TEEEVEQLVAGHEDSNGCINYEAFVRHILSG

>Bos taurus MYL6

MCDFTEDQTAEFKEAFQLFDRTGDGKILYSQCGDVMRALGQNPTNAEVLKVLGNPKSDEM

NVKVLDFEHFLPMLQTVAKNKDQGTYEDYVEGLRVFDKEGNGTVMGAEIRHVLVTLGEKM

TEEEVEMLVAGHEDSNGCINYEAFVRHILSG

>Takifugu rubripes MYL6

EFKEAFYLFDRTGDGKITYSQCGDVMRALGQNPINADVLKVLGNPKIEEMNHKLLDFEQF

LPKLQDIAKNKDQGSFEDIVEGLRVFDKEGNGTVMGAELRHVLTTLGEKMTEEEVETLLA

GHEDANGCINYEELVRMVMNG

>Danio rerio MYL6

MSDFTEDQICEFKEAFLLFDRTGDGKIMYNQCGDVMRALGQNPVNAEVLKVLGNPSNEDM

NMKMLDFEQFLPMLQAIAKNKDQGSFEDFVEGLRVFDKEGNGTVMGAELRHVLTTLGEKM

TEEEVETLLAGHEDANGCINYEAFVRHIMAG

>Apis mellifera XP_623504)

MASYSEDQLAEFQEAFQLFDSRGDGKIHVAQIGDALRALGQNPTESDVKKFTHQHKPDER

ISFEVFLPIYQAISKSRTSDTADDFIEGLRHFDKDGNGFISSAELRHLLTTLGEKLSDEE

VETLLAGHEDSQGNINYEDFVRQVMCG

>Apis mellifera(XP-625016)

MSSRKTAGRRATTKKRAQRATSNVFAMFDQAQIAEFKEAFNMIDQNHDGFIDKEDLHDML

ASLGKNPTDEYLEAMMNEAPGPINFTMFLTLFGERLQGTDPEDVIKNAFGCFDEENTGHI

NEERLRELLTTMGDRFTDDDVDEMYREAPIKGSMFDYIEFTRILKHGAKKDEQ

>Drosophila melanogaster(Mylc)

MAAYTEDQLAEFQEAFNLFDNRGDGKIQLSQVGECLRALGQNPTESDVKKCTHQLKPDER

ISFEVFLPIYQAISKARSGDTADDFIEGLRHFDKDASGYISSAELRHLLTTLGEKLTDEE

VEQLLANMEDQQGNINYEEFVRMVMSG

>Drosophila melanogaster(Sqh)

MSSRKTAGRRATTKKRAQRATSNVFAMFDQAQIAEFKEAFNMIDQNRDGFVEKEDLHDML

ASLGKNPTDDYLDGMMNEAPGPINFTMFLTLFGERLQGTDPEDVIKNAFGCFDEENMGVL

PEDRLRELLTTMGDRFTDEDVDEMYREAPIKNGLFDYLEFTRILKHGAKDKDEQ

**Anion exchanger family SLC4A**

>Homo sapiens SLC4A2

MSSAPRRPAKGADSFCTPEPESLGPGTPGFPEQEEDELHRTLGVERFEEILQEAGSRGGE

EPGRSYGEEDFEYHRQSSHHIHHPLSTHLPPDARRRKTPQGPGRKPRRRPGASPTGETPT

IEEGEEDEDEASEAEGARALTQPSPVSTPSSVQFFLQEDDSADRKAERTSPSSPAPLPHQ

EATPRASKGAQAGTQVEEAEAEAVAVASGTAGGDDGGASGRPLPKAQPGHRSYNLQERRR

IGSMTGAEQALLPRVPTDEIEAQTLATADLDLMKSHRFEDVPGVRRHLVRKNAKGSTQSG

REGREPGPTPRARPRAPHKPHEVFVELNELLLDKNQEPQWRETARWIKFEEDVEEETERW

GKPHVASLSFRSLLELRRTLAHGAVLLDLDQQTLPGVAHQVVEQMVISDQIKAEDRANVL

RALLLKHSHPSDEKDFSFPRNISAGSLGSLLGHHHGQGAESDPHVTEPLMGGVPETRLEV

ERERELPPPAPPAGITRSKSKHELKLLEKIPENAEATVVLVGCVEFLSRPTMAFVRLREA

VELDAVLEVPVPVRFLFLLLGPSSANMDYHEIGRSISTLMSDKQFHEAAYLADEREDLLT

AINAFLDCSVVLPPSEVQGEELLRSVAHFQRQMLKKREEQGRLLPTGAGLEPKSAQDKAL

LQMVEAAGAAEDDPLRRTGRPFGGLIRDVRRRYPHYLSDFRDALDPQCLAAVIFIYFAAL

SPAITFGGLLGEKTQDLIGVSELIMSTALQGVVFCLLGAQPLLVIGFSGPLLVFEEAFFS

FCSSNHLEYLVGRVWIGFWLVFLALLMVALEGSFLVRFVSRFTQEIFAFLISLIFIYETF

YKLVKIFQEHPLHGCSASNSSEVDGGENMTWAGARPTLGPGNRSLAGQSGQGKPRGQPNT

ALLSLVLMAGTFFIAFFLRKFKNSRFFPGRIRRVIGDFGVPIAILIMVLVDYSIEDTYTQ

KLSVPSGFSVTAPEKRGWVINPLGEKSPFPVWMMVASLLPAILVFILIFMETQITTLIIS

KKERMLQKGSGFHLDLLLIVAMGGICALFGLPWLAAATVRSVTHANALTVMSKAVAPGDK

PKIQEVKEQRVTGLLVALLVGLSIVIGDLLRQIPLAVLFGIFLYMGVTSLNGIQFYERLH

LLLMPPKHHPDVTYVKKVRTLRMHLFTALQLLCLALLWAVMSTAASLAFPFILILTVPLR

MVVLTRIFTDREMKCLDANEAEPVFDEREGVDEYNEMPMPV

>Mus musculus SLC4A2

MSSAPRRPASGADSLHTPEPESLSPGTPGFPEQEEDELRTLGVERFEEILQEAGSRGGEE

PGRSYGEEDFEYHRQSSHHIHHPLSTHLPPDARRRKTPQGPGRKPRRRPGASPTGETPTI

EEGEEDEEEASEAEGFRAPPQQPSPATTPSAVQFFLQEDEGAERKPERTSPSPPTQTPHQ

EAAPRASKGAQTGTLVEEMVAVASGTAGGDDGGAAGRPLTKAQPGHRSYNLQERRRIGSM

TGVEQALLPRVPTDESEAQTLATADLDLMKSHRFEDVPGVRRHLVRKNAKGSTQAAREGR

EPGPTPRARPRAPHKPHEVFVELNELLLDKNQEPQWRETARWIKFEEDVEEETERWGKPH

VASLSFRSLLELRRTLAHGAVLLDLDQQTLPGVAHQVVEQMVISDQIKAEDRANVLRALL

LKHSHPSDEKEFSFPRNISAGSLGSLLGHHHAQGTESDPHVTEPLIGGVPETRLEVDRER

ELPPPAPPAGITRSKSKHELKLLEKIPENAEATVVLVGCVEFLSRPTMAFVRLREAVELD

AVLEVPVPVRFLFLLLGPSSANMDYHEIGRSISTLMSDKQFHEAAYLADERDDLLTAINA

FLDCSVVLPPSEVQGEELLRSVAHFQRQMLKKREEQGRLLPPGAGLEPKSAQDKALLQMV

EVAGAAEDDPLRRTGRPFGGLIRDVRRRYPHYLSDFRDALDPQCLAAVIFIYFAALSPAI

TFGGLLGEKTKDLIGVSELIMSTALQGVVFCLLGAQPLLVIGFSGPLLVFEEAFFSFCSS

NELEYLVGRVWIGFWLVFLALLMVALEGSFLVRFVSRFTQEIFAFLISLIFIYETFYKLI

KIFQEHPLHGCSGSNDSEAGSSSSSNMTWATTILVPDNSSASGQSGQEKPRGQPNTALLS

LVLMAGTFFIAFFLRKFKNSRFFPGRIRRVIGDFGVPIAILIMVLVDYSIEDTYTQKLSV

PSGFSVTAPDKRGWVINPLGEKTPFPVWMMVASLLPAVLVFILIFMETQITTLIISKKER

MLQKGSGFHLDLLLIVAMGGICALFGLPWLAAATVRSVTHANALTVMSKAVAPGDKPKIQ

EVKEQRVTGLLVALLVGLSMVIGDLLRQIPLAVLFGIFLYMGVTSLNGIQFYERLHLLLM

PPKHHPDVTYVKKVRTMRMHLFTALQLLCLALLWAVMSTAASLAFPFILILTVPLRMVVL

TRIFTEREMKCLDANEAEPVFDECEGVDEYNEMPMPV

>Rattus norvegicus SLC4A2

MSSAPRRPASGADSLHTPEPESLSPGTPGFPEQEEEDELRTLGVERFEEILQEAGSRGGE

EPGRSYGEEDFEYHRQSSHHIHHPLSTHLPPDARRRKTPQGPGRKPRRRPGASPTGETPT

IEEGEEDEDEVGEAEGFRAPPQQPSPASSPSAVQFFLQEDEGTDRKAERTSPSPPTQTPH

QEAAPRASKGAQTGTLVEEMVAVASGTAGGDDGGAAGRPLTKAQPGHRSYNLQERRRIGS

MTGVEQALLPRVPTDESEAQTLATADLDLMKSHRFEDVPGVRRHLVRKNAKGSTQAAREG

REPGPTPRARPRAPHKPHEVFVELNELQLDKNQEPQWRETARWIKFEEDVEEETERWGKP

HVASLSFRSLLELRRTLAHGAVLLDLDQQTLPGVAHQVVEQMVISDQIKAEDRANVLRAL

LLKHSHPSDEKEFSFPRNISAGSLGSLLGHHHAQGTESDPHVTEPLIGGVPETRLEVDRE

RELPPPAPPAGITRSKSKHELKLLEKIPENAEATVVLVGCVEFLSRPTMAFVRLREAVEL

DAVLEVPVPVRFLFLLLGPSSANMDYHEIGRSISTLMSDKQFHEAAYLADERDDLLTAIN

AFLDCSVVLPPSEVQGEELLRSVAHFQRQMLKKREEQGRLLPPGAGLEPKSAQDKALLQM

VEVAGAAEDDPLRRTGRPFGGLIRDVRRRYPHYLSDFRDALDPQCLAAVIFIYFAALSPA

ITFGGLLGEKTQDLIGVSELIMSTALQGVIFCLLGAQPLLVIGFSGPLLVFEEAFFSFCK

SNQLEYLVGRVWIGFWLVLLALLMVALEGSFLVRFVSRFTQEIFAFLISLIFIYETFYKL

IKIFQEHPLHGCSVSNDSEADSSSNNMTWAATTLAPDNSSASGQERPRGQPNTALLSLVL

MAGTFFIAFFLRKFKNSRFFPGRIRRVIGDFGVPIAILIMVLVDYSIEDTYTQKLSVPSG

FSVTAPDKRGWVINPLGEKTPFPVWMMVASLLPAVLVFILIFMETQITTLIISKKERMLQ

KGSGFHLDLLLIVAMGGICALFGLPWLAAATVRSVTHANALTVMSKAVAPGDKPKIQEVK

EQRVTGLLVALLVGLSMVIGDLLRQIPLAVLFGIFLYMGVTSLNGIQFYERLHLLLMPPK

HHPDVTYVKKVRTMRMHLFTALQLLCLALLWAVMSTAASLAFPFILILTVPLRMVVLTRI

FTEREMKCLDANEAEPVFDECEGVDEYNEMPMPV

>Canis familiaris SLC4A2

FVELNELLLDRNQEPQWRETARWIKFEEDVEEETERWGKPHVASLSFRSLLELRRTLAHG

AVLLDLDQQTLPGVAHQVVEQMVISDQIKAEDRANVLRALLLKHRRVALPRSDPSCHHWL

LGCCVEFLSRPTMAFVRLREAVELDAVLEVPVPVRFLFLLLGPSSANMDYHEIGRSISTL

MSDKQFHEAAYLADEREDLLTAINAFLDCSVVLPPSEVQGEELLRSVAHFQRQMLKKREE

QGRLLPPGAGLEPKSAQDKGAVEDDPLRRTGRPFGGLIRDVRRRYPHYLSDFRDALDPQC

LAAVIFIYFAALSPAITFGGLLGEKTQDLIGVSELIMSTALQGVVFCLLGAQPLLVIGFS

GPLLVFEEAFFSFCSSNNLEYLVGRVWIGFWLVLLALLMVALEGSFLVRFVSRFTQEIFA

FLISLIFIYETFYKLIKIFQEHPLHGCSVSNGSEAHSGDNDTWHGAGATLGPGNGSSPGP

VGQGRPRGQPNTALLSLVLMAGTFFIAFFLRKFKNSRFFPGRVRRVIGDFGVPIAILIMV

LVDYSIEDTYTQKLSVPSGFSVTAPEKRGWVINPLGENSSFPVWMMVASLLPAILVFILI

FMETQITTLIISKKERMLQKGSGFHLDLLLIVAMGGICALFGLPWLAAATVRSVTHANAL

TVMSKAVAPGDKPKIQEVKEQRVTGLLVALLVGLSIVIGDLLRQIPLAVLFGIFLYMGVT

SLNGIQFYERLHLLLMPPKHHPDVTYVKKVRTLRMHLFTALQLLCLALLWAVMSTAASLA

FPFILILTVPLRMVVLTRIFTEREMKCV

>Tetraodon nigroviridis SLC4A2

SLLHGTARRDEEEDGDLNKALGVHRFQQILSPASTVPDEQLHNYHEEDIEYHRHSSHNIH

RPLSKLPSEGRRKKSSKKRRKNKDLKSKLVALSSPVEEVEDEEEEEEEEVTDAPSSPSEA

ERPRDVEFFLSDDDCVVRRGGESVHSAREQDFASPSVEGDASSTDKSTSPEASSFTPQLV

PPEHPPLTRVSSRSYDLQERRRTGNMTGAEQAKYQQIPTDETEAQTLASADLDGIKSHRF

EDVPGVRRHLVRKSTKGQIVHIGKDHKEPTTRTRKQDRTPHEPLPVILYRSASQPEGVLL

DVGSGHMTQFREVFVELNELTMDKNQELQWKETARWIKFEEDVEEETDRWGKPHVASLSF

RSLLELRKTISHGAVLLDLDQKTLPGIAHQVVEQMIISDQIKAEDRANVLRALLLKHSHP

SDEKEHSSHFPRNISAASLGSLITHHHNANHAHQPEPSVTDPLMGSVHATRETDTHIDME

KNDVQKEPTAVSGMHRSKSKHELKLLEKIPENAEATVVLVGQWSPVILSGSTSNNYPFLT

CLCSAHRVPPPPGCVDFLEQPTMAFVRLQEAVELESVLEVPVPVRFLFVLLGPPTSSMDY

HQIGRSISTLMSDKRFHEAAYLADDRHDLLNAINSFLDCSIVLPPSEVGGDELLRSVARF

QREMLRKREELEVKLSAKEPKSLKDKEALLTPLKKSDDPLERTRRPFGGLIRDVRRRYPK

YLSDFKDALNSQCMAAVIFIYFAALSPAITFGGLLGGKQEFGYCRLLILQTVAQGVFFFF

LLGEKTDGLIGVSELIVSTAVQGVIFCLLGAQPLLIVGFSGPLLVFEEAFYSFCKTNNME

YLTGRVWIGFWLIIIVTLMVAFEGSFLVRFVSRFTQEIFSFLISLIFICETFIKLGRIFK

EHPLKRCSLDNSTGGNVTVENATSVPVNSTQPGAEVTLGEPNTALLSLVLMAGTFFIAFY

LRKFKNSAFFPGRLRRVIGDFGVPIAILIMVLVAYNLSDTFIQKLSVPKGFSVTSPSKRG

WLINPLGSDGKFPVWMMFACCLPALLVFILIFMETQITTLIVSKKERMLVKGSGFHLDLL

LIVVLGGTSAVFGLPWMAAATVRSVTHVNALTVMSKAVAPGDKPRIQEVKEQRVTGLLVS

ILVGLSIVIGDLLRQIPLAVLFGIFLYMGVMSLNGIQLTERMMLLLMPPKYHPDHTYVRK

VRTLRMHMFTCIQLVCLAVLWSVMSTQASLAFPFILILTVPVKTFLLPRIFTAREMASLD

ADDAEPKFDERECQDEYSEMHMPV

>Takifugu rubripes SLC4A2

SSLHGTARRDEEDDGDLNKALGVQRFQQILSPAAAVPDEQLHNYHEEDIEYHRHSSHHIH

RPLSKLPSEGRRKKGSKKRRKDKHHKSSHAAPSSPYTAYVRPVGNKTHFFSEQTFTPSSL

QFFLSDDDRVVKHGRGSGHSAREQDIASQSGEGDASSTDKSTSPEASSSAPQLVPAEHPP

LTRVSSRSYDLQERRRTGNMTGAEQAKYQRIPTDETEAQTLASADLDGIKSHRFEDVPGV

RRHLVRKSTKGQIVHIGKDHKEPTTRARKQDRTPHEPLPVILYRSASRPEGFLLDLGSSH

MAQYKEVFVELNELTMDKNQELQWKETARWIKFEEDVEEETDRWGKPHVASLSFRSLLEL

RKTISHGAVLLDLDQKTLPGIAHQVVEQMIISDQIKAEDRANVLRALLLKHSHPSDEKEH

NSHFPRNISAASLGSLVTHHHNANHAHQPEPSVTDPLMGGGHAVGETDIHIDVEKNDVQK

EPAVVSGMHRSKSKHELKLLEKIPENAEATVVLVGQLSLVITSGIISNNYFTACLCPPPG

CVDFLEQPTMAFVRLQEAVELESVLEVPVPVRFLFVLLGPPNSSMDYHQIGRSISTLMSD

KRFHEAAYLADDRHDLLNAINSFLDCSIVLPPSEVGGDELLRSVARFQREMLRKREELEV

KLLAKEPKSLKDKEALLTPLKKSDDPLERTRRPFGGLVRDVRRRYPKYLSDFKDALNSQC

MAAVIFIYFAALSPAITFGGLLGETHTWRTGVGLLQAVEGVSLFLGEKTDGLIGVSELIV

STAVQGVIFCLLGAQPLLIVGFSGPLLVFEEAFYSFCKANNMEYLTGRVWIGFWLIIIVT

LTVAFEGSFLVRFVSRFTQEIFSFLISLIFICETFIKLGRIFKEHPLKRCSVDNGTDGNV

TVENVTSLLNNDTQPAAVTTLGEPNTALLSLVLMAGTFFIAFYLRKFKNSAFFPGRLRRV

IGDFGVPIAILIMVLVAYNLSDTFIQKLSVPRGFSVTTPAKRGWLIHPLGSDGNFPIWMM

FACCLPALLVFILIFMETQITTLIVSKKERMLVKGSGFHLDLLLIVVLGGTSAVFGLPWM

AAATVRSVTHVNALTVMSKAVAPGDKPRIQEVKEQRVTGLLVSILVGLSIVIGDLLRQIP

LAVLFGIFLYMGVMSLNGIQLTERMMLLLMPPKYHPDHSYVRKVRTLRMHMFTCIQVVCL

AVLWAVMSTQASLAFPFVLILTVPVKMFLLPRIFTAREMACLDADDAEPKFDERECQDEY

SEMRMPV

>Danio rerio SLC4A2

HRHTSLHIHHPLSKHLPDGRRKKPGRKRKDSGRRRSSSMGAAPPIDEDDEDEEADEDSCS

QQDREGNVTTTPTRLQFFVSEDDPKKNAKTVNHVPFKTTHQHQHPRGCDGSLVSVHTKHF

QMHVLCVLSSVSSPDGSCKTGRSYDLQERRRTGNMTGATLSHYQQMPTDESEAKTLATVD

LDGIKSHRFEDVPGVRRHLVKKSAKGQVVHIGKDYKEFSSRIRTKLDRTPHEVFVELNEL

LMDKNQEMHWKETARWIKFEEDVEEETERWGKPHVASLSFRSLLELRKTISHGAVLLDLD

QKTLPGIAHQVVEQMIISDQIRAHDRANVLRALLLKHSHPSDGKEHSLFNRNISATSLGS

LISHYHSTNHIGAPELPATDPLIGGLRNFESRSSVDYVEKNEKDSPQFFGLHKTKSKHEL

KLLEKIPEDAEATVVLVGCVDFLDQPTMAFVRLKEAVLLESVLEVPIPVRFLFVLLGPPS

ANIDYHQIGRSISTLMSDKHFHEAAYLADGRQDLLTAINSFLDCSIVLPPSEVGGDELLH

SIARFQKEMLHKRHEQEVKLQAKEPKSPDDIVLMLSALQPPLKPEDDPLRRTGRLFGGVI

RDVRRRYPKYISDFKDALSPQCMATVIFIYFAALSPAVTFGGLLGEKTDGLIGVSELIIS

TAVQGMLFCLLGAQPLLIVGFSGPLLVFEEAFYSFCKSNDLEYLTGRMWIGMWLIIIVLL

TVAFEGSFLVRFVSRFTQEIFSILISLIFIYETFFKLGKIFMDHPLRSCSGPEENATSLS

TGSNDSRSTGASQTLNQPNTALLSLVLTSGTFFIAYYLRKFKNSAFFPGRLRRAIGDFGV

PIAISTMVLLDYSIKDTYTQKLNVPDGFSVTSPDKRGWFIHPLGSDGQFPIWMMGACILP

ALLVYILIFMETQITTLIVSKKERMLVKGSGFHLDLLIIVVSGGIAALFGLPWLTGATVR

SVTHANSLTVMSKAVAPGDKPRIQEVKEQRVTGFLVALLVGLSIVIGDLLRQVPIAVLFG

IFLYMGVMSLNGIQLTERMMLLFMPPKYHPDHTYVRKVRTLRMHLFTCLQLVCLAVLWIV

MSTAASLAFPFVLVLTVPFRRFLLSRIFSHREIQCLDADDAEPTLDDKDGQDEYTEMQMP

V

>Homo sapiens SLC4A1

MEELQDDYEDMMEENLEQEEYEDPDIPESQMEEPAAHDTEATATDYHTTSHPGTHKVYVE

LQELVMDEKNQELRWMEAARWVQLEENLGENGAWGRPHLSHLTFWSLLELRRVFTKGTVL

LDLQETSLAGVANQLLDRFIFEDQIRPQDREELLRALLLKHSHAGELEALGGVKPAVLTR

SGDPSQPLLPQHSSLETQLFCEQGDGGTEGHSPSGILEKIPPDSEATLVLVGRADFLEQP

VLGFVRLQEAAELEAVELPVPIRFLFVLLGPEAPHIDYTQLGRAAATLMSERVFRIDAYM

AQSRGELLHSLEGFLDCSLVLPPTDAPSEQALLSLVPVQRELLRRRYQSSPAKPDSSFYK

GLDLNGGPDDPLQQTGQLFGGLVRDIRRRYPYYLSDITDAFSPQVLAAVIFIYFAALSPA

ITFGGLLGEKTRNQMGVSELLISTAVQGILFALLGAQPLLVVGFSGPLLVFEEAFFSFCE

TNGLEYIVGRVWIGFWLILLVVLVVAFEGSFLVRFISRYTQEIFSFLISLIFIYETFSKL

IKIFQDHPLQKTYNYNVLMVPKPQGPLPNTALLSLVLMAGTFFFAMMLRKFKNSSYFPGK

LRRVIGDFGVPISILIMVLVDFFIQDTYTQKLSVPDGFKVSNSSARGWVIHPLGLRSEFP

IWMMFASALPALLVFILIFLESQITTLIVSKPERKMVKGSGFHLDLLLVVGMGGVAALFG

MPWLSATTVRSVTHANALTVMGKASTPGAAAQIQEVKEQRISGLLVAVLVGLSILMEPIL

SRIPLAVLFGIFLYMGVTSLSGIQLFDRILLLFKPPKYHPDVPYVKRVKTWRMHLFTGIQ

IICLAVLWVVKSTPASLALPFVLILTVPLRRVLLPLIFRNVELQCLDADDAKATFDEEEG

RDEYDEVAMPV

>Mus musculus SLC4A1

MGDMRDHEEVLEIPDRDSEEELENIIGQIAYRDLTIPVTEMQDPEALPTEQTATDYVPSS

TSTPHPSSGQVYVELQELMMDQRNQELQWVEAAHWIGLEENLREDGVWGRPHLSYLTFWS

LLELQKVFSKGTFLLGLAETSLAGVANHLLDCFIYEDQIRPQDREELLRALLLKRSHAED

LGNLEGVKPAVLTRSGGASEPLLPHQPSLETQLYCGQAEGGSEGPSTSGTLKIPPDSETT

LVLVGRANFLEKPVLGFVRLKEAVPLEDLVLPEPVGFLLVLLGPEAPHVDYTQLGRAAAT

LMTERVFRITASMAHNREELLRSLESFLDCSLVLPPTDAPSEKALLNLVPVQKELLRRRY

LPSPAKPDPNLYNTLDLNGGKGGPGDEDDPLRRTGRIFGGLIRDIRRRYPYYLSDITDAL

SPQVLAAVIFIYFAALSPAVTFGGLLGEKTRNLMGVSELLISTAVQGILFALLGAQPLLV

LGFSGPLLVFEEAFFSFCESNNLEYIVGRAWIGFWLILLVMLVVAFEGSFLVQYISRYTQ

EIFSFLISLIFIYETFSKLIKIFQDYPLQQTYAPVVMKPKPQGPVPNTALFSLVLMAGTF

LLAMTLRKFKNSTYFPGKLRRVIGDFGVPISILIMVLVDSFIKGTYTQKLSVPDGLKVSN

SSARGWVIHPLGLYRLFPTWMMFASVLPALLVFILIFLESQITTLIVSKPERKMIKGSGF

HLDLLLVVGMGGVAALFGMPWLSATTVRSVTHANALTVMGKASGPGAAAQIQEVKEQRIS

GLLVSVLVGLSILMEPILSRIPLAVLFGIFLYMGVTSLSGIQLFDRILLLFKPPKYHPDV

PFVKRVKTWRMHLFTGIQIICLAVLWVVKSTPASLALPFVLILTVPLRRLILPLIFRELE

LQCLDGDDAKVTFDEENGLDEYDEVPMPV

>Rattus norvegicus SLC4A1

MGDMQDHEKVLEIPDRDSEEELEHVIEQIAYRDLDIPVTEMQESEALPTEQTATDYIPTS

TSTSHPSSSQVYVELQELMMDQRNQELQWVEAAHWIGLEENLREDGVWGRPHLSYLTFWS

LLELQKVFSKGTFLLDLAETSLAGVANKLLDSFIYEDQIRPQDRDELLRALLLKRSHAED

LKDLEGVKPAVLTRSGAPSEPLLPHQPSLETKLYCAQAEGGSEEPSPSGILKIPPNSETT

LVLVGRASFLVKPVLGFVRLKEAVPLEDLVLPEPVSFLLVLLGPEAPHIDYTQLGRAAAT

LMTERVFRVTASLAQSRGELLSSLDSFLDCSLVLPPTEAPSEKALLNLVPVQKELLRKRY

LPRPAKPDPNLYEALDGGKEGPGDEDDPLRRTGRIFGGLIRDIRRRYPYYLSDITDALSP

QVLAAVIFIYFAALSPAVTFGGLLGEKTRNLMGVSELLISTAVQGILFALLGAQPLLVLG

FSGPLLVFEEAFYSFCESNNLEYIVGRAWIGFWLILLVVLVVAFEGSFLVQYISRYTQEI

FSFLISLIFIYETFSKLIKIFQDYPLQESYAPVVMKPKPQGPVPNTALLSLVLMVGTFLL

AMMLRKFKNSTYFPGKLRRVIGDFGVPISILIMVLVDTFIKNTYTQKLSVPDGLKVSNSS

ARGWVIHPLGLYNHFPKWMMFASVLPALLVFILIFLESQITTLIVSKPERKMIKGSGFHL

DLLLVVGMGGVAALFGMPWLSATTVRSVTHANALTVMGKASGPGAAAQIQEVKEQRISGL

LVSVLVGLSILMEPILSRIPLAVLFGIFLYMGITSLSGIQLFDRILLLFKPPKYHPDVPF

VKRVKTWRMHLFTGIQIICLAVLWVVKSTPASLALPFVLILTVPLRRLLLPLIFRELELQ

CLDGDDAKVTFDEAEGLDEYDEVPMPV

>Canis familiaris SLC4A1

DDHEEGLEDTLEQEEYEDPYVPMVQVEEPVAYPTEPTATDEDTVTHPDTTPRAGTHEIYV

ELHELVMDEKNQELQWMEAAHWVRLEENLGEDGVWGPPHLSYLTFWSLLELQKAFVKGTV

LLDLPETSLAGVANQLLDQFIYEEQIRPQDRDLLLRVLLLKHSHAGDLEALGGVKPAVLM

RSGDPSQPLLPQQPSLETQLFCKQGEGGTEDSPSRILEKIPPDSEATLVLVGRAAFLERP

VLGFVRLQMATELDAVGLPLPVRFLFVLLGPEAPNTDYTQLGRAVATLMSERVFRTDAYL

AQTKEELVRNLDCFLDCSLVLPPCEVPSEQALLSLVPVQKELLRRRYLLSPAKPDPHFYK

GLDLNGGPGGPGESDDPLQRTGLLFGGLVRDIRRRYPLYLSDITDAFSPQVLAAVIFIYF

AALSPAITFGGLLGEKTQNQMGVSELLISTAVQGILFSLLGAQPLLVVGFSGPLLVFEEA

FFSFCTSNNLEYIVGRVWIGFWLVLLVVLMVAFEGSFLVRFISRYTQEIFSFLISLIFIY

ETFIKLIKIFQDHPLQRYYDYNMTIIPKPQGPLPNTALLSLVLMAGTFFFAMMLRKFKNS

SYFPGKLRRVIGDFGVPISILIMVMVDFFIKDTYTQKLSVPRGLSVSNASARGWVIHPLG

LYSPFPIWMMFASVLPALLVFILIFLESQITTLIISKPERKMVKGSGFHLDLLLVIGMGG

VAALFGMPWLSATTVRSVTHANALTVMGKANIPGAAPQIQEVKEQRISGLLVAVLVGVSI

LMGPILSLIPLAVLFGIFLYMGVTSLSGIQLFDRVLLLFKPPKYHPDVPYVKRVKTWRMH

LFTVIQIICLAVLWTMKTFPTTSLTLPFILILTVPLRRLLLPLIFRKLELQCLDADDAKP

TFNEEEGQDEYNEVHMPV

>Tetraodon nigroviridis SLC4A1

GALDFLEKPAVVFVRLKESAVLGSALEAPMPVRFVFVLVGPSSADMDYHETGRAMAALLA

DKVFNQCALQAKTARELTDAVADFMDCSIVIPPTEIQNEAMLTSIINFQKKLLQDRNQAS

NPLARRDSKARRVSISTGPPPEDPLSRTGRPFGGMIRDIRRRYQHYRSDITDALNAQVLA

AVIFIYFAALSPAITFGGLLADKVENMMGVPELLISTSIQGIIFCFVAAQPVLVIGFSGP

LLVFEEAFYAFCKSQEIEYIVGRVWVGVWLVIIVVAIVAFEGSFLVRFISRFTQEIFSIL

ISLIFIYETFAKLGRVRRLIGDFGVPISMFLMIVLDYNIADTYTQKLVVPKGLMVSNPAK

RGWLINPFGEHEPFPVWLMFASCVPALLVFILIFLESQITTLIVSKPERKMVKGSGFHFD

LLILVGMGGLSAIFGVPWLSAATVRSVTHANALTVMSKGPKPAIEKVMEQRVSGILVALL

VGLSILMEPILKMIPVSALFGIFLYMGVTSLNGIQLWDRMLLLLIPKKYHPDEPYVTKVS

TGRMHLFTAIQIVCLALLWIVKSSPDLSCSSLCAHPHHPSAHAHDRAPVHRSGNEMCKCF

PRLSRSRNVCRGRSKL

>Homo sapiens SLC4A10

MEPLLPTRNDEEAVVDRGGTRSILKTHFEKEDLEGHRTLFIGVHVPLGGRKSHRRHRHRG

HKHRKRDRERDSGLEDGRESPSFDTPSQRVQFILGTEDDDEEHIPHDLFTELDEICWREG

EDAEWRETARWLKFEEDVEDGGERWSKPYVATLSLHSLFELRSCILNGTVLLDMHANTLE

EIADMVLDQQVSSGQLNEDVRHRVHEALMKQHHHQNQKKLTNRIPIVRSFADIGKKQSEP

NSMDKNAGQVVSPQSAPACVENKNDVSRENSTVDFSKVDLHFMKKIPPGAEASNILVGEL

EFLDRTVVAFVRLSPAVLLQGLAEVPIPTRFLFILLGPLGKGQQYHEIGRSIATLMTDEV

FHDVAYKAKDRNDLVSGIDEFLDQVTVLPPGEWDPSIRIEPPKNVPSQEKRKIPAVPNGT

AAHGEAEPHGGHSGPELQRTGRIFGGLILDIKRKAPYFWSDFRDAFSLQCLASFLFLYCA

CMSPVITFGGLLGEATEGRISAIESLFGASMTGIAYSLFGGQPLTILGSTGPVLVFEKIL

FKFCKEYGLSYLSLRASIGLWTATLCIILVATDASSLVCYITRFTEEAFASLICIIFIYE

ALEKLFELSEAYPINMHNDLELLTQYSCNCVEPHNPSNGTLKEWRESNISASDIIWENLT

VSECKSLHGEYVGRACGHDHPYVPDVLFWSVILFFSTVTLSATLKQFKTSRYFPTKVRSI

VSDFAVFLTILCMVLIDYAIGIPSPKLQVPSVFKPTRDDRGWFVTPLGPNPWWTVIAAII

PALLCTILIFMDQQITAVIINRKEHKLKKGCGYHLDLLMVAVMLGVCSIMGLPWFVAATV

LSITHVNSLKLESECSAPGEQPKFLGIREQRVTGLMIFILMGSSVFMTSILKFIPMPVLY

GVFLYMGASSLKGIQFFDRIKLFWMPAKHQPDFIYLRHVPLRKVHLFTIIQMSCLGLLWI

IKVSRAAIVFPMMVLALVFVRKLMDLLFTKRELSWLDDLMPESKKKKLEDAEKEEEQSML

AMEDEGTVQLPLEGHYRDDPSVINISDEMSKTALWRNLLITADNSKDKESSFPSKRTPS

>Mus musculus SLC4A10

RNDEEAVVDRGGTRSILKTHFEKEDLEGHRTLFIGVHVPLGGRKSHRRHRHRGHKHRKRD

RERDSGLEDGRESPSFDTPSQRVQFILGTEDDDEEHLPHDLFTELDEICWREGEDAEWRE

TARWLKFEEDVEDGGERWSKPYVATLSLHSLFELRSCILNGTVLLDMHANTIEEIADMVL

DQQVSSGQLNEDVRHRVHEALMKQHHHQNQKKLANRIPIVRSFADIGKKQSEPNSMDKNA

GQVVSPQSAPACAENKNDVSRENSTVDFSKVDLHFMKKIPPGAEASNILVGELEFLDRTV

VAFVRLSPAVLLQGLAEVPIPSRFLFILLGPLGKGQQYHEIGRSIATLMTDEVFHDVAYK

AKDRNDLVSGIDEFLDQVTVLPPGEWDPSIRIEPPKNVPSQEKRKIPAVPNGTAAHGEAE

PHGGHSGPELQRTGRIFGGLILDIKRKAPFFWSDFRDAFSLQCLASFLFLYCACMSPVIT

FGGLLGEATEGRISAIESLFGASMTGIAYSLFGGQPLTILGSTGPVLVFEKILFKFCKEY

GLSYLSLRASIGLWTATLCIILVATDASSLVCYITRFTEEAFASLICIIFIYEALEKLFE

LSETYPINMHNDLELLTQYSCNCMEPHSPSNDTLKEWRESNLSASDIIWGNLTVSECRSL

HGEYVGRACGHGHPYVPDVLFWSVILFFSTVTMSATLKQFKTSRYFPTKVRSIVSDFAVF

LTILCMVLIDYAIGIPSPKLQVPSVFKPTRDDRGWFVTPLGPNPWWTIIAAIIPALLCTI

LIFMDQQITAVIINRKEHKLKKGCGYHLDLLMVAVMLGVCSIMGLPWFVAATVLSITHVN

SLKLESECSAPGEQPKFLGIREQRVTGLMIFILMGSSVFMTSILKFIPMPVLYGVFLYMG

ASSLKGIQLFDRIKLFWMPAKHQPDFIYLRHVPLRKVHLFTVIQMSCLGLLWIIKVSRAA

IVFPMMVLALVFVRKLMDFLFTKRELSWLDDLMPESKKKKLEDAEKEEEQSMLAMEDEGT

VQLPLEGHYRDDPSVINISDEMSKTAMWGNLLVTADNSKEKESRFPSKSSPS

>Rattus norvegicus SLC4A10

RNDEEAVVDRGGTRSILKTHFEKEDLEGHRTLFIGVHVPLGGRKSHRRHRHRGHKHRKRD

RERDSGLEDGGESPSFDTPSQRVQFILGTEDDDEEHIPHDLFTELDEICWREGEDAEWRE

TARWLKFEEDVEDGGERWSKPYVATLSLHSLFELRSCILNGTVLLDMHANTLEEIADMVL

DQQVSSGQLNEDVRHRVHEALMKQHHHQSQKKLTNRIPIVRSFADIGKKQSEPNSMDKNG

QVVSPQSAPACAENKNDVSRENSTVDFSKGLGGQQKGHTSPCGMKQRLDKGPPHQQEREV

DLHFMKKIPPGAEASNILVGELEFLDRTVVAFVRLSPAVLLQGLAEVPIPSRFLFILLGP

LGKGQQYHEIGRSIATLMTDEVFHDVAYKAKDRNDLVSGIDEFLDQVTVLPPGEWDPSIR

IEPPKNVPSQEKRKTPSLPNGTAAHGGPEQHGGHSGPELQRTGRIFGGLILDIKRKAPFF

WSDFRDAFSLQCLASFLFLYCACMSPVITFGGLLGEATEGRISAIESLFGASMTGIAYSL

FGGQPLTILGSTGPVLVFEKILFKFCKEYGLSYLSLRASIGLWTATLCIILVATDASSLV

CYITRFTEEAFASLICIIFIYEALEKLFELSESYPINMHNDLELLTQYSCNCMEPHSPSN

DTLKEWRESNISASDIIWGNLTVSECRSLHGEYVGRACGHGHPYVPDVLFWSVILFFSTV

TMSATLKQFKTSRYFPTKVRSIVSDFAVFLTILCMVLIDYAIGIPSPKLQVPSVFKPTRD

DRGWFVTPLGPNPWWTIIAAIIPALLCTILIFMDQQITAVIINRKEHKLKKGCGYHLDLL

MVAVMLGVCSIMGLPWFVAATVLSITHVNSLKLESECSAPGEQPKFLGIREQRVTGLMIF

ILMGSSVFMTSILKFIPMPVLYGVFLYMGASSLKGIQLFDRIKLFWMPAKHQPDFIYLRH

VPLRKVHLFTVIQMSCLGLLWIIKVSRAAIVFPMMVLALVFVRKLMDFLFTKRELSWLDD

LMPESKKKKLEDAEKEEEQSMLAMEDEGTVQLPLEGHYRDDPSVINISDEMSKTAMWGNL

LVTADNSKEKESRFPSKRSSPG

>Canis familiaris SLC4A10

QCRTWPAARVSRSHVTLVSWSVQPPPASEVPDPNTKQSECRAGCRTLKTLQRRVLIPEAL

QDMEIKDQGAQMEPLLPTRNDEEAVVDRGGTRSILKTHFEKEDLEGHRTLFIGVHVPLGG

RKSHRRHRHRGHKHRKRDRERDSGLEDGRESPSFDTPSQRVQFILGTEDDDEEHIPHDLF

TELDEICWRESEDAEWRETARWLKFEEDVEDGGERWSKPYVATLSLHSLFELRSCILNGT

VLLDMHANTLEEIADMVLDQQVSSGQLNEDVRHRVHEALMKQHHHQNQKKLTNRIPIVRS

FADIGKKQSEPNSMDKNGQVVSPQSAPACVENKNDVSRENSTVDFSKVDLHFMKKIPPGA

EASNILVGELEFLDRTVVAFVRLSPAVLLQGLAEVPIPTRFLFILLGPLGKGQQYHEIGR

SIATLMTDEVFHDVAYKAKDRNDLVSGIDEFLDQVTVLPPGEWDPSIRIEPPKNVPSQEK

RKIPAVPNGTAAHGEAEPHGGHSGPELQRTGRIFGGLILDIKRKAPYFWSDFRDAFSLQC

LASFLFLYCACMSPVITFGGLLGEATEGRISAIESLFGASMTGIAYSLFGGQPLTILGST

GPVLVFEKILFKFCKEYGLSYLSLRASIGLWTATLCIILVATDASSLVCYITRFTEEAFA

SLICIIFIYEALEKLFELSEAYPINMHNDLELLTQYSCNCVEPHNPSNDTLKEWKESNIS

ASDIIWENLTVSVRSIVSDFAVFLTILCMVLIDYAIGIPSPKLQVPSVFKPTRDDRGWFV

TPLGPNPWWTVIAAIIPALLCTILIFMDQQITAVIINRKEHKLKKGCGYHLDLLMVAVML

GVCSIMGLPWFVAATVLSITHVNSLKLESECSAPGEQPKFLGIREQRVTGLMIFILMGSS

VFMTSILKFIPMPVLYGVFLYMGASSLKGIQFFDRIKLFWMPAKHQPDFIYLRHVPLRKV

HLFTVIQMSCLGLLWIIKVSRAAIVFPMMVLALVFVRKLMDFLFTKRELSWLDDLMPESK

KKKLEDAEKEEEQSMLAMEDEGTVQLPLEGHYRDDPSVINISDEMSKTALWRNLLMTADN

SKDKESSFPSKSSPS

>Homo sapiens SLC4A8

VLPREELQATATSRERRQEGPAWPSPFILLSGVVRRKRKRRRLAEEEDNYIESVVKFRLE

HNKCSMFNKNNSNKLRSTPRYRRGDPGYLNFTELGPLKPEQKDQWSQHRPDEEAVVDQGG

TSTILNIHYEKEELEGHRTLYVGVRMPLGRQSHRHHRTHGQKHRRRGRGKGASQGEEGLE

ALAHDTPSQRVQFILGTEEDEEHVPHELFTELDEICMKEGEDAEWKETARWLKFEEDVED

GGERWSKPYVATLSLHSLFELRSCLINGTVLLDMHANSIEEISDLILDQQELSSDLNDSM

RVKVREALLKKHHHQNEKKRNNLIPIVRSFAEVGKKQSDPHLMDKHGQTVSPQSVPTTNL

EVKNGVNCEHSPVDLSKVDLHFMKKIPTGAEASNVLVGEVDILDRPIVAFVRLSPAVLLS

GLTEVPIPTRFLFILLGPVGKGQQYHEIGRSMATIMTDEIFHDVAYKAKERDDLLAGIDE

FLDQVTVLPPGEWDPSIRIEPPKNVPSQEKRKMPGVPNGNVCHIEQEPHGGHSGPELQRT

GRLFGGLVLDIKRKAPWYWSDYRDALSLQCLASFLFLYCACMSPVITFGGLLGEATEGRI

SAIESLFGASMTGIAYSLFAGQALTILGSTGPVLVFEKILFKFCKDYALSYLSLRACIGL

WTAFLCIVLVATDASSLVCYITRFTEEAFASLICIIFIYEAIEKLIHLAETYPIHMHSQL

DHLSLYYCRCTLPENPNNHTLQYWKDHNIVTAEVHWANLTVSECQEMHGEFMGSACGHHG

PYTPDVLFWSCILFFTTFILSSTLKTFKTSRYFPTRVRSMVSDFAVFLTIFTMVIIDFLI

GVPSPKLQVPSVFKPTRDDRGWIINPIGPNPWWTVIAAIIPALLCTILIFMDQQITAVII

NRKEHKLKKGCGYHLDLLMVAIMLGVCSIMGLPWFVAATVLSITHVNSLKLESECSAPGE

QPKFLGIREQRVTGLMIFVLMGCSVFMTAILKFIPMPVLYGVFLYMGVSSLQGIQFFDRL

KLFGMPAKHQPDFIYLRHVPLRKVHLFTLIQLTCLVLLWVIKASPAAIVFPMMVLALVFV

RKVMDLCFSKRELSWLDDLMPESKKKKLDDAKKKAKEEEVIVLAPTVYLGASNYRT

>Mus musculus SLC4A8

RLRPAMPAGSNEPDGVLSYQRPDEEAVVDQGGTSTILNIHYEKEELEGHRTLYVGVRMPL

GRQSHRHHRTHGQKHRRRGGRGKGASQGEEGLEALAHDTPSQRVQFILGTEEDEEHVPHE

LFTELDEICMKEGEDAEWKETARWLKFEEDVEDGGERWSKPYVATLSLHSLFELRSCLIN

GSVLLDMRASSIEEISDLILDQQELLRDLSDSVRVKVREALLKKHHHQNERRRNNLIPIV

RSFAEVGKKQSDPHSMDRDGQTVSPQSATNLEVKNGVNCEHSPVDLSKVDLHFMKKIPTG

AEASNVLVGEVDTLDRPIVAFVRLSPAVLLSGLTEVPIPTRFLFILLGPVGKGQQYHEIG

RSMATIMTDEIFHDVAYKAKERDDLLAGIDEFLDQVTVLPPGEWDPSIRIEPPKNVPSQE

KRKMPGVPNGNVCHIEPEPHGGHSGPELERTGRLFGGLVLDVKRKAPWYWSDYRDALSLQ

CLASFLFLYCACMSPVITFGGLLGEATEGRISAIESLFGASMTGIAYSLFAGQPLTILGS

TGPVLVFEKILFKFCKDYALSYLSLRALIGLWTAFLCIVLVATDASSLVCYITRFTEEAF

ASLICIIFIYEAIEKLIHLAETYPIHMHSQLDHLSLYYCRCVLPENPNNHTLQYWKDHNI

LAAEVNWANLTVSECQEMHGEFMGSACGHHGPYTPDVLFWSCILFFATFIVSSTLKTFKT

SRYFPTRVRSMVSDFAVFLTIFTMVVLDFLIGVPSPKLQVPNVFKPTRDDRGWFINPIGP

NPWWTVIAAIIPALLCTILIFMDQQITAVIINRKEHKLKKGCGYHLDLLMVAVMLGVCSI

MGLPWFVAATVLSITHVNSLKLESECSAPGEQPKFLGIREQRVTGLMIFVLMGCSVFMTA

VLKFIPMPVLYGVFLYMGVSSLQGIQFFDRLKLFGMPAKHQPDFIYLRHVPLRKVHLFTL

VQLTCLVLLWVIKASPAAIVFPMMVLALVFVRKVMDLCFSKRELSWLDDLMPESKKKKLD

DAKKKEEEEAEKMLDIGGDKFPLESRKLLSSPGKSSSFRCDPSEINISDEMPKTTVWKAL

SINSGNTKEKSPFC

>Rattus norvegicus SLC4A8

MPAGSNEPDGVLSYQRPDEEAVVDQGGTSTILNIHYEKEELEGHRTLYVGVRMPLGRQSH

RHHRTHGQKHRRRGGRGKGASQGEEGLEASAHDTPSQRVQFILGTEEDEEHVPHELFTEL

DEICMKEGEEAEWKETARWLKFEEDVEDGGERWSKPYVATLSLHSLFELRSCLINGSVLL

DMRASSIEEISDLILDQQELLRDLSDSVRVKVREALLKKHHHQNEKKRNNLIPIVRSFAE

VGKKQSDPHSMDRDGQTVSPQSAPATNLEVKNGVNCEHSPVDLSKADLHFMKKIPAGAEA

SNVLVGEVDTLDRPIVAFVRLSPAVLLSGLTEVPIPTRFLFILLGPVGKGQQYHEIGRSM

ATIMTDEIFHDVAYKAKERDDLLAGIDEFLDQVTVLPPGEWDPSIRIEPPKNVPSQEKRK

MPGVPNGNICHVEPEPHGGHSGPELERTGRLQCLASFLFLYCACMSPVITFGGLLGEATE

GRISAIESLFGASMTGIAYSLFAGQPLTILGSTGPVLVFEKILFKFCKDYALSYLSLRAC

IGLWTAFLCIVLVATDASSLVCYITRFTEEAFASLICIIFIYEAIEKLIHLAETYPIHMH

SQLDHLSLYYCRCALPENPNNHTLQYWKEHSIPTADVNWANLTVSECQEMHGEFIGSACG

HHGPYTPDVLFWSCILFFATFIVSSTLKTFKTSRYFPTRVRSTVSDFAVFLTIFTMVILD

FLIGVPSPKLQVPSVFKPTRDDRGWFISPIGPNPWWTVIAAIIPALLCTILIFMDQQITA

VIINRKEHKLKKGCGYHLDLLVVAIMLGVCSLMGLPWFVAATVLSITHVNSLKLESECSA

PGEQPKFLGIREQRVTGLMIFVLMGCSVFMTAVLKFIPMPVLYGVFLYMGVSSLQGIQFF

DRLKLFGMPAKHQPDFIYLRHVPLRKVHLFTLVQLTCLVLLWVIKASPAAIVFPMMVLAL

VFVRKVMDLCFSKRELSWLDDLMPESKKKKLDDAKKKEEEEEAEKMLDIGGDKFPLESRK

LLSSPGKNNSFRCDPSEINISDEMPKTTVWKALSINSGNTKEKSPFN

>Canis familiaris SLC4A8

QQRPDEEAVVDQGGTSTILNIHYEKEELEGHRTLYVGVRMPLGRQSHRHHRTHGQKHRRR

GRGKGASQGEEGPEALAHDTPSQRVQFILGTEEDEEHVPHELFTELDEICMKEGEDAEWK

ETARWLKFEEDVEDGGERWSKPYVATLSLHSLFELRSCLINGTVLLDMRANSIEEISDLI

LDQQELFSDLNDSMRVKVREALLKKHHHQNEKKRNNLIPIVRSFAEVGKKQSDPHSMDKN

GQTVSPQSVPTTSLEVKNGVNYEHSPVDLSKVDLHFMKKIPTGAEASNVLVGEVDSLDRP

IVAFVRLSPAVLLSGLTEVPIPTRFLFILLGPVGKGQQYHEIGRSMATIMTDEIFHDVAY

KAKERDDLLAGIDEFLDQVTVLPPGEWDPSIRIEPPKNVPSQEKRKMPGVPNGNVCHIEP

EPHGGHSGPELQRTGRLFGGLVLDIKRKAPWYWSDYRDALSLQCLASFLFLYCACMSPVI

TFGGLLGEATEGRISAIESLFGASMTGIAYSLFAGQALTILGSTGPVLVFEKILFKFCKD

YALSYLSLRACIGLWTAFLCIVLVATDASSLVCYITRFTEEAFASLICIIFIYEAIEKLI

HLAETYPIHMHSQLDHLSLYYCRCTVPENPSNHTLQYWKDHHIVTTEVQWANLTVSECQE

LHGEFTGSACGHHGPYTPDVLFWSCILFFTTFILSSTLKTFKTSRYFPTRVRSMVSDFAV

FLTIFTMVIIDFLIGVPSPKLQVPSVFKPTRDDRGWIISPIGPNPWWTVIAAIIPALLCT

ILIFMDQQITAVIINRKEHKLKKGCGYHLDLLMVAIMLGVCSIMGLPWFVAATVLSITHV

NSLKLESECSAPGEQPKFLGIREQRVTGLMIFVLMGCSVFMTAILKFIPMPVLYGVFLYM

GVSSLQGIQFFDRLKLFGMPAKHQPDFIYLRHVPLRKVHLFTLIQLTCLVLLWVIKASPA

AIVFPMMVLALVFVRKVMDLCFSKRELSWLDDLMPESKKKKLDDAKKKAKEEEVIVLAPT

VHSGAASSYRT

>Tetraodon nigroviridis SLC4A8

RPDEEVVVDQGGTSSVLNIHYEKEELEGHRTLFVGVRMPRQSHRHHKTHSSRHRKREKRT

GSIVTQQSEESVTISSIHDTPSQRVQFILGTEDDAEHVAHELFTELDEICVKDGKDAEWK

ETARWLKFEEDVEDGGERWSKPYVATLSLHSLFELRSCIINGSVLLDMRATCIEEIADMV

LDHQEASSELDDSVRVKVREALLKRHHHQNEKKKNLIPIVRSITEGTRKQSEPHLMGTAT

SPQPQPAADLAKNGGGQDSTQVDLSKVDLHFMKKIPEGAEASNVLVGELDFLERPIVAFV

RLSPAVLLTGLTEVPIPTRFLFILLGPDGKAQQYHEIGRSMATIMTDEIFHDVAYKAKDR

SDLLAGIDEFLDQVTVLPPGDSLIFVCNVQEKRKMPGVPNGTVCPVEEELHAEHHGPELQ

RTGKLFGGLIQDIKRKAPFYLSDFKDGVSLQCVASFLFLYCACMSPVITFGGLLGEATEG

RISAIESLFGASMTGVAYSLFAGQPLTILGSTGPVLVFEKILFKFCKDYDLSYLSLRACI

GLWTALLCLILVATDASSLVCYITRFTEEAFAALICLIFIYEALEKLLHLGEVYPFNAHS

DLDQLTLAYCRCSEPDSPSNKTLELWRDRNVTASAVTWSNLTVKECISLHGQFVGTACGH

HGPYTPDVLFWSTILFFSTFFMSTFLKQFKTSRYFPTKVRSTISDFAVFLTIVIMVLLDF

IIGVPSQKLKVPSKFQPTRDDRGWLINPIGRNPWWTVLAASIPALLCTILIFMDQQITAV

IINRKEHKLLKGCGYHLDLLMVGVMLAVCSIMGLPWFVAATVLSISHVNSLKLESESSAP

GEQPRFLGIREQRLTGLVIFLLMGCSVFMTGALQFIPMPVLYGVFLYMGVSSLKGIQFFD

RLKLFGMPPKHQPDFIYLRHVPLRKVHLFTVTQLTCLVLLWIIKTSPAAIVFPMMANLFS

FYVLQVLALVFVRKVLDLCFSNRELSYLDDLMPEWKKKNLDDASKKIEEVMLSEQRENTI

EIPMVSSEPAHVQKTHDPRIEEITYIDE

>Takifugu rubripes SLC4A8

RPDEEVVVDQGGTSSAMNIHYEKEELEGHRTLFVGVRMPRQSHRHHKPHGSRHRKREKRT

GSIVTQQSEESVTITSSHDTPSQRVQFILGTEDDAEHVAHELFTELDEICVKDGKDAEWK

ETARWLKFEEDVEDGGERWSKPYVATLSLHSLFELRSCIINGSVLLDMRASCIEEIADMV

LDHQEASSELDDSVRVKVREALLKRHHHQNEKKKNLIPIVRSIAEGTRKQSEPHLMGGNN

RTATSPQPQPVADPAKNGGGQDSTQVDLSKVDLHFMKKIPEGAEASNVLVGELDFLERPI

VAFVRLSPAVLLTGLTEVPIPTRFLFILLGPDGKAQQYHEIGRSMATIMTDEIFHDVAYK

AKDRSDLLAGIDEFLDQVTVLPPGEWDPSIRIEPPKSVPSQAGKRKMPGVPNGTACPVEE

ELHAEHHGPELQRTGKLFGGLIQDIKRKAPFYLSDFKDGMSLQCVASFLFLYCACMSPVI

TFGGLLGEATEGRISAIESLFGASMTGVAYSLFAGQPLTILGSTGPVLVFEKILFKFCKD

YDLSYLSLRACIGLWTALLCLFLVATDASSLVCYITRFTEEAFAALICLIFIYEALEKLC

HLGEVYPFNTHSDLDHLTLAYCRCAEPDIPTNKTLELWRERNVTASVVPWSNLTVKECIG

LHGQFVGTACGHHGPYTPDVLFWSTILFFSTFFMSTFLKQFKTSRYFPTKVRSTISDFAV

FLTIVVMVLLDFVIGVPSQKLKVPSKFQPTRDDRGWLINPIGRNPWWTVLAASIPALLCT

ILIFMDQQITAVIINRKEHKLLKGCGYHLDLLMVGVMLAVCSIMGLPWFVAATVLSITHV

NSLKLESESSAPGEQPRFLGIREQRLTGLVIFLLMGCSVFMTGALQFIPMPVLYGVFLYM

GVSSLKGIQFFDRLKLFGMPPKHQPDFIYLRHVPLRKVHLFTVTQLTCLVLLWVIKTSPA

AIVFPMMASLQTHFPRSPSKYEQYQQFFFLCLQVLALVFVRKVLDLCFSNRELSYLDDLM

PEWKKKNLDDASKKIEEVMLSEKRENTVEIPMESSKPTHVQKTHDPRSDPSDINISDEMS

KTTVWKSLNSNQKDSRPATAKKARP

>Danio rerio SLC4A8

MPAENSDQESMLSYQCTCSSSGIPIARDNSSHHAFIIRVIDCVPAGARTVSVPRYPQMTL

TPSSPSLDERPGEEAVVDHGGTSSVLNIHYEKEELEGHRTLFVGVRMPMDRPSHRHHRPH

SSRHRRRSDRMRAGGSAQDDDNSESTPGAHDTPSQRVRFILGTEEDEEHVAHELFTELDE

ICVKEGKDAEWKETARWLKFEEDVEDGGERWSKPYVATLSLHSLFELRSCIINGTVLLDM

KAGSIEEIADLVLEKQETPHELDDSVRMKVREALLKRHHHQNDKKRNNLLPMVKTLTEAN

RRHSETHFMDKTGLATSPQPPTTSTDIKNGATSEIGQMDLSKVDTHFMKKIPTGAEASNV

LVGELDFLERSIVAFVRLSPAVLLTGLTEVPIPTRFLFILLGPDGKAQQYHEIGRSMATI

MTDEIFHDVAYKAKDRTDLLAGIDEFLDQVTVLPPGEWDPSIRIEPPKCVPSQEKRKMPG

VPNGTAVTVEEELHAEHHGPELQRTGRLFGGLILDLKRKAPYYLSDFKDGLTLQCVASFL

FLYCACMSPVITFGGLLGEATEGQISAIESLLGASMTGVAYSLFAGQPLTILGSTGPVLV

FEKILFKFCKDYDLSYLSLRTCIGLWTAFLCLLLVATDASSLVCYITRFTEEAFAALICL

IFIYEALEKLFYLGELYPINRHSDLDKLTLAYCRCAEPDNPSNKTLEVWNQRNITASAVP

WTNLTVTECLDLQGQFIGPSCGHHGPYTPDVLFWSSILFFGTFLMSSFLKQFKASRYFPT

RVRSMISDFAVFLTIVFMVLIDYIIGVPSQKLQVPSKFKPTRDDRGWVMNPIGRNPWWTV

LAALIPALLCTILIFMDQQITAVIINRKEHKLVKGCGYHLDLLMVGVMLAVCSMLGLPWF

VAATVLSISHVNSLKLESESSAPGEQPRFLGIQEQRLTGLFIFLLMGCSVFMTGLLQFIP

MPVLYGVFLYMGVSSLKGIQLFDRLKLFGMPAKHQPDFIYLRHVPLRKVHLFTLTQLTCL

VLLWVIKHSPAAIVFPMMVLALVFIRKLLDFCFSKRELSYLDDLMPENRKKKLDDDSKKA

EEESQEMLVDESGESDNKDTVQIPMESKPLHSPRTPDPRTDPSDINISDEMSKTTVWKSL

NSNNMDTQRASTHKKHPLIYAVPHKSGLRNVSAFSGESVLLPCSCLDPQHRPNSVSWETN

SRRASKDALYTDISSVFELYRHKITLFNQTSPGNFSLLLSDLTEEDQGSVRINDRESSEV

SHDGHISKEELKNALQDDTVAQKLIEDFKKYGRDYELKLRDYLTRSIPNYPTPVEFHTSE

VSHATHMDALDRILDSEGFKADGEDLKFSWWSLKIDEECIKAAEERYLEKVFPNRSQEQR

ERQEAFLSKFTTSPAFHIHESRYGNFRFVFSLNKMMEAYKEQFCGGRDPVLREYKTVFYK

QEIMYAVLVHSPDDDEKFQNYSTIEESRFIDYRNNQIVWRAQAIGDSIQFNLILDEENKT

ANAERAFGENYYVWDHVCLAFHFRDLLKFPKDALLQNCIPCKKKKSRNKNCKGKMVLI

>Homo sapiens SLC4A5

MKVKEEKAGVGKLDHTNHRRRFPDQKECPPIHIGLPVPTYPQRKTDQKGHLSGLQKVHWG

LRPDQPQQELTGPGSGASSQDSSMDLISRTRSPAAEQLQDILGEEDEAPNPTLFTEMDTL

QHDGDQMEWKESARWIKFEEKVEEGGERWSKPHVSTLSLHSLFELRTCLQTGTVLLDLDS

GSLPQIIDDVIEKQIEDGLLRPELRERVSYVLLRRHRHQTKKPIHRSLADIGKSVSTTNR

SPARSPGAGPSLHHSTEDLRMRQSANYGRLCHAQSRSMNDISLTPNTDQRKNKFMKKIPK

DSEASNVLVGEVDFLDQPFIAFVRLIQSAMLGGVTEVPVPTRFLFILLGPSGRAKSYNEI

GRAIATLMVDDLFSDVAYKARNREDLIAGIDEFLDEVIVLPPGEWDPNIRIEPPKKVPSA

DKRKSVFSLAELGQMNGSVGGGGGAPGGGNGGGGGGGSGGGAGSGGAGGTSSGDDGEMPA

MHEIGEELIWTGRFFGGLCLDIKRKLPWFPSDFYDGFHIQSISAILFIYLGCITNAITFG

GLLGDATDNYQGVMESFLGTAMAGSLFCLFSGQPLIILSSTGPILIFEKLLFDFSKGNGL

DYMEFRLWIGLHSAVQCLILVATDASFIIKYITRFTEEGFSTLISFIFIYDAIKKMIGAF

KYYPINMDFKPNFITTYKCECVAPDTVNTTVFNASAPLAPDTNASLYNLLNLTALDWSLL

SKKECLSYGGRLLGNSCKFIPDLALMSFILFFGTYSMTLTLKKFKFSRYFPTKVRALVAD

FSIVFSILMFCGIDACFGLETPKLHVPSVIKPTRPDRGWFVAPFGKNPWWVYPASILPAL

LVTILIFMDQQITAVIVNRKENKLKKAAGYHLDLFWVGILMALCSFMGLPWYVAATVISI

AHIDSLKMETETSAPGEQPQFLGVREQRVTGIIVFILTGISVFLAPILKILGLIIVRRLL

DFIFSQHDLAWIDNILPEKEKKETDKKRKRKKGAHEDCDEEPQFPPPSVIKIPMESVQSD

PQNGIHCIARKRSSSWSYSL

>Mus musculus SLC4A5

MDTLQHDGDQMEWKESARWIKFEEKVEEGGERWSKPHVSTLSLHSLFELRTCLQTGTVLL

DLDSCSLPQIIDDVIEKQIEDGLLRPELRERVSYVLLRKHRHQTKKPIHRSLADIGKSVS

TTNRSSARSSSAGPTLHRSTEDLRIRQSTSYGHLCHAQSRSMNDISHTPNTDQRKNKFMK

KIPKDSEASNVLVGEVDFLDQPFIAFVRLVQSAMLGGVTEVPVPTRFLFILLGPSGRAKS

YNEIGRAIATLMVDDLFSDVAYKARNREDLIAGIDEFLDEVIVLPPGEWDPNIRIEPPKK

VPSADKRKSVFSLAEPGQMNGSVGGGGASAGGGGSGGGAGGSGAGGVGSGDEAEMPAMHE

IGEELIWTGRFFGGLCLDVKRKLPWFPSDFYDGFHLQSISAVLFIYLGCITNAITFGGLL

GDATDNYQGVMESFLGTAMAGSLFCLFSGQPLIILSSTGPILIFEKLLFDFSKANGLDYM

EFRLWIGLHSAIQCLILVATDASFIIKYITRFTEEGFSTLISFIFIYDAIKKMIGAFKYY

PINTDFKPDFITTYKCECVAPDTVNTTTVNASAPLAPNTNTSLYTPLNLTALDWSLLSKK

ECLSYGGRLLGSSCQFVPDLALMSFILFFGTYSMTLTLKKFKFSRYFPTKVRTLVADFSI

VFSILLFCGIDACFGLQTPKLHVPSVIKPTRPDRGWFVAPFGKNPWWVYPASILPALLVT

ILIFMDQQITAVIVNRKENKLRKAAGYHLDLFWVGILMALCSFTGLPWYVAATVISIAHI

DSLKMETETSAPGEQPQFLGVREQRVTGVMVFILTGISVFLAPILKYIPMPVLYGVFLYM

GVASLNGIQFWERCKLFLMPAKHQPDHAFLRHVPLRRIHLFTLVQILCLALLWILKSTMA

AIIFPVMILGLIIVRRLLDLIFSQHDLAWIDNILPEKDKKETDKKKKRRKEVHETAEKEV

AMEKDLPVGVTLSDSSISGSELDRSITLHFKISCPASPAFSYSRSPVFMVPQVRIEMESE

LNLTDDDEEDYGREVGGETTL

>Rattus norvegicus SLC4A5

MKVEEKAGVKKLEPTSYRRRHPEQDFPSIHIGFPVPGYSQRKSDSKGHLSGLQRVQWSLQ

PDKSQQDLAGPDGIKASSLGGSVDFTKRIRSPAAEQLQDILGEEDEAPNPTLFTEMDTLQ

HDGDQMEWKESARWIKFEEKVEEGGERWSKPHVSTLSLHSLFELRTCLQTGTVLLDLDSG

SLPQIIDDVIEKQIEGGLLRPELRERVSYVLLRKHRHQTKKPIHRSLADIGKSVSTTNRS

SARSPSAGPTLHHSTEDLRIRQSTSYGHLCHAQSRSMNDISHTPNTDQRKNKFMKKIPKD

SEASNVLVGEVDFLDQPFIAFVRLAQSSMLGGVTEVPVPTRFLFILLGPSGRAKSYNEIG

RAIATLMVDDLFSDVAYKARNREDLIAGVDEFLDEVIVLPPGEWDPNIRIEPPKKVPSAD

KRKSVFSLAELGQMNGSVGRSGASAGGGGSGGGAGGSGAGGGGSGNEAEMPAMHEIGEEL

IWTGRFFGGLRLDVKRKLPWFPSDFYDGFHIQSISAVLFIYLGCITNAITFGGLLGDATD

NYQGVMESFLGTAMAGSLFCLFSGQPLIILSSTGPILIFEKLLFDFSKANGLDYMEFRLW

IGLHSAIQCLILVATDASFIIKYITRFTEEGFSTLISFIFIYDAIKKMIGAFKYYPINTD

FKPDSITTYKCECVAPDTVNTTTVNDSALLVPNTNMSVYTPLNLTALDWSLLSKKECLSY

GGRLLGSSCQFVPDLALMSFILFFGTYSMTLTLKKFKFSRYFPTKVRTLVADFSIVFSIL

LFCGIDACFGLQTPKLHVPNVIKPTRPDRGWFVAPFGKNPWWVYPASILPALLVTILIFM

DQQITAVIVNRKENKLRKAAGYHLDLFWVGILMALCSFMGLPWYVAATVISIAHIDSLKM

ETETSAPGEQPQFLGVREQRVTGVMVFILTGISVFLAPILKYIPMPVLYGVFLYMGVASL

NGIQFWDRCKLFLMPAKHQPDHAFLRHVPLRRIHLFTLVQILCLALLWILKSTMAAIIFP

VMILGLIIVRRLLDLIFSQHDLAWIDNILPEKEKKESDRKKRRKEVHENTDKEPQFLPPS

VVKIPMEGIPSDPQNGIHCVGRKRSSSWSHSL

>Tetraodon nigroviridis SLC4A5

SPAAERLRYILSEDDDGPTPTLFTEMDTLQREGDELEWKESARWVKFEEKVEEGGERWSK

PHVSTLSLHSLFELRTCLQTGTVLLDLDGYSLPQIVDDIIERQVEEGMISPELREKISFV

LLRKHRHQTKKPIHRSLADIGKSNSSSRNVAPRGGQSVGPMATFNRSTEDLRAKQQPSGY

GRLRHAQSRSMNDIADTPSSDQLKNKFMKKIPRDAEASNVLVGEVDFLNKPFVAFVRLAQ

ATTLGGLTEVPVPTRFLFILLGPQGKAKSYNEIGRAIATLMVDDLFSDVAYKARDREDLI

AGIDEFLDEVIVLPPGEWDPKIRIEPPKKVLSADKRKSVFSLNELGQVNGTAGGGGGGGG

GGGGGGLANDEEMPEQHELGEELAFTGRFCGGLYLDIKRKLPWLPSDIYEGFHIQSISAV

LFIYLGCITNAITFGGLLGDATDNYQGVMESFLGTALAGSVFCLFSGQPLIILSSTGPIL

IFEKLLYEFSKNNSIDYMELRLWIGIHSCLQCLVLVATDASYIIKYMTRFTEEGFSSLIS

FIFISDAIKKMVGSFKYYPINTDFKPDYITTYKCECLAPDPTAAMTFGGSVPVADNSSAV

SGLNVTALDWSQLGKKECLKYGGSLVGKSCKYVPDLALMSFILFFGTYSMTVTLKKFKFS

RYFPTKLRKLISDFSIFMSIMTFVGLDMLMGLKTPKLIVPTEFKPTRPDRGWLVMPFGKN

PWWIYLASFVPALLVTILIFMDQQISAVIVNRKENKLKKGCGYHLDLFWVGILMAACSFM

GLPWYVAATVISIAHIDSLKMESESSAPGEQPQFLGVRLGDGGTFSGAGRQRLALFDPCP

SCREQRITGVLVFALTGVSIFLAPVLKFIPMPVLYGVFLYMGVASLSGIQFWDRIKLYLM

PSKHQPDFSYLRHVPLRKVHLFTLVQILCLAVLWILKSTFLAIIFPVMILGLMVVRKMLD

MIFSQHDLAWLDDLLPEKEKKKKEDIKKKGKGKEKE

>Danio rerio SLC4A5

MESMVLPKPLEDDGNRSEAVWVKFEEKVEEGGERWSKPHVSTLSLHSLFELRTCLQTGTV

LLDLEGYSLPQIVDDIIDRQVEEGLIGPELRDKISFVLLRKHRHQTKKPIHRSLADLGKS

GSGGTRSPARTPAAQAANFNRSTEDLRTKQAANYGRLRHAQSRSMNDIADTPSTDQLKNK

FMKKIPRDAEASNVLVGEVDFLEKPFVAFVRLSQATTLGGLTEVPVPTRFLFVLLGPPGK

AKSYNEIGRAIATLMVDDLFSDVAYKARDREDLIAGIDEFLDEVIVLPPGEWDPKIRIEP

PKKTPSADKRRKSVFSLNELGQMNGTAGGGGPIEEDEEMPVPHELGEELAYTGRFCGGLF

LDMKRKLPWYPSDFYEGFHIQSISAVLFIYLGCITNAITFGGLLGDATDNYQGVMESFLG

TALAGTVFCVFGGQPLIILSSTGPILIFEKLLYEFSKNNAIDYKELRLWIGIHSCIQCFI

LVATDASYIIKYMTRFTEEGFSSLISFIFISDAIKKMVGSFKYYPINTDFKPDYVTAYKC

ECVAPDPITHDEITLMRRKTKTYTSLDWTQLSKKECVKYGGSLVGQSCKYVPDLALMSFI

LFCGTYTMTVTLKKFKFSRYFPTKLRKLISDFSIFMSIMTFVGLDMLIGLKTPKLIVPTE

FKPTRPDRGWFVMPFGKNPWWVYLASFVPALLVTILIFMDQQISAVIVNRKENKLKKGCG

YHLDLLWVGVLMAACSFMGLPWYVAATVISIAHIDSLKMESESSAPGEQPQFLGVREQRL

TGILVFVLTGVSIFLAPILQFIPMPVLYGVFLYMGVASLSGIQFWDRIKLIMMPAKHQPD

FSYLRHVPLRRVHLFTLVQIVCLAVLWILKSTFLAIIFPVMILGLMVVRKMLDMVFSQHD

LAWLDDILPEKEKKKKDDEKKNIKKDKKKGKGGDDSEDDVNKYHPYTNCTPGDSDLDRRY

CVLKLHVPYRDLLQAPDSNSDITLHLKISCPSSPAMPLARGMPCPVPQVKIEMESDYEDT

DINPRGRHRDHRDMSSETTL

>Drosophila melanogaster(CG8177)

MSFSSASGRENNVRKLSFLGFNTKKKSGNEDPDEVLLDSEMDKVFAGNSARKDKFDVNTF

QDNSQLPIGSRKKNSIRTNDLNIEEDSEYESQTEPLNNAQNYDDIPEDFPLVSERAGHGD

HSDNSVDEKHVQFGGKKKIPPSLSYDEQPTDQSHERKRRRSRHQYYRQRKFSHQDSVEPK

NKLEENGDAGARRISVQPEDTALEEADLNELRSHRSDDPRALRRHKIHHSSIKLRELPQI

TISPFTNKKPEVDHSPHEIFVQLDELTGVGEDREWKETARWIKYEEDVEEGSDRWGKPHV

ASLSFHSLLNLRRCLETGVVLLDLNEKDLPAVAYRVVEQMVIEDLIDINDKPSVMRSLLL

RHRHVNEHQGVLPFTKRKYNSYTSLQFLWMGADSAHQPYQQAQPPPRNLAKARRSICVTS

SAPPTAAMLNVATATAAAAAIDHRRHSTMSYLGNLSGTDDKKIKIMPAAEIGGSKRSNEL

KIDMKDDMYSSSQEDLKKLQNDTILKRIPAGAEATTVLVGAVEFLEQPTIAFVRLSEGVL

MPTLTEVPVPVRFMFVLLGPRNFDLDYHEVGRSISTLMANEHFHSIAYKADDRKDLLSAI

NEFLDDSIVLPPGNWDRHDLLPFEELKAKKDWIRTRKIKALQVKRDSEMIKIGKDEEKAL

LEKQTLGAIGFTIGGGDGGGDGDSDDDNGRKKKKPSPLEKTGRLWGGLRNDLKRRMPMYK

SDILDGLNTETLAATIFMYFACLSTAITFGGLVSAKTNSWIGISETLISCSLVGIVFHCL

SCQPLVIIGTTGPLLLFDEALMVFCTQHEFDFLSLRVYVGVWLIIIALTVSAFEGSVYVR

LLTRFTQEIFSALITLIYIVETFMKLISIYRENPLLSDYNLPPPTLVAHEHATNESLLNA

TASVTTNITQMVMNISTTAMPIGPPPLPKNQPNTALFCTILTLATFVVAYYLKLFRNSHF

LGRNARRALGDFGVPISIAIFVLVDYLVPAVYTEKLVVPEGLSPSDPSKRGWYIGFDTSS

TWIPFACVIPALLVYILIFMESQISELIVDKPDRGLKKGSGLHWDIVLLCLLNCACGIFG

MPWHCAATVRSVTHVSSVTIMSRTHAPGESPRIVDVKEQRLSGFFVCLMIGLSVLMAPLL

RLIPMAVLFGVFLYMGVASMSGVQLFERIRLYFMPVKHYPPTSYVKRLRPWKLHLFTTIQ

VLCLVLLWSVKSSQFSLAFPFFLIMMVPIRQNLTKLYKPEEMQALDGSEMKKNDDDEPDF

YEQTNIPA

>Drosophila melanogaster(CG4675-PB)

MAEKNEYIELPWTMNSSSGDDEAPKDPRTGGEDFTQQFTENDFEGHRAHTVYVGVHVPGG

RRHSQRRRKHHHSGPGGGGGGGGGGGSIGGSGSVGGGAGKDNVSEKQQEVERPVTPPAQR

VQFILGEDVDDGTHVSHPLFSEMGMLVKEGDEIEWKETARWIKFEEDVEEGGNRWSKPHV

ATLSLHSLFELRRLLVNGSVMLDMEAQNLEVMADLVCDHMVSAGTLPPGVKDKVKDALLR

RHRHQHEYAKKTRLPIIRSLADMRNHSSSKKKKSNSKHSRPAQNLSSITEDMVKSPSNQS

MARPGSGTELSEQQHKGNTHFMRKIPPGAEASNILVGEVDFLERTLSCFIRLSQAVVLGD

LTEVPVPTRFVFILLGPPGSQSNFHEIGRAMATLMSDEIFHEVAYRARKRDHLLSGVDEF

LDAVTVLPPGEWDPTIRIEPPAAIPSQEVRKRPPELPKEEVDEEEEEARLREENGLSRTG

RLFGGLINDIKRKAPWYISDYKDALSMQCVASWIFLYFACLSPIITFGGLLAEATGKHMA

AMESLVSGFVCGMGYGFFSGQPLTILGSTGPVLVFESIIYEFCLKMGWDYMTFRFWIGMW

VAGICIVLTAIDASALVCYITRFTEENFATLIAFIFIYKAIENVMVIGKNFPVNQGIYDC

VCTPPIGSNASVIDYAKYNWDSCESYNGTLVGGDCGTPPTENVFLMSVVLCAGTFLISTV

LKEFKNALFFPSIVRQYISDFSVLIAIFAMSFFDYSLGVPTQKLEVPNELKPTLSTRGWL

IPPFVEKNPWWSAIIAVFPALLGTILIFMDQQITAVIVNRKENKLKKGCGYHLDLFILSI

LIAICSMMGLPWFVAATVLSINHVNSLKLESECSAPGEKPQFLGVREQRVTHILIFLTIG

VSVLLTPLLGNIPMPVLFGVFLYMGVASLKGLQFFDRILIMFMPAKYQPDYMFLRQVPIK

RVHLFTMIQLACLIILWLIKSFSQTSILFPLMLVVMIGIRKALDLVFTRRELKILDDIMP

EMTKRAAADDLHKLDAEVGLLARIFPWGKGSRSRVVTKPPGLDDGIVGSGGAVGAGLITC

TTSNANEKEFEAQSSLLKK

>Caenorhabditis elegans(abts-1)

MTHSVRRRMSKARRESQSIELSSLAPGTRVMFLLKEKENTPALFTEMGELNNEEWRETAR

WVKFEEDVEQGGNRWSKPHVATLSLHSLFQLRSCIMNGHFMNDLAETDIPSIFNAVIENL

EKSGDLPSENREELREILLRKHVHQYEQAKKNGAGGEKGGFLSTVRSISDIGKSFSHGKN

LSKIEEKPEGPVTSQTAGAPSGSSNQLTLPTVESVPKDISSEESSKGNLHFLKKLPAGVE

ASNVLIGEVDFLTRHICCFIRLKNANQLGDLTEVPVPTRFIFLLLGPTGHGQQYREIGRA

IATLMADEIFHDVAYGARDVDDLLDGIDEFLDQVTVLPPGEWDPNIRIEPPSKLPSQEKR

KQIGQELLVEPSSHKHVKKVVEEEEIHSHGDDPALKRTGKLFGGLILDIKRKAPHFVSDF

TDAFNLQCLASICFMYFGLLAPIVTFGGLLEEATHQRMAAMENLFGGALCGVIYHFFAGQ

PLTIVGSTGPVLVFETIVFDFCHRFGIVYLSFRFWVHVWTAVIIFLMVVTDASALVSFIT

RFTEESFATLIAVIFIYEAIMKLVKIKGQLDMINYSRDFVEGGFCSCVPPIGKPSVSPDH

ARLVIKSTGLPIAFNDTFIDYTAANLTDCRTIGGSFDGTTCFPLYDKLLMSILLTVGTFF

LATTLKKMRNSCYFPSSVRQLLSDFAVMIAIASMTFVDIFVGVNTPKLNVPSTFRPTWEG

RGWFIPPFDANEWWTAPLAILPALLACILIFMDQQITTVIVNRKENKLKKGCGYHLDLLV

LSVSILMVGFLGLPIYVAATVLSINHINSLKVESECKAPGEVAQFVGVREQRVTGIVTFL

IIGLSVLATNILGRIPMPVLYGVFLYMGISALGGIQLFDRTLLLFMPMKYQPDTIYIRHV

PIRKIHLFTAFQIGCLALLWVIKSIKSTSILFPIMLVVMVAIRKMMEKAFTTTDLKYLDD

PMPDFHLRKKEDAKRRQSEGEAVEIEFDNENQATIHAVKTEAHLHIPMASGNVIKIPLAA

IQEPSHSINLTKEVNNSGMWKHITSVDSKNSLVRDVPASSDPTSSRDAPIEQPEDDDDPI

MIKVIRPSPHTSTSNLTTENTPLLKDEEPPKTNPDEPKV

**SP family**

>Homo sapiens SP1

MSDQDHSMDEMTAVVKIEKGVGGNNGGNGNGGGAFSQARSSSTGSSSSTGGGGQESQPSP

LALLAATCSRIESPNENSNNSQGPSQSGGTGELDLTATQLSQGANGWQIISSSSGATPTS

KEQSGSSTNGSNGSESSKNRTVSGGQYVVAAAPNLQNQQVLTGLPGVMPNIQYQVIPQFQ

TVDGQQLQFAATGAQVQQDGSGQIQIIPGANQQIITNRGSGGNIIAAMPNLLQQAVPLQG

LANNVLSGQTQYVTNVPVALNGNITLLPVNSVSAATLTPSSQAVTISSSGSQESGSQPVT

SGTTISSASLVSSQASSSSFFTNANSYSTTTTTSNMGIMNFTTSGSSGTNSQGQTPQRVS

GLQGSDALNIQQNQTSGGSLQAGQQKEGEQNQQTQQQQILIQPQLVQGGQALQALQAAPL

SGQTFTTQAISQETLQNLQLQAVPNSGPIIIRTPTVGPNGQVSWQTLQLQNLQVQNPQAQ

TITLAPMQGVSLGQTSSSNTTLTPIASAASIPAGTVTVNAAQLSSMPGLQTINLSALGTS

GIQVHPIQGLPLAIANAPGDHGAQLGLHGAGGDGIHDDTAGGEEGENSPDAQPQAGRRTR

REACTCPYCKDSEGRGSGDPGKKKQHICHIQGCGKVYGKTSHLRAHLRWHTGERPFMCTW

SYCGKRFTRSDELQRHKRTHTGEKKFACPECPKRFMRSDHLSKHIKTHQNKKGGPGVALS

VGTLPLDSGAGSEGSGTATPSALITTNMVAMEAICPEGIARLANSGINVMQVADLQSINI

SGNGF

>Mus musculus Sp1

MSDQDHSMDEVTAVVKIEKDVGGNNGGSGNGGGAAFSQTRSSSTGSSSSSGGGGGQESQP

SPLALLAATCSRIESPNENSNNSQGPSQSGGTGELDLTAAQLSQGANGWQIISSSSGATP

TSKEQSGNSTNGSESSKNRTVSGGQYVVAATPNLQNQQVLTGLPGVMPNIQYQVIPQFQT

VDGQQLQFAATGAQVQQDGSGQIQIIPGANQQIIPNRGSGGNIIAAMPNLLQQAVPLQGL

ANNVLSGQTQYVTNVPVALNGNITLLPVNSVSAATLTPSSQAGTISSSGSQESSSQPVTS

GTAISSASLVSSQASSSSFFTNANSYSTTTTTSNMGIMNFTSSGSSGTSSQGQTPQRVGG

LQGSDSLNIQQNQTSGGSLQGSQQKEGEQSQQTQQQQILIQPQLVQGGQALQALQAAPLS

GQTFTTQAISQETLQNLQLQAVQNSGPIIIRTPTVGPNGQVSWQTLQLQNLQVQNPQAQT

ITLAPMQGVSLGQTSSSNTTLTPIASAASIPAGTVTVNAAQLSSMPGLQTINLSALGTSG

IQVHQLPGLPLAIANTPGDHGTQLGLHGSGGDGIHDETAGGEGENSSDLQPQAGRRTRRE

ACTCPYCKDSEGRASGDPGKKKQHICHIQGCGKVYGKTSHLRAHLRWHTGERPFMCNWSY

CGKRFTRSDELQRHKRTHTGEKKFACPECPKRFMRSDHLSKHIKTHQNKKGGPGVALSVG

TLPLDSGAGSEGTATPSALITTNMVAMEAICPEGIARLANSGINVMQVTELQSINISGNG

F

>Rattus norvegicus Sp1

FLDQDHSMDEVTAVKIEKGVGGNNGGSGNGGGAAFSQTRSSSTGSSSSSGGGGGQESQPS

PLALLAATCSRIESPNENSNNSQGPSQSGGTGELDLTATQLSQGANGWQIISSSSGATPT

SKEQSGNSTNGSNGSESSKNRTVSGGQYVVAATPNLQNQQVLTGLPGVMPNIQYQVIPQF

QTVDGQQLQFAATGAQVQQDGSGQIQIIPGANQQIITNRGSGGNIIAAMPNLLQQAVPLQ

GLANNVLSGQTQYVTNVPVALNGNITLLPVNSVSAATLTPSSQAGTISSSGSQESGSQPV

TSGTAISSASLVSSQASSSSFFTNANSYSTTTTTSNMGIMNFTSSGSSGTSSQGQTSQRV

GGLQGSDSLNIQQNQTSGGSLQGSQQKEGEQSQQTQQQQILIQPQLVQGGQALQALQAAP

LSGQTFTTQAISQETLQNLQLQAVQNSGPIIIRTPTVGPNGQVSWQTLQLQNLQVQNPQA

QTITLAPMQGVSLGQTSSSNTTLTPIASAASIPAGTVTVNAAQLSSMPGLQTINLSALGT

SGIQVHQLPGLPLAIANTPGDHGAQLGLHGPGGDGIHDETAGGEEGENSPDPQPQAGRRT

RREACTCPYCKDSEGRGSGDPGKKKQHICHIQGCGKVYGKTSHLRAHLRWHTGERPFMCN

WSYCGKRFTRSDELQRHKRTHTGEKKFACPECPKRFMRSDHLSKHIKTHQNKKGGPGVAL

SVGTLPLDSGAGSEGSGTATPSALITTNMVAMEAICPEGIARLANSGINVMQVTELQSIN

ISGNGF

>Xenopus tropicalis Sp1

MSDQEHTSDDMPTIKSENRTGGGYIQKGQDSQPSPLALLAATCSRIEPPENGNGNSQQQG

ATELDLSTAQLAQTANGWQIISTAGSASKDQAGGDASSKNRPIAPGQFVVSTPSVQNQQV

LASLQGVMPNIQYQVIPQFQTVDGQQLQFTTAPAQVSVQQDASGQFQIIPATNQQIITTN

RTGTGNILAMPNLLQQAVPIQGMGLTNNVLSGQTQYLTNVPVALNGNITLLPVNAASLTP

TSQSVTLSGTQENNSQPVTSGVAISSSQLASQANSGAYFTNANSFSTTTTPSNVSLMNCS

SAGSTPGSNVQVQTSQRSTGQLSLLTDSVQQGNGNMQKDGDQNQQQQILIQPQIVQSGQT

IQALQAAQLSGQAFSTQAISQDALQNLQIQTVPNTGPIIIRTPAVGPNGQVTWQTIQLQN

LQVQNPQAQTITLAPMQGVSLGQSGSTNTLTSMPAGTVTVNAAQLSSMPGLQTINLGALG

ASGIQVHQLQGVPLTITNATGDIASGTIR

>Gallus gallus Sp1

MSDSESPEEMAAVKGTPERGEGDGSSDSSSPAGEGQESQPSPLALLAATCSRIESPNENS

NSSQGQQGGSGELDLTAAAAQIAQSANGWQIISTGSTTPTPSKEQGSNGSNGDPSKSRPV

STGPYVVTAASNLQNQQVLTGLPGVIPNIQYQVIPQFQTIDGQQLQFATTPAQVNVQQDA

SGQLQIIPGTNQQIITSRAGTGNLIAMPNLLQQAVPIQGVGLANNTLSGQTQYVANVPVA

LNGNITLLPVNSVAASLAPTSQTVTLSGSGSPDSSSQPTTSGAAISSSNIATSHASSGSF

FTNANSYSTTTTTSNMGIMNFSSSSTVGTNVQVQTPQRAPSTDSLQSQVSGVALQPGQQK

EGDQSQQSQQQQQQILIQPQLVQGGQTIQALQTAPLSGQTFATQAISQDALQNLQLQAVP

NSGPIIIRTPTVGPNGQVSWQTIQLQNLQVQNPQAQTITLAPMQGVSLGQTGSTSTTLTP

IASLPSGTVTVNAAQLSSMPGLQTINLSALGASGIQVHQLQGLPLAIANATGEHGTQLGL

HGASGDGLGDDSAAVEEGETSPDPQPQQGRKVRREACTCPYCKDSEGRSSGDPGKKKQHI

CHIPGCGKVYGKTSHLRAHLRWHTGERPFICGWMLCGKRFTRSDELQRHKRTHTGEKKFA

CPECPKRFMRSDHLSKHIKTHQNKKGGAANSVAMNVSAVPMDTGASSEGSGGPTPSALIA

TNMVAMEAICPDGIARLASSGINVMQVADLQSINISGNGF

>Tetraodon nigroviridis SP1

MSWLRLHQCQGEQVLTTISGVMPNIQYQVIPSFQTVDGQPLQLAHAQQESAGPAAAAPGQ

QFQIVSSPNGQQIIAATNRAGAAGNIITMPSLLQGAIPIQNISLGNSVLKNQPQFLANMP

VSLNGNITLLPISTAASSGTAGDANGGDTARNPITQQQPASSNSATGYATSVSTAQTSTS

YGLTQTQNSNGVMTGTFQQNAATLGVPIQPENREGQQPQQILIQPQQVLPGGATLQGIQA

GTVATAGGQVFAAPTISQEGLQNLQIMPNAGPILLHTVGPNGQVSWQTIQIQNPAGPQIT

LAPVQTLPQLGQGQGAAAASGVSVNAVQIPGIQTINLNTLGGSGLQLQTVPLTLANAAGE

KRFACPECPKRFMRSDHLSKHIKTHMNKKVQAASASGPAGSTDSSSPAIVGTKVETGAAA

TADQHTIVTMETLSAESIARLASSGINMMQVDLHQMNGNNY

>Takifugu rubripes SP1

QPSPLALLAATCSRIDTPGESDSPSDQQNQQQQLELNQGAFTSNSNGWQVIPLNIQASSG

ANTATTDSSAVMAGGDAGKSRQLLSPSVAVGSTQGQQHQPQQYVVAQAPSMPGQQVLTTI

SGVMPNIQYQVIPQFQTIDGQPLQLAHAQQESAVPAAAAPGQQFQIVSSPNGQQLIAATN

RAGTAGNIITMPSLIQGAIPIQNISLGNGMLKNQPQFLANMPVSLNGNITLLPISTATGG

TGADTNGGDTARNQVTQQQQPVSSTGSTGYVTSVSTAQTATSYGLTQTQNSNGVMTFQQN

TANLGVPIQPESREGQPQQQILIQPQQVIQGGATLQPIQAGTVATAGGQVFAAPTISQEG

LQNLQIMPNTGSILLHTVGPNGQVSWQTIQIQNPAGPQITLAPMQTLPQLGQAQGAAAAA

GVSVNAVQIPGIQTINLNTLGGSGLQLQTVPITLANTAGDHQNLHSGGDTLDDGTAIDEG

DMSPPPQGRRNRREACTCPFCKDGEGRDPTKKKQHICHISGCGKIYGKTSHLRAHLRWHT

GERPFVCSWSFCGKRFTRSDELQRHKRTHTGEKRFACPECPKRFMRSDHLSKHIKTHTNK

KVPAASASGIAGSSDPTAVGTKVEQGAGAASDQHTIVTMETLSAESIARLASSGLNMMQV

DLHPMN

>Homo sapiens SP4

MATEGGKTSEPENNNKKPKTSGSQDSQPSPLALLAATCSKIGTPGENQATGQQQIIIDPS

QGLVQLQNQPQQLELVTTQLAGNAWQLVASTPPASKENNVSQPASSSSSSSSSNNGSASP

TKTKSGNSSTPGQFQVIQVQNPSGSVQYQVIPQLQTVEGQQIQINPTSSSSLQDLQGQIQ

LISAGNNQAILTAANRTASGNILAQNLANQTVPVQIRPGVSIPLQLQTLPGTQAQVVTTL

PINIGGVTLALPVINNVAAGGGTGQVGQPAATADSGTSNGNQLVSTPTNTTTSASTMPES

PSSSTTCTTTASTSLTSSDTLVSSADTGQYASTSASSSERTIEESQTPAATESEAQSSSQ

LQPNGMQNAQDQSNSLQQVQIVGQPILQQIQIQQPQQQIIQAIPPQSFQLQSGQTIQTIQ

QQPLQNVQLQAVNPTQVLIRAPTLTPSGQISWQTVQVQNIQSLSNLQVQNAGLSQQLTIT

PVSSSGGTTLAQIAPVAVAGAPITLNTAQLASVPNLQTVSVANLGAAGVQVQGVPVTITS

VAGQQQGQDGVKVQQATIAPVTVAVGGIANATIGAVSPDQLTQVHLQQGQQTSDQEVQPG

KRLRRVACSCPNCREGEGRGSNEPGKKKQHICHIEGCGKVYGKTSHLRAHLRWHTGERPF

ICNWMFCGKRFTRSDELQRHRRTHTGEKRFECPECSKRFMRSDHLSKHVKTHQNKKGGGT

ALAIVTSGELDSSVTEVLGSPRIVTVAAISQDSNPATPNVSTNMEEF

>Mus musculus SP4

VADQKKEEEEEAAAAMATEGGKTSEPENNNKKPKTSGSQDSQPSPLALLAATCSKIGTPG

ENQATGQQQIIIDPSQGLVQLQNQPQQLELVTTQLAGNAWQLVASTPPASKENNVSQPAS

SSSSSSSSNNGSSSPTKTKSGNPSTPNQFQVIQVQNPSGSVQYQVIPQLQTVEGQPIQIN

PTSSSSLHDSQGQIQLISAVPLQIRPGVSIPLQLQTLPGTQAQVVTTLPINIGGVTLALP

VINNVTAGGGTGQVGQPTTTTDSGTSNGNQLVSTPTTSTAPASTMPESPSSSTTCTTTAS

TTLTSSDTLVSSADTGQYASTSASSSERTIEEPQTPAATESEAQSSSQLQSNGIQNAQDQ

SNSLQQVQIVGQPILQQIQIQQPQQQIIQAIPPQSFQLQSGQTIQTIQQQPLQNVQLQAV

NPTQVLIRAPTLTPSGQISWQTVQVQNIQSLSNLQVQNAGLSQQLTITPVSSSGGTTLAQ

IAPVAVAGAPITLNTAQLASVPNLQTVSVANLGAAGVQVQGVPVTITSVAGQQQGQDGVK

VQQATIAPVTVAVGGIANATIGAVSPDQLTQVHLQQGQQTSDAEVQPGKRLRRVACSCPN

CREGEGRGSSEPGKKKQHVCHIEGCGKVYGKTSHLRAHLRWHTGERPFICNWMFCGKRFT

RSDELQRHRRTHTGEKRFECPECSKRFMRSDHLSKHVKTHQNKKGGGTALAIVTSGELDS

SVTEVLGSPRIVTVAAISQDSNPATPNVSTNMEEF

> Rattus norvegicus SP4

MSDQKKEEEEEAAAAMATEGGKTSEPENNNKKPKTSGSQDSQPSPLALLAATCSKIGTPG

ENQATGQQQIIIDPSQGLVQLQNQPQQLELVTTQLAGNAWQLVASTPPASKENNVSQPAS

SSSSSSSSNNGSSSPTKTKSGNPSTPNQFQVIQVQNPSGSVQYQVIPQLQTVEGQQIQIN

PTSSSSLQDLQGQIQLISAGNNQAILTAANRTASGNILAQNLANQTVPVQIRPGVSIPLQ

LQTLPGTQAQVVTTLPINIGGVTLALPVINNVTAGGGTGQVGQPTTTTTDSGTSNGNQLV

STPTTSTAPASTMPESPSSSTTCTTTASTSLTSSDTLVSSADTGQYASTSASSSERTIEE

PQTPAATESEAQSSSQLQSNGIQNAQDQSNSLQQVQIVGQPILQQIQIQQPQQQIIQAIP

PQSFQLQSGQTIQTIQQQPLQNVQLQAVNPTQVLIRAPTLTPSGQISWQTVQVQNIQSLS

NLQVQNAGLSQQLTITPVSSSGGTTLAQIAPVAVAGAPITLNTAQLASVPNLQTVSVANL

GAAGVQVQGVPVTITSVAGQQQGQDGVKVQQATIAPVTVAVGGIANATIGAVSPDQLTQV

HLQQGQQTSDAEVQPGKRLRRVACSCPNCREGEGRGSSEPGKKKQHVCHIEGCGKVYGKT

SHLRAHLRWHTGERPFICNWMFCGKRFTRSDELQRHRRTHTGEKRFECPECSKRFMRSDH

LSKHVKTHQNKKGGGTALAIVTSGELDSSVTEVLGSPRIVTVAAISQDSNPATPNVSTNM

EEF

>Xenopus tropicalis Sp4

MASEGGKASESENKKKGKASGSQDSQPSPLALLAATCSKIGTTGENQGAGQQQIIIDPSQ

GLVQLQNQPQQLELVTTQLAGSAWQIVAAAPPVSKENNITQQGVAIAASTAASSSINSEN

ASPTKSKPSNAATAPGQFQVIQVQTPGGNVQYQVIPQIQTVEGQHIQISPGNTTALQDLQ

GQIQFIPAGNNQAIITAGNRTASGNIIAQNLANQTVPVQIRPGVSIPLQLQTIPGAPAQV

VTTLPINIGGVTLALPVINSMPAGGGSAQIVQPTDGGVSNGNQLLSTPITTSSVSTMPES

PSSSSSSTCTTTVSATLTSSDTLVSSAETNQYTSTSASAERNTEESPSSVTDGEAQSSNQ

LQSNGLQNMSEQSNSLQQVQIVGQPVLQQIQIQQPQQQIIQAIPQQSFQLQSGQTIQTLQ

QQQLQNVQLQALSPTQVLIRAPTLTPSGQISWQTVQVQNIQSLQNLQVQNPGLPQQLTLT

PVSTSSGTTIAQIAPVTVAGTPITLNAAHFTSVPNLQTVSVANLGAAGVQVQGVPVTITS

VGGQQQGAEGVKVQQATIAPVTVAVGGIANATIGAVSPDQLTQVQLQQSQPSSDQEVQTG

KRLRRVACSCPNCREGEGRGNSEPGKKKQHICHIENCGKVYGKTSHLRAHLRWHTGERPF

VCNWIFCGKRFTRSDELQRHRRTHTGEKRFECPECSKRFMRSDHLSKHVKTHQNKKGGGT

ALAIVTSGDLDSSVTGVLGSPRIVTVAAISQDSNPATPNVSTNIDEF

>Gallus gallus Sp4

FADQKKEETMPTEGEKSPEAENNNNNNKKGKTGGSQDSQPSPLALLAATCSKIGTPGENQ

ATGQQQIIIDPNQGLVQLQNQPQQLELVTTQLAGNSWQLVAASPSASKDNNVAQQGSSVA

SSAPSPSSSNNGSASPTKTKSGNSSATTPGQFQVIQVQNPSGSVQYQVIPQIQTTEGQQI

QINPANATGLQDIQGQIQLIPAGNNQAILTASNRTASGNIIAQNLANQTVPVQIRPGVSI

PLQLQTIPGTQAQVVTTLPINIGGVTLALPVINNMAAGGGSGQVGQSTEGGVSNGSQLAS

TPVTSASVSSMPDSPSSSSTSTTTASTSLTSSDTLVSSAETGQYTSTAGSSSEQPTEESQ

TTATDSEAQSSSQLQSNGLQNVQDQSGSLQQVQIVGQPILQQIQIQQPQQQIIQAIPPQS

FQLQSGQTIQTIQQQSLQNVQLQAVSPTQVLIRAPTLTPSGQISWQTVQVQNLQSLSNLQ

VQNAGLPQQLTITPVSSSGGTTIAQIAPVAVAGTPITLNAAQLASVPNLQTVSVANLGAA

GVQVQGVPVTITSVAGQQQGQDGVKVQQATIAPVTVAVGGIANAGIGAVSPDQITQVQLQ

QAQQASDQEVQPGKRTRRVACSCPNCREGEGRSSNEPGKKKQHICHIEGCGKVYGKTSHL

RAHLRWHTGERPFICNWVFCGKRFTRSDELQRHRRTHTGEKRFECPECSKRFMRSDHLSK

HVKTHQNKKGGGTALAIVTSGELDSSVTEVLGSPRIVTVAAISQDSNPATPNVSTNMEEF

>Tetraodon nigroviridis SP4

SQPSPLALLAATCSKIGGQGGAEGAQAGAQQIQVQAGPIQLQAGQLQGQIVVDAAGGQAL

VPQQLELVPAQFTGNGWQIITTAPTMAKETSNQPVAVTVATTLANDSSPGGRKAKEVGPA

AQQQQQQQQFQIIQVQNLPSAGSGVQYQVIPHLQTADGQQIHISPAQTAPIGALPEQVQL

IQTQSPSQTQAILQPASQQAILTSTANQTVPLQIRPAQSFPLQLQTLQGSQAPVMTTVPI

NIGGMTLALPVINNVAGAGAVQLIQSADGTFSVANGNQLVTTALPGPAPASSGSTAVAEG

DGVSDGTQMVSVGAEGAQGDGQVQSGEADSQTQNQANGVQNQPDGTGTVQQVIVGQVGHQ

LVQQIQLQPQAQSPGQAQGQQQPQHIQTLQLAPGQTLQPIQAFQNPAQVLIRTPTLSPSG

QITWQTLQLPSGVSLQGGLGAAVPQQLTLAPVASGTPVGSGGLVSLSGAPLTLSAAQINP

GSGVQTVSIAGLGAAGVQVQGVPLTITGLQGQPQGQDGVKVQSSPVTVAVGNVASGSSMS

PEQLGSVQSSSEQDGPPNKRLRRVACSCPNCRDGEGRNSGDPTKKKQHICHMEGCGKVYG

KTSHLRAHLRWHTGERPFVCNWIFCGKRFTRSDELQRHRRTHTGGKPLDTRGRTPGRAFF

SRVLFLFPSSQGKSASSVRSAPSASCAATTSPSTSKPTRTRRAEPRWQSSPPKTWRRTPP

KAWARPLTSSPWRLSPATLTPRRPPPLITWRKRRTRTRSLSRRRTLLFTES

>Takifugu rubripes SP4

SQPSPLALLAATCSKIGGQGGAEVAQAGAQPIQVQAGQIQLQAGQLQGQIVVDTAGGQAL

VPQQLELVPAQFTGNGWQIITTAPTMAKETSNQPVAVTVATTLANDSSPGGRKVKPVGGT

NSSPATQQQFQIIQVQNLPSAGSGVQYQVIPHLQTADGQQIHISPAQTAPIGALPEQVQL

IQTQSPSQTQAILQPANQQAILTSSANQTVPLQIRPAQSFPLQLQTLQGSQAPVMTTVPI

NIGGMTLALPVINNVGGAGAVQLIQAADGTLSVANGNQLVTTAVPGPAPGTASSGSTAVA

DGDSASDGTQMVSVGTEGAQADAQVQSSEADSQTQNQANGLQSQTDATGTVQQVIVGQVG

HQFVQQIQLQPQAQSPGQVQGQQQPQHIQTLQLAPGQTLQPIQAFQNPAQVLIRTPTLSP

SGQLTWQTLQLPSSVSLQSGLGAAVPQQLTLAPVAGGTPMGGGGLVSLSGAPLTLSAAQI

NPGSGVQTVSIAGLGAAGVQVQGVPLTITGIQGQPQGQDGVKVQSSPVTVAVGNVASGSS

MSPEQLGSVQSSSEQDGPPNKRLRRVACSCPNCRDGEGRNSGDPTKKKQHICHMEGCGKV

YGKTSHLRAHLRWHTGERPFVCNWIFCGKRFTRSDELQRHRRTHTGGEQQPCKGARFFYS

GIFFLARILCLCPREKSASSVRSAQSASCAATTSPSTSRPTRTRRVELRWQSSPLKTWRK

TLPKAWARPLTLSPWRLSRVTLTLLRPPPLITWRKRRRTTRSLSRR

>Danio rerio Sp4

MSDQKKEAMATDGGKASGGDGGTKGKTSGSQDAQPSPLALLAATCSKIGGVGAEGQAAAQ

QQIIIDPNQGLLQLQNPTQQLELVPAQLTGNGWQIIATSPATATKDSIQQAGANAASSEA

AAGRKVKAVGSNNAAQQQFQIIQVQNLPNSSGGIQYQVIPHLQTADGQQIQISPSNPAAL

SVQPEQIQLISTGNNQAILATPQRTGSASIVQNQTIPLQIRPSFPLQLQTIQGTQAPMVT

TLPINLGGVTLALPVINNVAGAGGSVQLLQSSDGSISNGNQLITTVTTSTADSTGSSTST

TTATGSDSVVVTSADGLSTTSMVSAESPKDNEADLTQSNGLQSTSEQAGQIQQFQIVGHP

VLQQIQIQSPQQQVVQGSIQIQPGQTLQPVQQNLQLQAFQNPAQVLIRTPTLTPSGQISW

QTVQVQNAGMPQQLTLAPVASSAGGGTFAQIAPLTLGGSPITLSAAQLPAGSGVQTVNIA

GLGAAGVQVQGVPLTITGVQGQQQGQDGVKVQSAPVTVTVGNISNAVSPEQMGQVQSPSD

QEGQPSKRLRRVACSCPNCRDGEGRNNSDPSKKKQHVCHMEGCGKVYGKTSHLRAHLRWH

TGERPFVCNWIFCGKRFTRSDELQRHRRTHTGEKRFECPECSKRFMRSDHLSKHIKTHQN

KKGGAALTIITTDEMEEEVDEVLGSPRIVTVATLSHDSNPATPTTSNNLEEEFE

>Homo sapiens SP3

MTAPEKPVKQEEMAALDVDSGGGGGGGGGHGEYLQQQQQHGNGAVAAAAAAQDTQPSPLA

LLAATCSKIGPPSPGDDEEEAAAAAGAPAAAGATGDLASAQLGGAPNRWEVLSATPTTIK

DEAGNLVQIPSAATSSGQYVLPLQNLQNQQIFSVAPGSDSSNGTVSSVQYQVIPQIQSAD

GQQVQIGFTGSSDNGGINQESSQIQIIPGSNQTLLASGTPSANIQNLIPQTGQVQVQGVA

IGGSSFPGQTQVVANVPLGLPGNITFVPINSVDLDSLGLSGSSQTMTAGINADGHLINTG

QAMDSSDNSERTGERVSPDINETNTDTDLFVPTSSSSQLPVTIDSTGILQQNTNSLTTSS

GQVHSSDLQGNYIQSPVSEETQAQNIQVSTAQPVVQHLQLQESQQPTSQAQIVQGITPQT

IHGVQASGQNISQQALQNLQLQLNPGTFLIQAQTVTPSGQVTWQTFQVQGVQNLQNLQIQ

NTAAQQITLTPVQTLTLGQVAAGGAFTSTPVSLSTGQLPNLQTVTVNSIDSAGIQLHPGE

NADSPADIRIKEEEPDPEEWQLSGDSTLNTNDLTHLRVQVVDEEGDQQHQEGKRLRRVAC

TCPNCKEGGGRGTNLGKKKQHICHIPGCGKVYGKTSHLRAHLRWHSGERPFVCNWMYCGK

RFTRSDELQRHRRTHTGEKKFVCPECSKRFMRSDHLAKHIKTHQNKKGIHSSSTVLASVE

AARDDTLITAGGTTLILANIQQGSVSGIGTVNTSATSNQDILTNTEIPLQLVTVSGNETM

E

>Mus musculus Sp3

MTAPEKPVKQEEMAALDVDGGGGGGGHGEYLQQQQQQQQQHGNGAAAAAAQDTQPSPLAL

LAATCSKIGPPSPGDDDEEAAVAAAAGVPAAAAGATGDLASAQLGGAPNRWEVLSATPTT

IKDEAGNLVQIPGAATSSGQYVLPLQNLQNQQIFSVAPGSDSSNGTVSNVQYQVIPQIQS

TDAQQVQIGFTGSSDNGGINQENSQIQIIPGSNQTLLASGTPPANIQNLIPQTGQVQVQG

VAIGGSSFPGQTQVVANVPLGLPGNITFVPINSVDLDSLGLSGSSQTMTAGINADGHLIN

TGQAMDSSDNSERTGERVSPDVNETNADTDLFVPTSSSSQLPVTIDSTGILQQNTNSLTT

TSGQVHSSDLQGNYIQSPVSEETQAQNIQVSTAQPVVQHLQLQDSQQPTSQAQIVQGITP

QTIHGVQASGQNISQQALQNLQLQLNPGTFLIQAQTVTPSGQITWQTFQVQGVQNLQNLQ

IQNTAAQQITLTPVQTLTLGQVAAGGALTSTPVSLSTGQLPNLQTVTVNSIDSTGIQLHP

GENADSPADIRIKEEEPDPEEWQLSGDSTLNTNDLTHLRVQVVDEEGDQQHQEGKRLRRV

ACTCPNCKEGGGRGTNLGKKKQHICHIPGCGKVYGKTSHLRAHLRWHSGERPFICNWMFC

GKRFTRSDELQRHRRTHTGEKKFVCPECSKRFMRSDHLAKHIKTHQNKKVIHSSSTVLAS

VEAGRDDALITAGGTTLILANIQQGSVSGIGTVNTSATSNQDILTNTEIPLQLVTVSGNE

TME

> Rattus norvegicus Sp3

AAAAGATGDLASAQLGGAPNRWEVLSATPTTIKDEAGNLVQIPGAATSSGQYVLPLQNLQ

NQQIFSVAPGSDSSNGTVSNVQYQVIPQIQSTDAQQVQIGFTGSSDNGGINQESSQIQII

PGSNQTLLASGTPPANIQNLIPQTGQVQVQGVAIGGSSFPGQTQVVANVPLGLPGNITFV

PINSVDLDSLGLSGSSQTMTAGINADGHLINTGQAMDSSDNSERTGERVSPDVNETNADT

DLFVPTSSSSQLPVTIDSTGILQQNTNSLTTTSGQVHSSDLQGNYIQSPVSEETQAQNIQ

VSTAQPVVQHLQLQDSQQPTSQAQIVQGITPQTIHGVQASGQNISQQALQNLQLQLNPGT

FLIQAQTVTPSGQITWQTFQVQGVQNLQNLQIQNTAAQQITLTPVQTLTLGQVAAGGALT

STPVSLSTGQLPNLQTVTVNSIDSTGIQLHPGENADSPADIRIKEEEPDRKSQLSGDSTL

NTNDLTHLRVQVVDEEGDQQHQEGKRLRRVACTCPNCKEGGGRGTNLGKKKQHICHIPGC

GKVYGKTSHLRAHLRWHSGERPFICNWMFCGKRFTRSDELQRHRRTHTGEKKFVCPECSK

RFMRSDHLAKHIKTHQNKKVIHSSSTVLASVEAGRDDALITAGGTTLILANIQQGSVSGI

GTVNASATSNQDILTNTEIPLQLVTVSGNETME

>Xenopus tropicalis Sp3

LAAPEQPVKREEMAAMEVDGQSEYLQQHGDSSSAGDDSDQGGGGGQTEDLAVQISGGPNR

WEVLTPAQAALKDEAGNIVQIPGGATVTSSGQYVLPIQTLQNQHLFSVAPGSDTANGTLS

NVQYQVIPQLQTSDGQQVQLGFTTSSDNGGLDQDGGQIQIIPGNHSGSLLSSSPNILTQA

GQIQHSGGSVQIQGVTIGGTSYAGQTQVVANVPIGLPGNITFVPINSVDLDSLGLTGSTE

AMTAGLTADGHLISGHGMENSENSERTAEHEHISPDGVDTNSDTDLFVPTSSSSQLPVTI

DGTGMLQQNANSLTDSPSGHVQTSDLHGNYIQTSMTDESQNIQVSASQTMVQQIHLQDSQ

PTSQAQLVQSIAQQAIQAVQGGTQGLSSQALQNLQLQLNPGTFLIQAQTVTPSGQIAWQT

FQVQGVQNLQNLQIPNSSAQQITLTPVQTLTLGQVAGGGTPVSLNAGQLPNLQTVTVNSV

DAAGIHLHHGDNSDSPTEIETHQTRQHFSSLQLSNFGIRIKEEEPDPDEWQLAGDSTVNT

NDLTHLRVQVVDDELDQPNQEGKRLRRVACTCPNCKDGAGRGTNLGKKKQHICHIPGCGK

VYGKTSHLRAHLRWHSGERPFVCTWMFCGKRFTRSDELQRHRRTHTGEKKFVCPECSKRF

MRSDHLAKHIKTH

>Gallus gallus Sp3

MQLMNHSQTGDLASVQLAGTPNRWEVLSAAPATIKDEAGNIVQIPGAATVTSSGQYVLPI

QSLQNQQIFSVAPGSDSSNGTVSNVQYQVIPQIQTADGQQVQLGFAASSDNSSINQETGQ

IQIIPGSNQTIIASGSPSANIQNILSQSGQVQVQGVAIGGSSFPGQAQVVANVPLGLPGN

ITFVPINSVDLDSLGLGSGSQTMTAGINADGHLINTGQAMDSSDNSERTGEQVSPEITET

ATDNDLFVPTSSSSQLPVTIDSSSILEQNANNLTTTSGQVHSSDLQGNYIQTSVSDDTQA

QNIQVSTAQPIVQHIQLQESQQPTSQAQIVQGIAQQTIHGVQASQSISPQALQNLQLQLN

PGTFLIQAQTVTPSGQITWQTFQVQGVQNLQNLQIQNAPGQQITLTPVQTLTLGQVAAGG

ALTSTPVSLSTAQLPNLQTVTVNSIDSAGIQLHQGENAGSPAGCTTLIQQHQTEHWEEVK

PEKRIVAKVTDALSGASVDMFNNINTNDLTHLRVQVVDEEGDQPHQEGKRLRRVACTCPN

CKEGGGSRGSNLGKKKQHICHIPGCGKVYGKTSHLRAHLRWHSGERPFVCNWMFCGKRFT

RSDELQRHRRTHTGEKKFVCPECSKRFMRSDHLAKHIKTHQNKKGIHSSSTVLASVEATS

DDTLITAGGTTLILANIQQGSVSGIGTVNTSGTSN

>Tetraodon nigroviridis Sp3

ATQLTGTDRWEVLTPTSEKDESGMIQIQSHGVFTSNGQYVLPLQNLQSQPVFVTSETDTS

SAASVPNIQYQVIPQIQTADGQLSFSTSTMEGAALAQDATGQIQILPDGSQTLGVSSAAN

ILNNNQNLISQTGNVQQIQGVSIGSSTFSNQGQVVTNVPVGLPGNITFVPINSVDLDSLG

LSGAQTIATGVTADGQLIMAGQPVDGSQNLAKTENHLPQTLPGNDSSANPELYVPTSSSQ

SQLHIPASEAALLTQDASLSSEATARSNSSSGLQEGFVQENQEPNVQASSAQPIIQLQQV

PIQTANGQVVPSVSPGGQGLQNVQLINPGTFIIQAQTVTPSGQIQWQTFQVQGVQNLQNL

QLPTSPPQQITLAPVQALSLGSAPVSISTGQIPNLQTVTVNSVAPNEGEAEGLGDIQIKE

EPDSGDWQLSTDSTLNTSDLSHLRGRLVDEDDQIGQEGKRLRRVACTCPNCKESGGRGSS

TGKKKQHICHIAGCGKVYGKTSHLRAHLRWHSGERPFVCSWMLCGKRFTRSDELQRHRRT

HTGEKKFVCPECSKRFMRSDHLAKHIKTHQNKKG

>Takifugu rubripes Sp3

STQLTGTDRWEVLTPTSEKDESGMIQIQSQGIFTSNGQYVLPLQNLQSQSVFVTSGTDTS

SATSVPNIQYQVIPQIQTADGQLSFSTSNMEGAGLAQDATGQIHILPDGSQTLNVSSAAN

ILNNQNLISQTGNVQQIQGVSIGSSTFNNQGQVVTNVPVGLPGNITFVPINSVDLDSLGL

SGAQTIATGVTADGQLIMANQPVDGSQNLVKTDNHLSQTLPVNANANREIYVPTSSSQSQ

LHIPVSESALLTQDTSLSSVTTAQTNSSSGLQDGFIQENQDQNIQVSSAQPIIQLQQVPI

QTTNGQVIQSVTPGGQGLQNVQLINPGTFIIQAQTVTPSGQIQWQTFQVQGVQNLQNLQL

PTTPHQQITLAPVQTLSLGSTPVSIGTGQIPNLQTVTVNSVALNEGEADTLGDIQIKEEP

DWQLSTDSTLNTSDLSHLRGRLVDEDDQIGQEGKRLRRVACTCPNCKESGGRGSSTGKKK

QHICHIAGCGKVYGKTSHLRAHLRWHSGERPFVCSWMLCGKRFTRSDELQRHRRTHTGEK

KFVCPECSKRFMRSDHLAKHIKTHQNKKGMNSGGSVVASMESTGSSDSIIATAGGTTLIL

TNIQQGSSNAQDILANAEIPLQLVTTVAASEVME

>Homo sapiens SP8

APGLLGEEPRLGSTPLAMLAATCNKIGSPSPSPSSLSDSSSSFGKGFHPWKRSSSSSSAS

CNVVGSSLSSFGVSGASRNGGSSSAAAAAAAAAAAAAALVSDSFSCGGSPGSSAFSLTSS

SAAAAAAAAAAAASSSPFANDYSDGSHQPVFISKVHTSVDGLQGIYPRVGMAHPYESWFK

PSHPGLGAAGEVGSAGASSWWDVGAGWIDVQNPNSAAALPGSLHPAAGGLQTSLHSPLGG

YNSDYSGLSHSAFSSGASSHLLSPAGQHLMDGFKPVLPGSYPDSAPSPLAGAGGSMLSAG

PSAPLGGSPRSSARRYSGRATCDCPNCQEAERLGPAGASLRRKGLHSCHIPGCGKVYGKT

SHLKAHLRWHTGERPFVCNWLFCGKRFTRSDELQRHLRTHTGEKRFACPVCNKRFMRSDH

LSKHVKTHSGGGGGGGSAGSGSGGKKGSDTDSEHSAAGSPPCHSPELLQPPEPGHRNGLE

>Mus musculus Sp8

MLAATCNKIGSPSPSPSSLSDSSSSFGKGFHPWKRSSSSSSGSCNVVGSSLSSFGVSGAS

RNGGSSSAAAAAAAAAAAAAALVSDSFSCGGSPGSSAFSLTSSSAAAAAAAAAAAASSSP

FANDYSVFQAPGVSGGSGGGGGGGGGGSGAHSQDSSHQPVFISKVHTSVDGLQGIYPRVG

MAHPYESWFKPSHPGLGAAADVGSAGASSWWDVGAGWIDVQNPNGAAALPGSLHPAAGGL

QTSLHSPLGGYNSDYSGLSHSAFSSGASSHLLSPAGQHLMDGFKPVLPGSYPDSAPSPLA

GAGSSMLSAGPAAQLGGSPRSSARRYSGRATCDCPNCQEAERLGPAGASLRRKGLHSCHI

PGCGKVYGKTSHLKAHLRWHTGERPFVCNWLFCGKRFTRSDELQRHLRTHTGEKRFACPV

CNKRFMRSDHLSKHVKTHSGGGGSAGSGGGKKGSDTDSEHSAAGSPPCHSPELLQPPEPG

HRNGLE

> Rattus norvegicus Sp8

MATSLLGEEPRLGSTPLAMLAATCNKIGSPSPSPSSLSDSSSSFSKGFHPWKRSSSSSSG

SCNVVGSSLSSFGVSGASRNGGSSSAAAAAAAAAAAAAALVSDSFSCGGSPGSSAFSLTS

SSAAAAAAAAAAAASSSPFANDYSVFQAPGVSGGSGGGGGGGGGGSGAHSQDSSHQPVFI

SKVHTSVDGLQGIYPRVGMAHPYESWFKPSHPGLGAAADVGSAGASSWWDVGAGWIDVQN

PNGAAALPGSLHPAAGGLQTSLHSPLGGYNSDYSGLSHSAFRGSPRSSARRYSGRATCDC

PNCQEAERLGPAGASLRRKGLHSCHIPGCGKVYGKTSHLKAHLRWHTGERPFVCNWLFCG

KRFTRSDELQRHLRTHTGEKRFACPVCNKRFMRSDHLSKHVKTHSGGGGSAGSGGGKKGS

DTDSEHSAAGSPPCHSPELLQPPEPGHRNGLE

>Xenopus tropicalis Sp8

MLAATCNKIGSPSPSPSSLSDSSASFGKGFHPWKRSSSTSSSCTLASGSLSGFSVGGSSR

GGNGSSSAAAAAAAAAAAAAAAALVSDSFSCGGSPGSSAFSLTSGVSGGGGGGGGSSGSV

SAASPFANEYSVFPVSGGSQEAAAAAAAAAAAAAAAASHHQPVFLSKVHASVDGLQSIYP

RVGMAHPYESWFKPSHPGEVSAAAAGGASSWWEVGAGWIDVQSPGAGAALHPGALQGSLH

SPLGGYGTDYSAAGLGHFSAGGGGGAGSGGGAAGAGNGGGGGPGSHLLSSGGQHIMDGFK

PVIGPGSYPDPSSSPLSAGSMLAPTGPLGGSPRSSARRYSGRATCDCPNCQEAERLGPAG

ASLRRKGLHSCHIPGCGKVYGKTSHLKAHLRWHTGERPFVCNWLFCGKRFTRSDELQRHL

RTHTGEKRFACPVCNKRFMRSDHLSKHVKTHSAGGAGGGAGSTGPGSGSKKGSDTDSEHS

PSTSPPCHSPDLLHPPDRNGLE

>Tetraodon nigroviridis SP8

EEPRLGSTPLALLAAACSRIGDPSPSCSPALSEPGLVKGFHPWKGGSGGTSNPGPFGVSS

ANCRNNGSGLTNTSSAFSVTTVNSFVGNNYSMYQTSLSSDNIISDSAHTQRSAFLTKFPP

AVEDIAGIYPRVPGHQSWFKPVGEAGSGTSSWWDIGTSWVDAPSPAGLPASLGVCNAEYS

SAFSPGAPQHLLPATQHLFDGFRQPVLAPYADPSAMGAPAAAAAATNPVSLPASSRSSSR

RYSGRATCDCPNCREAESPGQGGASPRRRGLHSCHIPGCGKVYGKTSHLKAHLRWHTGER

PFVCNWLFCGKRFTRSDELQRHLRTHTGEKRFECAVCQKRFMRSDHLSKHVRTHSADGSE

DSGEAGARGGSDTDHSPPGSPAAPPAAPERLPTTGRQN

>Takifugu rubripes SP8

EEPRLGSTPLAMLAATCSRIGDPGPSCSPALSDGAPGLVKGFHPWKGGLGGSSNLGAFGV

PPGNCRNNGTGLANTNSAFSVTTVNSLMGNNYSMYQTSVSPDNIIPDSAHSQRSAFLKKF

PPSVEDLTGIYPRVHGHQSWFKPMGDTGNGTSAWWDIGTSWMDAQSPTGIPASFGACNTE

YSSPFSPSTSQHLLPAAQHLFDGFRPSVPAPYADTTKIGAPVVATNPVPLPASSRSSSRR

YSGRATCDCPNCQEAESLGPGGASPQRRGLHSCHIPGCGKVYGKTSHLKAHLRWHTGERP

FVCNWLFCGKRFTRSDELQRHLRTHTGEKRFECAICQKRFMRSDHLSKHIRTHTADGSED

SGETGHGKGSSDTDTSLSGSPASQSA

>Homo sapiens Sp2

MAATAAVSPSDYLQPAASTTQDSQPSPLALLAATCSKIGPPAVEAAVTPPAPPQPTPRKL

VPIKPAPLPLSPGKNSFGILSSKGNILQIQGSQLSASYPGGQLVFAIQNPTMINKGTRSN

ANIQYQAVPQIQASNSQTIQVQPNLTNQIQIIPGTNQAIITPSPSSHKPVPIKPAPIQKS

STTTTPVQSGANVVKLTGGGGNVTLTLPVNNLVNASDTGAPTQLLTESPPTPLSKTNKKA

RKKSLPASQPPVAVAEQVETVLIETTADNIIQAGNNLLIVQSPGGGQPAVVQQVQVVPPK

AEQQQVVQIPQQALRVVQAASATLPTVPQKPSQNFQIQAAEPTPTQVYIRTPSGEVQTVL

VQDSPPATAAATSNTTCSSPASRAPHLSGTSKKHSAAILRKERPLPKIAPAGSIISLNAA

QLAAAAQAMQTININGVQVQGVPVTITNTGGQQQLTVQNVSGNNLTISGLSPTQIQLQME

QALAGETQPGEKRRRMACTCPNCKDGEKRSGEQGKKKHVCHIPDCGKTFRKTSLLRAHVR

LHTGERPFVCNWFFCGKRFTRSDELQRHARTHTGDKRFECAQCQKRFMRSDHLTKHYKTH

LVTKNL

>Mus musculus Sp2

EAAEAREEDVVMSDPQMSMAATAAVSPSDYLQPAAATTQAKQPSPLALLAATCSKIGPPA

VEAAVTPPAPPQPTPRKLVPIKPAPLPLSPCKNSFSILSSKGNILQIQGSQLSTSYPGGQ

FVFAIQNPTLINKGSRSNASIQYQVPQIQGNSSQTIQVQPSLTNQIQVIPGTNQAITTPS

TSGHKPVPIKPAPVQKSSTTTTPVQSGANVVKLTGGGSNMTLTLPLNNLVNTSDIGGPAQ

LLTESPPTPLSKTNKKARKKSLPVSQPSVAVAEQVETVLIETTADNIIQAGNNLLIVQSP

GGGQPAVVQQVQVVPPKAEQQQVVQIPQQALRVVQAASATLPTVPQKPSQNFQIQTTEPT

PTQVYIRTPSGEVQTVLVQDSPPATAATTSTVTCNSPALRAPHLSGTSKKHSAAILRKER

PLPKIAPAGSIISLNAAQLAAAAQAMQTININGVQVQGVPVTITNTGGQQQLTVQNVSGN

NLTISGLSPTQIQLQMEQALAGEAQPGEKRRRMACTCPNCKDGEKRSGEQGKKKHVCHIP

DCGKTFRKTSLLRAHVRLHTGERPFVCNWFFCGKRFTRSDELQRHARTHTGDKRFECAQC

QKRFMRSDHLTKHYKTHLGTKGL

>Rattus norvegicus Sp2

EAAEAREEDVVMSDSQMSMAATAAVSPSDYLQPAAAATQAIQPSPLALLAATCSKIGPPA

VEAAVTPPAPPQPTPRKLVPIKPAPLPLSPCKNSFSILSSKGNILQIQGSQLSASYPGGQ

FVFAIQNPTLINKGSRSNASIQYQVPQIQGNSSQTIQVQPNLTNQIQIIPGTNQAIITPS

TSGHKPVPIKPAPVQKSNTTTTPVQSGANVVKLTGGGGNMTLTLPLNNLVNTSDIGAPAQ

LLTESPPTPLSKTNKKARKKSLPVSQPSVAVAEQVETVLIETTAENIIQAGNNLLIVQSP

GGGQPAVVQQVQVVPPKAEQQQVVQIPQQALRVVQAASATLPTVPQKPSQNFQIQTTEPT

PTQVYIRTPSGEVQTVLVQDSPPATAATTSTVSCSSPALRAPHLSGSSKKHSAAILRKER

PLPKIAPAGSIISLNAAQLAAAAQAMQTININGVQVQGVPVTITNTGGQQQLTVQNVSGN

NLTISGLSPTQIQLQMEQALAGEAQPGEKRRRMACTCPNCKDGEKRSGEQGKKKHVCHIP

DCGKTFRKTSLLRAHVRLHTGERPFVCNWFFCGKRFTRSDELQRHARTHTGDKRFECAQC

QKRFMRSDHLTKHYKTHLGTKGL

>Gallus gallus Sp2

SALTADLTPVFLLPPCRLSDSMAATAAVSPSEYLQPAASTAQDSQPSPLALLAATCSKIG

PPAVEAAVTPPAPPQPTPRKLVPIKPAPLPLGSGKNSFGILSSKGNLFQIQGSQVGTSYP

GGQLVFAIQNPTVISKGTRSSANIQYQAVPQIQAAGGQTIQVQPNLTNQIQIIPGTNQAI

LTPSSSSHKPVPIKPAPAQKSGASPVQGSSNVVKLTGGSNVTLTLPMNNLVNTTESSTQA

QVIAESPSKPGKKTRKKAMSPSQPSTVAVAEQVETVLIETTAENIIQAGNNLLIVQSPGS

GQPAVVQQVQVVQPKQESQVVQIPQQALRVVQAASATLPTVPQKSSQNIQIQTTESTPTQ

VYFKTPSGELQTVLLQEAPAVTVAPSSTSCSSPVSRNSGTVTSSKKPTARKERPLPKIAP

AGGVISLSAAQLAAAAQAMQTININGVQVQGVPVTITNAGGQQQLTVQNVSGNNLTISGL

SPTQIQLQMEQALSGEMQPGEKRRRMACTCPNCKDGEKRPGDQGKKKHICHIPECGRTFR

KTSLLRAHVRLHTGERPFVCNWVFCGKRFTRSDELQRHARTHTGDKRFECAQCQKRFMRS

DHLTKHYKTHLITKNL

>Tetraodon nigroviridis Sp2

EQQDSMAATAAVSPSEYLQPSTASSQDAQPSPFGLLAATCSKIGPPAAQAPVTSAPEQPQ

PRRLLPIKPAPIAPAPPKNLGFLSAKGNVIQLPAGLTPTTPGSPIVLTIQQSPARANNPT

PTNIQYQVVPQIQGAQTIQVMPQAGQIQLIPGTNHAIITTPMTIPAPTAATTSVTPQKTV

AIKPSPKPRKADTAAAGNVVQLPGGLTLPLNVATGEVGGAQVVTETASAPPIPVKGRRGR

KKKVAVAAQPPPPTPPPPPPPPPPPQPASPSSGPMETIVIEAGDNIIQAGNNLLIVQSPG

QPAVVQQVQVVQPKPDSQVVQIPQQALKVVQAASATLPPVPQKQALPQSLQVAQTEPSPT

QVSLSLSFSLSLFLFTPPHRVSSRVPNVPLIFISLRLFSKQHRGSGRRFSSRTPSPRRCL

LAAQAVQTISINGVHVVGLPVTITNTGRNPTWCLTGQQHLTVQTMQGGGLQLAATAGQPT

LQVDQTLTLELPSQPGEKKRRMACTCPNCKDADKRPGEVGKRKHICHVPGCEKTFRKTSL

LRAHVRLHTGERPFVCSWVFCGKRFTRSDELQRHARTHTGDKRFECSQCQKRFMRSDHLT

KHYKTHINTKNL

>Danio rerio Sp2

MDCFAEFLCVDIGWNSAWTLPGAQLPASLPADANEHQPTGISSLTNIGQREIRGVLEYCL

YQKDSMATTVAVSPSEYLQPSTTTSQDSQPSPLALLAATCSKIGPPAAQAPVSTPPSQPT

TRRLHPIKPAPIAPAPPKNLGFLSAKGNIIQLPAGLGSSGASPIVFTFQSPSRPAGTSTA

NFQYQVIPQFQGSQTIQMMPQGGQIQIIPGTNQAIITSPVTVQAATPTAPPLAPAPVPQH

KTVAIKPSTQKRRQNNASVNANIVRLPSGLTLPLNVTTGDVGGAQVLTETAAAPVKPKRG

RKRQVAIAAPAPAPQPASPPPVAEQVEALLIETTADNIIQAGNNLLIVQNPGGSQPAVVQ

QVQLLQQKSDQQVVQIPPQALKVVQAASATLPPVPQRQTVTPSVQVSPPEPTQVLIKTAS

GEWQAVQIQETTVTTPTTPTSTPTPAVVTKKVQTGTRKERTLPKIAPAGGGLITLNAAQL

ASAAQAVQTININGVQVQGVPVTITNAGGQQHLTVQTVPSAGLQLGSVQTQTQTMQIEQT

QTLALELQTQPGEKKRRMACTCPNCKDAEKKPGEVGKRKHICHIAGCEKTFRKTSLLRAH

VRLHTGERPFVCNWVFCGKRFTRSDELQRHARTHTGDKRFECNKCQKRFMRSDHLTKHYK

THINTKSL

>Strongylocentrotus(XP_793203)

MGTPETNVIRSVLMWCPDRAIDLEPSRKGRPPLAMLAATCDRVAEGSPESFPDPPKAKGF

HPFKRTTPTMSGLTAEHAASRFAHMAAPGSMLGASLPYPLAPSSSLTSPFSGELFFSRKT

EPRIPIATETAKSNTSPYGHKTGFSSETEHLAGILPPHLCGHSAYESWLKAATSASMLSR

ENGVHGFSPAFWSLPGAQASLLDLHSSAGTLPSHFGKMPTCSSASDCSALALSHSVLPYP

PLTTTNPHLFSPSQLFPPSSYNSFLPPGYDIMSLTEMRSSFFGPAGLGLSLSRAGRRYSG

RATCDCPNCQENERLAAAGQPLRKKNVHSCHIPGCGKIYGKTSHLKAHLRWHTGERPFVC

NWLFCGKRFTRSDELQRHLRTHTGEKRFSCPTCNKRFMRSDHLSKHVKTHQNNNNNSGND

NGGNADSKAAGETGKMSPIKKLASVGSSEAESSPSGTEGSPSPVSTGPGPISMSVPQSAA

LKVASP

>Strongylocentrotus(XP_784359)

MAKKSTKKGQSNYIQPSSMSQETQPSPLALLAATCSKIGTPAEGQAGAVNQGQTVTVLGQ

NQGQAVQIPGGFINAANAQQIQQALGLPPGFPLQFTTASTGVAGQQGTAAGGPMYIEVGP

GGNIPSSSVGGATPTKSINAANILSLNQQSATGGQQIVTNGNVQYNITPQYQIDSEGNLI

TTHVATPVSVSQAQAQTTQTVVRQAPTTTSQATNVAGSNVAQIALPGAGVQYIQNGQIIQ

AVAPQPAPTQQRIMLGSQTITLQLAPNAVMSNANSHDQPVVTPVYNIISSTPPQGNQDNS

AQTQQLQIQDQLQNAQIISNSGQIQAGNAANSNQATFVQVAGKPGQIILQQPQQAGQVQQ

IQVSGLQTSNTNVQTTQIRQQSGKVVASVIPQQTVQQQPQAQVIQIHQPIQGQAGTQISL

QQQPGTGYYTIQQPTPQPQQQAQTITLPVNVAGSQGSVQTITLPIQGNQLQGGSITIPQS

VLQSIQQINGGQFMQNPIVLKAPQTQVQTVHLQHGGTPVATPQSSSSQVQQQVITTDVSP

AISTANNSTIAGLPNYNVHLAPLSPGPGPAGNTSAVNINTSTAATQQQITQQQQQQISQA

LVGMKAEKQQQASWQGVIQTEATGGAQGGTVTTISTNGTNYPPTMASYELQIDQSQIPKQ

EPPKKVRRLACTCPNCKDGDGRNSEKGKKQHICHIADCGKIYGKTSHLRAHLRWHTGERP

FVCDWLFCGKRFTRSDELQRHRRTHTGEKKFVCKSCGKKFMRSDHLAKHQRTHIRKPGTV

SMKGQSGGGDAPLQVDLSQGVDEFDEEMEKVMRDPQHEAMVQDAVQEAVY

**Zinc finger protein subfamily 1A**

>Homo sapiens ZNFN1A1

MDADEGQDMSQVSGKESPPVSDTPDEGDEPMPIPEDLSTTSGGQQSSKSDRVVASNVKVE

TQSDEENGRACEMNGEECAEDLRMLDASGEKMNGSHRDQGSSALSGVGGIRLPNGKLKCD

ICGIICIGPNVLMVHKRSHTGERPFQCNQCGASFTQKGNLLRHIKLHSGEKPFKCHLCNY

ACRRRDALTGHLRTHSVGKPHKCGYCGRSYKQRSSLEEHKERCHNYLESMGLPGTLYPVI

KEETNHSEMAEDLCKIGSERSLVLDRLASNVAKRKSSMPQKFLGDKGLSDTPYDSSASYE

KENEMMKSHVMDQAINNAINYLGAESLRPLVQTPPGGSEVVPVISPMYQLHKPLAEGTPR

SNHSAQDSAVENLLLLSKAKLVPSEREASPSNSCQDSTDTESNNEEQRSGLIYLTNHIAP

HARNGLSLKEEHRAYDLLRAASENSQDALRVVSTSGEQMKVYKCEHCRVLFLDHVMYTIH

MGCHGFRDPFECNMCGYHSQDRYEFSSHITRGEHRFHMS

>Mus musculus ZNFN1A1

MDVDEGQDMSQVSGKESPPVSDTPDEGDEPMPVPEDLSTTSGAQQNSKSDRGMGERPFQC

NQCGASFTQKGNLLRHIKLHSGEKPFKCHLCNYACRRRDALTGHLRTHSVGKPHKCGYCG

RSYKQRSSLEEHKERCHNYLESMGLPGMYPVIKEETNHNEMAEDLCKIGAERSLVLDRLA

SNVAKRKSSMPQKFLGDKCLSDMPYDSANYEKEDMMTSHVMDQAINNAINYLGAESLRPL

VQTPPGSSEVVPVISSMYQLHKPPSDGPPRSNHSAQDAVDNLLLLSKAKSVSSEREASPS

NSCQDSTDTESNAEEQRSGLIYLTNHINPHARNGLALKEEQRAYEVLRAASENSQDAFRV

VSTSGEQLKVYKCEHCRVLFLDHVMYTIHMGCHGFRDPFECNMCGYHSQDRYEFSSHITR

GEHRYHLS

>Rattus norvegicus ZNFN1A1

MDVDEGQDMSQVSGKESPPVSDTPDEGDEPMPVPEDLSTSGAQQNSKSDRGMGERPFQCN

QCGASFTQKGNLLRHIKLHSGEKPFKCHLCNYACRRRDALTGHLRTHSVGKPHKCGYCGR

SYKQRSSLEEHKERCHNYLESMGLPGMYPVIKEETNHSEMAEDLCKIGAERSLVLDRLAS

NVAKRKSSMPQKFLGDKCLSDMPYDSANYEKDEMMTSHVMDQAINNAINYLGAESLRPLV

QTPPGSSEVVPVISSMYQLHKPPSDGPPRSNHSAQDSAVENLLLLSKAKSVSSEREASPS

NSCQDSTDTESNAEEQRSGLIYLTNHITPHARNGLALKEEQRAYEVLRAASENSQDAFRV

VSTSGEQLKVYKCEHCRVLFLDHVMYTIHMGCHGFRDPFECNMCGYHSQDRYEFSSHITR

GEHRYHLS

>Xenopus tropicalis ZNFN1A1

METDEAQDITQMSGNQSPAMSDGLDDPDEPMPVPEDLSTNAASQQNSKNEKSIAGNIKME

NHSDEENGRACEMNGEECAEDLRMLDSAGEKINGSLNGQGTKALTGVGGERPFQCNQCGA

SFTQKGNLLRHIKLHSGEKPFKCHMCNYACRRRDALTGHLRTHSVGKPHKCGYCGRSYKQ

RSSLEEHKERCHNYLQSMGLQSHLYAVKEESNQNDMAEDLSKIGSERSLVLDRLASNVAK

RKSCMPQKFVGEKRLSDIPYDHNANYEKENEMMQTHVMDQAINNAITYLGAESLRPLVQT

PPGGSEGVPINSMYALHKPHADITTNTSQSAHESAVENLLLLSQVKSISSEREVSPSNSC

QDSTDTESNNEERGGLIYLTNHMTPHPRNGLSTKEHRPFEMVRASSDNSQDAFKVVSGSG

ESLKVHKCEHCRVIFLDHVMYTIHMGCHGFRDPFECNMCGYQSQDRYEFSSHITRGEHRF

NMG

>Canis familiaris ZNFN1A1

MDADEGQDMSRVSGKESPPVSDTPDDGDEPMPVPEDLSTTSGGQQTSKNERGVGERPFQC

NQCGASFTQKGNLLRHIKLHSGEKPFKCHLCNYACRRRDALTGHLRTHSVGKPHKCGYCG

RSYKQRSSLEEHKERCHNYLQSMGLPGTMYPVIKEEANHSEMGEDLCKIGSERSLVLDRI

ASNVAKRKSSMPQKFVGDKCLSDMPYDSSASYEKENEMMQTHVMDQAINNAISYLGAESL

RPLVQTPPGSSEVVPVISPMYQLHKPHAEGPPRSNHSAQDSAVENLLLLSKAKSVSSERE

ASPSNSCQDSTDTESNNEEQRSSLIYLTNHINPHARNGLSIKEEHPAYDVLRAASESNQD

AFRVIGTSGEPMKVYKCEHCRVLFLDHVMYTIHMGCHGFRDPFECNMCGYHSQDRYEFSS

HITRGEHRFHMS

>Bos taurus ZNFN1A1

MDADEGQDMSQVSGKESPPVSDTPDDSDEPMPVPEDLSTTSGGQQSSKSERGLAGNVKVE

TQSDEENGRACEVNGEECAEDLRMLDASGEKMNGSHSVQGSKALSGAGGIRLPNGKLKCD

VCGIICIGPNVLMVHKRSHTGERPFQCNQCGASFTQKGNLLRHIKLHSGEKPFKCHLCNY

ACRRRDALTGHLRTHSVGKPHKCGYCGRSYKQRSSLEEHKERCHNYLQSMGLPGTLYPVI

KEETNHSEMAEDLCKMGSDRSLVLDRLASNVAKRKSSMPQKFVGDKCLSELPYDGSASYE

KENEMMQTHVMDQAINNAISYLGAESLRPLVQTPPGSSDVVPVLSPMYQLHKPHGEGPAR

SNHSAQDGAVENLLLLSKAKSASSEREASPSNSCQDSTDTESNTEEQRGGLIYLTNHINP

HARNGLSIKEEHAAYDVLRAASEGPPDALRVIGTSGEALKVYRCEHCRVLFLDHVMYTIH

MGCHGFRDPFECNMCGYHSQDRYEFSSHITRGEHRFHMS

>Tetraodon nigroviridis ZNFN1A1

SAGGTRLPNGKLQCDICGIVCIGPNVLMVHKRSHTGERPFQCSQCGASFTQKGNLLRHIK

LHSGEKPFKCHLCNYACRRRDALTGHLRTHSVGKPHKCAYCGRSYKQRSSLEEHKERCHN

YLHYMGLQNSIYTVMKEESNHNEQREDLSQMGSDRALVLDRLANNVAKRKSTMPQKFVGD

KRLSDLSYDGGAGELTQPQVIDQTINSAISYLEAESLRPLVQTSPASSSDVGLSSMFPLH

KTASEGQTGTSMSAKDSAAENLLLLSNSKSACSEKDGSPSHSGQDSTDTGEQQRRPPRCN

APGLIYLTNHITSRMRNGVMPLVKEEQQRQYEAIQASIEMASEGFKVTTADGEQVRAYRC

EHCRVLFLDHVMYTIHMGCHGFRDPFECNLCGHRSQDRYEFSSHITRGEHRL

>Takifugu rubripes ZNFN1A1

METEEAQEMTQMPGRDSPHGNGATEEAEEPMAVPEDLSANSIHQQNNRADKVCNIKVEAR

SDEENGLASDMNGVEEEECAEDLRVIDASGAKVNGSQSSPQAKAFSSAGGTRLPNGKLQC

DICGIVCIGPNVLMVHKRSHTGERPFHCNQCGASFTQKGNLLRHIKLHSGEKPFKCHLCS

YACRRRDALTGHLRTHSVGKPHKCAYCGRSYKQRSSLEEHKERCHNYLHYMGLQNSIYTV

IKEESNQNEQREDLSQTGSDRALVLDRLANNVAKRKSTMPQKFVGDKRLSDLSYDGGAGE

LTQPQGIDQTINSAISYLEAESLRPLVQTSPASSSDVALSSMFSLHKTASDGQGGTVMSA

KDSAAENLLLLSKSACSEKDGSPSHSGQDSTDTESNNDDRPGVTAPGLIYLTNHITSRMR

NGVLPLVKEEQQRQYEAIQASMEMASEGFKVITTDGEQVRAYRCEHCRVLFLDHVMYTIH

MGCHGFRDPFECNLCGHRSQDRYEFSSHITRGEHRF

>Danio rerio ZNFN1A1

METEEAQEMSQITGRDSPMNANEGGEDQDEAMPVPEDLSASTGLQHNNRTDKPLACNIKV

EARSDEENGLSCEMNGEAEECAAEDLRILDGSGAKVNGSHAGPDSKPAAYPTAGGIRLPN

GKLKCDICGIVCIGPNVLMVHKRSHTEERKSVLEQQKGERPFQCNQCGASFTQKGNLLRH

IKLHSGEKPFKCHLCNYACRRRDALTGHLRTHSVGKPHKCAYCGRSYKQRSSLEEHKERC

HNYLQCMGLQNSIYTVKEENSQNEQREDMPASERALVLDRIANNVAKRKSSMPQRFVGEN

RLSELSFESGSGELMQPHVIDQAINSAISYLGAESLRPLVQTSPGSADMVVSPLYNLHKS

QTAEGNGVSAKDSAAEHLLLLSKSKSASVDKDGSPSPSGQDSTDTESNNEERSAGVSGTA

ATGGLIYLTNHMAPGMRNGGLPGVKEEQHRQFEALRAAGMDLSIASSEGFKVLSGDGEEL

RAYRCIHCRVLFLDHVMYTIHMGCHGFRDPFECNLCGYRSQDRYEFSSHITRGEHRI

>Homo sapiens ZNFN1A2

METEAIDGYITCDNELSPEREHSNMAIDLTSSTPNGQHASPSHMTSTNSVKLEMQSDEEC

DRKPLSREDEIRGHDEGSSLEEPLIESSEVADNRKVQELQGEGGIRLPNGKLKCDVCGMV

CIGPNVLMVHKRSHTGERPFHCNQCGASFTQKGNLLRHIKLHSGEKPFKCPFCSYACRRR

DALTGHLRTHSVGKPHKCNYCGRSYKQRSSLEEHKERCHNYLQNVSMEAAGQVMSHHVPP

MEDCKEQEPIMDNNISLVPFERPAVIEKLTGNMGKRKSSTPQKFVGEKLMRFSYPDIHFD

MNLTYEKEAELMQSHMMDQAINNAITYLGAEALHPLMQHPPSTIAEVAPVISSAYSQVYH

PNRIERPISRETADSHENNMDGPISLIRPKSRPQEREASPSNSCLDSTDSESSHDDHQSY

QGHPALNPKRKQSPAYMKEDVKALDTTKAPKGSLKDIYKVFNGEGEQIRAFKCEHCRVLF

LDHVMYTIHMGCHGYRDPLECNICGYRSQDRYEFSSHIVRGEHTFH

>Mus musculus ZNFN1A2

METDAIDGYITCDNELSPEGEHANMAIDLTSSTPNGQHASPSHMTSTNSVKLEMQSDEEC

DRQPLSREDEIRGHDEGSSLEEPLIESSEVADNRKVQDLQGEGGIRLPNGKLKCDVCGMV

CIGPNVLMVHKRSHTGERPFHCNQCGASFTQKGNLLRHIKLHSGEKPFKCPFCSYACRRR

DALTGHLRTHSVGKPHKCNYCGRSYKQRSSLEEHKERCHNYLQNVSMEAAGQVMSHHVPP

MEDCKEQEPIMDNNISLVPFERPAVIEKLTANMGKRKSSTPQKFVGEKLMRFSYPDIHFD

MNLTYEKEAELMQSHMMDQAINNAITYLGAEALHPLMQHAPSTIAEVAPVISSAYSQVYH

PNRIERPISRETSDSHENNMDGPISLIRPKSRPQEREASPSNSCLDSTDSESSHDDRQSY

QGNPALNPKRKQSPAYMKEDVKALDATKAPKGSLKDIYKVFNGEGEQIRAFKCEHCRVLF

LDHVMYTIHMGCHGYRDPLECNICGYRSQDRYEFSSHIVRGEHTFH

>Rattus norvegicus ZNFN1A2

NSVKLEMQSDEECDRQPLSREDEIRGHDEGSSLEDPLIESGEVADNRKVQDLQGEGGIRL

PNGKLKCDVCGMVCIGPNVLMVHKRSHTGERPFHCNQCGASFTQKGNLLRHIKLHSGEKP

FKCPFCSYACRRRDALTGHLRTHSVGKPHKCNYCGRSYKQRSSLEEHKERCHNYLQNVSM

EAAGQVMSHHVPPMEDCKEQEPIMDNNISLVPFERPAVIEKLTANMGKRKSSTPQKFVGE

KLMRFSYPDIHFDMNLTYEKEAELMQSHMMDQAINNAITYLGAEALHPLMQHAPSTIAEV

APVISSAYSQVYHPNRIERPISRETADSHENNMDGPISLIRPKSRPQEREASPSNSCLDS

TDSESSHDDRQSYQGNPALNPKRKQSPPYMKEDVKALDATKAPKGSLKDIYKVFNGEGEQ

IRAFKCEHCRVLFLDHVMYTIHMGCHGYRDPLECNICGYRSQDRYEFSSHIVRGEHTFH

>Xenopus tropicalis ZNFN1A2

MSQNQACERERQFSQNLIQTGTPGALHLKKLKAWNSAGDPDISTEKTEQPKNLCTSTMES

ERSDGYVTSDNEIVPETEHSNMAIDLTSCTPNGQHSSPTHMASTNSVKMEMQSDDEGEGK

PLRRDTPLQLQDDESGLEEPLIDANGMSDSRSSQDMPLDGGIRLPNGKLKCDVCGMVCIG

PNVLMVHKRSHTGERPFHCNQCGASFTQKGNLLRHIKLHSGEKPFKCPFCSYACRRRDAL

TGHLRTHSVGKPHKCNYCGRSYKQRSSLEEHKERCHNYLQNVGMEAGQGIVHHASPMDEC

KNPETMMENNMSLIPFERPAVIERLASNMGKRKSSTPQKFVGEKLMRFGYPDLHFEMAYE

KEAEIMQSQMMDQAINNAITYLGADALRPLIHHSAAAMPDVPPIVSSLYSQVYHPARVER

PTSRETSDSNDNNMDGPISLIRPKNHPQEREASPSNSCLDTTDSESSHEDRQSYQGNHAL

NSKRKQSPVYPKEDGKALDATKSSLG

>Tetraodon nigroviridis ZNFN1A2

GDRPFQCNQCGVSFTQKGNLLRHIKLHTGEKPFKCPFCSYACRRRDALTGHLRTHAVGKP

HKCNYCGRSYKQRTSLEEHKERCHNYLRGISLDPNINAGPYPGEVAMESRPMAEPNSLVT

FDRPPVIERLNTVGKRKSTPPQKFMGDKMLRYSYPELAYGMGLKYEKQSEMLPAHMDQAI

SNTSIPYMLSETLRPMLHHPGPPLSIAEMSSMVNPLFHHVLPLGQRAECPGSGGTLPPQP

HDVHGQPTANGPAALTRLIKLPRKGQEESPNNSGLDSDSARSSPQERQAYHGTNLPSGLR

SQASPAVASADERVLESSPRSGTGAGNGSIRATTERSTSQEGVRVFGREGHELRAFQCEH

CQVLFLDHVMYTIHMGCHGYRNPLECNICGHHSKDRYEFSSHIVRGEHTF

>Homo sapiens ZNFN1A3

MEDIQTNAELKSTQEQSVPAESAAVLNDYSLTKSHEMENVDSGEGPANEDEDIGDDSMKV

KDEYSERDENVLKSEPMGNAEEPEIPYSYSREYNEYENIKLERHVVSFDSSRPTSGKMNC

DVCGLSCISFNVLMVHKRSHTGERPFQCNQCGASFTQKGNLLRHIKLHTGEKPFKCHLCN

YACQRRDALTGHLRTHSVEKPYKCEFCGRSYKQRSSLEEHKERCRTFLQSTDPGDTASAE

ARHIKAEMGSERALVLDRLASNVAKRKSSMPQKFIGEKRHCFDVNYNSSYMYEKESELIQ

TRMMDQAINNAISYLGAEALRPLVQTPPAPTSEMVPVISSMYPIALTRAEMSNGAPQELE

KKSIHLPEKSVPSERGLSPNNSGHDSTDTDSNHEERQNHIYQQNHMVLSRARNGMPLLKE

VPRSYELLKPPPICPRDSVKVINKEGEVMDVYRCDHCRVLFLDYVMFTIHMGCHGFRDPF

ECNMCGYRSHDRYEFSSHIARGEHRALLK

>Mus musculus ZNFN1A3

FLDIQPTVELKSTEEQPLPTESPDALNDYSLPKPHEIENVDSREAPANEDEDAGEDSMKV

KDEYSDRDENIMKPEPMGDAEESEMPYSYAREYSDYESIKLERHVPYDNSRPTGGKMNCD

VCGLSCISFNVLMVHKRSHTGERPFQCNQCGASFTQKGNLLRHIKLHTGEKPFKCHLCNY

ACQRRDALTGHLRTHSVEKPYKCEFCGRSYKQRSSLEEHKERCRAFLQNPDLGDAASVEA

RHIKAEMGSERALVLDRLASNVAKRKSSMPQKFIGEKRHCFDANYNPGYMYEKENEMMQT

RMMDQAINNAISYLGAEALRPLVQTPPAPTSEMVPVISSVYPIALTRADMPNGAPQEMEK

KRILLPEKILPSERGLSPNNSAQDSTDTDSNHEDRQHLYQQSHVVLPQARNGMPLLKEVP

RSFELLKPPPICLRDSIKVINKEGEVMDVFRCDHCHVLFLDYVMFTIHMGCHGFRDPFEC

NMCGYRSHDRYEFSSHIARGEHRAMLK

>Rattus norvegicus ZNFN1A3

MKVKDEYSERDENILKPEPMVDAEESEPPYSYAREYSDYENIKLERHGPYDSSRPTSGKM

NCDVCGLSCISFNVLMVHKRSHTGERPFQCNQCGASFTQKGNLLRHIKLHTGEKPFKCHL

CNYACQRRDALTGHLRTHSVEKPYKCEFCGRSYKQRSSLEEHKERCRAFLQNPDLGDAAS

VEARHIKAEMGSERALVLDRLASNVAKRKSSMPQKFIGEKRHCFDANYNPGYMYEKENEM

MQTRMMDQAINNAISYLGAEALRPLVQTPPAPTSEMVPVISSVYPIALTRADMPNGAPQE

LEKKRILLPEKILPSERGLSPNNSAQDSTDTDSNHEDRQNHIYQQGHMVLPQARNGMPLL

KEVPRSFELLKPPPICLRDSIKVINKEGEVMDVFRCDHCHVLFLDYVMFTIHMGCHGFRD

PFECNMCGYRSHDRYEFSSHIARGEHRAMLK

>Xenopus tropicalis ZNFN1A3

FLDADTSMDSMSSQDQPEPADLTVCSAKRNQEAEPNDDVFQKEEAAKHMDQVSPQGAKDD

GAEDMVKIKDEFCSVNKEPMEVESLDPAERTDLAYDYGSDCGPYDSIQLARHAAPYDMSR

PGLAKLYCDICGLACNSLNVLLVHKRSHTGERPFQCNQCGASFTQKGNLLRHGKLHTGEK

PFKCHLCSYACQRRDALTGHLRTHSVEKPYKCEFCGRSYKQRSSLEEHKERCRIYQQNLS

ESATAASEDSRPPKPEMGSERALVLDRLASNVAKRKSTMPQKFIGEKQPCFEISYNGYLY

DKEHEMMAARIMEQNIGATMAYVNPEVLRPMVHTPAVPTAEMVSVISSLYPVPLTRVEIP

NGHVTGQLMKEKRSVPDRVRSPIPSAQDSTDTDSSHEESSFPPTLTA

>Canis familiaris ZNFN1A3

DIKPNVELKSSQEESMPTECPVILNDYDLTKPHETENVDSAEGPTNEDEDLGDDSMKVKD

EYSERDENILKPEPMGNAEEPEIPYSYSREYNEYENVKLERHVVSYDSSRPTSGKMNCDV

CGLSCISFNVLMVHKRSHTGERPFQCNQCGASFTQKGNLLRHIKLHTGEKPFKCHLCNYA

CQRRDALTGHLRTHSVEKPYKCEFCGRSYKQRSSLEEHKERCRTFLQSTDLGETASAEAR

HIKAEMGSERALVLDRLASNVAKRKSSMPQKFIGEKRHCFDVNYNPSYMYEKESEIIQTR

IMDQAINNAISYLGAEALRPLVQTPPAPTSEMVPVISSMYPIALTRAEMPNGNPQDLEKK

NIHLPEKSLPSERGLSPNNSGHDSTDTDSNHEERQNHIYQQNHMVPPRARNGMPLLKEIP

RSYELLKPPPICPRDSIKVINKEGEVMDVYRCDHCRVLFLDYVMFTIHMGCHGFRDPFEC

NMCGYRSHDRYEFSSHIARGEHRAMLK

>Bos taurus ZNFN1A3

VSDIKPNVELKSTQEQSVPTDDSMKVKDEYSERDENVLKPEPMGNAEEPEIPYSYSREYN

EYENIKLERHVVSYDSSRPTSGKMNCDVCGLSCISFNVLMVHKRSHTGERPFQCNQCGAS

FTQKGNLLRHIKLHTGEKPFKCHLCNYACQRRDALTGHLRTHSVEKPYKCEFCGRSYKQR

SSLEEHKERCRTFLQSTDLGETASVEARHIKAEMGSERALVLDRLASNVAKRKSSMPQKF

IGEKRHCFDVSYNPSYMYEKESEMIQTRMMDQAINNAISYLGAEALRPLVQTPPAPTSEM

VPVISSMYPIALTRAEMPNGAPQELEKKNIHLPEKSLPSERGLSPTNSGHDSTDTDSNHE

ERQNHIYQQNPMVPPRARNGMPLLKEGPRSYDLLKPPPICPRDSIKVINKEGEVTDVYRC

DHCRVLFLDYVMFTIHMGCHGFRDPFECNMCGYRSHDRYEFSSHIARGEHRAMLK

>Homo sapiens ZNFN1A4

AMDSRYLQLQLYLPSCSLLQGSGDSSLEKEFLGAPVGPSVSTPNSQHSSPSRSLSANSIK

VEMYSDEESSRLLGPDERLLEKDDSVIVEDSLSEPLGYCDGSGPEPHSPGGIRLPNGKLK

CDVCGMVCIGPNVLMVHKRSHTGERPFHCNQCGASFTQKGNLLRHIKLHSGEKPFKCPFC

NYACRRRDALTGHLRTHSVSSPTVGKPYKCNYCGRSYKQQSTLEEHKERCHNYLQSLSTE

AQALAGQPGDEIRDLEMVPDSMLHSSSERPTFIDRLANSLTKRKRSTPQKFVGEKQMRFS

LSDLPYDVNSGGYEKDVELVAHHSLEPGFGSSLAFVGAEHLRPLRLPPTNCISELTPVIS

SVYTQMQPLPGRLELPGSREAGEGPEDLADGGPLLYRPRGPLTDPGASPSNGCQDSTDTE

SNHEDRVAGVVSLPQGPPPQPPPTIVVGRHSPAYAKEDPKPQEGLLRGTPGPSKEVLRVV

GESGEPVKAFKCEHCRILFLDHVMFTIHMGCHGFRDPFECNICGYHSQDRYEFSSHIVRG

EHKVG

>Mus musculus ZNFN1A4

MESLFCESSGDSSLEKEFLGAPVGPSVSTPNSQHSSPSRSLSANSIKVEMYSDEESSRLL

GPDERLLDKDDSVIVEDSLSEPLGYCDGSGPEPHSPGGIRLPNGKLKCDVCGMVCIGPNV

LMVHKRSHTGERPFHCNQCGASFTQKGNLLRHIKLHSGEKPFKCPFCNYACRRRDALTGH

LRTHSVSSPTVGKPYKCNYCGRSYKQQSTLEEHKERCHNYLQSLSTDAQALTGQPGDEIR

DLEMVPDSMLHPSTERPTFIDRLANSLTKRKRSTPQKFVGEKQMRFSLSDLPYDVNASGG

YEKDVELVAHHGLEPGFGGSLAFVGTEHLRPLRLPPTNCISELTPVISSVYTQMQPIPSR

LELPGSREAGEGPEDLGDGGPLLYRARGSLTDPGASPSNGCQDSTDTESNHEDRIGGVVS

LPQGPPPQPPPTIVVGRHSPAYAKEDPKPQEGLLRGTPGPSKEVLRVVGESGEPVKAFKC

EHCRILFLDHVMFTIHMGCHGFRDPFECNICGYHSQDRYEFSSHIVRGEHKVG

>Rattus norvegicus ZNFN1A4

MESLFCESSGDSSLEKEFLGAPVGPSVSTPNSQHSSPSRSLSANSIKVEMYSDEESSRLL

GPDERLLDKDDSVIVEDSLSEPLGYCDGSGPEPHSPGGIRLPNGKLKCDVCGMVCIGPNV

LMVHKRSHTGERPFHCNQCGASFTQKGNLLRHIKLHSGEKPFKCPFCNYACRRRDALTGH

LRTHSVSSPTVGKPYKCNYCGRSYKQQSTLEEHKERCHNYLQSLSTDAQTLAGQPGDEIR

DLEMVPDSMLHPSTERPTFIDRLANSLTKRKRSTPQKFVGEKQMRFSLSDLPYDVNPSGG

YEKDVELVAHHGLEPGFGGSLAFVGTEHLRPLRLPPTNCISELTPVISSVYTQMQPLPSR

LELPGSREAGEGPEDLGDGGPLLYRARGSLTDPGASPSNGCQDSTDTESNHEDRIGGVVS

LPQGPPPQPPPTIVVGRHSPAYAKEDPKPQEGLLRGTPGPSKEVLRVVGESGEPVKAFKC

EHCRILFLDHVMFTIHMGCHGFRDPFECNICGYHSQDRYEFSSHIVRGEHKVG

>Xenopus tropicalis ZNFN1A4

GKLKCDICGMVCIGPNVLMVHKRSHTGERPFHCNQCGASFTQKGNLLRHIKLHSGEKPFK

CPFCNYACRRRDALTGHLRTHAVGKPYKCNYCGRSYKQQNTLEEHKERCHNYLQSLSNEA

QHLPAHPGEWEPQGGDSMYSFMDRLACNLTKRKRSTPQKFMGQKSFFFCFFFYPFSFEKD

VEIVSHHPLDSAYSNSLAFVGGPMRLPPTNCISEITPVISSVYTQLQPMQGRPDMPGNRE

AAEGHEDIPDGTQIHYRGRSEHGASPTNGCQDSTTDTESNHEERVSQATSSRQSPAYAKE

DQRPSDGGLVLPSRSMPGTAKESLRVLGENGEQVKVFKCEHCRVLFLDHVMFTIHMGCHG

FRDPFECNICGYHSQDRYEFSSHIVRGEHKV

>Canis familiaris ZNFN1A4

MHTPPALPRRFQGGGRARTPGSHRRGKDNLERDPSGGCVPDFLPQAQDSNHFIMESLFCE

SSGDSSLEKEFLGAPVGPSVSTPNSQHSSPSRSLSANSIKVEMYSDEESSRLLGPDERLL

EKDDSVIVEDSLSEPLGYCDGSGPEPHSPGGIRLPNGKLKCDVCGMVCIGPNVLMVHKRS

HTGERPFHCNQCGASFTQKGNLLRHIKLHSGEKPFKCPFCNYACRRRDALTGHLRTHSVS

SPTVGKPYKCNYCGRSYKQQSTLEEHKERCHNYLQSLSTEAQALAGQPGDEIRDLEMVPD

SMLHSSSERPTFIDRLANSLTKRKRSTPQKFVGEKQMRFSLSDIPYDVNSGGYEKDVELV

AHHGLEPGFGGSLAFVGAEHLRPLRLPPTNCISELTPVISSVYTQMQPLPGRLELPGSRE

AGEGPEDLADGGPLLYRARGPLTDPGASPSNGCQDSTDTESNHEDRVGGVVSLPQGPPPQ

PPPTIVVGRPSPAYAKEDPKPQEGLLRGTPGPSKEVLRVVGESGEPVKAFKCEHCRILFL

DHVMFTIHMGCHGFRDPFECNICGYHSQDRIHLIPRLLGPVLRISGREKGVSFLSSNFQY

NQNYPRTRKGICPSPHSILVLARMGWAQL

>Bos taurus ZNFN1A4

ANSIKVEMYSDEESSRLLGPDERLLEKDDSVIVEDSLSEPLGYCDGSGPEPHSPGGIRLP

NGKLKCDVCGMVCIGPNVLMVHKRSHTGERPFHCNQCGASFTQKGNLLRHIKLHSGEKPF

KCPFCNYACRRRDALTGHLRTHSVSSPTVGKPYKCNYCGRSYKQQSTLEEHKERCHNYLQ

SLSTEAQALAGQSGDEIRDLEMVPDSMLHSSSERPTFIDRLANSLTKRKRSTPQKFVGEK

QMRFSLSDLPYDVNSGGYEKDVELVAHHGLEPGFGGSLAFVGAEHLRPLRLPPTNCISEL

TPVISSVYTQMQPLPGRLELPGSREAGEGPEDLADGGPLLYRARGPLTDPGASPSNGCQD

STDTESNHEDRVGGVVSLPQGPPPQPPPAVVVGRPSPAYAKEDPKPQEGLLRGTPGPSKE

VLRVVGESGEPVKAFKCEHCRILFLDHVMFTIHMGCHGFRDPFECNICGYHSQDRYEFSS

HIVRGEHKYKFS

>Strongylocentrotus purpuratus (XP_790671)

MEKLYYSSSGPLEPSPYRPRSPPCGPGERTLIPSHSLPAHSSLPGYDSNHTPVHLSAYNT

GPQPMKNLSLYPESDFTRPQTNHMTTLHSPVMLPRKPLSDYPLSGVSNIHHHYHHQPPQP

VDNPSHHGAVLPYSDLLREGYGVQGSDQQGYGGSWQKSPTLNGTSGILPCSDVLPHHQPN

FLNSSSAAAARSQSAHSPGHHTNHPTVHPNHQQVSPFVSTASTPTSVGGATYEDYASKLR

SSADESRFSLPYTPTPPAWRTNHHHHASQPSVPPPPPPPPLYFCGRMVQVRSNKNKSEHK

KSSNQCNICGKSYARPSTLKTHMRTHSGEKPYHCPTCQKSFSQTANLTAHMRTHSGEKPF

RCLICNRQFSQSSSVTTHMRTHSGERPYRCAYCRKAFADSSTLTKHLRIHSGEKPYQCKI

CLLRFSQSGNLTRHMKVHKNE
